# IRE1 activity is prognostic in localised and metastatic prostate cancer and affects epithelial lineage states

**DOI:** 10.1038/s44321-026-00490-w

**Published:** 2026-08-22

**Authors:** Dimitrios Doultsinos, Ingrid Tomljanovic, Eleftherios Pilalis, Sophia M Abusamra, Mohammed Alshalalfa, Romuald Parmentier, Vidhula Ahire, Imogen E Bridges, Sandy Figiel, Joanna Hester, Damien Leach, Ashwin Nandakumar, Rensheng Wan, Yanis Zekri, Jichang Zhang, Charlotte L Bevan, Edward O’Neill, Alastair D Lamb, Valentine Macauley, Jean Phillipe Theurillat, Alfonso Urbanucci, R Jeffrey Karnes, Daniel E Spratt, Elai Davicioni, David Quigley, Wilbert Zwart, Clementine Le Magnen, Aristotelis Chatziioannou, Ian G Mills

**Affiliations:** 1 Nuffield Department of Surgical Sciences, University of Oxford, Oxford, UK; 2 Department of Urology, Erasmus MC Cancer Institute, Erasmus University Medical Center, Wytemaweg 80, 3015 CN Rotterdam, the Netherlands; 3 e-NIOS PC, Kallithea-Athens, Greece; 4 Veracyte, South San Francisco, CA USA; 5 Institute of Medical Genetics and Pathology, University Hospital Basel, Basel, Switzerland; 6 Department of Urology, University Hospital Basel, Basel, Switzerland; 7 Department of Biomedicine, University of Basel, University Hospital Basel, Basel, Switzerland; 8 Imperial College London, London, UK; 9 Department of Urology, UCSF, San Francisco, CA USA; 10 Division of Oncogenomics, The Netherlands Cancer Insitute, 1066 CX Amsterdam, The Netherlands; 11 Oncode Institute, Amsterdam, The Netherlands; 12 Faculty of Biomedical Sciences, Institute of Oncology Research, USI, Bellinzona, TI 6500 Switzerland; 13 Centre for Cancer Evolution, Barts Cancer Institute, Queen Mary University of London, London, UK; 14 Center for Cancer Eradication Research, Faculty of Medicine and Health Technology, Tampere University and Tays Cancer Center, Tampere, Finland; 15 Department of Tumor Biology, Institute for Cancer Research, Oslo University Hospital, Oslo, Norway; 16 Mayo Clinic Dept of Urology, Rochester, MN USA; 17 University Hospitals Seidman Cancer Center, Case Western Reserve University, Cleveland, OH USA; 18 Helen Diller Family Comprehensive Cancer Center, University of California, San Francisco, CA USA; 19 Department of Epidemiology and Biostatistics, UCSF, San Francisco, CA USA; 20 Center of Systems Biology, Biomedical Research Foundation of the Academy of Athens, Athens, Greece

**Keywords:** Biomarkers, Cancer, Chromatin, Transcription & Genomics

## Abstract

Prostate cancer (PCa) is an androgen receptor (AR) driven, high-incidence disease significantly contributing to cancer mortality. To improve treatment outcomes for patients at high risk of metastasis, PCa is in need of better risk stratification at diagnosis. The unfolded protein response (UPR) is an AR-dependent process. However, the impact of the UPR transducer IRE1 on AR-dependent biology and acquired treatment resistance has not been defined. We use pre-clinical models of stress response to describe the impact of IRE1 activity loss on multiple PCa stages and demonstrate its involvement with poor prognosis (RB1 loss), and cell lineage determination (club-like phenotypes). Integrating clinical transcriptomic datasets, we chart IRE1 activity throughout PCa evolution and develop an IRE1 activity signature (IRE1sig1.0) reflecting both tumoral and micro-environmental stress responses. IRE1sig1.0 correlates with tumoral identity, and prognosticates localised and metastatic disease independently from AR activity. Using IRE1sig1.0 as a tool may inform ARSI suitability and guide IRE1 modulation as a novel combination therapeutic in prostate cancer.

## Synopsis

Combined IRE1-induced and IRE1-repressed transcriptional programs were investigated as a proxy for prostate cancer progression towards treatment resistance, revealing associations with features of RB1 loss and club cell phenotypes.

- IRE1 activity was suppressed by androgen deprivation therapy.
- IRE1 activity loss led to decreased RB1 and increased RBL1 signalling.
- IRE1 activity loss promoted lineage reprogramming, with partial deregulation of luminal and basal programs, reduced tumoural characteristics, and increased club cell features.
- Integrated analysis of AR- and IRE1-perturbed bulk RNA-seq datasets from preclinical models, refined using clinical bulk, single-cell, and spatial transcriptomic datasets, generated the IRE1 activity signature IRE1sig1.0.
- IRE1sig1.0 captured tumour grade and NCCN risk group, distinguished between prostate cancer subtypes, and was prognostic in both localised and metastatic disease.

## Abstract (web summary)

Combined IRE1-induced and IRE1-repressed transcriptional programs were investigated as a proxy for prostate cancer progression towards treatment resistance, revealing associations with features of RB1 loss and club cell phenotypes.

## Graphical abstract

**Figure:**
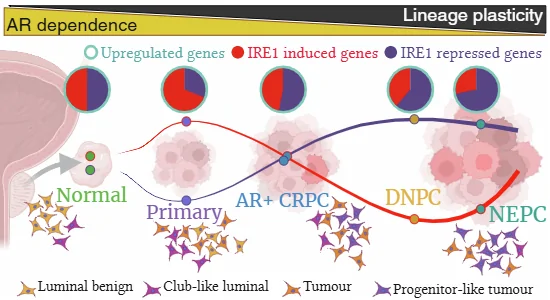

**Figure:**
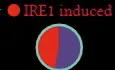

The paper explained **Problem**

Solid tumours such as prostate cancer (PCa) develop and progress in a hostile microenvironment characterised by nutrient deprivation, poor vascularisation, inflammation and hypoxia. These stresses are further exacerbated by treatments such as surgery, radiotherapy, and chemotherapy. While many tumour cells fail to survive under these conditions, some adapt by becoming independent of androgen receptor (AR) signalling and acquiring resistance to therapy. This adaptation is driven, in part, by the unfolded protein response (UPR), a cellular stress response that promotes survival and facilitates changes in tumour cell lineage.

**Results**

We investigated the role of the UPR sensor IRE1 during prostate cancer (PCa) progression. Low IRE1 activity correlated with increased disease severity, and both IRE1-induced and IRE1-repressed transcriptomic programmes were markedly altered in treatment-resistant disease. Suppressed IRE1 activity in AR-positive tumour cells triggered cell cycle rewiring and lineage reprogramming, promoting features associated with RB1 loss and a transition toward a club cell-like state, a plastic epithelial population linked to androgen deprivation, immune evasion, and treatment resistance. These findings identify IRE1 as a potential regulator of lineage plasticity and acquired therapeutic resistance. To capture these changes, we developed an IRE1 activity signature (IRE1sig1.0) by integrating multiple pre-clinical and clinical datasets and filtering them through clinical single-cell and spatial transcriptomic datasets. This approach ensured that IRE1sig1.0 is a proxy for multiple biological processes and cell types. This multicellular signature predicts metastatic and prostate cancer-specific survival, correlates with histological grade, and captures multiple clinically relevant features of disease progression.

**Impact**

This work improves our understanding of how prostate cancers become resistant to treatment, which may help inform future treatment decisions. Furthermore, it provides a way to track these changes and identify whether activating or inhibiting IRE1 could restore sensitivity to existing therapies. In the long term, this strategy could improve treatment effectiveness while reducing the need for repeated toxic therapies and improving patients’ quality of life.

## Introduction

Prostate cancer (PCa) is a heterogenous, multifocal, high-incidence disease that affects one in eight men globally. PCa evolution depends on transcriptional events and epigenetic modifications, forming an adaptive response to intrinsic/extrinsic stresses, culminating in lineage reprogramming and treatment resistance (Doultsinos and Mills, 2020). PCa results in ~360,000 deaths annually (Rawla, 2019) due to metastatic progression, supported by treatment/stress-resistant cancer cells which experience high proliferative rates and androgen receptor (AR)-driven high metabolic demand.

These events require high protein turnover, leading to misfolded protein accumulation, causing endoplasmic reticulum (ER) stress. The unfolded protein response (UPR) resolves this stress through three main transducers: inositol-requiring enzyme alpha (IRE1α), protein kinase RNA-like endoplasmic reticulum kinase (PERK), and activating transcription factor 6α (ATF6). IRE1α, hereafter referred to as IRE1 and encoded by the gene *ERN1*, is the most evolutionarily conserved transducer of the UPR and signals through two distinct downstream functional outputs (Almanza et al, 2019). Firstly, through the unconventional splicing of the transcript encoding XBP1, a transcription factor, resulting in a non-canonical splice variant lacking a 60-nucleotide sequence, which encodes the active protein (XBP1s). Secondly, through the targeted degradation/decay (RIDD) of a subset of mRNA (e.g. *DGAT2* (Almanza et al, 2022), *BLOC1S1* (Bright et al, 2015)) and miRNA transcripts to ease transcriptional load (Raymundo et al, 2020). Both functions require IRE1 endoribonuclease activity and can be thought of as a continuum of early (XBP1s) and late (RIDD) overlapping responses that are designed to adapt to stress. IRE1 is a type II transmembrane dual serine/threonine kinase and endoribonuclease. The endoribonuclease effector function depends on autophosphorylation kinase-driven conformational changes. Mutations affecting IRE1 activity have previously been described, including a truncation mutation at tryptophan 780, which produces an IRE1 protein without a ribonuclease domain and, as such, no catalytic activity (Lhomond et al, 2018).

Using short (up to 24 h) pre-clinical model treatments, we have previously shown that the IRE1-XBP1 axis comprises AR-induced genes, notably *ERN1* itself (Sharma et al, 2013). Targeting IRE1 in androgen-dependent pre-clinical models can restrain tumorigenesis (Sheng et al, 2019). However, it remains unclear which IRE1-dependent genes or biological processes underpin these responses and how IRE1 activity may change in response to sustained stress, reflecting PCa evolution. Most studies so far have focused on the role of IRE1 as an activator of XBP1 impacting tumour development (Sheng et al, 2015). However, IRE1-RIDD may impact RNA stability, which could be restraining the emergence of cancers that are more difficult to treat.

In this study, we generated bulk RNAseq data from cell lines, patient-derived explants (PDEs) and prospectively collected patient radical prostatectomy (RP) samples. We integrated these with single-cell (SC) and spatial (ST) RNA sequencing data derived from further prospectively collected patient radical prostatectomy (RP) and biopsy samples. We observed that adaptation to IRE1 loss correlated with transcriptomic repertoires usually driven by prognostically poor genomic alterations such as RB1 loss. In parallel, we observed that IRE1-deficient tumour cells exhibited a club cell-resembling phenotype (Gerhauser et al, 2018; Karthaus et al, 2020). Club cells are benign cells which interact with immunosuppressive microenvironmental myeloid cells, can be induced by androgen deprivation and have reprogramming capacity.

Big transcriptomic data integration-driven generation of transcriptome-wide expression classifiers (AR_Spratt, Decipher) have shown increased prognostic power in localised disease (Grist et al, 2025). To document IRE1 activity throughout PCa evolution, we integrated our multifaceted datasets to produce a novel IRE1 activity signature, IRE1sig1.0, representing significant ERN1 transcriptomic perturbations in PCa-specific models. IRE1sig1.0 is a proxy of multiple signalling pathways linked to PCa progression and can chart PCa evolution from early stage to treatment-resistant disease while prognosticating large clinical cohorts. Current clinical practices impact therapeutic effectiveness on PCa mortality (Hamdy et al, 2023). Therefore, using IRE1 activity to identify tumours with treatment resistance traits early in the diagnostic pathway could be transformative for patient care. Tumoral IRE1 activity could be used to stratify patients for therapy intensification or surveillance while testing the suitability of IRE1/UPR modulation as novel combination therapeutics alongside conventional treatments.

## Results

### IRE1 activity loss correlates with androgen deprivation-resistant phenotypes

**Figure 1:**
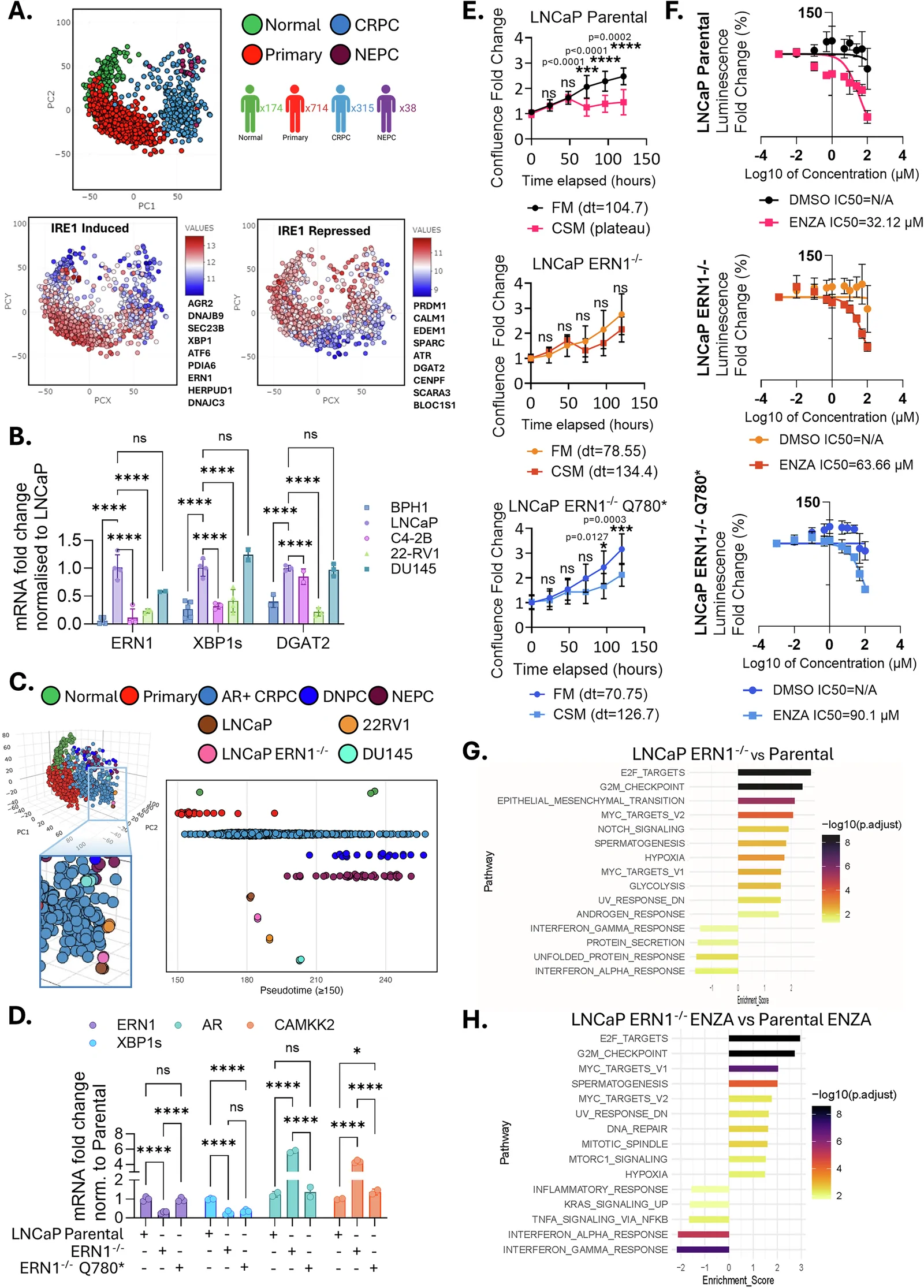
IRE1 activity loss correlates with androgen deprivation-resistant phenotypes. (**A**) IRE1 activity transcriptomic signatures in a comprehensive prostate cancer transcriptome atlas of integrated RNA-seq datasets from over 1000 clinical tissue samples ranging from benign (green) to NEPC (purple). Heatmaps show the distribution of signal (low/blue; high/red) of these signatures across the dataset. (**B**) mRNA fold change of *ERN1*, *XBP1s*, *DGAT2* in BPH1, C4-2B, 22RV1 and DU145 cells normalised to parental LNCaP in normal physiological conditions (*n* = 4). (**C**) Whole transcriptomes of parental LNCaP, LNCaP ERN1^−/−^, 22RV1 and DU145 cells in the Prostate Cancer Atlas. (**D**) mRNA fold change of androgen receptor activity markers (full-length *AR*, *CAMKK2*) and IRE1 activity markers (*ERN1*, *XBP1s*) in LNCaP parental, LNCaP ERN1−/− and ERN1−/− Q780* cells, normalised to parental LNCaP (*n* = 3). (**E**) Growth curves of LNCaP parental, LNCaP ERN1−/− and LNCaP ERN1−/− Q780* cells grown over 120 h in FM or CSM. Confluence fold change was normalised to Incucyte readings taken at the 0-h time point of each condition. Exponential (Malthusian) growth curve equations were used to estimate the doubling time of each cell line in FM or CSM (*n* = 3). Statistical analysis was performed using an ordinary one-way ANOVA with Dunnett’s multiple comparisons test and single-pooled variance. (**F**) Enzalutamide (ENZA) concentration gradient measuring cytotoxicity through ATP MTT assays in LNCaP parental, LNCaP ERN1^−/^^−^ and LNCAP ERN1^−/−^ Q780* cells treated for 12 h with increasing concentrations of ENZA. Logarithmic concentration vs normalised response curves were fit to calculate the IC50 of ENZA for each cell line. GSEA normalised enrichment scores (NES) for significantly enriched MSigDB Hallmark gene sets in LNCaP ERN1−/− cells compared to LNCaP parental cells grown in FM (**G**) and LNCaP ERN1−/− cells treated with ENZA compared to LNCaP parental cells treated with ENZA (**H**). Bar length indicates the normalised enrichment score (NES) and bar colour signifies the statistical significance. Statistical tests and *p* values associated with this figure can be found in Dataset EV33. Data information: Data are presented as mean ± SD. ns not significant, *P ≤ 0.05, **P ≤ 0.01, ***P ≤ 0.001, ****P ≤ 0.0001. Source data are available online for this figure.

To correlate dynamic changes in IRE1 activity status with PCa disease progression and AR status, we assessed AR/IRE1 activity genes in a compendium of integrated RNA-seq datasets comprising over 1000 clinical tissue samples ranging from benign to double negative (DNPC) and neuroendocrine prostate cancer (NEPC) (Data Ref: Prostate Cancer Atlas (Bolis et al, 2021)). We split these genes into “induced” (downregulated by AR/IRE1 activity loss) and “repressed” (upregulated by AR/IRE1 activity loss) groups. We observed that compared to normal, IRE1 activity increased in primary disease while steadily decreasing in AR-positive castration resistant prostate cancer (CRPC) and NEPC (Fig. 1A). In contrast, AR activity was similar across normal, primary and AR + CRPC samples before dramatically decreasing in NEPC (Appendix Fig. S1A). In PCa cell lines, we observed that IRE1 expression and activity (XBP1s/DGAT2) were decreased in AR + CRPC (C4-2B, 22RV1) and AR-ve BPH (BPH1) while unchanged in AR-ve CRPC (DU145) (Fig. 1B).

**Figure EV1:**
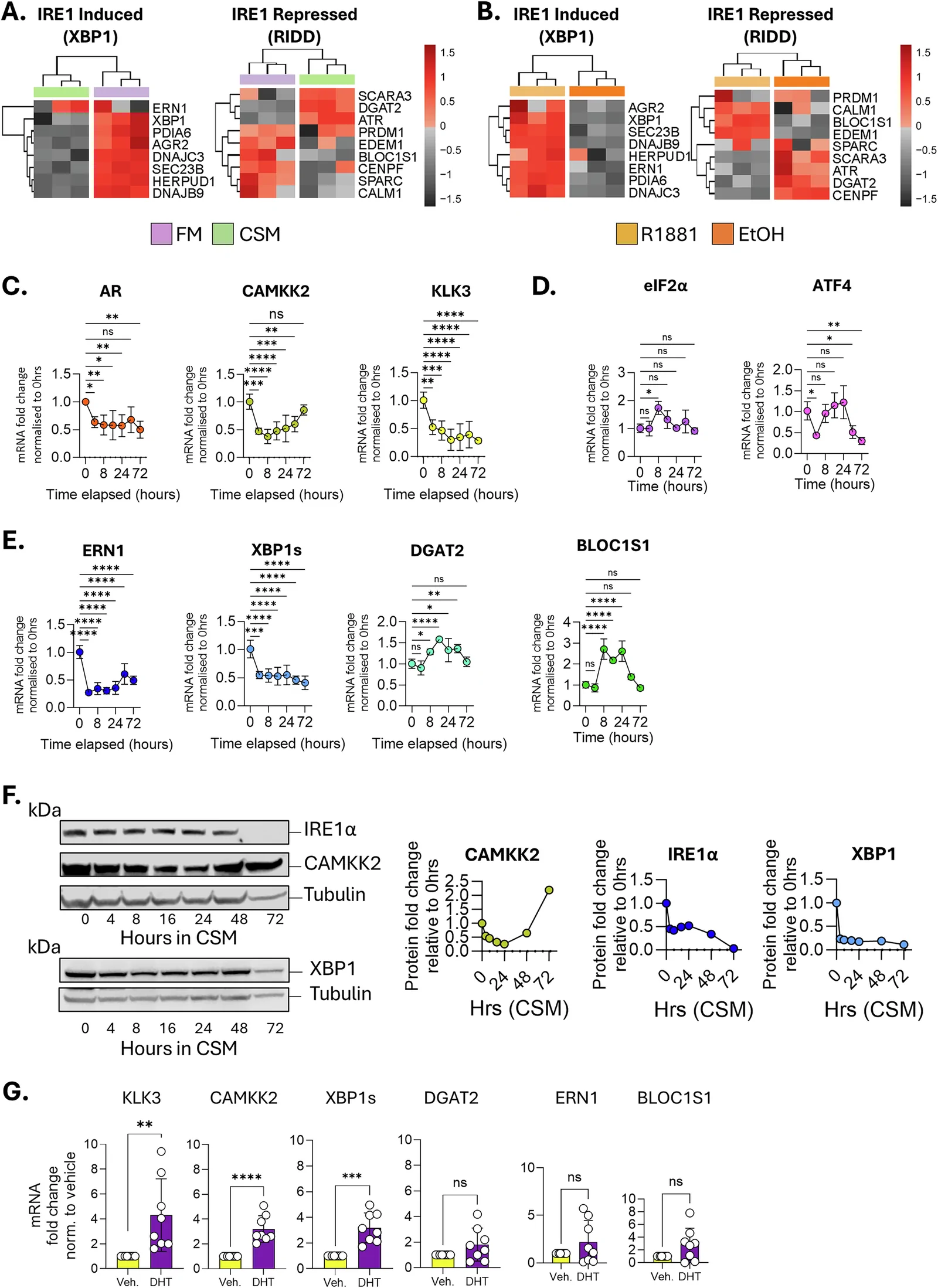
IRE1 activity is suppressed by physiological Androgen deprivation. (**A**, **B**) Expression heatmaps of selected “IRE1 induced” and “IRE1 repressed” genes according to the literature in LNCaP cells in CSM (green) vs FM (purple), (**A**); and R1881 (yellow) vs EtOH (orange) (**B**). The colour scale represents the expression z-score from black (lowest) to red (highest). (**C**–**E**) mRNA fold change of androgen receptor activity markers (full-length AR, CAMKK2 and KLK3), IRE1 activity markers (ERN1, XBP1s, DGAT2 and BLOC1S1), and PERK activity markers (eIF2α and ATF4) in LNCaP parental cells grown over 72 h in CSM, normalised to untreated FM control (*n* = 3). Statistical analysis was performed using an ordinary one-way ANOVA with Dunnett’s multiple comparisons test and single-pooled variance. (**F**) Blots of lysates derived from LNCaP cells grown in CSM for 72 h showing protein levels of IRE1α, CAMKK2, XBP1 and Tubulin with accompanying densitometry. Each time point was normalised to the housekeeping tubulin, and then the values were normalised to the 0-h time point for each of IRE1, XBP1 and CAMKK2. (**G**) mRNA fold change of AR and IRE1 activity markers KLK3, CAMKK2, XBP1s, DGAT2, ERN1, BLOC1S1 in patient-derived explants (PDEs) treated with dihydrotestosterone (DHT), normalised to PDEs treated with vehicle control (*n* = 8). Statistical analysis was performed using a *T*-test per condition. Data information: Data were presented as mean ± SD. ns not significant, *P ≤  0.05, **P ≤ 0.01, ***P ≤ 0.001, ****P ≤ 0.0001.

We cultured LNCaP cells in charcoal-stripped serum media (CSM) or full serum media (FM) for 72 h or treated with the synthetic androgen R1881 or EtOH for 12 h. We observed that AR-induced physiological stresses included the UPR in line with previous observations (Sheng et al, 2015) (Appendix Fig. S1B–E Datasets EV1 and2)). However, the UPR Hallmark is not IRE1 specific, containing target genes of all UPR transducers (ATF4, ATF6 and XBP1s), while RIDD may not be represented within it. Consequently, we used a curated list of XBP1, and RIDD target genes to correlate alterations in AR signalling with IRE1 activity. Whereas XBP1 target genes were largely androgen-induced, RIDD targets were not consistently androgen dependent (Fig. EV1A,B; Datasets EV3 and E4). CSM culture of LNCaP compared to FM showed a decrease in IRE1 activity (*XBP1s* downregulated, RIDD targets *DGAT2*, *BLOC1S1* upregulated) as early as 8 h, mirroring full-length AR and CAMKK2/KLK3 expression. *ATF4* and *eIF2α* either did not significantly or consistently change in the same timeframe (Fig. EV1C–F). These observations persisted in patient-derived explants (PDEs)(Centenera et al, 2018) treated with, or deprived of, dihydrotestosterone (DHT) for 12 h, despite their multicellularity and heterogeneity (Fig. EV1G).

**Figure EV2:**
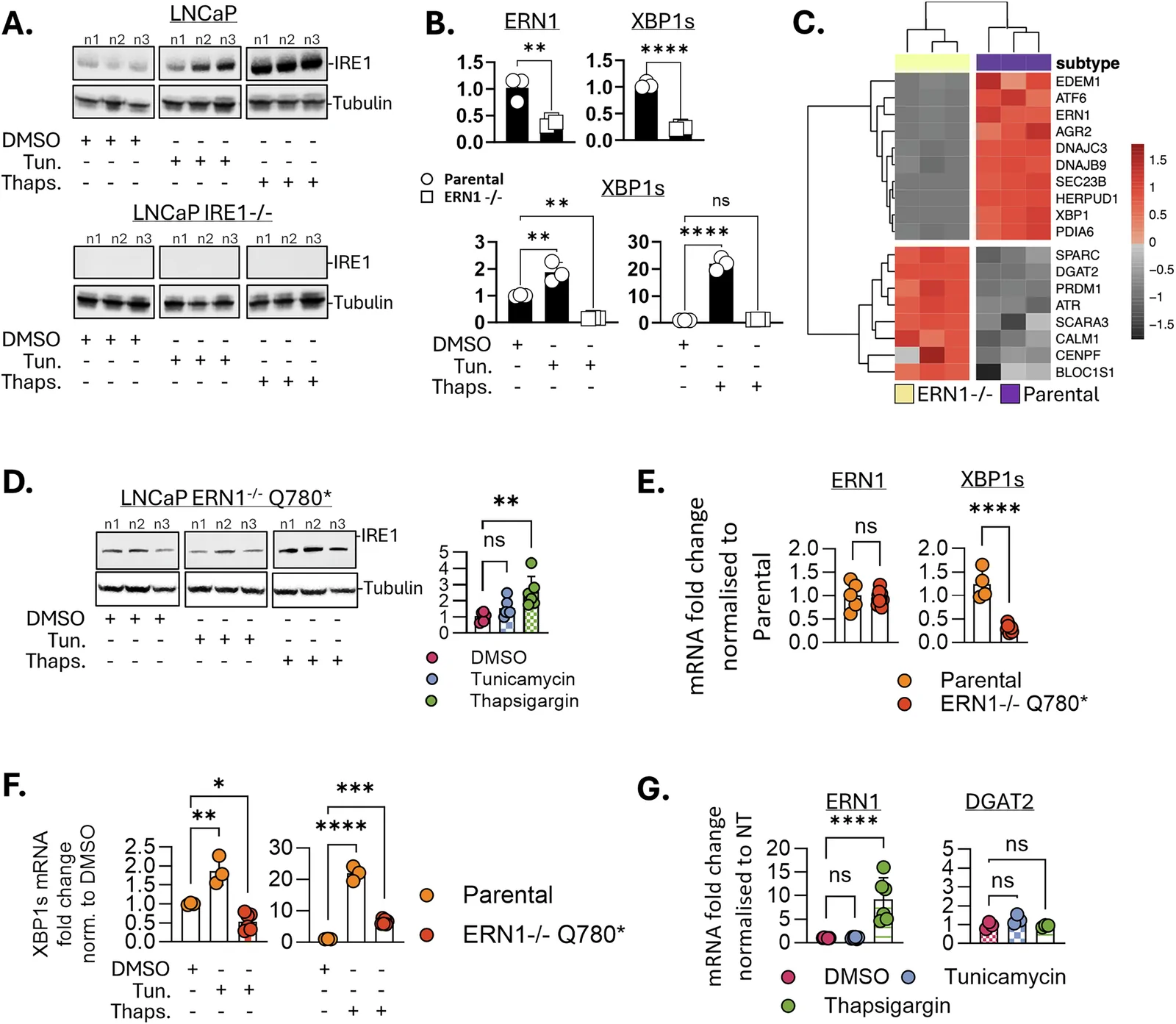
LNCaP IRE1 deficient cells have limited ER Stress response. (**A**) Western blots in triplicate for IRE1α of LNCaP parental (top) or LNCaP ERN1−/− cells (bottom) in the presence of ER stressors tunicamycin or thapsigargin (100 nM). The DMSO control is also shown. (**B**) mRNA fold change of ERN1, XBP1s in basal conditions (FM, top) or in the presence of tunicamycin or thapsigargin (bottom) in LNCaP parental or LNCaP ERN1−/− cells (*n* = 3). Top: Statistical analysis was performed using T-tests. Bottom: Statistical analysis was performed using an ordinary one-way ANOVA with Dunnett’s multiple comparisons test and single-pooled variance. (**C**) Heatmap of canonical IRE1-XBP1 or IRE1-RIDD targets in RNAseq data derived from LNCaP parental (purple) or LNCaP ERN1−/− (yellow) cells. (**D**) Western blots in triplicate for IRE1α of LNCaP ERN1−/− Q780* cells in the presence of ER stressors tunicamycin or thapsigargin (100 nM). The DMSO control is also shown. Accompanying densitometry measures the impact of ER stress on the protein levels of the IRE1 mutant protein. (**E**) mRNA fold change of ERN1 and XBP1s in LNCaP parental and LNCaP ERN1−/− Q780* cells under basal conditions (FM) (*n* = 6). Statistical analysis was performed using a *T*-test per condition. (**F**) IRE1 induced activity marker XBP1s mRNA fold change in LNCaP parental or LNCaP ERN1−/− Q780* cells treated with DMSO, Tunicamycin or Thapsigargin, normalised to parental cells treated with DMSO (*n* = 3). Statistical analysis was performed using an ordinary one-way ANOVA with Dunnett’s multiple comparisons test and single-pooled variance. (**G**) IRE1 repressed activity marker DGAT2 and ERN1 mRNA fold change in LNCaP ERN1−/− Q780* cells treated with DMSO, Tunicamycin or Thapsigargin, normalised the DMSO condition (*n* = 6). Statistical analysis was performed using an ordinary one-way ANOVA with Dunnett’s multiple comparisons test and single-pooled variance. Data information: Data were presented as mean ± SD. ns not significant, *P ≤ 0.05, **P ≤ 0.01, ***P ≤ 0.001, ****P ≤ 0.0001. Source data are available online for this figure.

We then investigated the adaptation of AR-positive cells (LNCaP) to stable IRE1 perturbation, using an IRE1 CRISPR-knockout (LNCaP ERN1^−/−^) (Stelloo et al, 2020), constitutively lacking IRE1 at steady state and IRE1 activity under chemically induced ER stress (tunicamycin/thapsigargin) (Fig. EV2A–C). We began characterising these cells by putting them in the context of the Prostate Cancer Atlas. Principal component 2 of the Atlas anticorrelates with canonical AR signalling. Consequently, it is expected that AR-ve or treatment-resistant models would shift away from treatment-sensitive models on that axis. We integrated whole transcriptomes of LNCaP parental, LNCaP ERN1^−/−^, 22RV1 and DU145 cells in the Atlas and intriguingly, IRE1 loss pushed LNCaP cells toward AR independence but not to the same extent of fully AR-independent 22RV1 and DU145 phenotypes (Fig. 1C).

To definitively link physiological observations to the absence of IRE1 catalytic activity, we stably expressed an IRE1 mutant in ERN1^−/−^ cells lacking the effector ribonuclease domain by a truncation mutation in tryptophan 780 (Lhomond et al, 2018). These newly generated ERN1^−/−^ Q780* cells were IRE1 activity-deficient both at steady state and under ER stress conditions (Fig. EV2D–G) while maintaining their AR levels. ERN1^-/-^ cells showed significant increases in full-length AR and CAMKK2 mRNA. Interestingly, re-introducing the truncated form of IRE1 reduced AR and CAMKK2 mRNA levels to those equivalent to LNCaP parental cells (Fig. 1D).

**Figure EV3:**
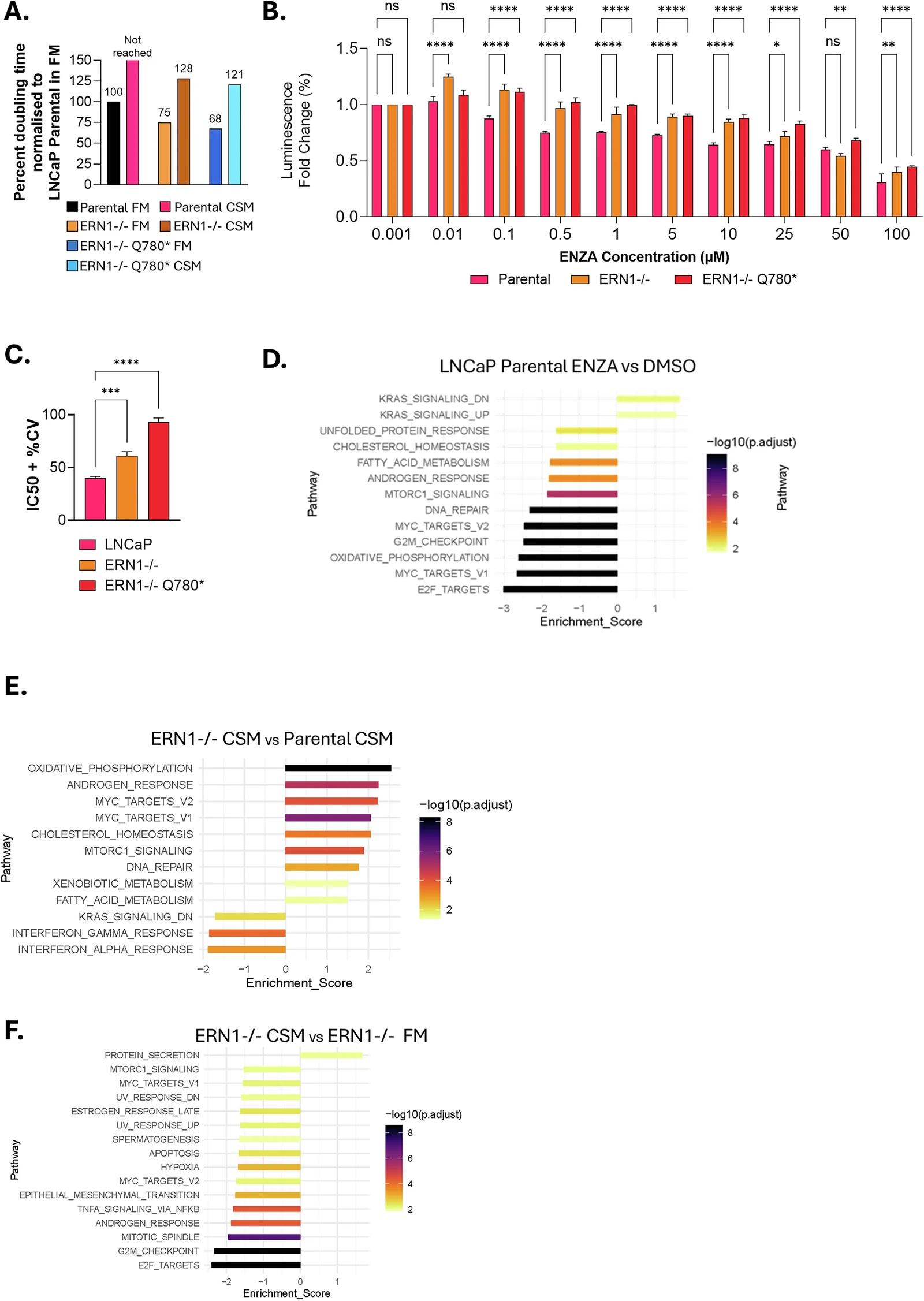
Response of IRE1 deficient LNCaP cells to Androgen deprivation compared to parental cells. (**A**) Growth curves of LNCaP parental, LNCaP ERN1^−^^/^^−^ and LNCaP ERN1−/− Q780* cells grown over 120 h in FM or CSM. Confluence fold change was normalised to Incucyte readings taken at the 0-h time point of each condition. Exponential (Malthusian) growth curve equations were used to estimate the doubling time of each cell line in FM or CSM. (**B**) Cell Titre Glo signal fold change in LNCaP, LNCaP ERN1−/− and LNCaP ERN1−/− Q780* cells treated with increasing doses of ENZA over 12 h (*n* = 3). All values are normalised to equivalent DMSO controls for each condition. Statistical analysis was performed using an ordinary one-way ANOVA with Dunnett’s multiple comparisons test and single-pooled variance. (**C**) Bar chart represents the ENZA IC50 comparison between the cell lines. Maximum variance is used to calculate significance by ordinary one-way ANOVA with multiple comparisons. (**D**) GSEA normalised enrichment scores (NES) of LNCaP parental cells treated with 10 μM ENZA for 12 h compared to DMSO or (**E**) of LNCaP ERN1−/− cells grown in CSM for 72 h compared to parental LNCaP cells grown in the same conditions or (**F**) of LNCaP ERN1−/− cells grown in CSM for 72 h compared to LNCaP ERN1−/− cells grown in FM for 72 h. Bar length indicates the normalised enrichment score (NES) and bar colour signifies the statistical significance. Data information: Data were presented as mean ± SD. ns not significant, *P ≤ 0.05, **P ≤ 0.01, ***P ≤ 0.001, ****P ≤ 0.0001. Source data are available online for this figure.

We then documented the growth of LNCaP, LNCaP ERN1^−/−^ and LNCaP ERN1^−/−^ Q780* in FM or CSM. ERN1^−/−^ and ERN1^−/−^ Q780* growth rates were not significantly affected by CSM while LNCaP cells stopped growing entirely after 48 h (Figs. 1E and EV3A). Similarly, increasing concentrations of the AR antagonist Enzalutamide (ENZA) on LNCaP, LNCaP ERN1^−/^^−^ and LNCaP ERN1^−/−^Q780* cells over short treatments (12 h) showed IRE1-deficient cells to be modestly more resistant to androgen deprivation than IRE1 naïve cells (Figs. 1F, and EV3B,C).

To define the biological functions underlying this ADT-resistant phenotype, we performed whole transcriptome pathway analysis showing enrichment for E2F targets, G2M checkpoint, and the EMT in ERN1^−^^/−^ cells (Fig. 1G; Dataset EV5). ENZA treatment of parental LNCaP suppressed E2F targets and the UPR (Fig. EV3D; Dataset EV6). Comparing ERN1^−^^/−^ to parental LNCaP treated with ENZA, we observed significant positive enrichment for E2F targets, G2M checkpoint and DNA repair (Fig. 1H; Dataset EV7), corroborating our CSM results. We then compared significantly altered Hallmarks induced by androgen deprivation with those altered upon IRE1 KO. ERN1^−^^/−^ cells were still sensitive to CSM as E2F targets, G2M checkpoint and AR Response were negatively enriched, while DNA repair, AR response and MTORC1 were positively enriched compared to ERN1^-/-^ cells grown in FM (Fig. EV3E,F; Datasets EV8; S9).

### IRE1 Activity influences cell cycle regulation while inducing changes in reprogramming markers

**Figure 2:**
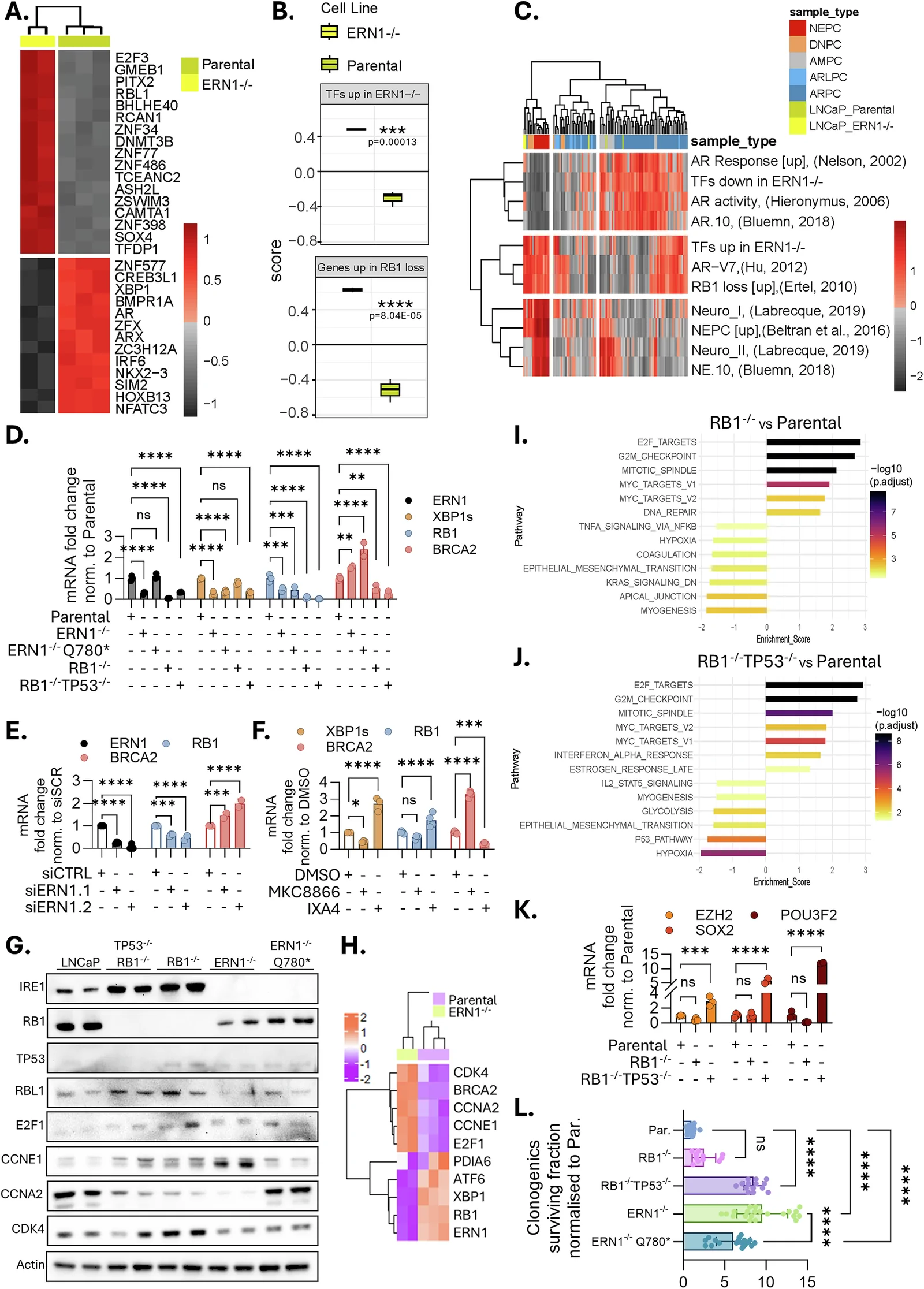
IRE1 activity influences cell cycle regulation while inducing changes in reprogramming markers. (**A**) Heatmap representing regulons significantly altered in LNCaP ERN1^−^^/^^−^ (yellow) compared to parental LNCaP (mustard). Top 30 statistically significant regulons shown (*p* < 0.001). Regulon levels are represented as single-sample enrichment scores with individual regulons denoted by the name of their corresponding transcription factor (TF). Colour scale represents regulon enrichment z-scores (from lowest in greyscale to highest in red-scale). (**B**) GSVA single-sample gene set enrichment scores in LNCaP ERN1^−^^/−^ cells (yellow) compared to LNCaP parental cells (mustard) shown for gene sets including: TFs with regulons activated in ERN1−/− cells, genes upregulated in the RB1-loss signature from Ertel et al, Limma was used for statistical testing. (**C**) Heatmap of GSVA enrichment scores for 11 selected PCa gene signatures shown across samples from the Labrecque patient cohort (*N* = 98) together with LNCaP parental and ERN1^−^^/−^ cell line samples (*N* = 5). Colour scale represents GSVA enrichment z- scores (from lowest in greyscale to highest in red-scale). mRNA fold change of *ERN1*, *XBP1s*, *RB1*, *BRCA2* in (**D**) LNCaP parental, LNCaP ERN1^−^^/^^−^, ERN1^−^^/−^ Q780*, RB1^−^^/−^, RB1^−^^/−^/TP53^−^^/−^ cells normalised to parental LNCaP, (**E**) LNCaP cells treated with two separate siRNAs against *ERN1* normalised to siCTRL, (**F**) LNCaP cells treated with 5 μM MKC8866 for 8 h normalised to DMSO and LNCaP cells treated with 25 μM IXA4 normalised to DMSO (*n* = 3). (**G**) Western blot of IRE1, RB1, TP53, RBL1, E2F1, CCNE1, CCNA2, CDK4 and Actin in LNCaP parental, LNCaP ERN1^−^^/^^−^, ERN1^−^^/−^ Q780*, RB1^−^^/−^, RB1^−^^/−^/TP53^−^^/−^ cells. (**H**) Heatmap of cell cycling genes significantly altered in LNCaP ERN1^−^^/−^ (yellow) compared to parental LNCaP (purple). GSEA normalised enrichment scores (NES) for significantly enriched MSigDB Hallmark gene sets in: (**I**) LNCaP RB1^−^^/−^ cells compared to LNCaP parental cells, and (**J**) LNCaP RB1^−^^/−^ TP53^−^^/−^ cells compared to LNCaP parental cells grown in FM. Bar length indicates the normalised enrichment score (NES) and bar colour signifies the statistical significance. (**K**) mRNA fold change of *EZH2, POU3F2, SOX2* in LNCaP RB1^−^^/−^, LNCaP RB1^−^^/−^TP53^−^^/−^ cells normalised to parental LNCaP (*n* = 3). (**L**) Clonogenic surviving fraction of LNCaP parental, LNCaP ERN1^−^^/^^−^, ERN1^−^^/−^ Q780*, RB1^−^^/−^, RB1^−^^/−^/TP53^−^^/−^ cells normalised to parental LNCaP (*n* = 14). Statistical tests and *p* values associated with this figure can be found in Dataset EV33. In the box-and-whisker plots, the horizontal lines mark the median, the box limits indicate the 25th and 75th percentiles, and the whiskers extend to the minimum and maximum values. Data information: Data were presented as mean ± SD. ns not significant, *P ≤ 0.05, **P ≤ 0.01, ***P ≤ 0.001, ****P ≤ 0.0001. Source data are available online for this figure.

To further deconvolute the observed transcriptomic cell cycle deregulation (Fig. 1), we mapped TF repertoires (regulons) significantly affected by *ERN1* knockout. Stress response (CREB3L1, down), inflammatory signalling (NFATC3, IRF6, down), cell cycling (E2F3, up), and embryogenesis/stemness/differentiation regulons (SOX4(up), PITX2(up), HOXB13(down)) were significantly altered. RBL1, which has been shown to regulate cell cycle in response to RB1 loss (Flores and Goodrich, 2022), was positively enriched (Fig. 2A; Dataset EV10) while RB1-loss gene signatures (Dataset EV11) segregated ERN1^−^^/−^ from parental cells, in a similar manner to the regulons (Fig. 2B). In a clinical dataset (mCRPC, 98 tumours from 55 men, Data Ref: (Labrecque et al, 2019)), the set of TFs with regulons suppressed by IRE1 KO clustered with AR activity signatures while the TFs with regulons activated by IRE1 KO clustered with RB1-loss and ARV-7 activity signatures (Thangavel et al, 2017; He et al, 2021), distinguishing AR positive from AR-negative PCa. Overall, ERN1^−^^/−^ cells clustered with NEPC and DNPC, as well as some AR-low (ARLPC) mCRPC samples (Fig. 2C), compared to WT cells, which clustered with AR+ve samples.

**Figure EV4:**
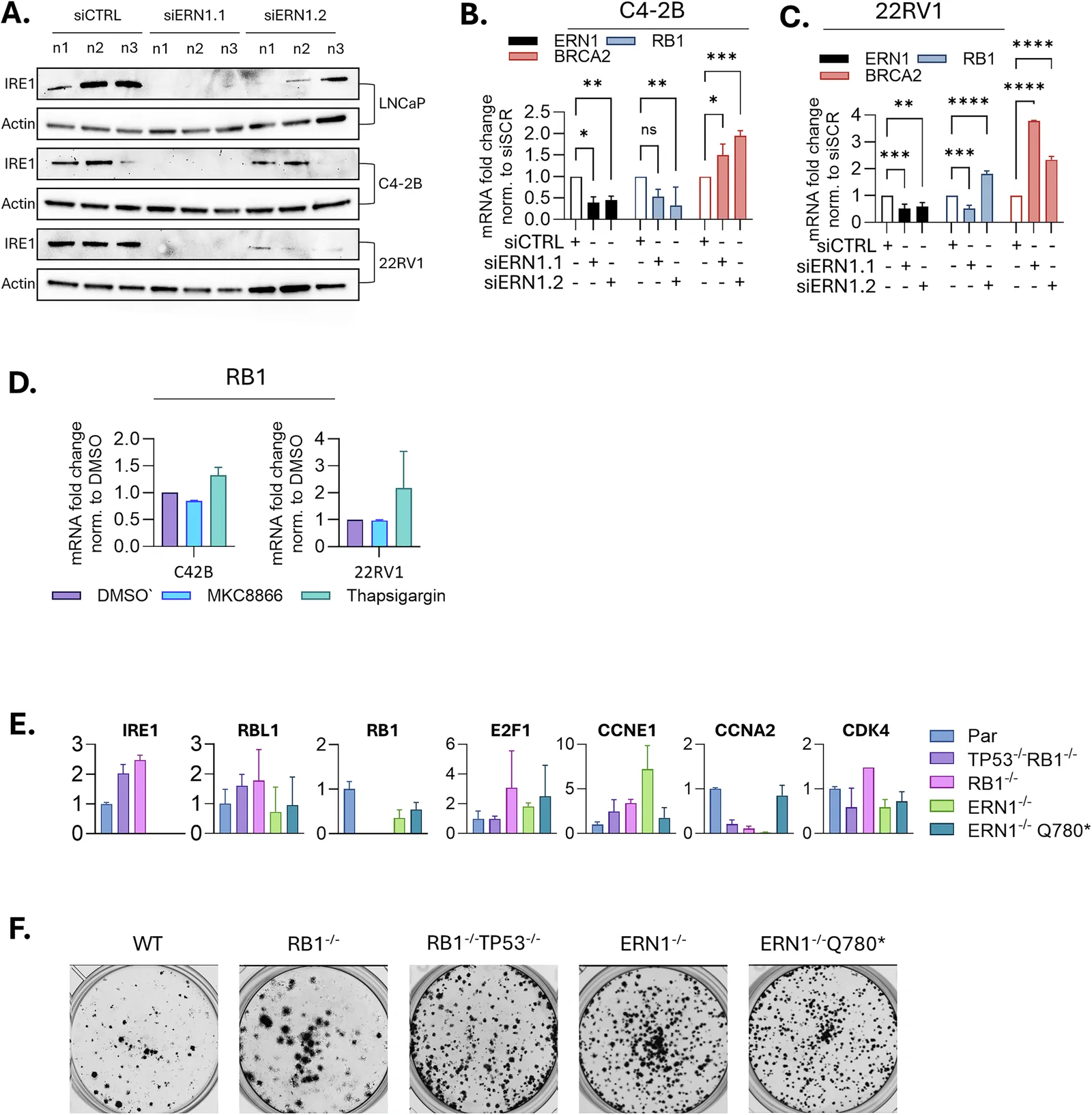
Genetic IRE1 modulation effect on RB1 expression, E2F1 target expression and cell clonogenic capacity. (**A**) Western Blots of IRE1 and Actin in LNCaP, C4-2B and 22RV1 cells treated with siCtrl or two separate siRNAs against ERN1. mRNA fold change of ERN1, RB1, BRCA2 in (**B**) C4-2B parental cells and (**C**) 22RV1 parental cells two siRNAs against ERN1 or XBP1 for 48 h normalised to scramble control (*n* = 3). (**D**) RB1 mRNA fold change in C4-2B and 22RV1 cells treated with MKC8866 (5 μM) and Thapsigargin (100 nM) for 5 h normalised to DMSO (*n* = 2). Statistical analysis was performed using an ordinary two-way ANOVA and Sidak’s multiple comparisons test with a single pooled variance. (**E**) Densitometry of blots presented in main Fig. 2 using Fiji by ImageJ. (**F**) Representative images of clonogenic assays used to measure surviving fractions presented in main Fig. 2. Data information: Data were presented as mean ± SD. ns not significant, *P ≤ 0.05, **P ≤ 0.01, ***P ≤ 0.001, ****P ≤ 0.0001.

Intriguingly, when measuring cell cycle regulation(Miao et al, 2022) gene expression, both IRE1 deficient cell lines showed decreased *RB1* but increased *BRCA2* mRNA expression compared to parental while RB1 deficient cells (RB1^−^^/−^, RB1^−^^/−^/TP53^−^^/−^) showed decreased *ERN1* expression compared to parental (Fig. 2D). *RB1* mRNA decrease was recapitulated by ERN1 silencing in LNCaP, C4-2B and 22RV1 cells (Figs. 2E and EV4A–C). Treatment with the salicylaldehyde reversible IRE1 RNase inhibitor MKC8866 suppressed *RB1* and increased *BRCA2* mRNA expression in LNCaP but not C4-2B or 22RV1 cells. The non-kinase binding, autophosphorylation requiring IRE1 activator IXA4 (Grandjean et al, 2020), and ER stressor thapsigargin had the opposite effect on *RB1* and *BRCA2* expression in all three cell lines (Figs. 2F and EV4D). Cell cycle marker deregulation was not confined to *RB1* and *BRCA2*, with E2F1 targets altered by IRE1 suppression at both protein and mRNA levels (Figs. 2G,H and EV4E,F).

Knocking out RB1, or knocking RB1 out alongside TP53 in LNCaP cells (Ku et al, 2017; Mu et al, 2017) causes resistance to replication stress, induces high proliferative rates and lineage plasticity and increases DNA repair capacity (Nyquist et al, 2020). Hallmarks affected by RB1 loss or RB1 and TP53 loss in LNCaP cells were similar to IRE1 loss (Fig. 2I,J; Datasets EV12 and 13), showing enrichment in E2F, G2M checkpoint and MYC targets. Reprogramming markers *EZH2*, *SOX2* and *POU3F2* were not significantly altered in RB1^−^^/−^ cells while were significantly increased in RB1^−^^/−^/TP53^−^^/−^ cells compared to parental (Fig. 2K). Intriguingly, IRE1-deficient cells showed similar clonogenic capacity to RB1^−^^/−^/TP53^−^^/−^ cells in contrast to RB1^−^^/^^−^ cells compared to parental (Fig. 2L). IRE1 loss in LNCaP cells leads to partial RB1-loss-like characteristics underpinned by some increased lineage reprogramming capacity observations. Thus, IRE1 activity in its role as a stress adaptation mechanism could play a role in the regulatory cascade between RB1 loss and lineage plasticity.

### Low IRE1 activity correlates with lineage reprogramming to a de-differentiated, club cell-like phenotype

**Figure 3:**
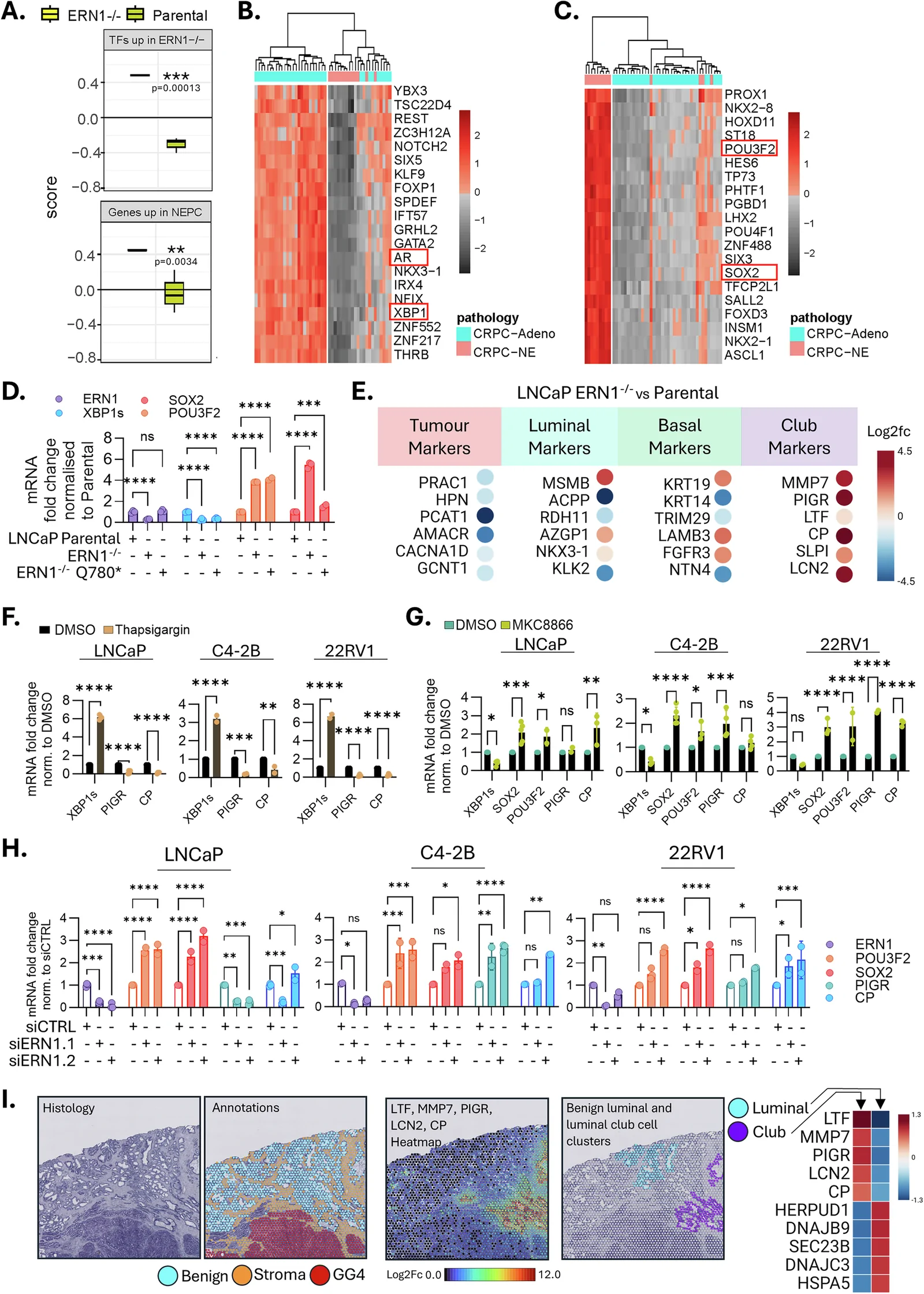
Low IRE1 activity correlates with lineage reprogramming to a club cell-like phenotype. (**A**) GSVA single-sample gene set enrichment scores in LNCaP ERN1^−^^/−^ cells (yellow) compared to LNCaP parental cells (mustard) shown for gene sets including: TFs with regulons activated in ERN1-/- cells, upregulated in the NEPC signature from Beltran et al, Limma was used for statistical testing. (**B**,** C**) Heatmaps representing regulons significantly altered in neuroendocrine (NEPC, pink) castration resistant (CRPC) tumours compared to adenocarcinoma CRPC (light blue). Regulon levels are represented as single-sample enrichment scores with individual regulons denoted by the name of their corresponding transcription factor (TF). Significantly activated and suppressed regulons are shown, with AR, XBP1 (AR, IRE1 activity), SOX2 and POU3F2 (reprogramming) in red boxes. Colour scale represents regulon enrichment z-scores from lowest in greyscale to highest in red-scale. (**D**) mRNA fold change of *XBP1s, EZH2, POU3F2, SOX2*, in LNCaP parental, LNCaP ERN1−/− and ERN1−/− Q780* cells normalised to parental LNCaP (*n* = 3). (**E**) Bulk RNAseq log2 fold change expression levels in ERN1^−^^/−^ cells compared to parental, of genes widely reported to be associated with tumour, luminal epithelial, basal epithelial and club epithelial cells. Downregulated genes in blue, upregulated genes in red. mRNA fold change of *XBP1s*, *PIGR*, *CP*, *SOX2* and *POU3F2* in LNCaP, C4-2B and 22RV1 cells treated with (**F**) 100 nM Thapsigargin, normalised to DMSO, (**G**) MKC8866 normalised to DMSO and (**H**) two separate siRNAs against *ERN1* normalised to siCTRL (*n* = 3). (**I**) Histological annotations of a 6.5 mm^2^ tissue slide where benign (blue), stroma (green) and grade group 4 (GG4) cribiform tumour is represented (red). Loupe browser-generated distribution maps of club- associated genes (*LTF, MMP7, PIGR, LCN2, CP*) across these histological designations, showing specific localisation to peri-tumoral tissue. Heatmap log fold change of IRE1 induced and club-cell genes comparing ~100 spots of benign (cyan) and club-benign (purple) cells. In the box-and-whisker plots, the horizontal lines mark the median, the box limits indicate the 25th and 75th percentiles, and the whiskers extend to the minimum and maximum values. Statistical tests and *p* values associated with this figure can be found in Dataset EV33. Data information: Data were presented as mean ± SD. ns not significant, *P ≤ 0.05, **P ≤ 0.01, ***P ≤ 0.001, ****P ≤ 0.0001. Source data are available online for this figure.

Lineage plasticity and associated lineage reprogramming (Venkadakrishnan et al, 2024; Mu et al, 2017) genes such as *SOX2* and *POU3F2* are strongly associated with NEPC phenotypes. NEPC signatures segregated ERN1^−^^/−^ from parental LNCaP cells, in a similar manner to TF regulons induced by IRE1 loss (Fig. 3A; Dataset EV11). Further regulon analyses of 48 CRPC samples split into CRPC-Adenocarcinoma (*n* = 35, AR positive) versus CRPC-neuroendocrine prostate cancer (NEPC, *n* = 13, AR negative, Data Ref: (Conteduca et al, 2019)) showed that XBP1 regulons were less active in NEPC samples, while reprogramming markers pertaining to stem-like lineages such as SOX2 and POU3F2 were increasingly active (Fig. 3B,C).

**Figure EV5:**
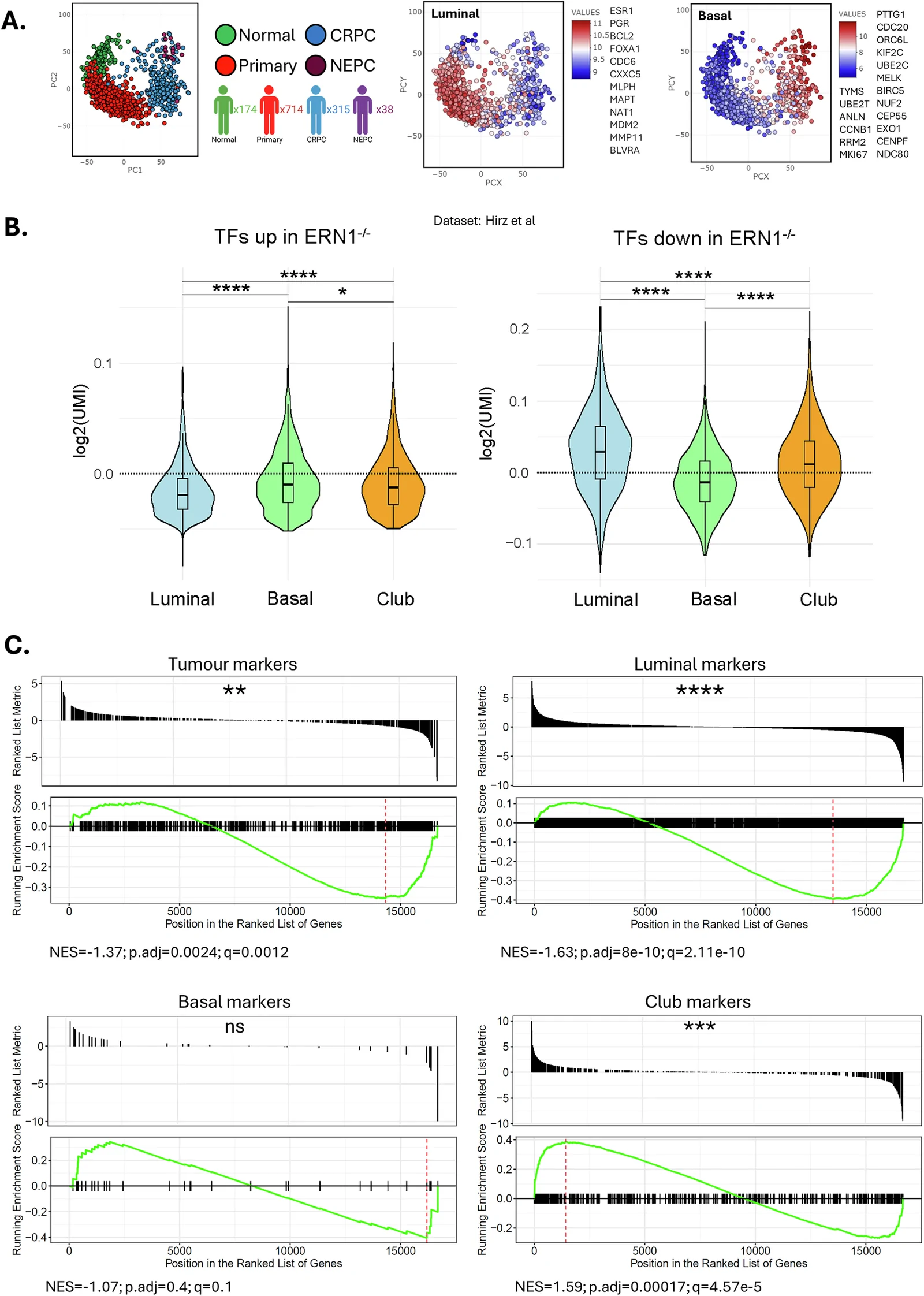
IRE1 deficient cells are enriched for club but deficient for tumour or luminal transcriptomic repertoires. (**A**) Basal/epithelial transcriptomic signatures in a comprehensive prostate cancer transcriptome atlas of integrated RNA-seq datasets from over 1000 clinical tissue samples ranging from benign (green) to NEPC (purple). Heatmaps show the distribution of signal (low/blue; high/red) of these signatures across the dataset. (**B**) Violin plots showing the enrichment of transcription factors significantly deregulated in the absence of IRE1 activity in basal cells compared to luminal epithelial cells in a single cell dataset. (**C**) Gene set enrichment plots of gene sets representing tumour, luminal epithelial, basal epithelial and club epithelial cells in the LNCaP ERN1−/− cells compared to parental.

Intriguingly, ^25,27^*SOX2* and *POU3F2* mRNA expression was significantly upregulated in IRE1-deficient cells (Fig. 3D) compared to parental. Therefore, adaptation to IRE1 activity loss may have induced a lineage shift in AR+ve PCa cells. To further test this association, we correlated luminal and basal gene signatures with disease progression in the Prostate Cancer Atlas (Fig. EV5A) and observed that luminal genes showed similar changes to IRE1-induced transcripts, decreasing with disease severity (Fig. 1A).

Comparing luminal, basal and club cells within a PCa SC dataset (Data Ref: (Hirz et al, 2023)), we observed that TFs with regulons activated upon IRE1-loss were significantly upregulated in basal and club cells compared to luminal epithelial cells (Fig. EV5B). We then documented the enrichment of genes upregulated in tumoral, luminal, basal and club cells (Kiviaho et al, 2024) (Dataset EV14) in the ERN1^−^^/−^ transcriptome. ERN1^−^^/−^ cells were positively enriched for club (Huang et al, 2023; Song et al, 2022; Joseph et al, 2022) while negatively enriched for tumoral and luminal cell markers, showing decreases of *AMACR*, ACPP and NTN4 but increases in *MMP7, PIGR, MSMB* and *KRT19* (Figs. 3E and EV4C). LNCaP ERN1^−^^/−^ Q780* cells also showed significantly increased expression of club-cell lineage markers. Stably overexpressing the Q780* mutant in C4-2B cells caused an upregulation of *SOX2*, *PIGR* and *CP* (Appendix Fig. S2A,B). Treatment with the ER stressor thapsigargin significantly suppressed *PIGR* and *CP* mRNA in LNCaP, C4-2B and 22RV1 cells (Fig. 3F) while MKC8866-induced IRE1 inhibition had the opposite effect, causing upregulation of both reprogramming and club markers (Fig. 3G) in all three cell lines in varying degrees. Interestingly, ERN1 silencing did not produce as consistent effects, showing decreases in PIGR and CP in LNCaP cells for at least one of the siRNAs, possibly due to unequal ERN1 suppression (Figs. 3H and EV4A) or indirect effects on other regulators of club-associated genes. To determine correlation trends between IRE1 activity and club lineages in patient tissue, we constructed ST (Data Ref: (Erickson et al, 2022)) data expression maps encompassing benign, stromal and localised grade group (GG)4 histological annotations. We thus demonstrated that club-cell gene enrichment overlapped with reduced IRE1-induced transcripts when comparing non-tumoral, epithelial regions positive for club markers to negative ones (Fig. 3I).

Typical club cell phenotype is more prone to reprogramming, a hallmark of treatment-resistant disease. In parallel, low IRE1 activity correlates with ADT-resistant clinical presentations. ADT can suppress IRE1 activity and induce club phenotypes. Finally, IRE1-deficient cells express high *RBL1* and low *RB1* mRNA levels while retaining characteristics of lineage reprogramming.

### Enzalutamide treatment inhibits the UPR and IRE1 activity induction, partially sensitises resistant cells to ADT

**Figure 4:**
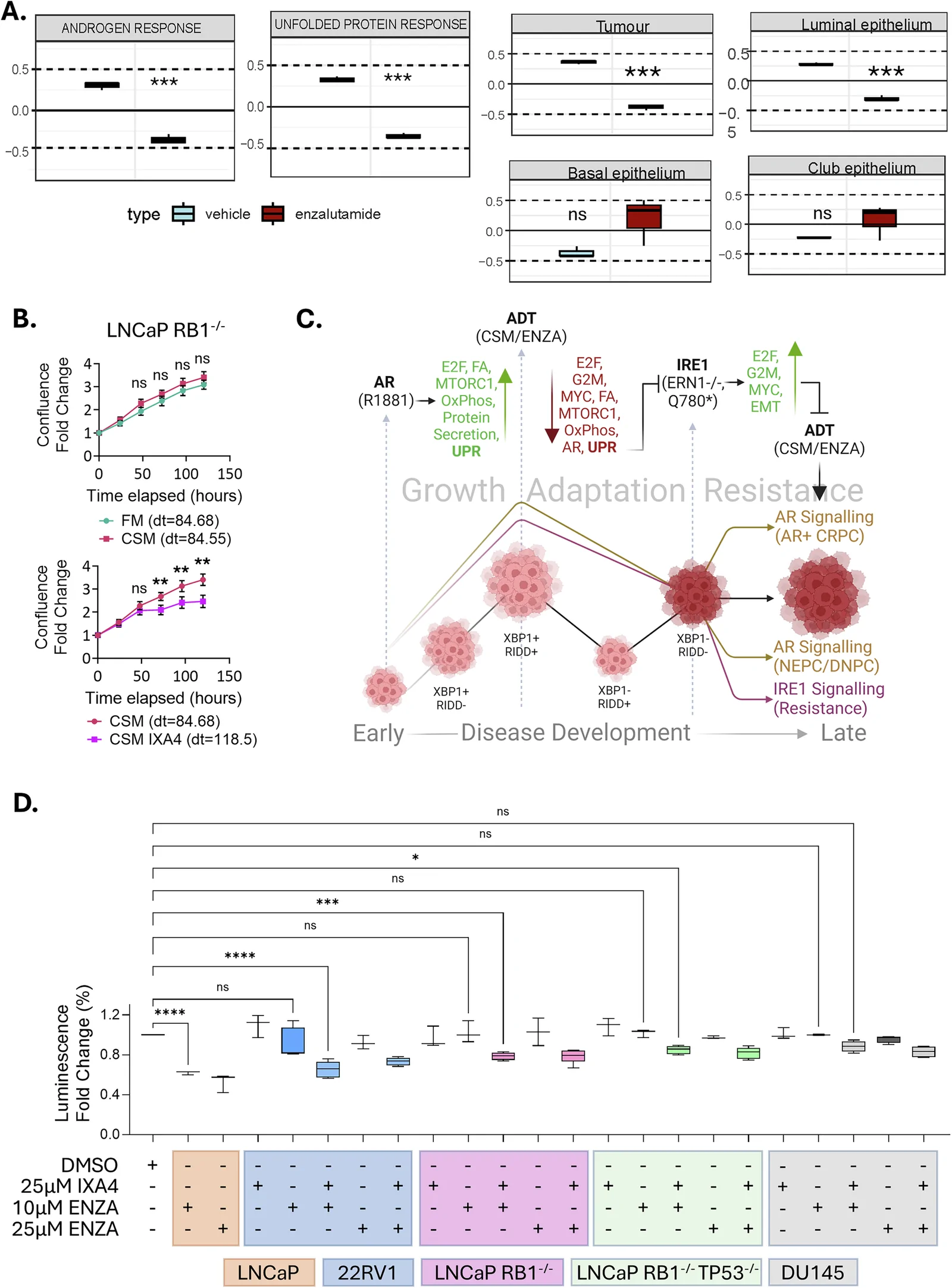
Enzalutamide treatment induces a UPR downregulation, which may act as a negative feedback loop that leads to treatment resistance to ADT in PCa. (**A**) Box plots of GSVA enrichment scores for Hallmark Androgen Response, Hallmark Unfolded Protein Response, and gene lists specific for tumour, luminal, club and basal epithelial cells, benchmarked in PDEs treated with 10 μM Enzalutamide (red, right) or vehicle control (blue, left) (*n* = 6). Statistical testing was performed using limma (lmFit function). (**B**) Growth curves of LNCaP RB1^−^^/−^ cells grown over 120 h in FM (green), CSM (red) or in CSM in the presence of subtoxic (25 μM) doses of IXA4 (purple) (*n* = 3). Confluence fold change was normalised to Incucyte readings taken at the 0-hour time point of each condition. *T*-tests were used for individual time point comparisons between FM and CSM; CSM and CSM plus IXA4. Exponential (Malthusian) growth curve equations were used to estimate the doubling time of each condition. (**C**) Schematic summarising observations of Figs. 1–4. During AR-driven tumour growth, represented by the R1881 dataset, multiple key mechanisms are upregulated, including the UPR. In the eventuality of ADT represented by the CSM and ENZA datasets, these processes are reversed/ downregulated. However, removing IRE1 activity in isolation leads to an increase in processes that directly contradict the effects of ADT, leading to partial treatment resistance. (**D**) Cell titre glo assay, fluorescence fold change of treatment-resistant LNCaP RB1^−^^/−^, LNCaP RB1^−^^/−^TP53^−^^/−^, 22RV1 and DU145 cells treated with 10 μM and 25 μM ENZA in the presence or absence of 25 μM IXA4 (*n* = 3). Statistical significance of 10 μM ENZA treatment is shown. In the box-and-whisker plots, the horizontal lines mark the median, the box limits indicate the 25th and 75th percentiles, and the whiskers extend to the minimum and maximum values. Complete statistical tests and *p* values associated with this figure can be found in Dataset EV33. Data information: Data were presented as mean ± SD. ns not significant, *P ≤ 0.05, **P ≤ 0.01, ***P ≤ 0.001, ****P ≤ 0.0001. Source data are available online for this figure.

We showed that adaptation to IRE1 activity loss may push AR-responsive cells towards an ADT-resistant phenotype. We then hypothesised that this event marks the starting point of IRE1 activity saturation and the shift from an active phenotype to a passive, adapted phenotype. ADT could catalyse this key event in the shift from early-stage to late-stage disease progression. Having reported that ENZA treatment reduces UPR/IRE1 activity in cell lines, we tested its effects on more complex, heterogenous PDEs. ENZA treatment not only suppressed AR and UPR hallmarks (Fig. 4A) while increasing inflammatory responses and EMT (Appendix Fig. S3A; Datasets EV15 and EV16), but also significantly decreased luminal and tumoral markers while inducing enrichment trends of club and basal repertoires (Fig. 4A; Dataset EV14). We have so far correlated IRE1 activity loss with resistance to ADT. We proceeded to test the effect of pharmacological IRE1 activity induction on the ability of ADT-resistant cells to grow in CSM. CSM did not affect the growth of either RB1^−^^/−^ or RB1^−^^/−^/TP53^−^^/−^ LNCaP cells. However, addition of subtoxic doses of the IRE1 activator IXA4, slowed their growth in CSM (Fig. 4B; Appendix Fig. S3B,C).

Thus, it seems that IRE1 activity suppression by ADT may play a role in acquired treatment resistance through lineage reprogramming to more cell plastic phenotypes (Fig. 4C). ADT-resistant models presented low IRE1 levels and activity as evidenced by similar transcriptomic responses to ENZA treatments between ERN1^−^^/−^ and RB1^−^^/−^TP53^−^^/−^ cells (Appendix Fig. S3A–D; Datasets EV17 and 18). Hence, we tested whether IRE1 activation could sensitise ADT-resistant cells to ENZA. Whereas the IRE1 activator IXA4 had no effect on viability, it modestly sensitised AR+ve CRPC cells (LNCaP RB1^−^^/−^, LNCaP RB1^−^^/−^TP53^−^^/−^, 22RV1) cells to physiologically relevant doses of ENZA over a short 12-h treatment. It did not affect the AR-ve DU145 cells (Fig. 4D; Appendix Fig. S3E–H). Consequently, ENZA treatment suppresses IRE1 activity in cell lines and PDEs; treatment-resistant PCa pre-clinical models have low IRE1 activity, recapitulating clinical observations; and a short-term increase in IRE1 activity enhances responses to AR antagonism.

### Integrative analysis of transcriptomic datasets gives rise to a novel IRE1 activity gene set (IRE1sig1.0) that associates with disease progression

**Figure 5:**
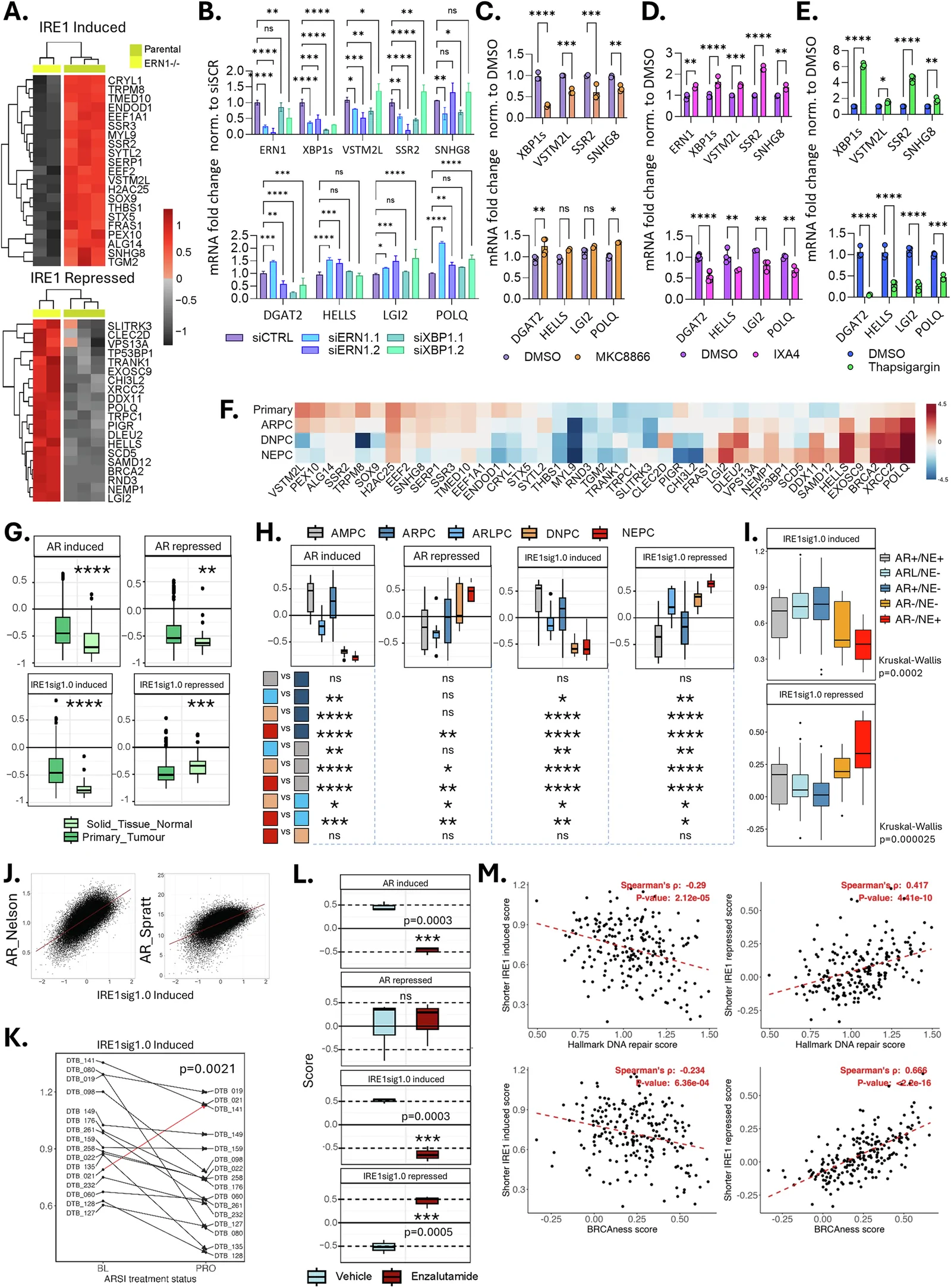
Integrative analysis of transcriptomic datasets gives rise to a novel IRE1 activity gene set (IRE1sig1.0) that associates with disease progression. (**A**) Gene expression heatmaps of genes in the IRE1 activity signature defined as “IRE1 induced” (top) when downregulated in LNCaP ERN1^−^^/−^ cells (yellow) compared to parental (mustard) and as “IRE1 repressed” (bottom) when upregulated in LNCaP ERN1^-/-^ cells compared to parental. mRNA fold change of *ERN1, XBP1s, DGAT2, VSTM2L, SSR2, SNHG8, HELLS, LGI2* in LNCaP parental cells treated with (**B**) two separate siRNAs against ERN1 or XBP1 for 48 h normalised to scramble control, (**C**) 5 μM MKC8866 for 5 h normalised to DMSO, (**D**) 25 μM IXA4 for 5 h normalised to DMSO and (**E**) 100 nM Thapsigargin for 5 h normalised to DMSO (*n* = 4). (**F**) Heatmap of the IRE1 activity gene set expression fold change in primary, AR-positive CRPC, NEPC and DNPC patient cohorts compared to normal tissue (Prostate Cancer Atlas). Fold changes calculated using “Normal” as control in the DESeq2 Prostate Cancer Atlas function. Downregulated genes in blue, upregulated in red. Box plots of GSVA enrichment scores for AR activity and IRE1sig1.0 gene sets, benchmarked in (**G**) localised disease (TCGA-PRAD) and (**H**) mCRPC (Labrecque) datasets. Patient populations represented include AR positive (dark blue), AR low (light blue), amphicrine (grey), NEPC (red), double negative (DNPC, orange), normal tissue (light green) and primary tumour (dark green). (**I**) Box and whiskers plot of GSVA single-sample gene set enrichment scores for induced (top) and repressed (bottom) part of IRE1sig1.0 gene sets across mCRPC molecular subtypes in WCDT cohort (*N* = 210). Boxplot showed median with hinges at 25th and 75th percentiles and whiskers at the largest/smallest value within 1.5 x interquartile range. Subtypes can be read as follows: AR + /NE + , grey; ARL/NE-, light blue; AR + /NE-, dark blue; AR-/NE-, orange; AR-/NE + , red. (**J**) Correlation analysis of IRE1sig1.0 induced to AR activity signatures showing strong correlation between the scores (Pearson’s correlation coefficient of 0.6 between IRE_18 induced and AR_Spratt and 0.68 with AR_Nelson. (**K**) Pre versus post ARSI treatment IRE1sig1.0 GSVA single-sample enrichment induced score comparison before (BL, Baseline, left, *N* = 16) and after (PRO, Progression, right, *N* = 16) treatment. Red arrows indicated an increase, whereas black arrows showed a decrease after treatment. *P* value was reported from Wilcoxon signed-rank test. (**L**) Testing of AR activity and IRE1sig1.0 gene sets were benchmarked in explants treated with Enzalutamide (10 μM, red, right) or vehicle control (blue, left) (*n* = 6). Statistical testing was performed using limma (lmFit function). (**M**) GSVA single-sample enrichment scores of IRE1sig1.0 induced and repressed scores correlation with those from Hallmark DNA repair and BRCAness (*n* = 210). In the box-and-whisker plots, the horizontal lines mark the median, the box limits indicate the 25th and 75th percentiles, and the whiskers extend to the minimum and maximum values. Statistical tests and *p* values associated with this figure can be found in Dataset EV33. Data information: Data were presented as mean ± SD. ns not significant, *P ≤ 0.05, **P ≤ 0.01, ***P ≤ 0.001, ****P ≤ 0.0001. Source data are available online for this figure.

**Figure EV6:**
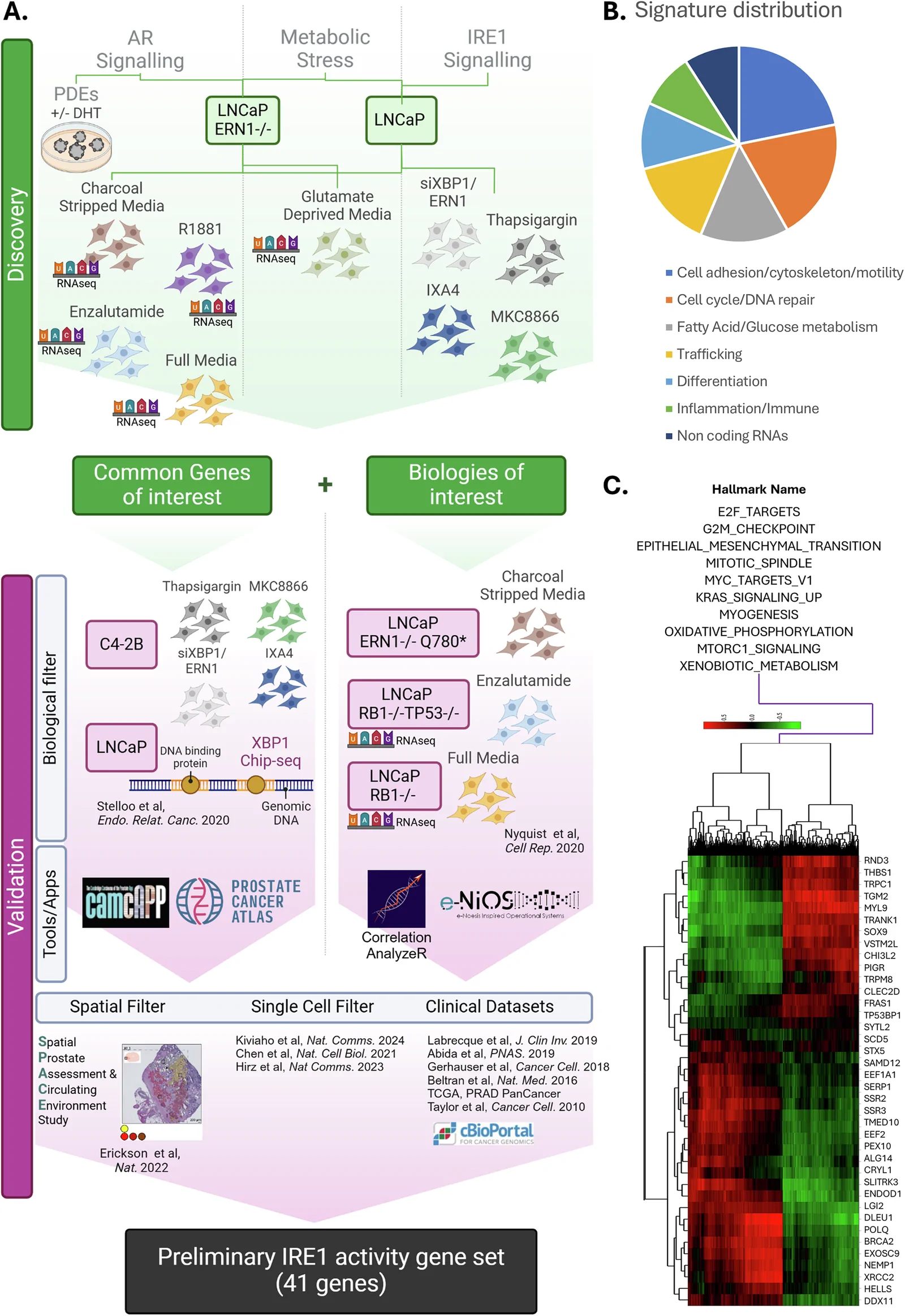
Integration of multiple transcriptomic datasets produces IRE1 activity signature. (**A**) Schematic showing the –omics datasets involved in generating the IRE1 activity gene set. Green lines signify internally generated datasets forming the Discovery component. External and internal datasets used to filter the signature forming the Validation component are highlighted in pink. (**B**) Pie chart indicating the biologies represented within the new IRE1 gene set. These biologies are the result of a GSEA analysis of the final gene list. (**C**) Heatmap correlating expression of the 41 genes in the IRE1 activity gene set with 500 genes significantly differentially expressed in prostate cancer. The hallmarks these 500 genes represent are displayed above the heatmap. This heatmap was generated using the Correlation AnalyzeR shiny app with the input being the list of 41 IRE1 activity genes, in the topology analysis suite and selecting the tissue type as prostate and sample type as cancer.

We identified a group of IRE1-responsive genes, labelling them “IRE1 activity induced” (significantly downregulated in ERN1^−^^/−^) or “IRE1 activity repressed” (significantly upregulated in ERN1^−^^/−^) (Dataset EV19) by integrating results from multiple RNAseq datasets (41 genes, Figs. 5A and EV6A). This gene set represents key PCa progression biological functions comprising cell adhesion, motility, cell cycle, DNA repair, and protein trafficking, while correlating with changes in cell cycle, EMT and metabolism PCa-associated genes (Fig. EV6B,C; Datasets EV20 and 21).

**Figure EV7:**
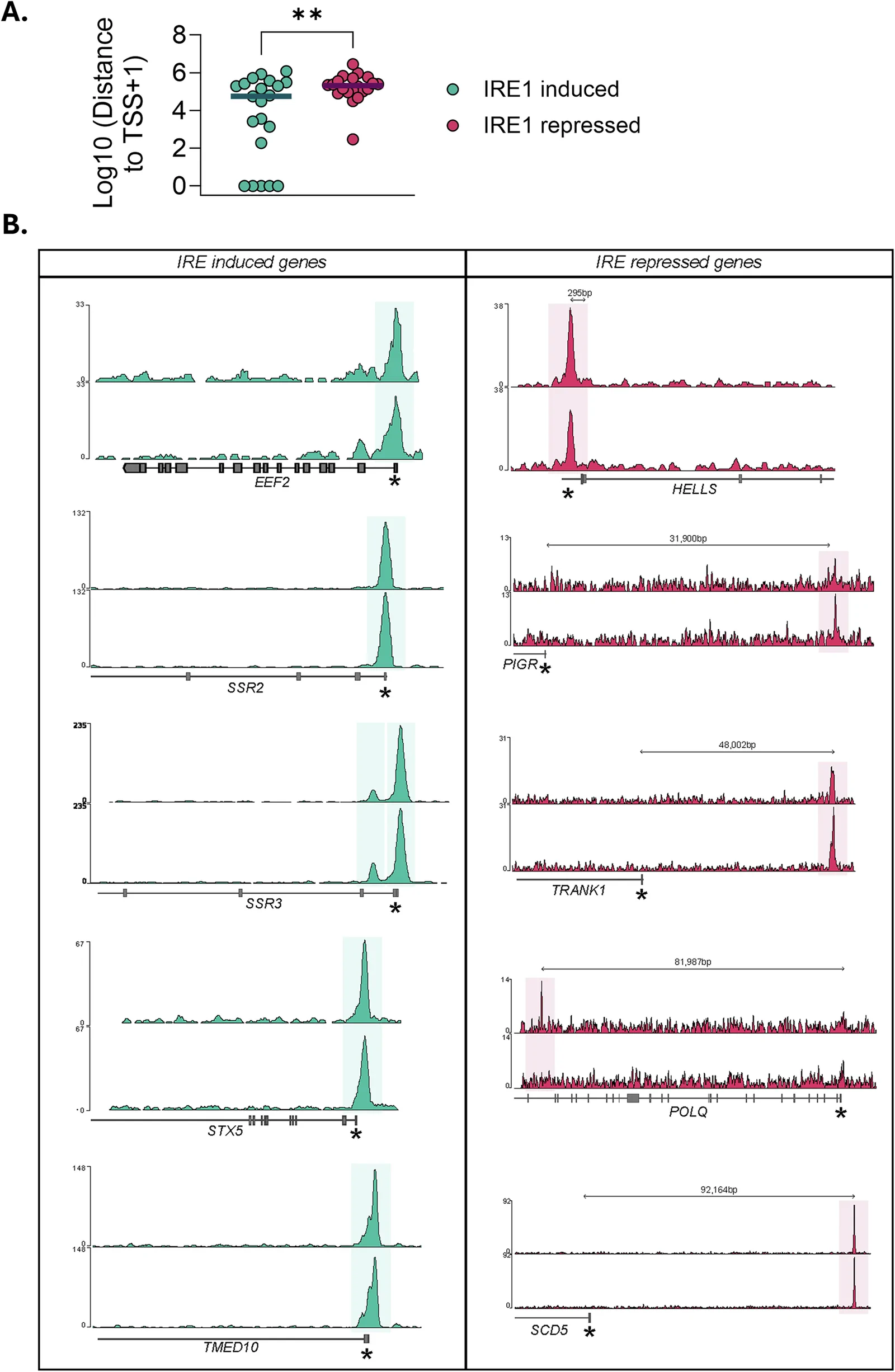
Association of IRE1 activity signature genes with XBP1 transcription factor binding sites. (**A**) Log10 of the distance between the transcription start site (TSS) of each of the IRE1_41 genes and the nearest significant XBP1 peak. 43% of “IRE1 induced” genes have an XBP1 peak in their promoter. (**B**) Genomic tracks for the five closest IRE-induced and five closest IRE-repressed genes to an XBP1 peak. The distance to the closest IRE-regulated gene is indicated with an arrow (except when the gene is at the TSS, where distance = 0). A star marks the TSS of each IRE-regulated gene. Transparent blocks highlight XBP1-called peaks. Data information: Data were presented as mean ± SD. ns not significant, *P ≤ 0.05, **P ≤ 0.01, ***P ≤ 0.001, ****P ≤ 0.0001. Source data are available online for this figure.

**Figure EV8:**
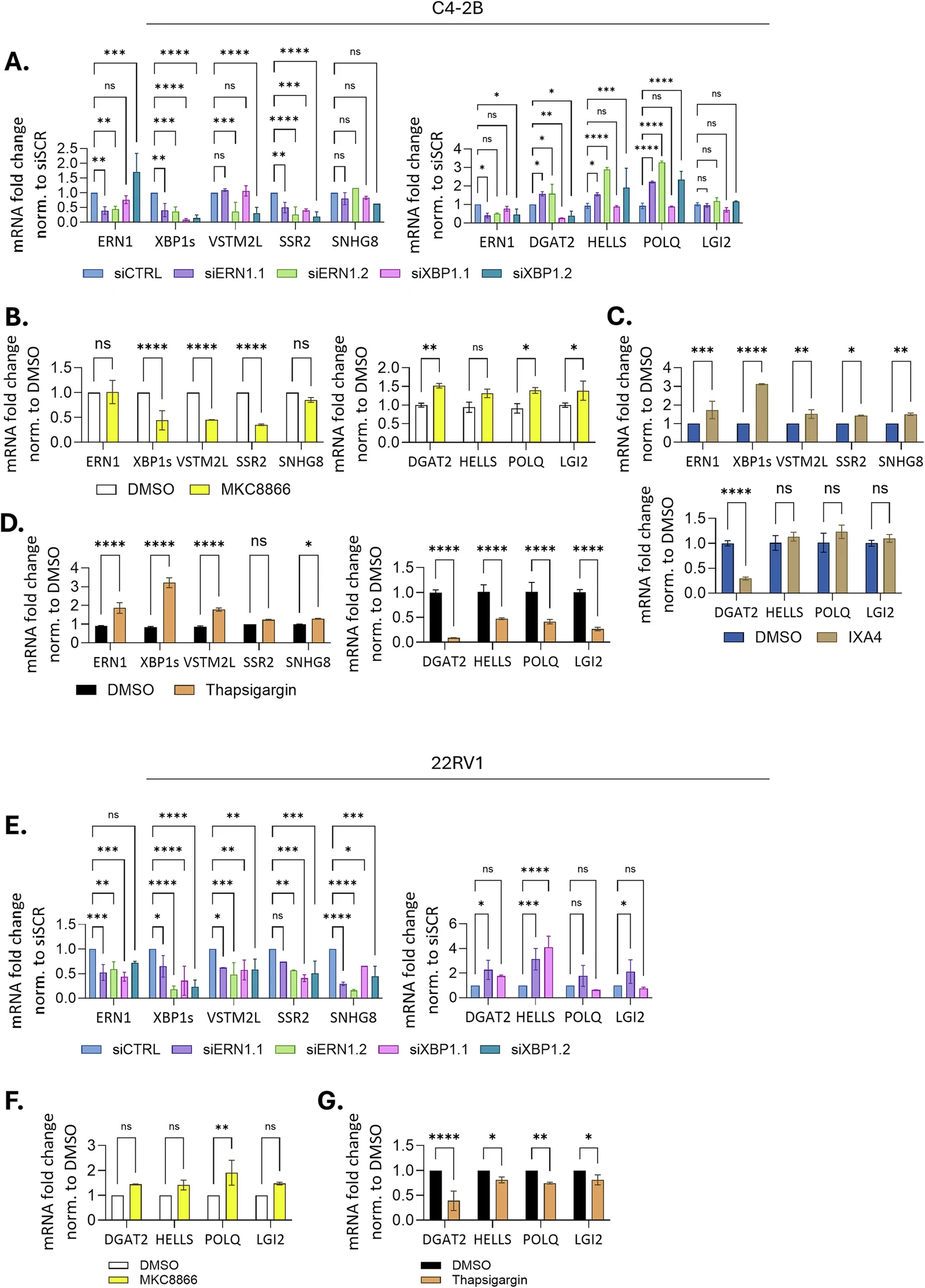
Changes in IRE1 activity signature mRNA upon pharmacological or genetic IRE1 modulation. mRNA fold change of ERN1, XBP1s, DGAT2, VSTM2L, SSR2, SNHG8, HELLS, LGI2, POLQ in C4-2B parental cells treated with (**A**) two siRNAs against ERN1 or XBP1 for 48 h normalised to scramble control, (**B**) 5 μM MKC8866 for 5 h normalised to DMSO, (**C**) 25 μM IXA4 for 5 h normalised to DMSO and (**D**) 100 nM Thapsigargin for 5 h normalised to DMSO (*n* = 3). mRNA fold change of ERN1, XBP1s, DGAT2, VSTM2L, SSR2, SNHG8, HELLS, LGI2, POLQ in 22RV1 parental cells treated with (**E**) two siRNAs against ERN1 or XBP1 for 48 h normalised to scramble control. mRNA fold change of DGAT2, HELLS, LGI2, POLQ in 22RV1 parental cells treated with (**F**) 5 μM MKC8866 for 5 h normalised to DMSO, (**G**) 100 nM Thapsigargin for 5 h normalised to DMSO (*n* = 3). Statistical analysis was performed using an ordinary two-way ANOVA and Sidak’s multiple comparisons test with a single pooled variance. Data information: Data were presented as mean ± SD. ns not significant, *P ≤ 0.05, **P ≤ 0.01, ***P ≤ 0.001, ****P ≤ 0.0001. Source data are available online for this figure.

We then investigated whether this multi-transcriptomic data integration approach yielded direct IRE1 activity targets. XBP1 ChIP-seq analysis of LNCaP cells treated with R1881 for 24 h showed that multiple “IRE1 induced” genes displayed XBP1 peaks directly in their promoter (Fig. EV7A,B; Dataset EV22). RNA silencing of *ERN1* or *XBP1* downregulated “IRE1 induced” and upregulated “IRE1 repressed” genes in LNCaP, C4-2B and 22RV1 cells (Figs. 5B and EV8A,E) with some variability observed between the two siRNAs. MKC8866 treatment decreased “IRE1 induced” but did not affect “IRE1repressed” genes (Figs. 5C and EV8B,F). IXA4 treatment suppressed “IRE1 repressed” while induced “IRE1 induced” expression (Figs. 5D and EV8C), similarly to thapsigargin (Figs. 5E and EV8D,G) treatment. To test whether the changes shown in the expression of IRE1 repressed genes were due to increased gene transcription or mRNA post-transcriptional regulation and as such bona fide RIDD targets, we treated LNCaP, C4-2B and 22RV1 cells with thapsigargin or MKC8866 in the presence or absence of the transcription inhibitor actinomycin D using *XBP1s* and *DGAT2* as controls. Expectedly, we observed inter-cell line variability with actinomycin D significantly lowering global mRNA expression. However, MKC8866 increased, while thapsigargin decreased mRNA expression of certain genes independently of transcription. *POLQ*, *BRCA2*, *TRPC1* and *HELLS* presented the more consistent expression patterns (Appendix Figs. S4A,B and S5A).

**Figure EV9:**
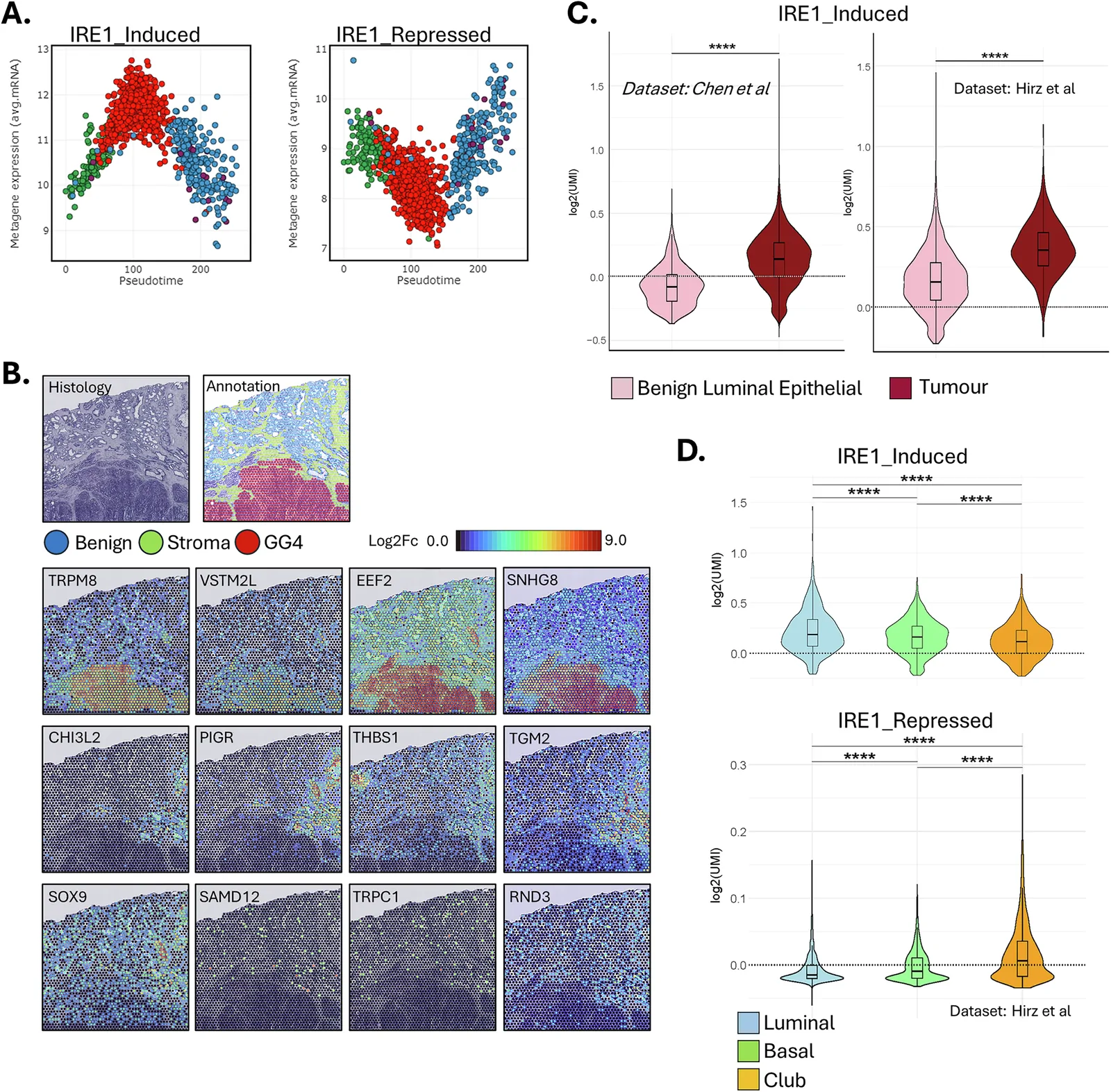
IRE1 activity signature signal distribution across cell types in single cell and spatial datasets. (**A**) Unbiased trajectory analysis by inferred pseudotime progression. Plot representing the correlation between mRNAs of IRE1_18 induced and repressed gene set and pseudotime inferred along the trajectory showing the change of these mRNAs across different clinical designations, including normal tissue (green), primary tumour (red), AR-positive CRPC (blue) and NEPC (purple). (**B**) Example distribution maps of genes within the IRE1_18 gene set representing GG4 tumour, generalised benign or specific benign histological annotations. This histology is referenced in Figs. 3I and 6B. (**C**) Violin plots showing the enrichment of the IRE1-induced part of the IRE1_18 signature in tumour cells compared to normal epithelial cells in two separate single-cell datasets. (**D**) Violin plots showing the enrichment of IRE1_18 induced or IRE1_18 repressed genes, in basal cells and club cells, compared to luminal epithelial cells in a single cell dataset.

Assessing the new IRE1 activity gene set in the Prostate Cancer Atlas, we documented the pseudotime disease progression trajectory attributed to the “IRE1 induced” and “IRE1 repressed” components (Fig. EV9A), which showed complementary pseudotime patterns. Individually, some genes distinguished AR+ from AR- disease (e.g. VSTM2L and TRPM8), some steadily increased (e.g. *BRCA2* and *POLQ*) or decreased (e.g. *CRYL1* and *STX5*) with disease severity and some (*MYL9, THBS1, TRANK1, TGM2, RND3* and *CHI3L2)* were consistently downregulated compared to normal tissue (Fig. 5F).

The IRE1 activity gene set was derived from significant differential expression upon IRE1 perturbation in pre-clinical cancer models. However, some of these genes under IRE1 control could be enriched in other cell types. ST (Erickson et al, 2022) data expression maps encompassing benign, stromal and localised grade group (GG)4 histological annotations, showed that signal was concentrated exclusively in the benign/stromal region; either ubiquitously (*RND3* and* MYL9*) or in very specific benign sub-compartments (*TGM2*). Some genes were tumour-region specific, with the majority of “IRE1 induced” genes (e.g. *SNHG8*) enriched in GG4 (Fig. EV9B). In addition, IRE1-induced genes were significantly upregulated in tumoral cells in two separate SC datasets of primary tumour and matched benign samples (Data Ref: (Chen et al, 2021; Hirz et al, 2023)) (Fig. EV9C). Recapitulating multicellularity, the IRE1 activity signature could distinguish between SC luminal, basal and club cells (Fig. EV9D).

Using these ST and SC sequencing filters, we selected refined IRE1 activity gene sets. Applying further redundancy filters (AR- dependent expression patterns, specific tissue spatial assignment (Appendix Fig. S6A)) produces shorter gene sets. We applied these to segregating mCRPC (Labrecque) or localised (PRAD TCGA) cohorts (Appendix Fig. S6B,C). The final, optimised set of 18 genes (labelled IRE1sig1.0) still represented diverse biological functions and cell types (Appendix Fig. S6D; Dataset EV23). Both “IRE1 induced” and “IRE1 repressed” parts of IRE1sig1.0 differentiated AR-positive and AR-negative tumours from normal tissue (Appendix Fig. S6E; Datasets EV24 and 25).

**Figure EV10:**
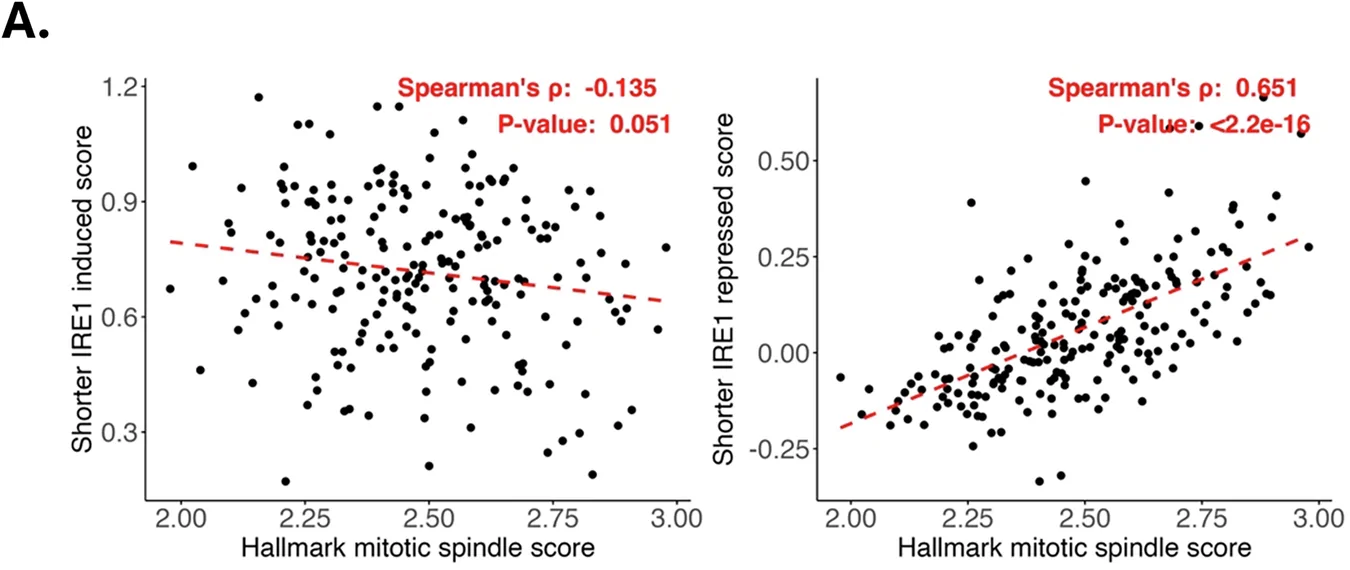
IRE1 activity signature induced and repressed score correlation with hallmark mitotic spindle. (**A**) IRE1sig1.0 induced score and IRE1sig1.0 repressed score correlation with Hallmark mitotic spindle score (*N* = 210). Source data are available online for this figure.

We next assessed how IRE1sig1.0 clustered metastatic (Labrecque) and localised (TCGA-PRAD) patient cohorts compared to AR signatures. In the TCGA-PRAD, the IRE1sig1.0_induced was significantly decreased in primary disease compared to benign tissue. The exact opposite was observed in the IRE1sig1.0_repressed (Fig. 5G). IRE1sig1.0_induced was significantly reduced in NEPC/DNPC mCRPC samples compared to AR+ve mCRPC samples, showing similarity to a 10-gene AR-induced signature (Bluemn et al, 2017). Within the AR+ve mCRPC groups, IRE1sig1.0_repressed most negatively enriched in the amphicrine group whilst most positively enriched in the AR-low group (Fig. 5H). We further validated these observations in the West Coast Dream Team dataset, where both parts of IRE1sig1.0 segregated AR-positive from AR-negative tumours (Fig. 5I; Dataset EV26). IRE1sig1.0_induced strongly correlates with AR activity signatures (Fig. 5J) in the Genomic Resource for Intelligent Discovery research dataset (GRID, Veracyte Inc.) while predicting Androgen Receptor Signalling Inhibition (ARSI) treatment status in both clinical (WCDT, Fig. 5K) and pre-clinical samples (ENZA-treated PDEs, Fig. 5L). IRE1sig1.0_repressed component conversely, was significantly upregulated, in ENZA-treated PDEs, while the AR repressed signature showed no change compared to vehicle control (Fig. 5L). IRE1sig1.0_repressed strongly correlated with DNA repair, BRCAness and spindle formation (Figs. 5M and EV10A; Dataset EV27).

As such, IRE1sig1.0, comprising some direct and some indirect IRE1 targets, shows that IRE1 activity is a proxy of multiple cell types and biological pathways within the PCa environment. IRE1sig1.0 could segregate benign from tumoral tissue, AR-positive from AR-negative disease, and indicate ARSI response in multiple datasets.

### IRE1sig1.0 may act as a proxy of multiple clinical attributes and is prognostic of prostate cancer progression

Recently, we described how gene expression profiling of virtual tumour and benign biopsies (punch biopsies of RP resections to mimic core needle biopsy) reveals differences between grade groups and tumour clones not reflected in bulk analyses. Therefore, bulk analyses of whole biopsies or tumour-only areas, as used in clinical practice, may provide inaccurate assessments (Figiel et al, 2023) as tissue type, tumour grade and clonal composition all influence gene expression.

**Figure EV11:**
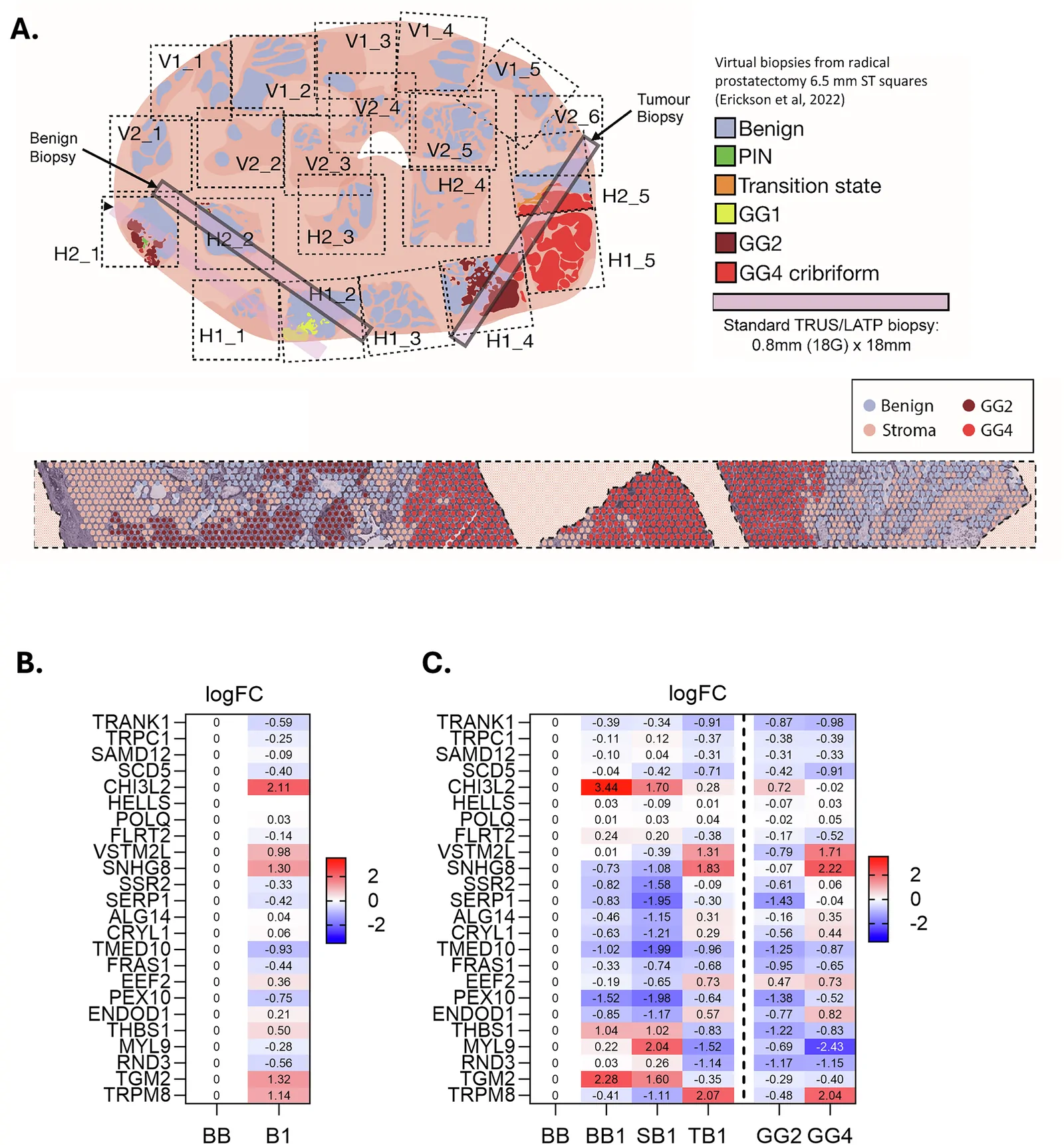
IRE1 activity signature signal distribution in virtual biopsies derived from spatial transcriptomic data. (**A**) Schematic representing the placement of a virtual biopsy containing GG2 and GG4 tumour or benign only tissue (black rectangles). (**B**) Pseudobulk analysis of tumour-bearing biopsy (B1) versus benign biopsy (BB). (**C**) Heatmaps showing changes in each sub-compartment within the virtual biopsy compared to the benign biopsy. These comparisons include benign tissue within the biopsy (BB1), stroma (SB1), tumour (TB1), as well as individual grade groups GG2 or GG4). Blue colour signifies downregulation, red colour signifies upregulation. The log2fc values for each gene are represented on the heatmap. Source data are available online for this figure.

**Figure 6:**
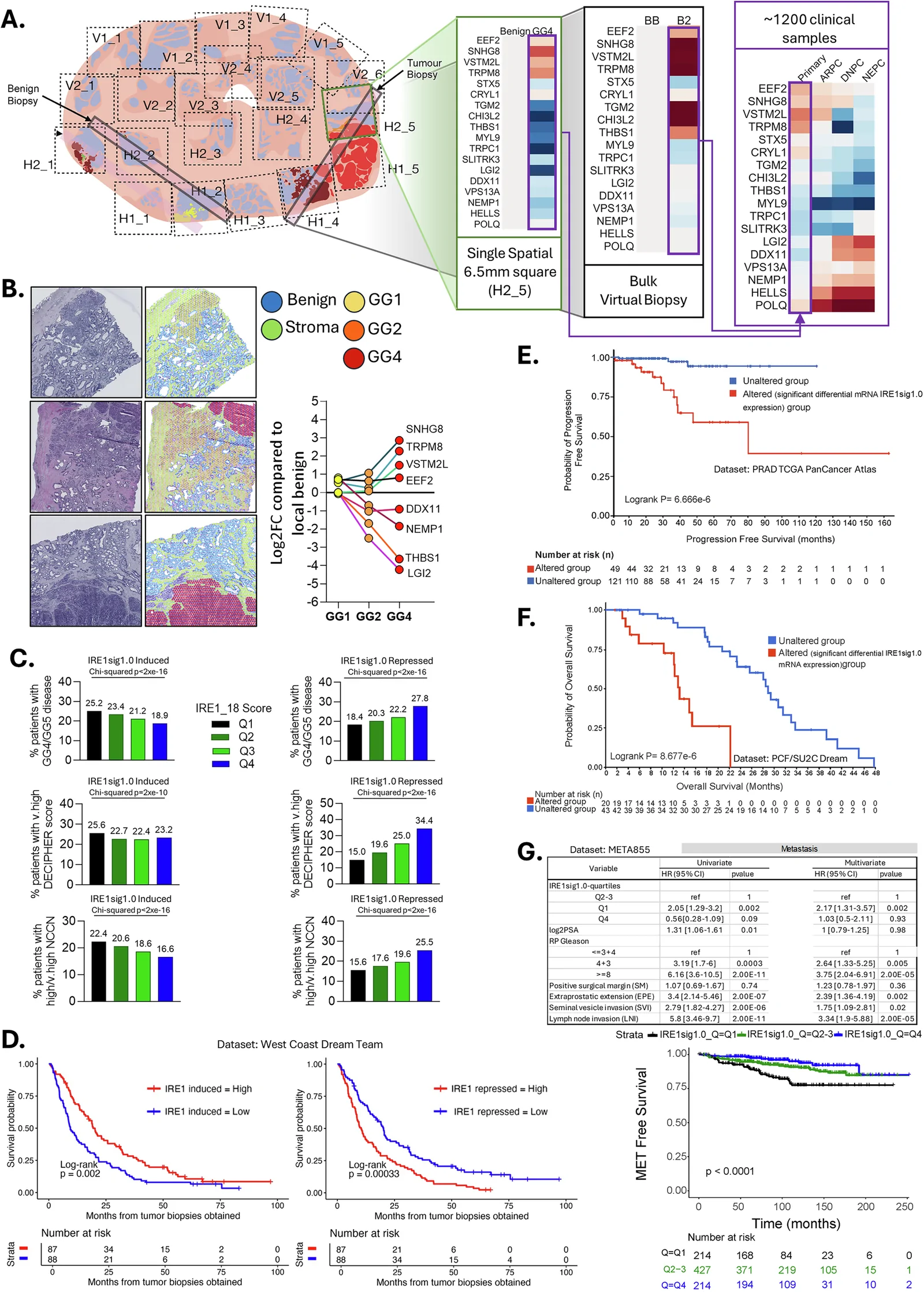
IRE1sig1.0 may act as a proxy of multiple clinical attributes and is prognostic of prostate cancer. (**A**) Schematic of whole radical prostatectomy axial disc with coded dashed squares signifying corresponding spatial transcriptomic 6.5mm2 squares. As previously described three rectangles show virtual biopsy placements corresponding to completely benign or tumour bearing 1.2 mm × 18 mm standard transurethral (TRUS) sampling. From left to right, log fold change expression of the IRE1 activity signature genes is represented in heatmaps locally comparing GG4 to benign (green); comparing a benign virtual biopsy to a tumour-bearing virtual biopsy carrying GG2 and GG4 (grey); and comparing primary, AR-positive CRPC, DNPC and NEPC bulk samples to normal tissue (purple). (**B**) Histological annotations (left) from three separate 6.5 mm^2^ squares from the same radical prostatectomy representing multifocality and heterogeneity. Grade groups (GG) 1 (yellow), 2 (orange) and 4 (red) can be seen. On the right, log fold change in expression of “IRE1 induced” (*EEF2, TRPM8, SNHG8* and* VSTM2L*) and “IRE1 repressed” (*THBS1, LGI2, DDX11* and* NEMP1*) genes when comparing increasing GG to each corresponding benign region. Each locally distinguishing comparison involves 331 GG1 spots, 938 GG2 spots, 1169 GG4 spots and 942, 703 and 1078 benign spots, respectively. Square 3 is also referenced in Figs. 3I and EV9B. (**C**) IRE1sig1.0 induced or repressed expression was divided into quartiles and correlated with GG4/5, very high Decipher score and high/very high NCCN scores. Q1 (black) indicates the lowest expression, while Q4 (blue) indicates the highest expression. Numbers associated with each clinical correlate are displayed above each bar and in the table. Statistical analysis was performed using the chi-squared test. (**D**) IRE1-induced and repressed scores are associated with survival analyses in the WCDT cohort. Kaplan–Meier curves of overall survival for dichotomised IRE1 induced and IRE1 repressed scores based on median value. The red line indicated the high group, while the blue line meant the low group. Shortened versions of the IRE1 activity signature ((*VPS13A, EEF2, TRPM8, POLQ, TGM2, THBS1, LGI2, STX5, TRPC1*), (*VSTM2L, HELLS, CHI3L2, SNHG8, DDX11, SLITRK3, CRYL1, NEMP1* and* MYL9*)) tested for prognostication power showing significance in probability of (**E**) progression-free survival and (**F**) overall survival in a primary disease (PRAD TCGA) and mCRPC (PCF/SU2C) cohort respectively. Altered expression is defined as (**F**) significant log-transformed mRNA z-scores compared to the expression distribution of all samples (RNA Seq FPKM) and (**E**) significant z-scores of tumour samples compared to the expression distribution of all log-transformed mRNA expression of adjacent normal samples in the cohort. Statistical analysis was performed using a log-rank (Mantel–Cox test). (**G**) Univariate and multivariate analysis of IRE1sig1.0 expression quartiles in tumours from a retrospective cohort of 855 patients treated with RP with available long-term follow-up data (META855) respective of metastasis. Kaplan–Meier curves of metastatic-free survival for each IRE1sig1.0 quartile in the META855 cohort.

Consequently, we tested if IRE1sig1.0 could be confounded by heterogeneity. Pseudobulk analysis of tumour-bearing virtual biopsy showed transcriptomic changes attributed to both tumoral and peri-tumoral tissue. Upregulation of *CHI3L2*, *TGM2* and *TRPM8*, *SNHG8*, *VSTM2L* (Fig. EV11A,B) reflected peri-tumoral (*TGM2* and *CHI3L2*) or tumoral (*SNHG8*, *TRPM8* and *VSTM2L*) regions (Fig. EV11C). We proceeded to determine if IRE1sig1.0 could profile a bulk virtual biopsy and place this profile in an exact subgroup of patients within a larger external clinical cohort. Significantly differentially expressed IRE1sig1.0 genes in GG4 compared to local benign in a 6.5 mm (Erickson et al, 2022) ST square, showed the same pattern in a virtual biopsy containing both GG2 and GG4. Since this patient solely underwent radical prostatectomy, we expected their virtual biopsy to correspond to primary disease, and indeed, the expression profile of IRE1sig1.0 corresponds to the primary disease subgroup of the Prostate Cancer Atlas (Fig. 6A).

Thereafter, we correlated the expression of genes within IRE1sig1.0 with increasing grade group in this radical prostatectomy using GG1, GG2 and GG4 spots to recapitulate the full spectrum of multifocality and heterogeneity. IRE1sig1.0_induced genes increased while IRE1sig1.0_repressed genes decreased with tumour grade (Fig. 6B). Expanding on this correlation, we explored the Genomic Resource for Intelligent Discovery research tool (GRID, Veracyte Inc.) comprising ~55,000 transcriptomes from RP specimens. IRE1sig1.0_induced expression negatively correlated with GG4/5 and high/very high NCCN risk groups, while IRE1sig1.0_repressed positively correlated with GG4/5, high/very high NCCN and very high (>0.85) Decipher scores (Fig. 6C; Dataset EV28). We observed similar correlations in smaller cohorts interrogating NEPC scoring, BCR status and mutation count (Appendix Fig. S7A,B; Dataset EV29).

**Figure EV12:**
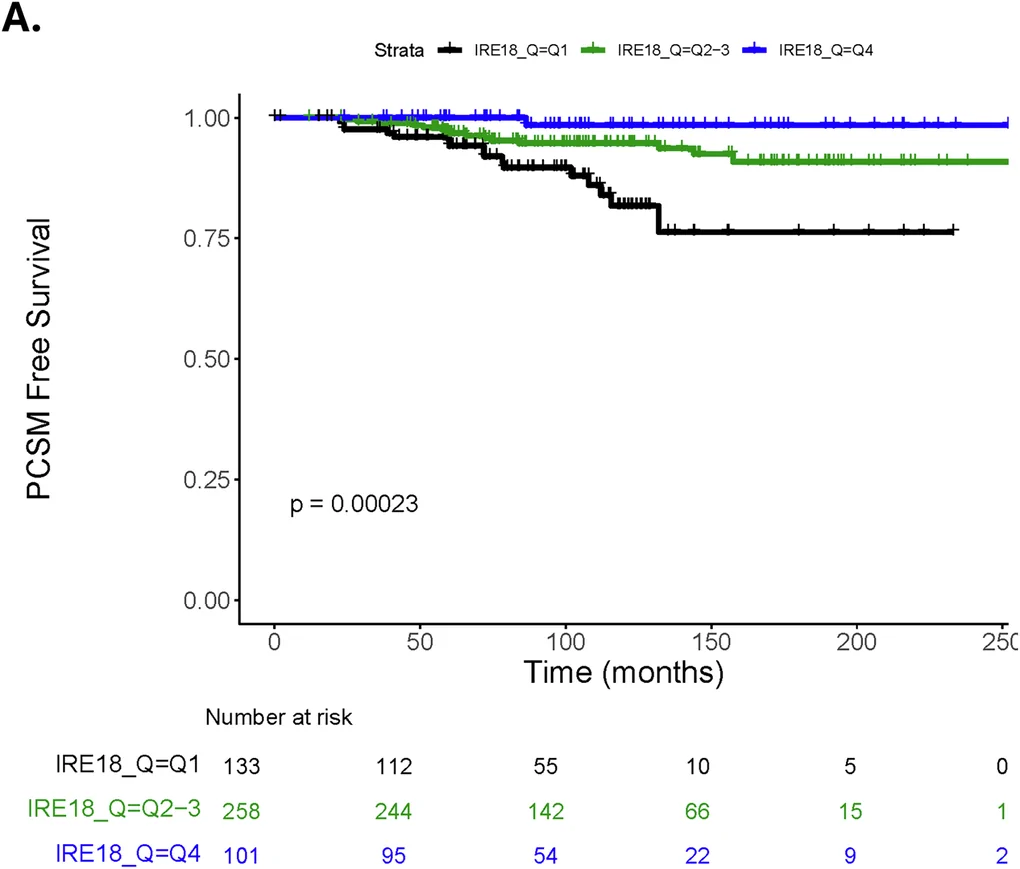
IRE1 activity signature prognosticates prostate cancer specific mortality. (**A**) Univariate and multivariate analysis of IRE1_18 expression quartiles in tumours from a retrospective cohort of 855 patients treated with RP with available long-term follow-up data (META855), with respect to prostate cancer-specific mortality. Kaplan–Meier curves of prostate cancer-specific mortality for each IRE1_18 quartile in the META855 cohort.

Correlations with clinical attributes should translate into prognostication power. Low IRE1sig1.0_induced and high IRE1sig1.0_repressed scores significantly associate with worse survival probability in WCDT (Fig. 6D). Since IRE1sig1.0 genes represent different cell types and biological functions, they should have specific prognostic power in different clinical settings. Indeed, splitting IRE1sig1.0 into two sub-groups comprising both “induced” and “repressed” genes significantly prognosticated both localised (TCGA-PRAD, Fig. 6E) and mCRPC disease (PCF/SU2C, Fig. 6F) for progression free and overall survival respectively. In the PCF/SU2C cohort, patients with significantly differentially expressed mRNA of IRE1sig1.0 had a worse probability of overall survival compared to patients without (Appendix Fig. S7C; Dataset EV30). In the TCGA/PRAD cohort, patients with significantly differentially expressed mRNA of IRE1sig1.0 showed significantly worse probability of progression-free survival, compared to patients without (Appendix Fig. S7D; Dataset EV31). Finally, producing a unified score for IRE1sig1.0, we observed that low IRE1 activity in both multivariate and univariate analysis correlated with significantly worse prognosis, both in terms of metastatic-free survival and prostate cancer-specific mortality (Figs. 6G and EV12A; Dataset EV32).

Therefore, IRE1 activity, represented by IRE1sig1.0, showcases the importance of stress response biology in PCa pathophysiology, progression and treatment response.

## Discussion

We have previously reported that the IRE1-XBP1 axis of the unfolded protein response is regulated by AR activity and that inhibiting IRE1 restricts tumorigenesis in androgen-dependent models of PCa. Sequencing of well-annotated clinical samples has revealed that while AR activity is maintained as PCa progresses to metastasis, AR variant and AR target gene expression supporting progression change. In addition, NEPC/DNPC PCa subtypes do not rely on AR function or expression. By contrast, the UPR/IRE1 axis can adapt as these disease states emerge, becoming a supporting actor of tumour survival that changes according to stresses induced by the tumour environment or treatment. Given that the UPR-IRE1 axis is ubiquitous in all cell types, we have sought to determine whether IRE1 activity contributes to the emergence of acquired treatment resistance by influencing a spectrum of PCa subtypes or cell lineages.

Our study thus introduces two major themes. Theme 1 focuses on IRE1 activity as a tool to inform the use of modulators of IRE1/UPR activity (ONC201 (Amoroso et al, 2021), NXP800 (Welti et al, 2025)) in combination with ADT. This requires systematic mapping of IRE1 activity to AR antagonism responses (Feng et al, 2021; preprint: Parry et al, 2023) and metastatic niches(Labrecque et al, 2019), alongside investigating its direct effects on cell cycle regulators with clinical significance, including RB1 and BRCA2 activity. Theme 2 examines IRE1 activity as a regulator of lineage plasticity. Tracking of differentiated luminal epithelial cells and low-AR, club-like epithelial progenitors, would monitor the emergence of AR-negative basal or stem-like cells (Pitzen et al, 2025) as precursors of treatment resistance.

To assign IRE1 activity to these lineages, we derived an activity signature (IRE1sig1.0) by integrating data derived from 2D (cell lines) and 3D (PDEs) PCa pre-clinical models (Fig. 5). Refinement using clinical datasets ensured retention of genes capable of discriminating between PCa subtypes and predicting disease progression. Mapping IRE1sig1.0 onto spatial transcriptomic clinical datasets, we determined that these genes are associated with a variety of cell types and biological functions likely to reflect niches in which treatment-resistant disease can emerge (Fig. 6). There are currently very few clinically available gene signatures that capture cell-type heterogeneity as well as have some prognostic value - principally Decipher (Kim et al, 2018) and Oncotype DX (Knezevic et al, 2013). Although IRE1sig1.0 is tumour cell–derived, it appears to reflect broader multicellular environments. A refined IRE1sig1.0 may thus be able to identify peri-tumoral tissue in false negative biopsies or inform risk stratification in histologically benign tissue with pre-tumoral genotypes, independent of accurate tumour sampling. Clinically approved genomic tests contain genes attributed to multiple cell types and thus significantly associate with risk of metastasis and PCa-specific mortality (Feng et al, 2021). Consequently, IRE1sig1.0 may enrich this post-diagnostic space, complementing existing genomic assays, and its inclusion within liquid biopsy platforms warrants further investigation.

Our cell-line/pre-clinical models cannot capture the cellular heterogeneity or biological complexity of multicellular niches. However, targeting IRE1 in these simple models has had an impact on their molecular characteristics, acquiring features of club-like epithelia (*MMP7, PIGR, CP* and* LCN2*), and lineage reprogramming (*SOX2, EZH2* and* POU3F2*) (Fig. 3). Similar effects have been observed when the AR is targeted as AR blockade (Joseph et al, 2022) impacts cellular de-differentiation to club lineages histologically associated with inflammatory atrophy (Huang et al, 2023). In parallel, *SOX2* has been associated with stemness, neuroendocrine differentiation and treatment resistance arising from knocking out TP53 and RB1 in androgen-dependent pre-clinical models (Venkadakrishnan et al, 2023). IRE1 activity loss has been associated with the acquisition of cancer stem cell characteristics in glioblastoma through XBP1s-driven repression of mir-148, which de-represses *SOX2* (preprint: Doultsinos et al, 2019). Conversely, club-associated phenotypes are influenced by other stress response mechanisms, as shown in a recent study that documents the integrated stress response (ISR) suppressing anti-tumour immunity(Bossowski et al, 2026) through an ATF4-LCN2 axis. However, to our knowledge, our study is the first example of this impact on lineage arising from targeting a regulator of the UPR in PCa.

Intriguingly, knocking out IRE1 lead to transcriptional changes resembling RB1 loss and oncogenic TF coregulation (Mandigo et al, 2022) while correlating with AR loss and NEPC transcriptomic features in mCRPC datasets (Fig. 2). *RB1* mRNA expression was decreased when blocking IRE1 activity in cell lines while pharmacological IRE1 activation modestly sensitised RB1 deficient cells to physiological and pharmacological androgen deprivation in short term (12 hr) experiments (Fig. 4). Although IRE1 and RB1 loss are not equivalent, we have observed cell cycle deregulation in IRE1 deficient cell lines both at the mRNA and protein levels accounting for mRNA-protein expression discordance. Although IRE1 activity suppression induces reprogramming repertoires, changes in specific club lineage markers in our models may be partly due to altering the balance within the UPR itself as opposed to exclusively due to IRE1-specific events. This is reflected by our observations in variable short-term (siRNA) vs stable long-term (ERN1 KO) IRE1 suppression effects on club marker expression. More work is required to temporally map the influence of all UPR transducers on PCa lineage in the presence of long-term ADT.

In addition, it is important to note that club cells are histologically benign, whereas suppressing IRE1 activity in tumour cells has not reprogrammed them into benign epithelia. Intriguingly, emerging single-cell studies are pointing toward the existence of club-like tumour sub-populations(preprint: Song et al, 2026). The discovery of these tumour cell subtypes may help fully characterise to which extent this IRE1-loss phenotype is a bona fide PCa lineage and elucidate its role in PCa treatment response.

The majority of significant work in the field of lineage plasticity has thus far focused on transcription factors (*ACSL1* and *POU3F2*) and chromatin-modifying enzymes (*EZH2* and *NSD2*). A central priority emerging from our work is to mechanistically determine whether changes in IRE1 activity create permissive states in which these established drivers of lineage plasticity can function more effectively, or whether IRE1’s contribution is mechanistically independent. Addressing this will require integrated temporal and single-cell approaches to map how UPR signalling impacts transcriptional and epigenetic networks during prolonged androgen deprivation in clinically relevant 3D models of disease that include the microenvironment, especially in the context of fully elucidating the relationship with RB1 loss.

Crucially, these studies should be coupled with functional and in vivo interrogation of IRE1 modulators to determine whether pharmacologically targeting IRE1 can prevent or delay the emergence of treatment-resistant, AR-independent states in early disease models or whether it can act as differentiation therapy against advanced disease toward an ADT-sensitive phenotype. In parallel, further refinement and validation of IRE1sig1.0 across tissue and liquid biopsy cohorts will be necessary to establish its utility as a tool for patient stratification and treatment selection. Together, these efforts will determine whether IRE1 represents a viable therapeutic entry point for limiting lineage plasticity and improving outcomes in advanced PCa.

## Methods

**Table:** Reagents and tools table

Reagent/resource	Reference or source	Identifier or catalogue number		
**Experimental models**				
LNCaP	ATCC	CRL-1740		
22RV1	ATCC	CRL-2505		
C4-2B	ATCC	CRL-3315		
LNCaP RB1−/−	P. Nelson			
LNCaP RB1−/−TP53−/−	P. Nelson			
LNCaP ERN1−/−	W. Zwart			
LNCaP ERN1−/− Q780*	D. Doultsinos			
Patient-derived explants	Imperial College Healthcare Tissue Bank (REC 22/WA/0214)			
**Recombinant DNA**				
**Antibodies**				
Antibody	RRID	Manufacturer	Catalogue number	Incubation dilution
Anti-IRE1 (phospho S724) antibody (rabbit)	AB_2231202	Abcam	ab48187	1 in 1000
Anti-XBP1 antibody (rabbit)	AB_778939	Abcam	ab37152	1 in 1000
Anti-CAMKK2 antibody (rabbit)	AB_1845956	Sigma-Aldrich	HPA017389	1 in 1000
Anti-alpha tubulin (DM1A) antibody (mouse)	AB_628412	Santa Cruz	sc-32293	1 in 5000
Monoclonal beta Actin antibody (mouse)	AB_2223210	Abcam	ab6276	1 in 5000
Anti-rabbit IgG, HRP-linked Antibody (goat)	AB_2099233	Cell Signaling	#7074	1 in 2000
Anti-mouse IgG, HRP-linked Antibody (horse)	AB_330924	Cell Signaling	#7076	1 in 2000
Anti-RB1 antibody	AB_823629	Cell Signaling	#9309	1 in 1000
Anti-RBL1 antibody	AB_2800144	Cell Signaling	#89798	1 in 1000
Anti-TP53 antibody	AB_628082	Santa Cruz	sc-126	1 in 1000
Anti-E2F1 antibody	AB_2096936	Cell Signaling	##3742	1 in 1000
Anti-CCNE1 antibody	AB_2783554	Cell Signaling	#20808	1 in 1000
Anti-CCNA2 antibody	AB_3075524	Cell Signaling	#67955	1 in 1000
Anti-CDK4 antibody	AB_3718580	Cell Signaling	#12790	1 in 1000

Oligonucleotides and other sequence-based reagents			
Gene	Direction	sequence	Manufacturer
DGAT2	Forward	GGCTGGTGTTTGACTGGAA	Eurofins Genomics
	Reverse	TCAGCAGGTTGTGTGTCTTC	
ACTIN	Forward	AGAGCTACGAGCTGCCTGAC	
	Reverse	AGCACTGTGTTGGCGTACAG	
XBP1s	Forward	TGCTGAGTCCGCAGCAGGTG	
	Reverse	GCTGGCAGGCTCTGGGGAAG	
ERN1	Forward	GCCACCCTGCAAGAGTATGT	
	Reverse	ATGTTGAGGGAGTGGAGGTG	
POLQ	Forward	CCTGTGGCATCTCCTTGGAGCA	
	Reverse	AATCCCTTGGCTGGTCTCCATC	
KLK3	Forward	CGTGACGTGGATTGGTGC	
	Reverse	ACTGCCCTGCCACGAGAG	
HELLS	Forward	GATTTTGGATCGAATGCTGCCAG	
	Reverse	ATGGACCCATCAAGCCTGCTGA	
AR	Forward	AAGACCTGCCTGATCTGTGG	
	Reverse	CGAAGACGACAAGATGGACA	
CAMKK2	Forward	TGAAGACCAGGCCCGTTTCTACTT	
	Reverse	TGGAAGGTTTGATGTCACGGTGGA	
BRCA2	Forward	AATGTCAGACAAGCTCAAAG	Merck
	Reverse	TCATGTATTTTTCAGGTGGC	
RB1	Forward	GACCCAGAAGCCATTGAAATCT	Eurofins Genomics
	Reverse	GGTGTGCTGGAAAAGGGTCC	
SSR2	Forward	CTTCACCTCGGCAACAATTACT	Sigma Kicqstart
	Reverse	GGGGAGAATCGC CTGTCAAAC	
SOX2	Forward	GAGCTTTGCAGGAAGTTTGC	Eurofins Genomics
	Reverse	GCAAGAAGCCTCTCCTTGAA	
POU3F2	Forward	ACACTGACCGATCTCCACGCAGTA	
	Reverse	GAGGGTGTGGGACCCTAAATATGAC	
EZH2	Forward	AATAAAAGCGATGGCGATTGG	
	Reverse	TCCACTGCCTTCTGAGTCC	
ATF4	Forward	GTTCTCCAGCGACAAGGCTA	
	Reverse	ATCCTGCTTGCTGTTGTTGG	
eIF2α	Forward	GCCTTTCTGTCCTCAGTCAAGC	
	Reverse	CTCTCCATCTGCCTCGCAAGTT	
ATF6	Forward	CTCCGAGATCAGCAGAGGAA	
	Reverse	AATGACTCAGGGATGGTGCT	
LGI2	Forward	TGGAGAACTGCATGGTGCTGGA	
	Reverse	CTTACAGCCCACGATGGACTGA	
VSTM2L	Forward	GATCCAGTGGTGGTATGTACGG	
	Reverse	ACACTTATTTTGGTTGCCTCCTTC	
SNHG8	Forward	AAGTTTACAAGCATGCGCGG	
	Reverse	TCAAACTGACGGTTCTCGGG	
MMP7	Forward	GGAACAGGCTCAGGACTATCTC	
	Reverse	GTCAGCAGTTCCCCATACAA	
LCN2	Forward	GTGAGCACCAACTACAACCAGC	
	Reverse	GTTCCGAAGTCAGCTCCTTGGT	
CP	Forward	CCATCTGAAAGCCGGTTTGC	
	Reverse	ACATGCTTCCCACGG	
PIGR	Forward	CAAGATTATCGAAGGAGAACCAAAC	
	Reverse	CCCGTGTTATTCCACTTGCA	

siRNA target	Sequence		Manufacturer
	Sense	Antisense	
XBP1.1	CCUGCCUACUGGAUGCUUAtt	UAAGCAUCCAGUAGGCAGGaa	Ambion
XBP1.2	AGUGGUAGAUUUAGAAGAAtt	UUCUUCUAAAUCUACCACUtg	Ambion
IRE1.1	GAAACUUCCUUUUACCAUCtt	GAUGGUAAAAGGAAGUUUCgt	Ambion
IRE1.2	CGCUGCUGCUGCCCGGCCUtt	AGGCCGGGCAGCAGCAGCGtc	Ambion
Control	Silencer Select Negative Control #1		Ambion

Chemicals, enzymes and other reagents			
Compound	Manufacturer	Lot number	Concentrations
IXA4	Cayman Chemical	0653659-8	10–25 μM
MKC8866	Cayman Chemical	0664887-3	5 μM
Thapsigargin	Sigma	0000120608	100 nM
Tunicamycin	Sigma	079M4004V	500 nM
Enzalutamide	Selleck Chemicals	MDV3100	10 μM
**Software**			
Prostate Cancer Atlas			
cBioportal			
Correlation AnalyzeR			
CAMCAPP			
**Other**			

### Cell lines and Culture conditions

LNCaP, 22RV1 and C4-2B cells were purchased from ATCC and cultured in RPMI supplemented with 10% foetal bovine serum (FBS) or DMEM supplemented with 10% foetal bovine serum (FBS), respectively. LNCaP knocked out for RB1 or knocked out for both RB1 and TP53 were kindly donated by Professor Peter Nelson (Fred Hutchinson Cancer Centre) and were cultured in RPMI supplemented with 10% foetal bovine serum (FBS). LNCaP knocked out for ERN1 were kindly donated by Professor Wilbert Zwart (The Netherlands Cancer Institute) and were cultured in RPMI supplemented with 10% foetal bovine serum (FBS). No antibiotics were used in the culturing of these cells, as they may have the capacity to bind a variety of kinases, including IRE1, as we previously reported (Doultsinos et al, 2021). For androgen-deprived media, Charcoal-Stripped FBS was used instead of normal FBS to supplement RPMI. Cell lines were authenticated using the AuthentiCell service provided by the UK Health Security Agency and routinely tested for mycoplasma using the MycoFluor Mycoplasma Detection Kit (Invitrogen).

### Patient-derived explant (PDE) tissue culture

PDEs were conducted with previously established protocols (Centenera et al, 2018), with changes to methodology for these studies. RPMI media supplemented with 5% FBS and 1 mM enzalutamide or equivalent vehicle control (VC) was used to soak collagen dental sponges (Surgispon, AnserMedical). From consenting patients, tissue was collected from surgery and immediately transferred to the laboratory in ice-cold RPMI. Tissue was carefully dissected into 1 mm^3^ cubes, and placed onto pre-soaked sponges in 24-well tissue culture plates. An additional 500 ul of media with appropriate treatment was added to each well. After 72-h culture, snapped frozen for RNA analysis. Additionally, matched pieces were formalin fixed and processed for histology as previously described (Leach et al, 2025). RNA for RNA-seq was isolated using the Monarch® kit according to the manufacturer’s instructions (New England Biolabs).

Patient material was collected under Imperial College Healthcare Tissue Bank (REC 22/WA/0214), and the samples for this project (R18041) were issued from sub-collection reference number Uro_MW_13_010 from patients undergoing diagnosis of high-volume primary disease, with an age range between 55 and 88 years old.

### Primers and qPCR

RNA was extracted from cell pellets using TRIzol reagent (Fisher Scientific, #12034977) according to the manufacturer’s instructions. cDNA was reverse transcribed from 2000ng of RNA (measured by Nanodrop) using Maxima Reverse Transcriptase (ThermoFisher Scientific EP0743) and associated reagents (RiboLock-Life Technologies-EO0381; DNTPs-Thermo Scientific-18427013; Random Hexamer-Life Technologies-SO142). qPCR was executed on a QuantStudio5 (Applied Biosystems) using Power SYBR Green PCR master mix (Thermo Fisher Scientific) and results were analysed using the Design & Analysis Software 2.8.3 (Applied Biosystems).

### Drug treatments

Cells were seeded in 96, 24, or 6-well plate format depending on the assay (96-well plate for viability, 96- or 24-well plate for growth, six-well plate for RNA extraction), and drugs were added using the range of concentrations below.

### Cell Titre Glo sensitivity assays

Cells were seeded into 96-well plates and incubated at 37 °C for 24 h. They were then treated with drug combinations for a further 12 h. Percentage survival was measured photometrically using the CellTiter-Glo 2.0 viability assay (Promega) in a microplate reader (FLUOstar OMEGA 415-2812). Survival was normalised to the control treatment.

### Growth assays

Cells were seeded into 96-well plates and incubated at 37 °C °C for 24 h. Complete media change followed by either full (FM) or charcoal-stripped media (CSM) for 120 h. Cell growth was monitored using Incucyte technology, taking confluence measurements every 24 h. Growth was normalised to the initial measurement at time 0.

### Transfection assays and RNA silencing

For stable expression of IRE1 mutant Q780*, LNCaP ERN1^-/-^ cells were transfected with the Q780* construct(Lhomond et al, 2018) using LTX Plus reagent (Thermo Fisher Scientific, 15338100) according to the manufacturer’s instructions. Successfully transfected cells were selected by cell sorting for GFP expression using a BD FACSAriaIII cell sorter. Thereafter, constant GFP expression was monitored using the ZOE^TM^ Fluorescent Cell Imager. Lipofectamine RNAiMax transfection reagent (Thermo Fisher Scientific, 13778150) was used for transfecting siRNAs. Cells were incubated at 37 °C for 48 h before extracting RNA for downstream analysis.

### Immunoblotting and antibodies

Antibodies were used according to the manufacturer’s specifications. Cell pellets were lysed using RIPA buffer (Thermo Fisher Scientific) with the addition of PhoSTOP inhibitors (Sigma- Aldrich). Cell lysates were quantified using the Bio-Rad protein Assay (BIO-RAD) and BSA standards. 30 μg of protein were added to 12% NuPAGE Bis-Tris gels (Thermo Fisher Scientific) in MOPS running buffer and run at 80 V for 1.5 h. Separated proteins were transferred using the iBlot system (Invitrogen). Membranes were blocked in 5% milk and incubated in primary antibody overnight at 4 °C. Washes were carried out using TBS-Tween (Sigma-Aldrich), and membranes were incubated in secondary antibody for 1.5 h at room temperature. Signal was captured using Immobilon Crescendo (Sigma-Aldrich) in a Bio-Rad chemiluminescence docking station.

### Online tools/databases

- cBioportal (RRID:SCR_014555): RNA sequencing datasets investigated included the metastatic prostate adenocarcinoma (SU2C/PCF Dream Team, PNAS 2019 (Abida et al, 2019)), prostate cancer (DKFZ, Cancer Cell 2018 (Gerhauser et al, 2018)), prostate adenocarcinoma (MSK, Cancer Cell 2010 (Taylor et al, 2010)) and prostate adenocarcinoma (TCGA, Firehose Legacy or PanCancer). The comparisons were based on log-transformed mRNA expression z-scores compared to expression distribution of all samples (RNA Seq FPKM) or of tumour samples compared to the expression distribution of all adjacent normal samples in the cohort when available, with a z-score threshold of ±2.0. Altered mRNA was defined as mRNA significantly high or low compared to all or normal samples in each dataset using the z-score threshold. Kaplan–Meier curves were generated for the Prostate Adenocarcinoma (SU2C/PCF Dream Team, PNAS 2019) and Prostate Adenocarcinoma (TCGA SCR_003193, PanCancer) datasets. Only samples from patients with survival data or progression data were used in the generation of these curves, respectively. Samples with any significant alteration in mRNA expression were compared to samples with zero significant changes in the former, whilst samples with significant alterations in at least three genes were compared to samples with zero significant changes in the latter.
- Prostate Cancer Atlas (Bolis et al, 2021): DESeq2 differential expression analysis was carried out for each of sample groups designated as Primary, AR + CRPC, NEPC and DNPC compared to the sample group designated as normal tissue. Differentially significant mRNA expression changes (*p*_adj_ < 0.01) were documented to mine for genes of interest representing IRE1 activity. Pseudotime disease progression plots were generated for “IRE1 induced” and “IRE1 repressed” sub-groups of the IRE1 activity gene set using the in-built functionality of the Prostate Cancer Atlas (https://prostatecanceratlas.org/app/home). Further in-built functionality was used to analyse gene set enrichment of our IRE1 activity gene set in the normal, primary, AR + CRPC, NEPC and DNPC sample groups.
- Correlation AnalyzeR (Miller and Bishop, 2021): The online interface of this tool was used to verify the correlation of the IRE1 activity gene set with other significantly differentially expressed genes in prostate cancer and the biological processes it represents.
- CAMCAPP (Dunning et al, 2017): This tool was used to verify gene by gene correlation of the IRE1 activity gene set with clinical co-variates in three separate clinical cohorts (Stockholm, Cambridge, MSKCC).

### RNA extraction, library prep and sequencing

RNA was extracted from cell line pellets using the RNeasy Plus Mini kit. Samples were QC’d using Nanodrop and the Qubit Flex High Sensitivity ID kit. Libraries were prepared using the TruSeq Stranded Total RNA with Illumina Ribo-Zero plus rRNA Depletion workflow (https://emea.support.illumina.com/downloads/truseq-stranded-total-rna-with-ribo-zero-plus-rrna-depletion-ref-guide.html). Index adaptors, rRNA depletion kit were acquired in addition to the TruSeq Stranded Total Library Prep Gold kit (https://emea.illumina.com/products/by-type/sequencing-kits/library-prep-kits/truseq-stranded-total-rna.html#tabs-bb5d9c771f-item-01fd0b3d08-order). cDNA library QC was carried out on Tapestation and Libraries were pooled, diluted to ∼10 nM, denatured, and further diluted for paired-end sequencing (NovaSeq6000 platform, Illumina, NovaSeq 6000 S2/S4 reagent kit v1.5, 300 cycles). A test run prior to full sequencing was executed on a MiSeq system. Bulk paired-end RNA sequencing was performed to obtain at least 20 mn reads per sample. Raw data were processed using a standardised computational workflow to obtain gene-level raw counts. FASTQ files were quality-checked using FastQC to assess read quality and adaptor contamination. High-quality reads were aligned to the human reference genome (GRCh38, Ensembl release 108) using the STAR aligner (Dobin et al, 2013). Gene-level quantification was performed using HTSeq-count (Anders et al, 2015).

### RNA sequencing data analysis

1. Spatial transcriptomics data were obtained from a previously published dataset derived from radical prostatectomy tissue of a patient diagnosed with multifocal prostate cancer (Nature). Each spatial transcriptomics spot was reviewed by two independent pathologists as part of a consensus approach for pathological annotation.

- Virtual biopsy: The analysis was performed using the same approach as previously described (Molecular Cancer). Gene expression data were extracted from the barcode ID of designed biopsies (LoupeBrowser, v8.0.0) using R (v4.4.0). Libraries were normalised using the spaceranger aggr (v2.0) function, which corrects for batch effects to facilitate the comparison of gene expression data across samples and minimise technical variability. The analysis included seven distinct tissue sections, representing a total of 3721 spatial transcriptomics spots. To maintain data integrity, spots with fewer than 500 unique molecular identifier (UMI) counts were removed. Fold changes were calculated using EdgeR (v3.40.1). as previously described (Figiel et al, 2023).
- Single visium 6.5 mm squares: The raw sequencing reads were directly processed using Space Ranger and mapped using reference genome GRCh38, release 93, as previously described (Erickson et al, 2022). Each histological square was treated as a distinct spatial transcriptomic experiment to determine if, in an independent manner, gene sets could be used as distinguishing tools between histological characteristics. Histological annotations (benign, GG1, GG2, GG4, stroma) were compared using either the locally distinguishing or globally distinguishing functions of the Loupe Browser, which was also used to generate ST images displayed.

2. Single cell: PCa tissue data from Chen et al, were accessed via the GEO online portal (GSM4203181) as a raw count matrix. Quality control filtering was applied based on the criteria described in the original publication: cells with fewer than 200 or more than 5218 detected genes, more than 20% mitochondrial content, and fewer than 56 housekeeping genes were discarded. The filtered data were then converted into an SCE object, separated by patient ID, and normalised using a cluster-based normalisation factor computed after each sample was first partitioned into biologically relevant clusters using the quickCluster(min.size = 100) function from the scran R package (v1.26.2).
  Next, to correct for differences in sequencing depth across batches, all SCE objects were rescaled using the MultiBatchNorm(min.mean = 0.1) function from the Batchelor R package (v1.14.1). Epithelial cells were retrieved using the singleR package (v2.0.0), leveraging the BlueprintEncodeData reference accessed via the celldex package (v1.16.0). Using scran (v1.26.2), variance modelling of gene expression was performed for each sample individually with the modelGeneVar() function; results were merged with combineVar(), and the top 1000 most highly variable genes were selected using getTopHVGs() for subsequent analysis. Batch correction was applied using the fastMNN (k = 300, d = 20) function from the Batchelor package.
  To separate luminal malignant cells from healthy epithelial cell types, clustering was performed on the integrated data using the clusterCells() function from the scran R package (v1.26.2). The clustering was conducted with the Walktrap algorithm, exploring different values of k = 10, which resulted in 14 distinct clusters. All clusters were considered tumour except clusters 7 and 14, which were negative for both tumour and luminal markers (e.g. AMACR, PCA3 and KLK3) and were positive for basal markers such as KRT19, KRT18 and KRT14. Specific cell types, such as club cells, were also distinguished using particular genes using cell-type annotation through the SingleR() function of the SingleR package (v2.0.0), using an SCE object containing cell-type annotations for reference and “de.method = wilcox”. Cell type was defined by overexpression of consensus genes for each type (e.g. club cells PIGR, CP, LCN2, MMP7).
  PCa tissue data from Hirz et al, were accessed via the GEO online portal (GSE181294) as a raw count matrix. Cells passing quality thresholds and cell-type annotation, as defined in the original publication, were retrieved from the annotation table provided by the authors and stored in an SCE object. Similar to the Chen et al, dataset, each sample first underwent cluster-based normalisation followed by inter-sample correction to account for sequencing depth differences between batches.
3. Bulk: The RNA sequencing data (raw counts) of the Labrecque mCRPC patient cohort (accession number GSE126078) and the RB1^−/−^/TP53^−/−^ cell line dataset (accession number GSE147250) was retrieved from GEO. TCGA data were accessed via the GDC Data Portal (https://portal.gdc.cancer.gov/projects/TCGA-PRAD, TCGA-PRAD.2022-04-27). The Beltran patient cohort FPKM data were obtained from the supplementary material of Data Ref: (Beltran et al, 2016).

Data visualisation and statistical analyses of the bulk RNA sequencing datasets were performed using R statistical software (v4.4.1). Batch effect correction was executed using the sva package (v.3.54.0). Differential gene expression analysis was performed using the DESeq2 package (v1.40.2), with subsequent LFC shrinkage using the ‘apeglm’ estimator. Gene set enrichment analysis (GSEA) was conducted on a subset of the Molecular Signatures Database (MSigDB) v2023.2.Hs using the clusterProfiler package (v4.8.2). Gene Set Variation Analysis (GSVA) was used for single-sample pathway enrichment as implemented in the GSVA package (v1.48.3). Differential expression at the pathway level was assessed using the lmFit function from limma (v3.62.2). For visualisation and clustering purposes, the variance stabilising transformation (VST) was applied using the DESeq2 package. Visualisation was performed using ggplot2 (v3.5.0), and heatmaps were generated using the pheatmap package (v1.0.12). The viper R package (v1.4.0) was used for single-sample regulon enrichment and differential testing of regulon activity levels between sample groups. The regulons of 1695 human transcription factors were previously inferred using ARACNE -AP on an in-house set of mCRPC patient tumours. Unless otherwise specified, the statistical significance threshold across all analyses was set at 0.05, and a Benjamini–Hochberg correction was applied to account for multiple testing.

### West Coast Dream Team cohort

All analyses were performed in R 4.3.1, using RStudio. Transcripts Per Million (TPM) read counts matrix of tissue RNA-seq data from Western Coast Dream Team (WCDT) cohort were used as inputs to calculate single-sample gene set enrichment scores for each gene set and performed with the GSVA R package (version 1.50.5; (4)). The difference between paired groups was performed with the Wilcoxon signed-rank test. The Kruskal-Wallis test was applied to compare variables among multiple groups. Multiple tests were corrected with Bonferroni. Correlation analyses were performed with Spearman’s correlation. Box plots should be interpreted as follows: horizontal lines represent median values; the boxes range from the 25th to the 75th percentile of each group’s distribution; the vertical lines indicate adjacent values, which are the most extreme values within 1.5 times the interquartile range from the 25th and 75th percentiles of each group. Overall survival from the time of tumour biopsy acquisition was compared between groups in the WCDT cohort. Survival analyses were performed and visualised with the survival and survminer packages in R, and survival probability was visualised with the Kaplan–Meier curves (5). Hazard ratios were modelled using the Cox proportional hazards model, and differences were tested using the Wald test. Log2-transformed z-scores were calculated for each gene to determine significance relative to all samples with a cut-off of ±2.0. The altered mRNA group was defined as having at least one gene with significantly high or low expression within each mix signature gene set.

### 55 K GRID and META855

A prospectively collected cohort of radical prostatectomy (RP) tumour samples (*n* = 55,329) tested with the Decipher prostate genomic classifier between 2016 and 2024 was interrogated to assess the association of IRE1 with genomic and pathological variables. De-identified transcriptomic and clinical data were retrieved from the Genomics Resource for Intelligent Discovery (GRID) registry (ClinicalTrials.gov ID: NCT02609269). Patient data was de-identified in accordance with the Safe Harbour method described in the HIPAA Privacy Rule 45 CFR 164.514(b) and (c) (Veracyte, Inc., San Diego, CA) prior to analysis.

Additionally, we used a retrospective cohort of 855 samples (META855) from patients who underwent RP and had a long follow-up time. This cohort was previously used to validate the Decipher genomic classifier [Spratt et al, (2017)]. The primary endpoint in this cohort was distant metastasis.

### Patient samples

Informed consent was obtained from all participants who donated tissue for the generation of explants at Imperial College London as part of a larger collection. Patient material was collected under Imperial College Healthcare Tissue Bank (REC 22/WA/0214), and the samples for this project (R18041) were issued from sub-collection reference number Uro_MW_13_010 from patients undergoing diagnosis of high-volume primary disease, with an age range between 55 and 88 years old. Experiments using the explants conform to the principles set out in the WMA Declaration of Helsinki and the Department of Health and Human Services Belmont Report.

### Graphics

Figure EV6A; Appendix Fig. S6A,E and main Figs. 3E,I, 4C, 5F, and 6A were created using Biorender.com.

## Supplementary information

Appendix

Peer Review File

Datasets EV1–EV32

Dataset EV33

Source data Fig. 1

Source data Fig. 2

Source data Fig. 3

Source data Fig. 4

Source data Fig. 5

Figure EV2 Source Data

Figure EV3 Source Data

Figure EV5 Source Data

Figure EV7 Source Data

Figure EV8 Source Data

Figure EV10 Source Data

Figure EV11 Source Data

Expanded View Figures

## Data Availability

Any unpublished data discussed in this publication have been deposited in NCBI’s Gene Expression Omnibus (Edgar et al, 2002) and are accessible through GEO Series accession number GSE297633. The source data of this paper are collected in the following database record: biostudies:S-SCDT-10_1038-S44321-026-00490-w.
