# Supplementary material for: IRE1 activity is prognostic in localised and metastatic prostate cancer and affects epithelial lineage states
Source: EMBO Mol Med. 2026 Aug 22;18(10):3898–938. doi: 10.1038/s44321-026-00490-w (PMC13658804; doi:10.1038/s44321-026-00490-w)
Supplement: Supplementary file 1 [file 44321_2026_490_MOESM17_ESM.pdf]

# Expanded View Figures

**Figure EV1. IRE1 activity is suppressed by physiological Androgen deprivation.**

(**A**, **B**) Expression heatmaps of selected "IRE1 induced" and "IRE1 repressed" genes according to the literature in LNCaP cells in CSM (green) vs FM (purple), (**A**); and R1881 (yellow) vs EtOH (orange) (**B**). The colour scale represents the expression z-score from black (lowest) to red (highest). (**C–E**) mRNA fold change of androgen receptor activity markers (full-length AR, CAMKK2 and KLK3), IRE1 activity markers (ERN1, XBP1s, DGAT2 and BLOC1S1), and PERK activity markers (eIF2α and ATF4) in LNCaP parental cells grown over 72 h in CSM, normalised to untreated FM control ($n = 3$). Statistical analysis was performed using an ordinary one-way ANOVA with Dunnett's multiple comparisons test and single-pooled variance. (**F**) Blots of lysates derived from LNCaP cells grown in CSM for 72 h showing protein levels of IRE1α, CAMKK2, XBP1 and Tubulin with accompanying densitometry. Each time point was normalised to the housekeeping tubulin, and then the values were normalised to the 0-h time point for each of IRE1, XBP1 and CAMKK2. (**G**) mRNA fold change of AR and IRE1 activity markers KLK3, CAMKK2, XBP1s, DGAT2, ERN1, BLOC1S1 in patient-derived explants (PDEs) treated with dihydrotestosterone (DHT), normalised to PDEs treated with vehicle control ($n = 8$). Statistical analysis was performed using a *T*-test per condition.
Data information: Data were presented as mean ± SD. ns not significant, $^{*}P \leq 0.05$, $^{**}P \leq 0.01$, $^{***}P \leq 0.001$, $^{****}P \leq 0.0001$.


© The Author(s)

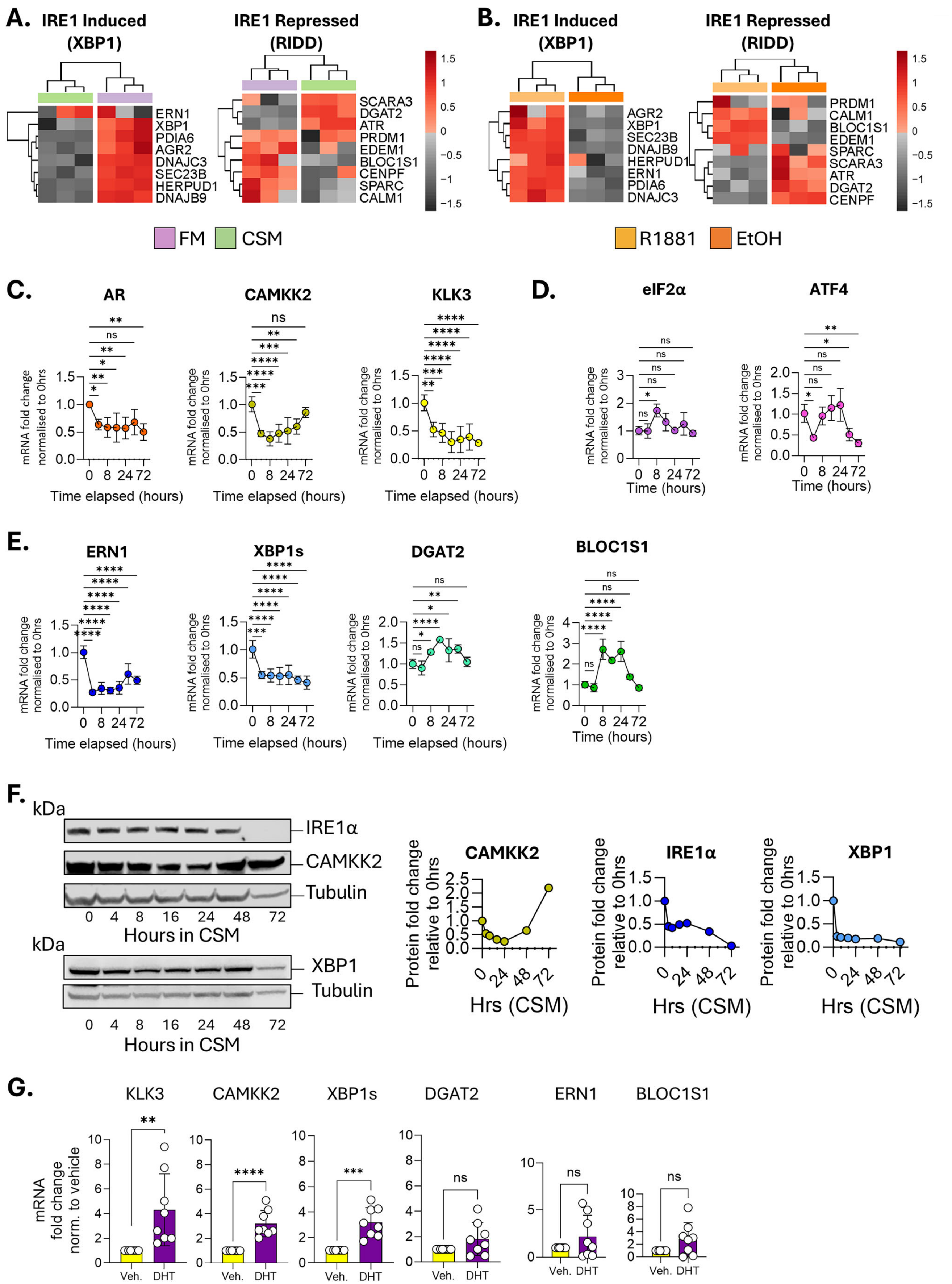
A.
IRE1 Induced (XBP1)
IRE1 Repressed (RIDD)
ERN1
XBP1
PDIA6
AGR2
DNAJC3
SEC23B
HERPUD1
DNAJB9
SCARA3
DGAT2
ATR
PRDM1
EDEM1
BLOC1S1
CENPF
SPARC
CALM1
FM
CSM
B.
IRE1 Induced (XBP1)
IRE1 Repressed (RIDD)
R1881
EtOH
C.
AR
CAMKK2
KLK3
mRNA fold change normalised to 0hrs
Time elapsed (hours)
D.
eIF2α
ATF4
Time (hours)
E.
ERN1
XBP1s
DGAT2
BLOC1S1
F.
kDa
IRE1α
CAMKK2
Tubulin
XBP1
Hours in CSM
Protein fold change relative to 0hrs
Hrs (CSM)
G.
KLK3
CAMKK2
XBP1s
DGAT2
ERN1
BLOC1S1
mRNA fold change norm. to vehicle
Veh.
DHT

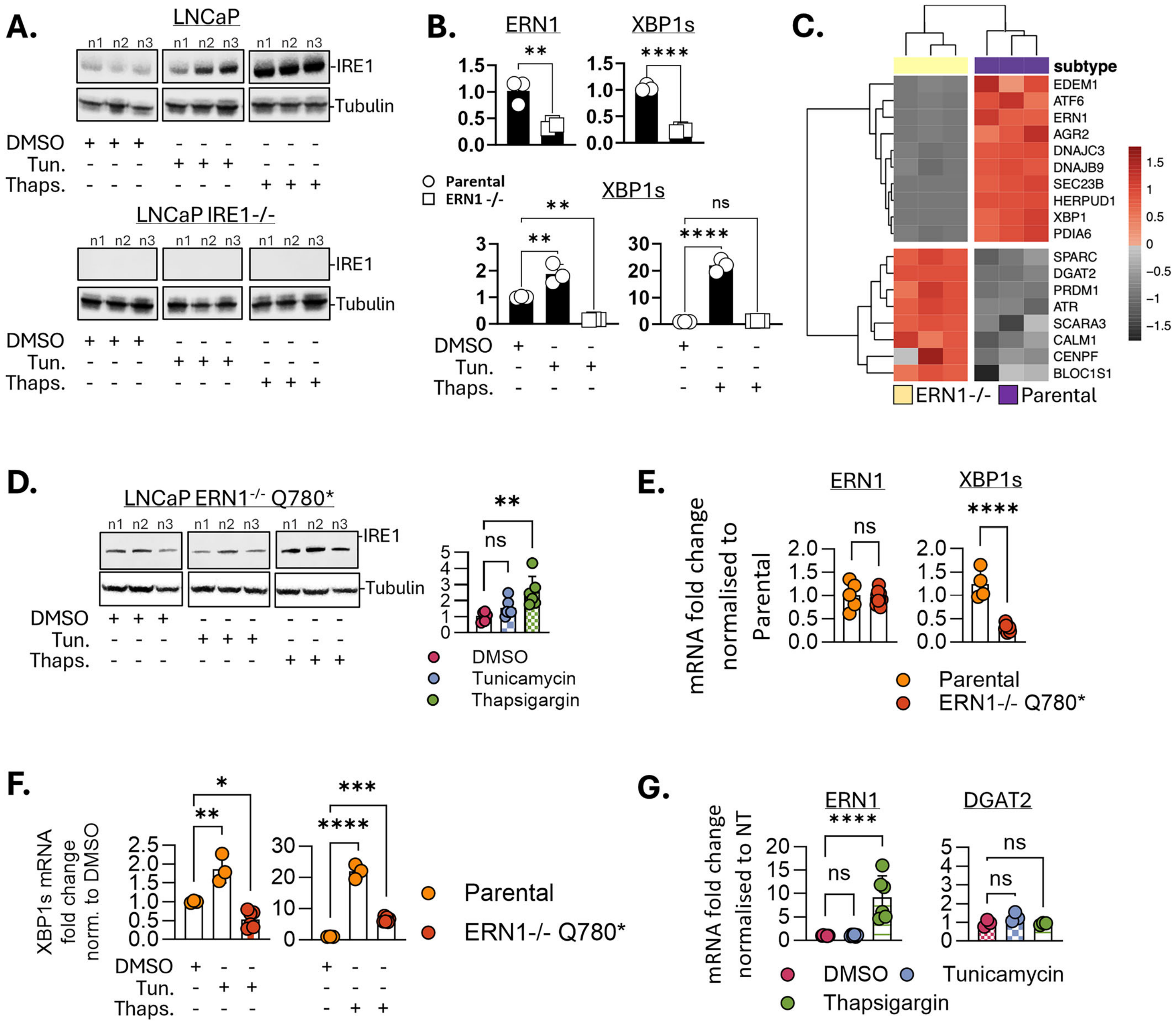

**Figure EV2. LNCaP IRE1 deficient cells have limited ER Stress response.**

(**A**) Western blots in triplicate for IRE1α of LNCaP parental (top) or LNCaP ERN1−/− cells (bottom) in the presence of ER stressors tunicamycin or thapsigargin (100 nM). The DMSO control is also shown. (**B**) mRNA fold change of ERN1, XBP1s in basal conditions (FM, top) or in the presence of tunicamycin or thapsigargin (bottom) in LNCaP parental or LNCaP ERN1−/− cells ($n = 3$). Top: Statistical analysis was performed using T-tests. Bottom: Statistical analysis was performed using an ordinary one-way ANOVA with Dunnett's multiple comparisons test and single-pooled variance. (**C**) Heatmap of canonical IRE1-XBP1 or IRE1-RIDD targets in RNAseq data derived from LNCaP parental (purple) or LNCaP ERN1−/− (yellow) cells. (**D**) Western blots in triplicate for IRE1α of LNCaP ERN1−/− Q780* cells in the presence of ER stressors tunicamycin or thapsigargin (100 nM). The DMSO control is also shown. Accompanying densitometry measures the impact of ER stress on the protein levels of the IRE1 mutant protein. (**E**) mRNA fold change of ERN1 and XBP1s in LNCaP parental and LNCaP ERN1−/− Q780* cells under basal conditions (FM) ($n = 6$). Statistical analysis was performed using a *T*-test per condition. (**F**) IRE1 induced activity marker XBP1s mRNA fold change in LNCaP parental or LNCaP ERN1−/− Q780* cells treated with DMSO, Tunicamycin or Thapsigargin, normalised to parental cells treated with DMSO ($n = 3$). Statistical analysis was performed using an ordinary one-way ANOVA with Dunnett's multiple comparisons test and single-pooled variance. (**G**) IRE1 repressed activity marker DGAT2 and ERN1 mRNA fold change in LNCaP ERN1−/− Q780* cells treated with DMSO, Tunicamycin or Thapsigargin, normalised the DMSO condition ($n = 6$). Statistical analysis was performed using an ordinary one-way ANOVA with Dunnett's multiple comparisons test and single-pooled variance. Data information: Data were presented as mean ± SD. ns not significant, *$P \le 0.05$, **$P \le 0.01$, ***$P \le 0.001$, ****$P \le 0.0001$. Source data are available online for this figure.


© The Author(s)

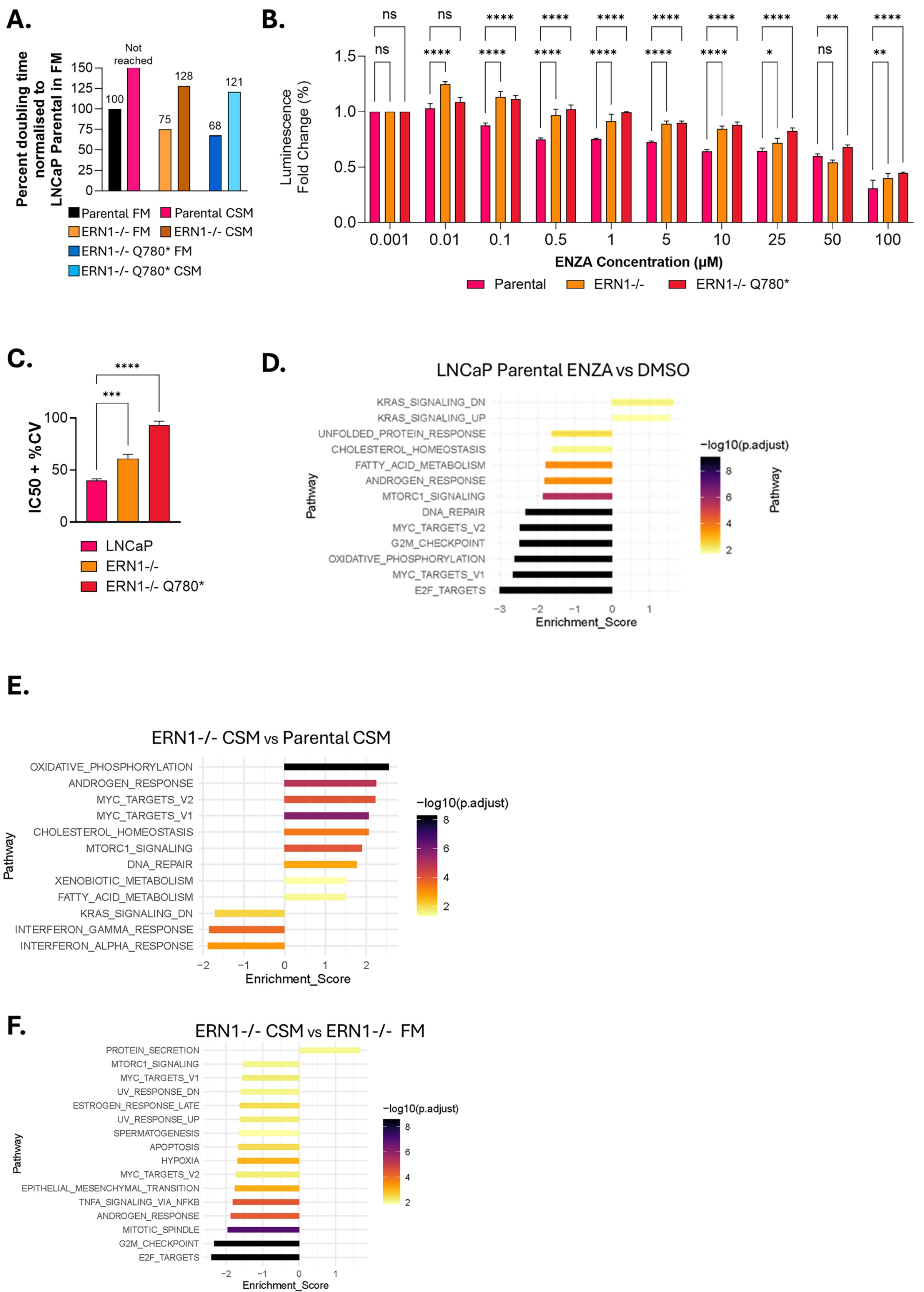
A.
Percent doubling time normalised to LNCaP Parental in FM
Not reached
100
75
128
68
121
Parental FM
Parental CSM
ERN1-/- FM
ERN1-/- CSM
ERN1-/- Q780* FM
ERN1-/- Q780* CSM
B.
Luminescence Fold Change (%)
ENZA Concentration (μM)
0.001 0.01 0.1 0.5 1 5 10 25 50 100
Parental
ERN1-/-
ERN1-/- Q780*
C.
IC50 + %CV
LNCaP
ERN1-/-
ERN1-/- Q780*
D.
LNCaP Parental ENZA vs DMSO
KRAS_SIGNALING_DN
KRAS_SIGNALING_UP
UNFOLDED_PROTEIN_RESPONSE
CHOLESTEROL_HOMEOSTASIS
FATTY_ACID_METABOLISM
ANDROGEN_RESPONSE
MTORC1_SIGNALING
DNA_REPAIR
MYC_TARGETS_V2
G2M_CHECKPOINT
OXIDATIVE_PHOSPHORYLATION
MYC_TARGETS_V1
E2F_TARGETS
Pathway
Enrichment_Score
−log10(p.adjust)
E.
ERN1-/- CSM vs Parental CSM
OXIDATIVE_PHOSPHORYLATION
ANDROGEN_RESPONSE
MYC_TARGETS_V2
MYC_TARGETS_V1
CHOLESTEROL_HOMEOSTASIS
MTORC1_SIGNALING
DNA_REPAIR
XENOBIOTIC_METABOLISM
FATTY_ACID_METABOLISM
KRAS_SIGNALING_DN
INTERFERON_GAMMA_RESPONSE
INTERFERON_ALPHA_RESPONSE
Pathway
Enrichment_Score
−log10(p.adjust)
F.
ERN1-/- CSM vs ERN1-/- FM
PROTEIN_SECRETION
MTORC1_SIGNALING
MYC_TARGETS_V1
UV_RESPONSE_DN
ESTROGEN_RESPONSE_LATE
UV_RESPONSE_UP
SPERMATOGENESIS
APOPTOSIS
HYPOXIA
MYC_TARGETS_V2
EPITHELIAL_MESENCHYMAL_TRANSITION
TNFA_SIGNALING_VIA_NFKB
ANDROGEN_RESPONSE
MITOTIC_SPINDLE
G2M_CHECKPOINT
E2F_TARGETS
Pathway
Enrichment_Score
−log10(p.adjust)

**Figure EV3. Response of IRE1 deficient LNCaP cells to Androgen deprivation compared to parental cells.**

(**A**) Growth curves of LNCaP parental, LNCaP ERN1$^{-/-}$ and LNCaP ERN1−/− Q780* cells grown over 120 h in FM or CSM. Confluence fold change was normalised to Incucyte readings taken at the 0-h time point of each condition. Exponential (Malthusian) growth curve equations were used to estimate the doubling time of each cell line in FM or CSM. (**B**) Cell Titre Glo signal fold change in LNCaP, LNCaP ERN1−/− and LNCaP ERN1−/− Q780* cells treated with increasing doses of ENZA over 12 h ($n = 3$). All values are normalised to equivalent DMSO controls for each condition. Statistical analysis was performed using an ordinary one-way ANOVA with Dunnett's multiple comparisons test and single-pooled variance. (**C**) Bar chart represents the ENZA IC50 comparison between the cell lines. Maximum variance is used to calculate significance by ordinary one-way ANOVA with multiple comparisons. (**D**) GSEA normalised enrichment scores (NES) of LNCaP parental cells treated with 10 μM ENZA for 12 h compared to DMSO or (**E**) of LNCaP ERN1−/− cells grown in CSM for 72 h compared to parental LNCaP cells grown in the same conditions or (**F**) of LNCaP ERN1−/− cells grown in CSM for 72 h compared to LNCaP ERN1−/− cells grown in FM for 72 h. Bar length indicates the normalised enrichment score (NES) and bar colour signifies the statistical significance. Data information: Data were presented as mean ± SD. ns not significant, *$P \leq 0.05$, **$P \leq 0.01$, ***$P \leq 0.001$, ****$P \leq 0.0001$. Source data are available online for this figure.

 © The Author(s)

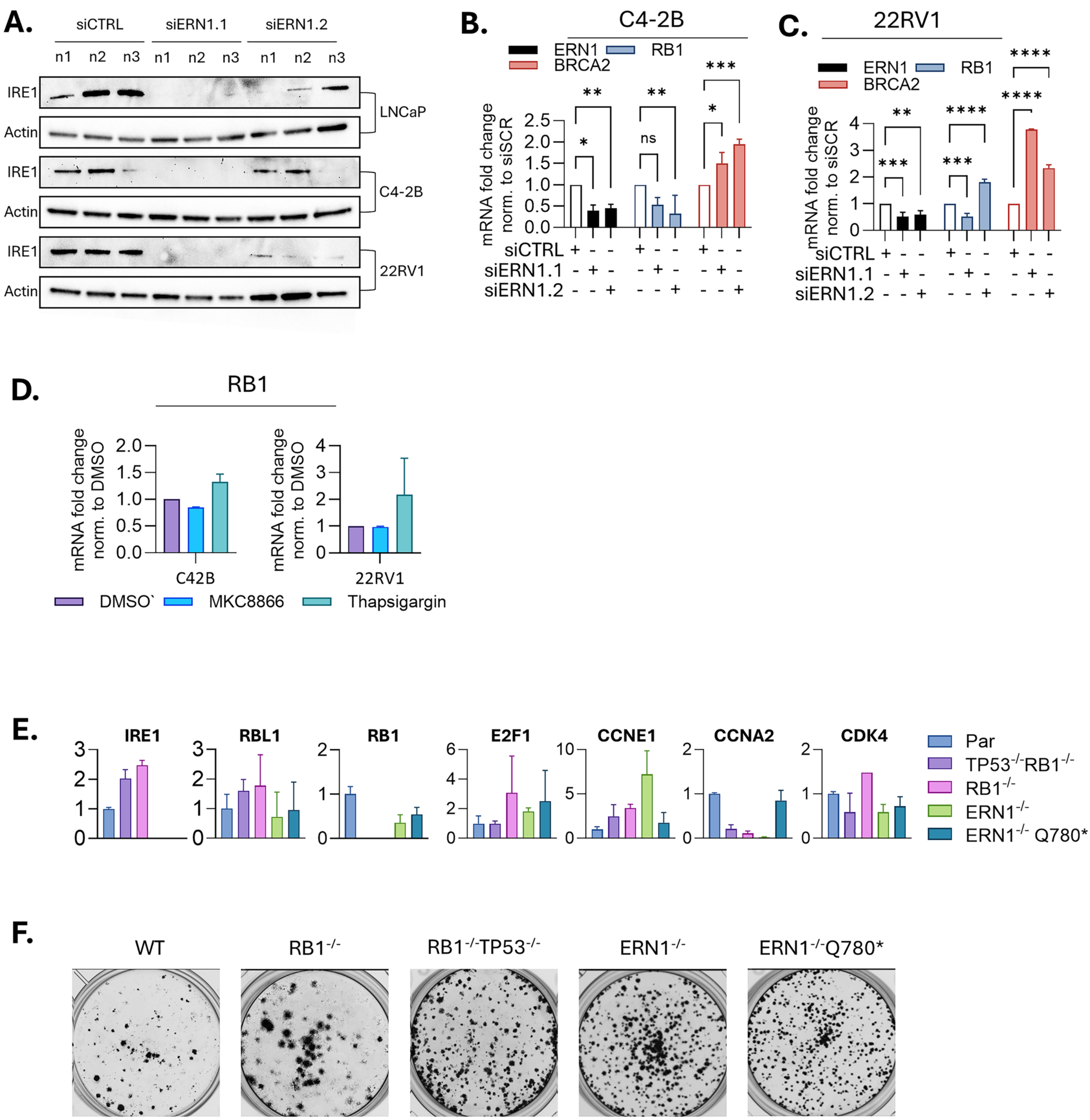


**Figure EV4. Genetic IRE1 modulation effect on RB1 expression, E2F1 target expression and cell clonogenic capacity.**

(**A**) Western Blots of IRE1 and Actin in LNCaP, C4-2B and 22RV1 cells treated with siCtrl or two separate siRNAs against ERN1. mRNA fold change of ERN1, RB1, BRCA2 in (**B**) C4-2B parental cells and (**C**) 22RV1 parental cells two siRNAs against ERN1 or XBP1 for 48 h normalised to scramble control ($n = 3$). (**D**) RB1 mRNA fold change in C4-2B and 22RV1 cells treated with MKC8866 (5 μM) and Thapsigargin (100 nM) for 5 h normalised to DMSO ($n = 2$). Statistical analysis was performed using an ordinary two-way ANOVA and Sidak's multiple comparisons test with a single pooled variance. (**E**) Densitometry of blots presented in main Fig. 2 using Fiji by ImageJ. (**F**) Representative images of clonogenic assays used to measure surviving fractions presented in main Fig. 2. Data information: Data were presented as mean ± SD. ns not significant, $*P \leq 0.05$, $**P \leq 0.01$, $***P \leq 0.001$, $****P \leq 0.0001$.

© The Author(s)

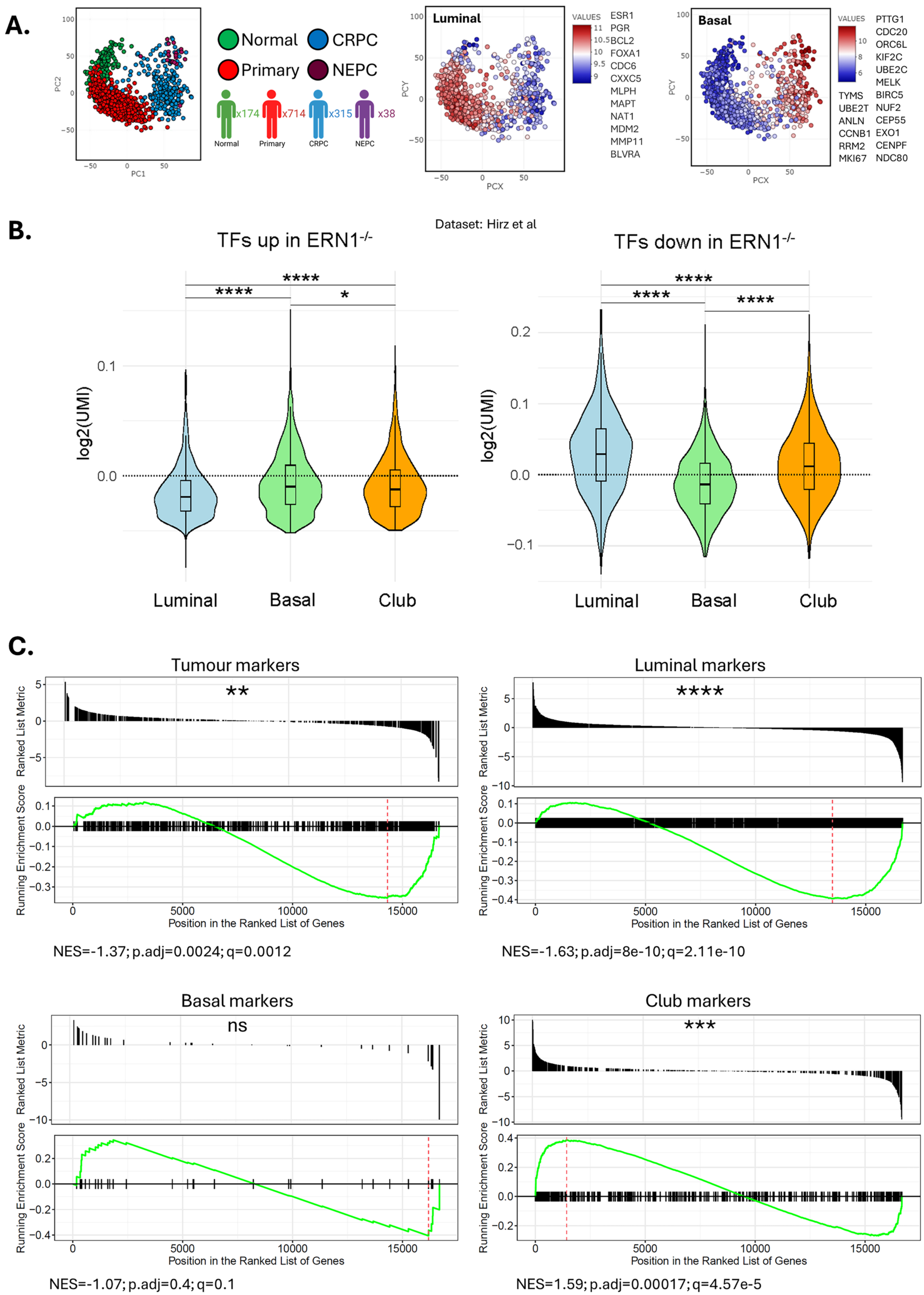
A.
Normal
CRPC
Primary
NEPC
x174
x714
x315
x38
Luminal
ESR1
PGR
BCL2
FOXA1
CDC6
CXXC5
MLPH
MAPT
NAT1
MDM2
MMP11
BLVRA
Basal
PTTG1
CDC20
ORC6L
KIF2C
UBE2C
MELK
TYMS
BIRC5
UBE2T
NUF2
ANLN
CEP55
CCNB1
EXO1
RRM2
CENPF
MKI67
NDC80
B.
Dataset: Hirz et al
TFs up in ERN1-/-
TFs down in ERN1-/-
log2(UMI)
Luminal
Basal
Club
C.
Tumour markers
Luminal markers
Basal markers
Club markers
Ranked List Metric
Running Enrichment Score
Position in the Ranked List of Genes
NES=-1.37; p.adj=0.0024; q=0.0012
NES=-1.63; p.adj=8e-10; q=2.11e-10
NES=-1.07; p.adj=0.4; q=0.1
NES=1.59; p.adj=0.00017; q=4.57e-5

 © The Author(s)

**Figure EV5. IRE1 deficient cells are enriched for club but deficient for tumour or luminal transcriptomic repertoires.**

(**A**) Basal/epithelial transcriptomic signatures in a comprehensive prostate cancer transcriptome atlas of integrated RNA-seq datasets from over 1000 clinical tissue samples ranging from benign (green) to NEPC (purple). Heatmaps show the distribution of signal (low/blue; high/red) of these signatures across the dataset. (**B**) Violin plots showing the enrichment of transcription factors significantly deregulated in the absence of IRE1 activity in basal cells compared to luminal epithelial cells in a single cell dataset. (**C**) Gene set enrichment plots of gene sets representing tumour, luminal epithelial, basal epithelial and club epithelial cells in the LNCaP ERN1−/− cells compared to parental.

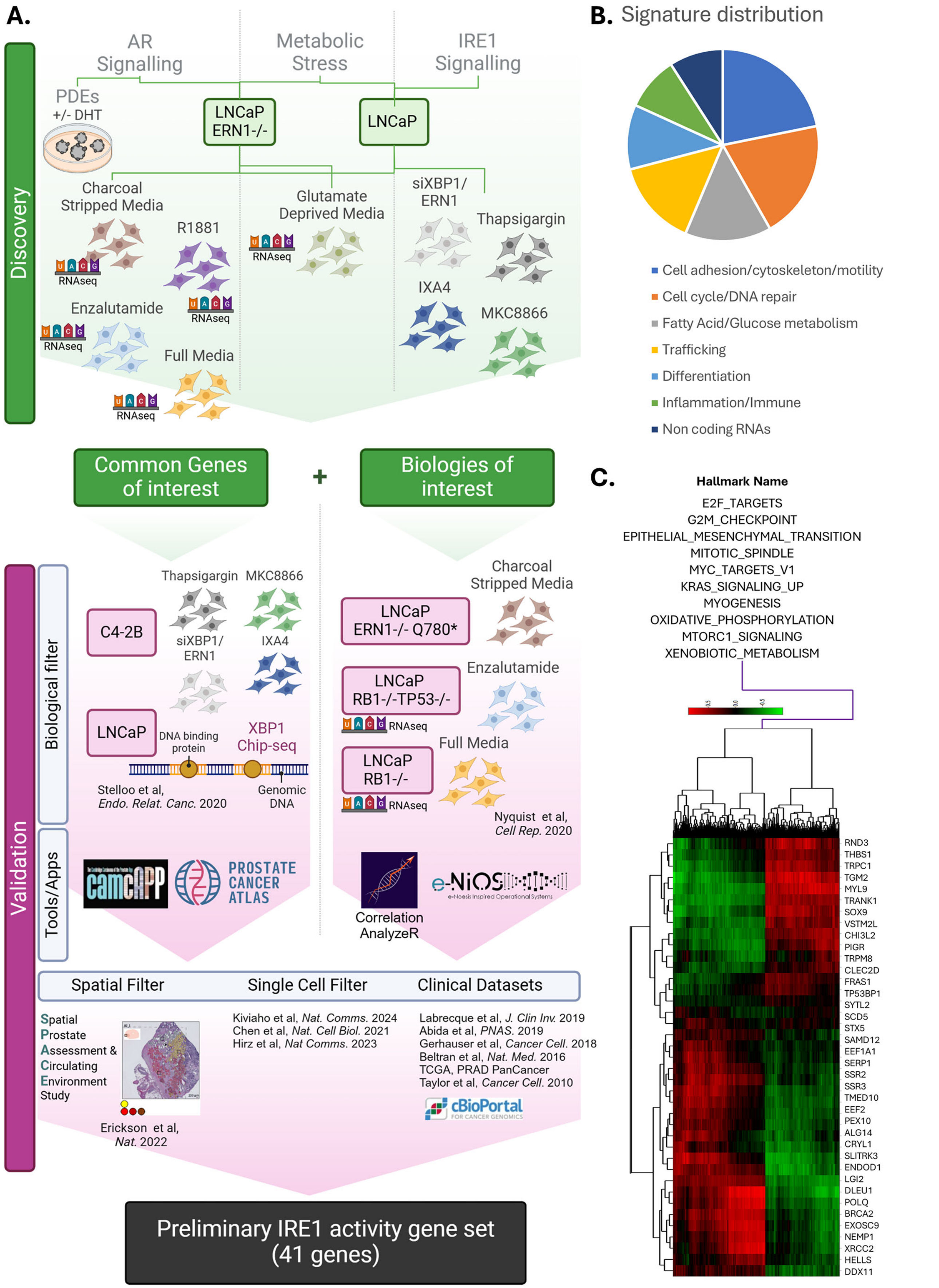
A.
Discovery
AR Signalling
Metabolic Stress
IRE1 Signalling
PDEs
+/- DHT
LNCaP ERN1-/-
LNCaP
Charcoal Stripped Media
R1881
Glutamate Deprived Media
siXBP1/ ERN1
Thapsigargin
RNAseq
Enzalutamide
IXA4
MKC8866
Full Media
Common Genes of interest
+
Biologies of interest
Validation
Biological filter
Tools/Apps
C4-2B
Thapsigargin
MKC8866
siXBP1/ ERN1
IXA4
LNCaP
DNA binding protein
XBP1 Chip-seq
Stelloo et al, Endo. Relat. Canc. 2020
Genomic DNA
Charcoal Stripped Media
LNCaP ERN1-/- Q780*
Enzalutamide
LNCaP RB1-/-TP53-/-
Full Media
LNCaP RB1-/-
Nyquist et al, Cell Rep. 2020
camcAPP
PROSTATE CANCER ATLAS
Correlation AnalyzeR
e-NIOS
e-Noesis Inspired Operational Systems
Spatial Filter
Single Cell Filter
Clinical Datasets
Spatial Prostate Assessment & Circulating Environment Study
Erickson et al, Nat. 2022
Kiviaho et al, Nat. Comms. 2024
Chen et al, Nat. Cell Biol. 2021
Hirz et al, Nat Comms. 2023
Labrecque et al, J. Clin Inv. 2019
Abida et al, PNAS. 2019
Gerhauser et al, Cancer Cell. 2018
Beltran et al, Nat. Med. 2016
TCGA, PRAD PanCancer
Taylor et al, Cancer Cell. 2010
cBioPortal FOR CANCER GENOMICS
Preliminary IRE1 activity gene set (41 genes)
B. Signature distribution
Cell adhesion/cytoskeleton/motility
Cell cycle/DNA repair
Fatty Acid/Glucose metabolism
Trafficking
Differentiation
Inflammation/Immune
Non coding RNAs
C.
Hallmark Name
E2F_TARGETS
G2M_CHECKPOINT
EPITHELIAL_MESENCHYMAL_TRANSITION
MITOTIC_SPINDLE
MYC_TARGETS_V1
KRAS_SIGNALING_UP
MYOGENESIS
OXIDATIVE_PHOSPHORYLATION
MTORC1_SIGNALING
XENOBIOTIC_METABOLISM
RND3
THBS1
TRPC1
TGM2
MYL9
TRANK1
SOX9
VSTM2L
CHI3L2
PIGR
TRPM8
CLEC2D
FRAS1
TP53BP1
SYTL2
SCD5
STX5
SAMD12
EEF1A1
SERP1
SSR2
SSR3
TMED10
EEF2
PEX10
ALG14
CRYL1
SLITRK3
ENDOD1
LGI2
DLEU1
POLQ
BRCA2
EXOSC9
NEMP1
XRCC2
HELLS
DDX11


© The Author(s)

◀ **Figure EV6. Integration of multiple transcriptomic datasets produces IRE1 activity signature.**

(**A**) Schematic showing the –omics datasets involved in generating the IRE1 activity gene set. Green lines signify internally generated datasets forming the Discovery component. External and internal datasets used to filter the signature forming the Validation component are highlighted in pink. (**B**) Pie chart indicating the biologies represented within the new IRE1 gene set. These biologies are the result of a GSEA analysis of the final gene list. (**C**) Heatmap correlating expression of the 41 genes in the IRE1 activity gene set with 500 genes significantly differentially expressed in prostate cancer. The hallmarks these 500 genes represent are displayed above the heatmap. This heatmap was generated using the Correlation AnalyzeR shiny app with the input being the list of 41 IRE1 activity genes, in the topology analysis suite and selecting the tissue type as prostate and sample type as cancer.

A.

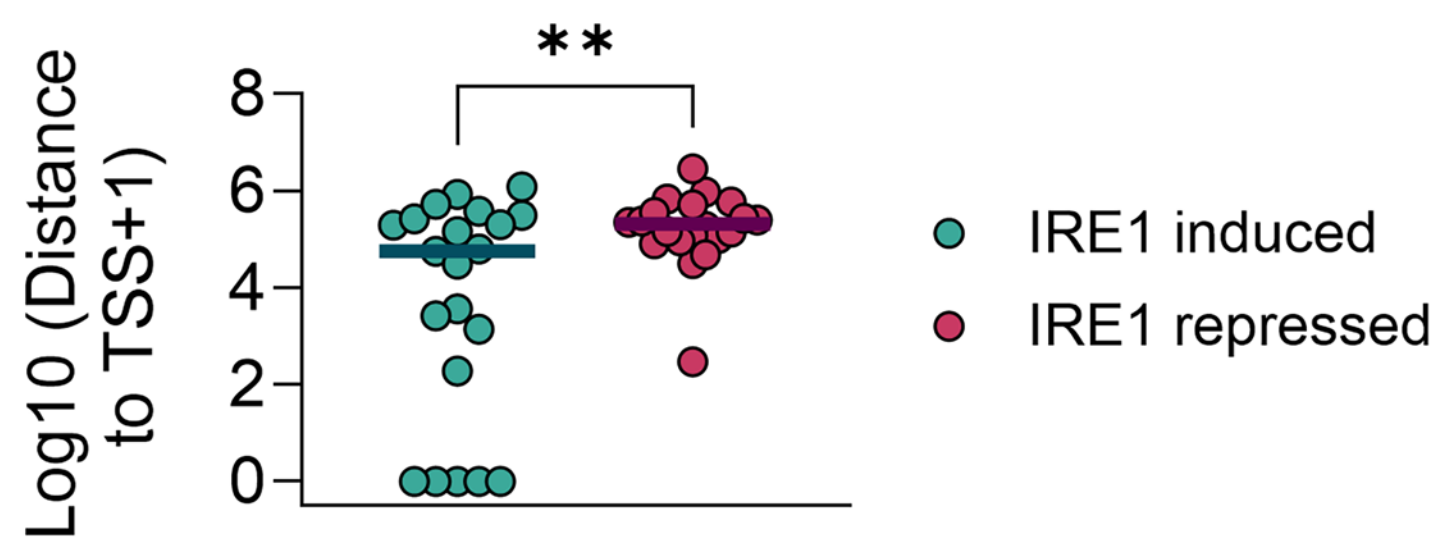


B.

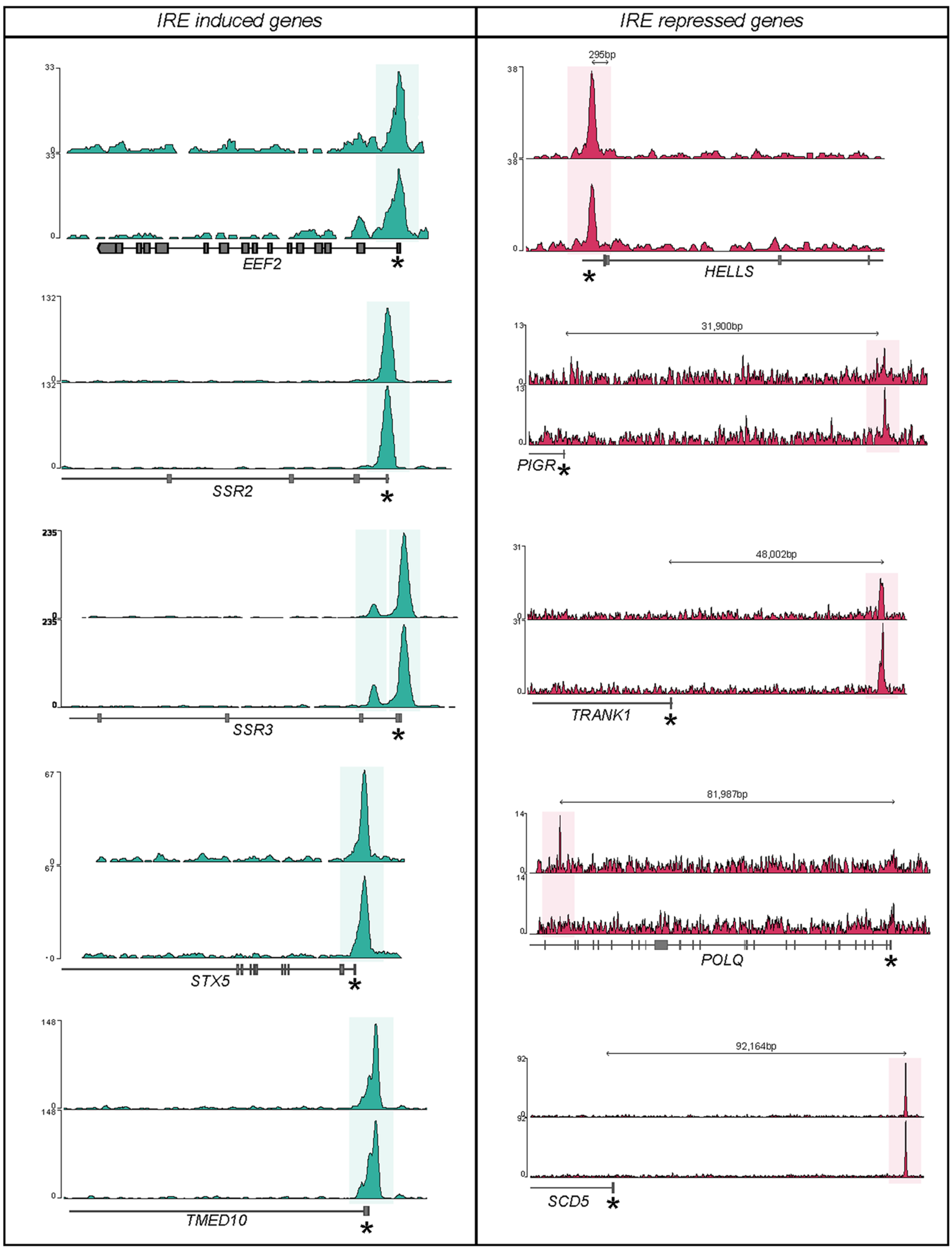


 © The Author(s)

**Figure EV7. Association of IRE1 activity signature genes with XBP1 transcription factor binding sites.**

(**A**) Log10 of the distance between the transcription start site (TSS) of each of the IRE1_41 genes and the nearest significant XBP1 peak. 43% of "IRE1 induced" genes have an XBP1 peak in their promoter. (**B**) Genomic tracks for the five closest IRE-induced and five closest IRE-repressed genes to an XBP1 peak. The distance to the closest IRE-regulated gene is indicated with an arrow (except when the gene is at the TSS, where distance = 0). A star marks the TSS of each IRE-regulated gene. Transparent blocks highlight XBP1-called peaks. Data information: Data were presented as mean ± SD. ns not significant, *$P \le 0.05$, **$P \le 0.01$, ***$P \le 0.001$, ****$P \le 0.0001$. Source data are available online for this figure.

© The Author(s) 

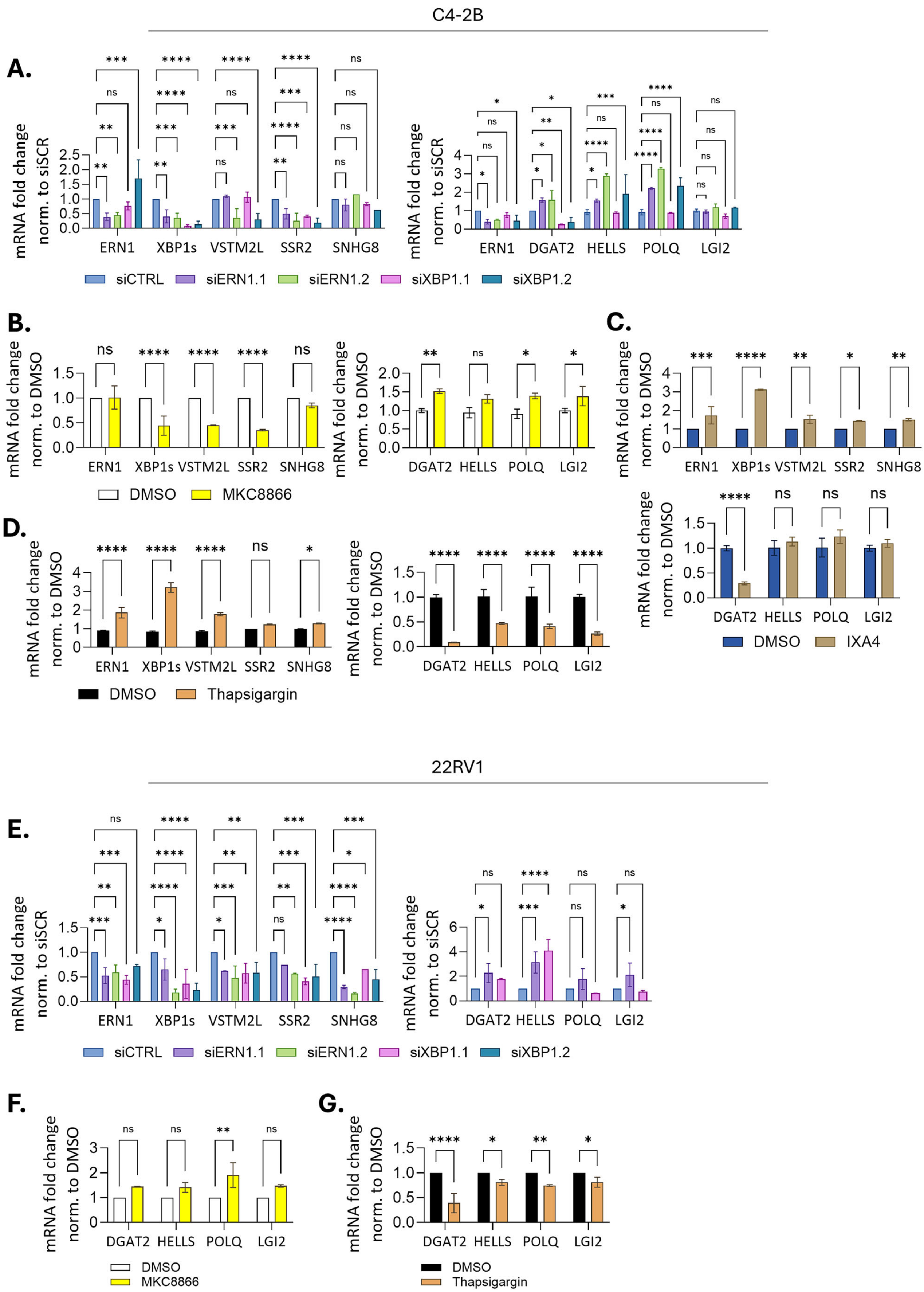
C4-2B
A.
mRNA fold change norm. to siSCR
ERN1 XBP1s VSTM2L SSR2 SNHG8
ERN1 DGAT2 HELLS POLQ LGI2
siCTRL siERN1.1 siERN1.2 siXBP1.1 siXBP1.2
B.
mRNA fold change norm. to DMSO
ERN1 XBP1s VSTM2L SSR2 SNHG8
DGAT2 HELLS POLQ LGI2
DMSO MKC8866
C.
mRNA fold change norm. to DMSO
ERN1 XBP1s VSTM2L SSR2 SNHG8
DGAT2 HELLS POLQ LGI2
DMSO IXA4
D.
mRNA fold change norm. to DMSO
ERN1 XBP1s VSTM2L SSR2 SNHG8
DGAT2 HELLS POLQ LGI2
DMSO Thapsigargin
22RV1
E.
mRNA fold change norm. to siSCR
ERN1 XBP1s VSTM2L SSR2 SNHG8
DGAT2 HELLS POLQ LGI2
siCTRL siERN1.1 siERN1.2 siXBP1.1 siXBP1.2
F.
mRNA fold change norm. to DMSO
DGAT2 HELLS POLQ LGI2
DMSO
MKC8866
G.
mRNA fold change norm. to DMSO
DGAT2 HELLS POLQ LGI2
DMSO
Thapsigargin


© The Author(s)

**Figure EV8. Changes in IRE1 activity signature mRNA upon pharmacological or genetic IRE1 modulation.**

mRNA fold change of ERN1, XBP1s, DGAT2, VSTM2L, SSR2, SNHG8, HELLS, LGI2, POLQ in C4-2B parental cells treated with (**A**) two siRNAs against ERN1 or XBP1 for 48 h normalised to scramble control, (**B**) 5 µM MKC8866 for 5 h normalised to DMSO, (**C**) 25 µM IXA4 for 5 h normalised to DMSO and (**D**) 100 nM Thapsigargin for 5 h normalised to DMSO ($n = 3$). mRNA fold change of ERN1, XBP1s, DGAT2, VSTM2L, SSR2, SNHG8, HELLS, LGI2, POLQ in 22RV1 parental cells treated with (**E**) two siRNAs against ERN1 or XBP1 for 48 h normalised to scramble control. mRNA fold change of DGAT2, HELLS, LGI2, POLQ in 22RV1 parental cells treated with (**F**) 5 µM MKC8866 for 5 h normalised to DMSO, (**G**) 100 nM Thapsigargin for 5 h normalised to DMSO ($n = 3$). Statistical analysis was performed using an ordinary two-way ANOVA and Sidak's multiple comparisons test with a single pooled variance. Data information: Data were presented as mean ± SD. ns not significant, *$P \leq 0.05$, **$P \leq 0.01$, ***$P \leq 0.001$, ****$P \leq 0.0001$. Source data are available online for this figure.

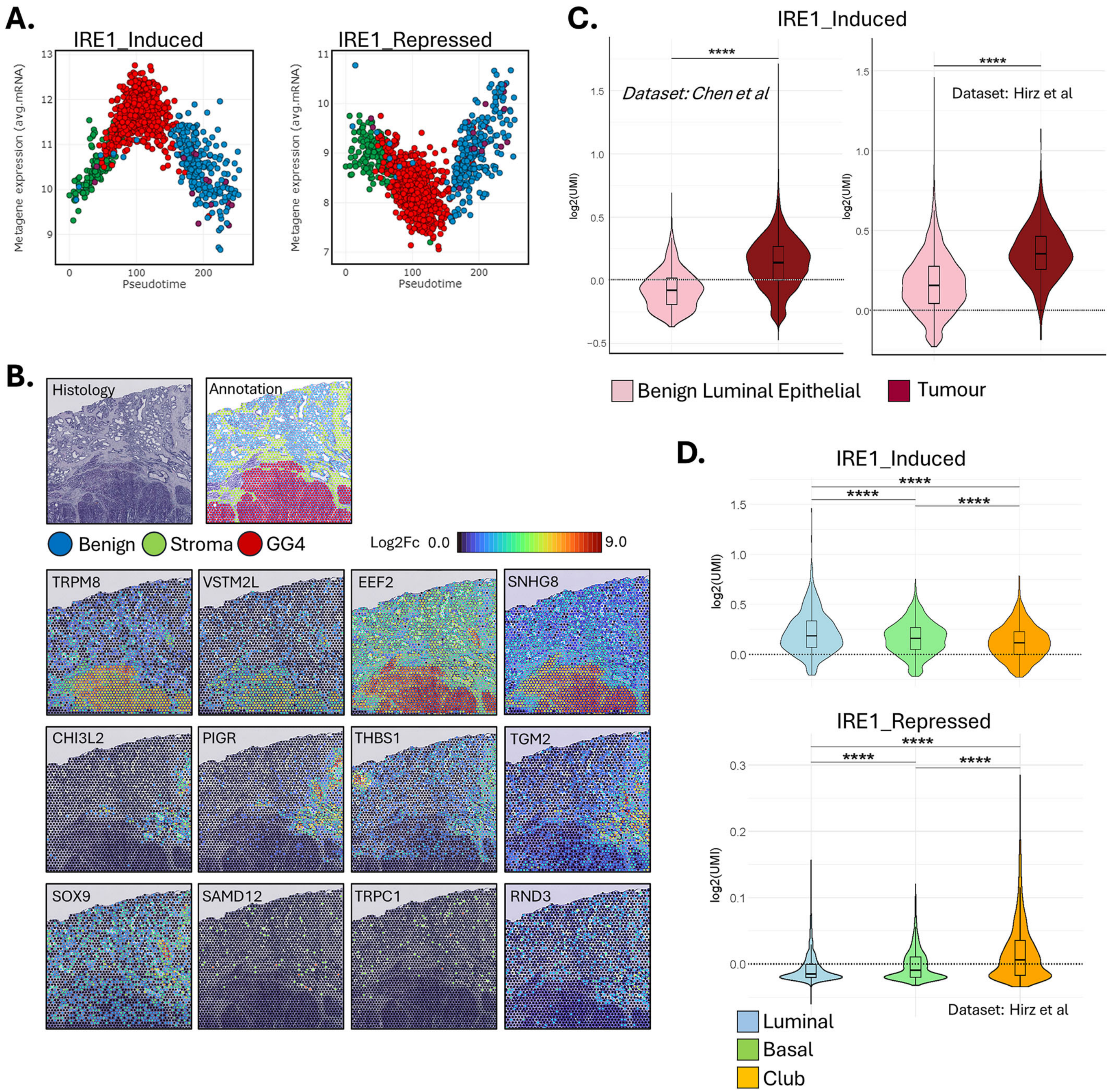


**Figure EV9. IRE1 activity signature signal distribution across cell types in single cell and spatial datasets.**

(**A**) Unbiased trajectory analysis by inferred pseudotime progression. Plot representing the correlation between mRNAs of IRE1_18 induced and repressed gene set and pseudotime inferred along the trajectory showing the change of these mRNAs across different clinical designations, including normal tissue (green), primary tumour (red), AR-positive CRPC (blue) and NEPC (purple). (**B**) Example distribution maps of genes within the IRE1_18 gene set representing GG4 tumour, generalised benign or specific benign histological annotations. This histology is referenced in Figs. 3I and 6B. (**C**) Violin plots showing the enrichment of the IRE1-induced part of the IRE1_18 signature in tumour cells compared to normal epithelial cells in two separate single-cell datasets. (**D**) Violin plots showing the enrichment of IRE1_18 induced or IRE1_18 repressed genes, in basal cells and club cells, compared to luminal epithelial cells in a single cell dataset.


© The Author(s)

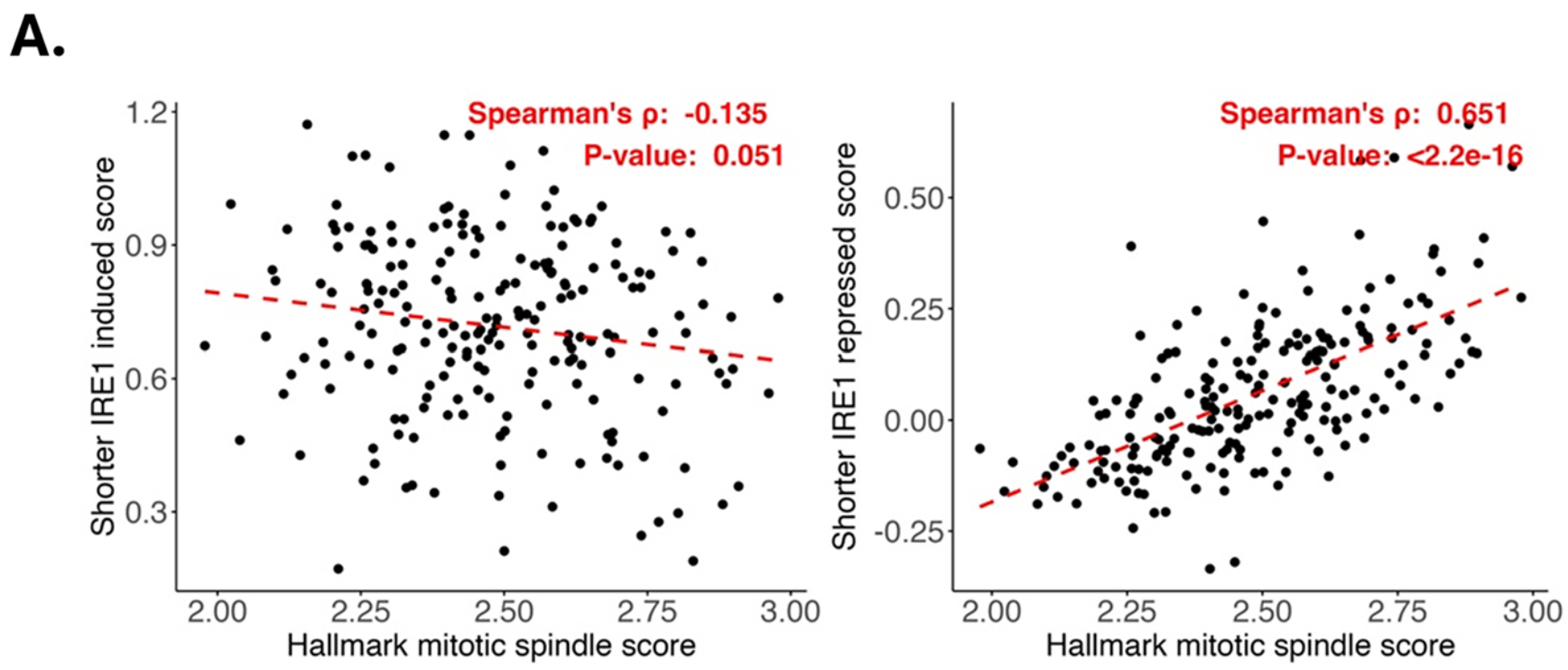


**Figure EV10. IRE1 activity signature induced and repressed score correlation with hallmark mitotic spindle.**

**(A)** IRE1sig1.0 induced score and IRE1sig1.0 repressed score correlation with Hallmark mitotic spindle score ($N = 210$). Source data are available online for this figure.

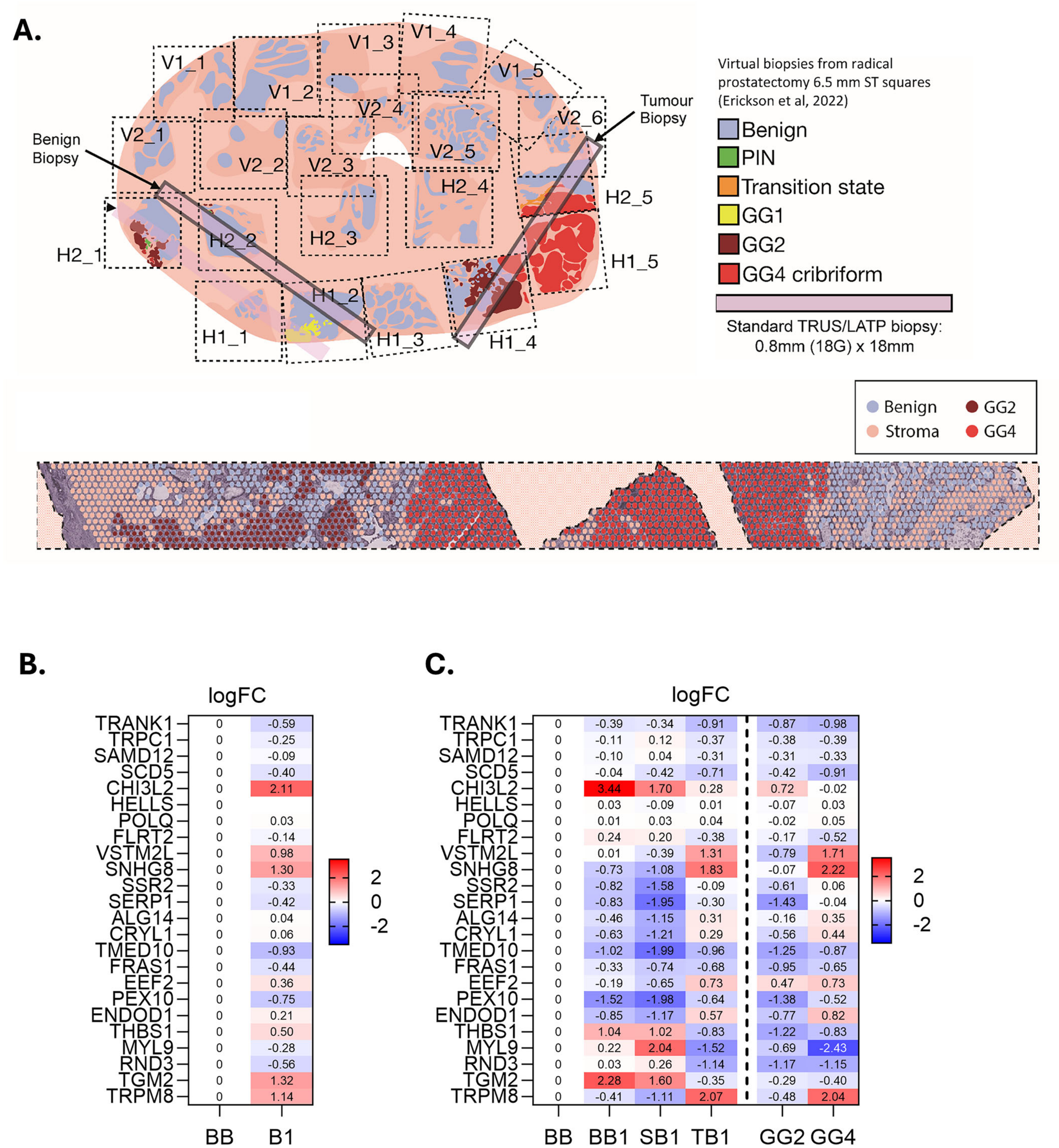


**Figure EV11. IRE1 activity signature signal distribution in virtual biopsies derived from spatial transcriptomic data.**

(**A**) Schematic representing the placement of a virtual biopsy containing GG2 and GG4 tumour or benign only tissue (black rectangles). (**B**) Pseudobulk analysis of tumour-bearing biopsy (B1) versus benign biopsy (BB). (**C**) Heatmaps showing changes in each sub-compartment within the virtual biopsy compared to the benign biopsy. These comparisons include benign tissue within the biopsy (BB1), stroma (SB1), tumour (TB1), as well as individual grade groups GG2 or GG4). Blue colour signifies downregulation, red colour signifies upregulation. The log2fc values for each gene are represented on the heatmap. Source data are available online for this figure.

 © The Author(s)

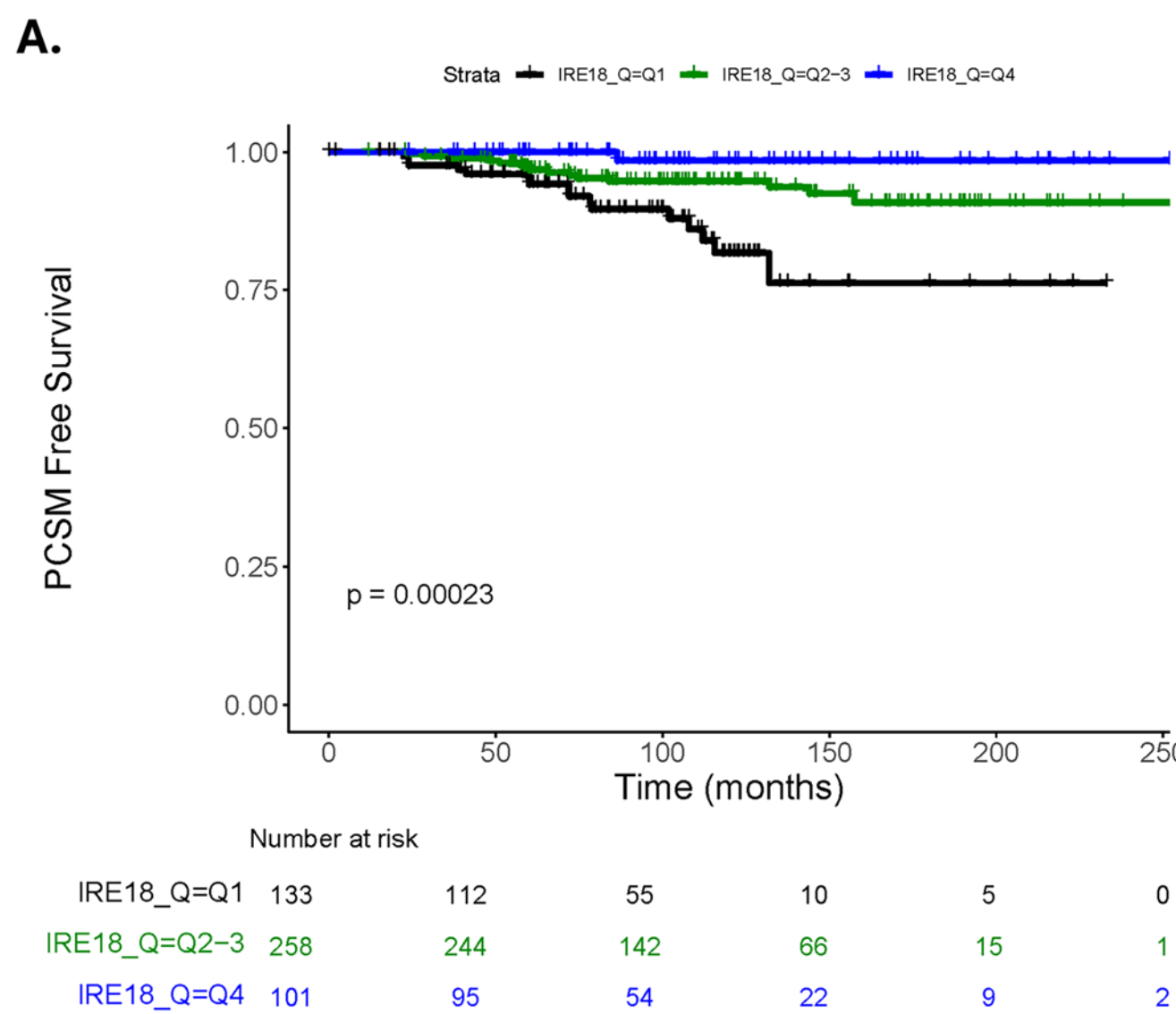


**Figure EV12. IRE1 activity signature prognosticates prostate cancer specific mortality.**

(**A**) Univariate and multivariate analysis of IRE1_18 expression quartiles in tumours from a retrospective cohort of 855 patients treated with RP with available long-term follow-up data (META855), with respect to prostate cancer-specific mortality. Kaplan–Meier curves of prostate cancer-specific mortality for each IRE1_18 quartile in the META855 cohort.

© The Author(s)
