# Supplementary material for: IRE1 activity is prognostic in localised and metastatic prostate cancer and affects epithelial lineage states
Source: EMBO Mol Med. 2026 Aug 22;18(10):3898–938. doi: 10.1038/s44321-026-00490-w (PMC13658804; doi:10.1038/s44321-026-00490-w)
Supplement: Supplementary file 2 [file 44321_2026_490_MOESM1_ESM.pdf]

IRE1 activity is prognostic in localised and metastatic prostate cancer and affects epithelial lineage states.

Dimitrios Doultsinos[1,✉], Ingrid Tomljanovic[2], Eleftherios Pilalis[3], Sophia M. Abusamra[1], Mohammed Alshalalfa[4], Romuald Parmentier[5], Vidhula Ahire[1], Imogen E. Bridges[1], Sandy Figiel[1], Joanna Hester[1], Damien Leach[6], Ashwin Nandakumar[1], Rensheng Wan[7], Yanis Zekri[10,11], Jichang Zhang[12], Charlotte L. Bevan[6], Edward O'Neill[1], Alastair D. Lamb[13], Valentine Macauley[1†], Jean Phillipe Theurillat[12], Alfonso Urbanucci[14,15], R. Jeffrey Karnes[16], Daniel E. Spratt[17], Elai Davicioni[4], David Quigley[7,8,9], Wilbert Zwart[10,11], Clementine Le Magnen[5], Aristotelis Chatziioannou[3,18], Ian G. Mills[1,✉]

Affiliations

[1] Nuffield Department of Surgical Sciences, University of Oxford, Oxford, UK;

[2]Department of Urology, Erasmus MC Cancer Institute, Erasmus University Medical Center, Wytemaweg 80, 3015 CN, Rotterdam, the Netherlands

[3]e-NIOS PC, Kallithea-Athens, Greece;

[4]Veracyte, South San Francisco, CA, USA

[5]Institute of Medical Genetics and Pathology, University Hospital Basel, Switzerland; Department of Urology, University Hospital Basel, Basel, Switzerland; Department of Biomedicine, University of Basel, University Hospital Basel, Basel, Switzerland

[6]Imperial College London, London, UK.

[7]Department of Urology, UCSF, San Francisco, CA, USA.

[8]Helen Diller Family Comprehensive Cancer Center, University of California, San Francisco.

[9]Department of Epidemiology and Biostatistics, UCSF, San Francisco, CA, USA.

[10]Division of Oncogenomics, The Netherlands Cancer Insitute, 1066 CX Amsterdam, The Netherlands

[11]Oncode Institute, The Netherlands

[12]Faculty of Biomedical Sciences, Institute of Oncology Research, USI, Bellinzona, TI, 6500, Switzerland.

[13]Centre for Cancer Evolution, Barts Cancer Institute, Queen Mary University of London, UK.

[14]Center for Cancer Eradication Research, Faculty of Medicine and Health Technology, Tampere University and Tays Cancer Center

[15] Department of Tumor Biology, Institute for Cancer Research, Oslo University Hospital, Norway.

[16]Mayo Clinic Dept of Urology, Rochester MN, USA

[17]University Hospitals Seidman Cancer Center, Case Western Reserve University, Cleveland, OH, USA

[18]Center of Systems Biology, Biomedical Research Foundation of the Academy of Athens, Athens, Greece.

†Professor Macauley is deceased.

✉ Corresponding authors

Corresponding author contact:

dimitrios.doultsinos@nds.ox.ac.uk

ian.mills@nds.ox.ac.uk

Appendix Table of contents

Appendix Figure S1 2
Appendix Figure S1 Legend 3
Appendix Figure S2 4
Appendix Figure S2 Legend 5
Appendix Figure S3 6
Appendix Figure S3 Legend 7
Appendix Figure S4 8
Appendix Figure S4 Legend 9
Appendix Figure S5 10
Appendix Figure S5 Legend 11
Appendix Figure S6 12
Appendix Figure S6 Legend 13
Appendix Figure S7 14
Appendix Figure S7 Legend 15

Appendix Figure S1

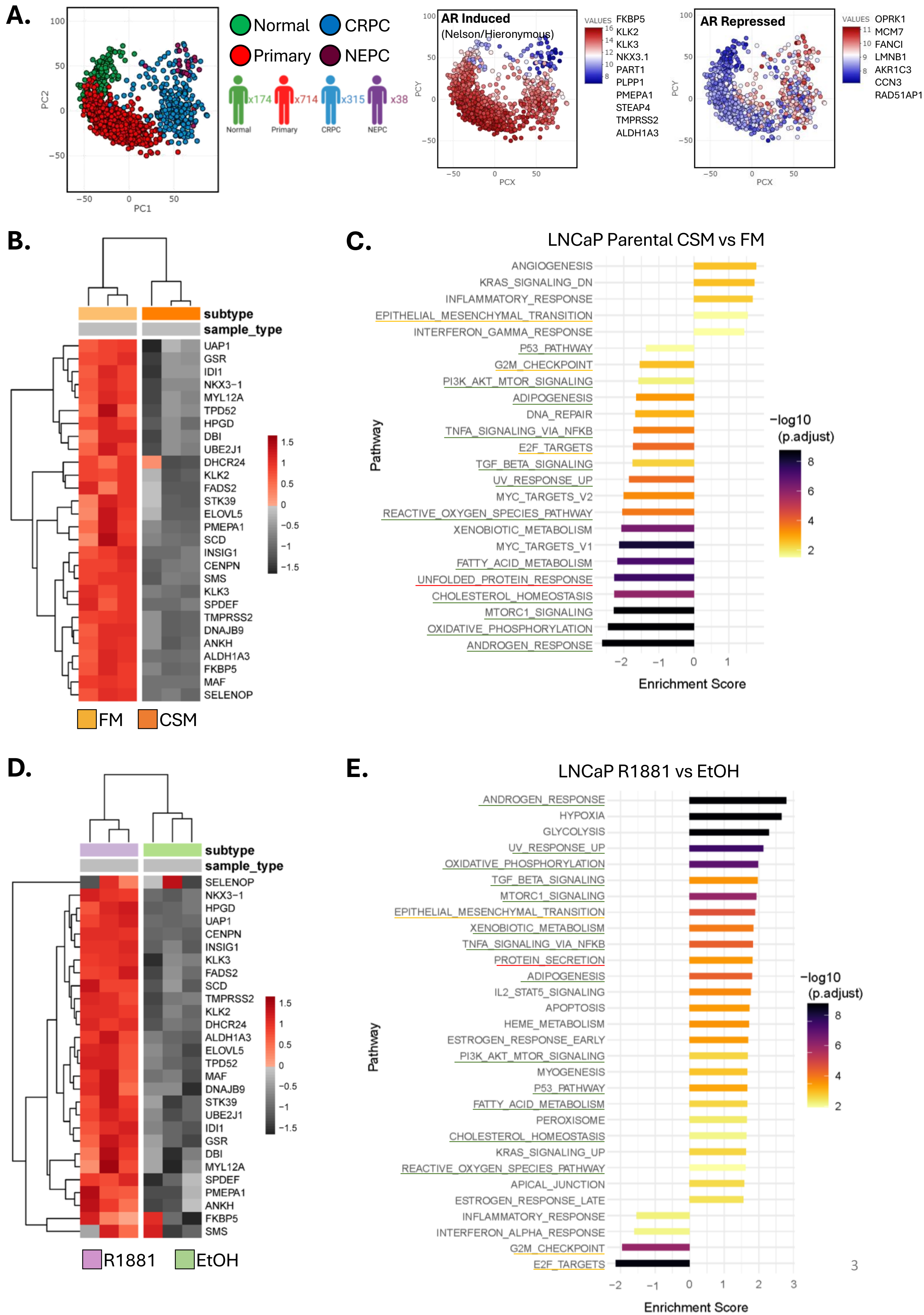

Appendix Figure S1. A) AR activity transcriptomic signatures in a comprehensive prostate cancer transcriptome atlas of integrated RNA-seq datasets from over 1000 clinical tissue samples ranging from benign (green) to NEPC (purple). Heatmaps show distribution of signal (low/blue; high/red) of these signatures across the dataset. B) Heatmap showing consensus AR signalling genes downregulated in the CSM (orange) condition compared to FM (yellow). C) Barplots displaying GSEA normalized enrichment scores (NES) for significantly enriched MSigDB Hallmark gene sets in: LNCaP cells grown in charcoal stripped media (CSM) for 72 hours compared to LNCaP cells grown in full media (FM) for 72 hours. D) Heatmap showing consensus AR signalling genes upregulated in the R1881 (purple) condition compared to EtOH (green). E) Barplots displaying GSEA normalized enrichment scores (NES) for significantly enriched MSigDB Hallmark gene sets in: LNCaP cells treated with R1881 for 12 hours compared to LNCaP cells treated with ethanol (EtOH) for 12 hours. Bar length indicates the normalized enrichment score and bar colour signifies the statistical significance. Hallmarks activated in C) but suppressed in E) are underlined in green. Hallmarks activated or suppressed in both C) and E) are underlined in orange. Hallmarks representing protein homeostasis are underlined in red. Each heatmap column represents a biological replicate.

Appendix Figure S2

A.

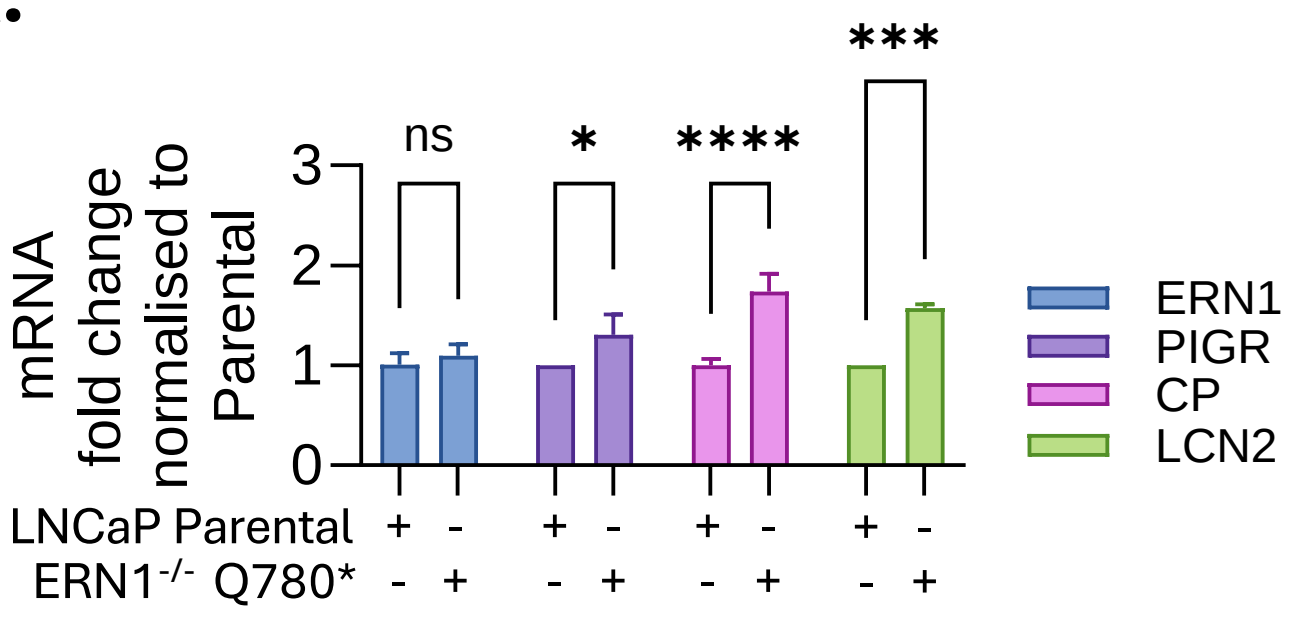

B.

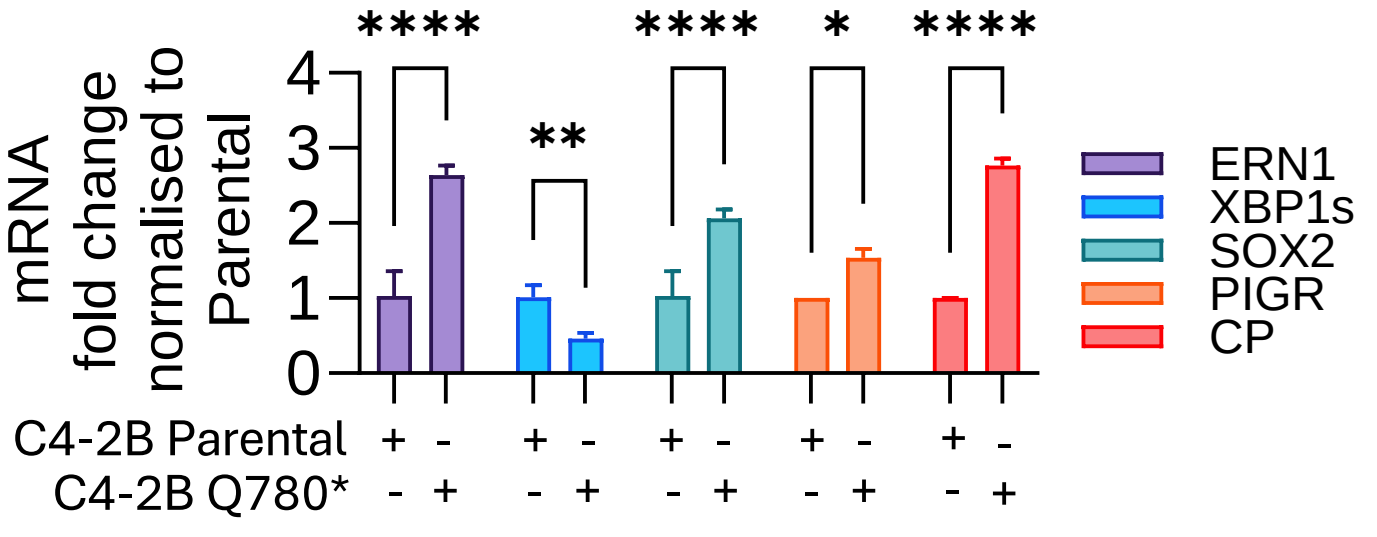

Appendix Figure S2. A) mRNA fold change of ERN1, CP, PIGR, LCN2 in LNCaP ERN1-/- Q780* cells (n=3) compared to LNCaP parental cells (n=3). B) A) mRNA fold change of ERN1, CP, PIGR, XBP1s, SOX2 in C42B Q780* cells (n=3) compared to C42B parental cells (n=3). Statistical analysis was performed using an ordinary two-way ANOVA and Sidak's multiple comparisons test with single pooled variance. Data information: Data are presented as mean ± SD. ns – not significant, *$P \leq 0.05$, **$P \leq 0.01$, ***$P \leq 0.001$, ****$P \leq 0.0001$.

Appendix Figure S3

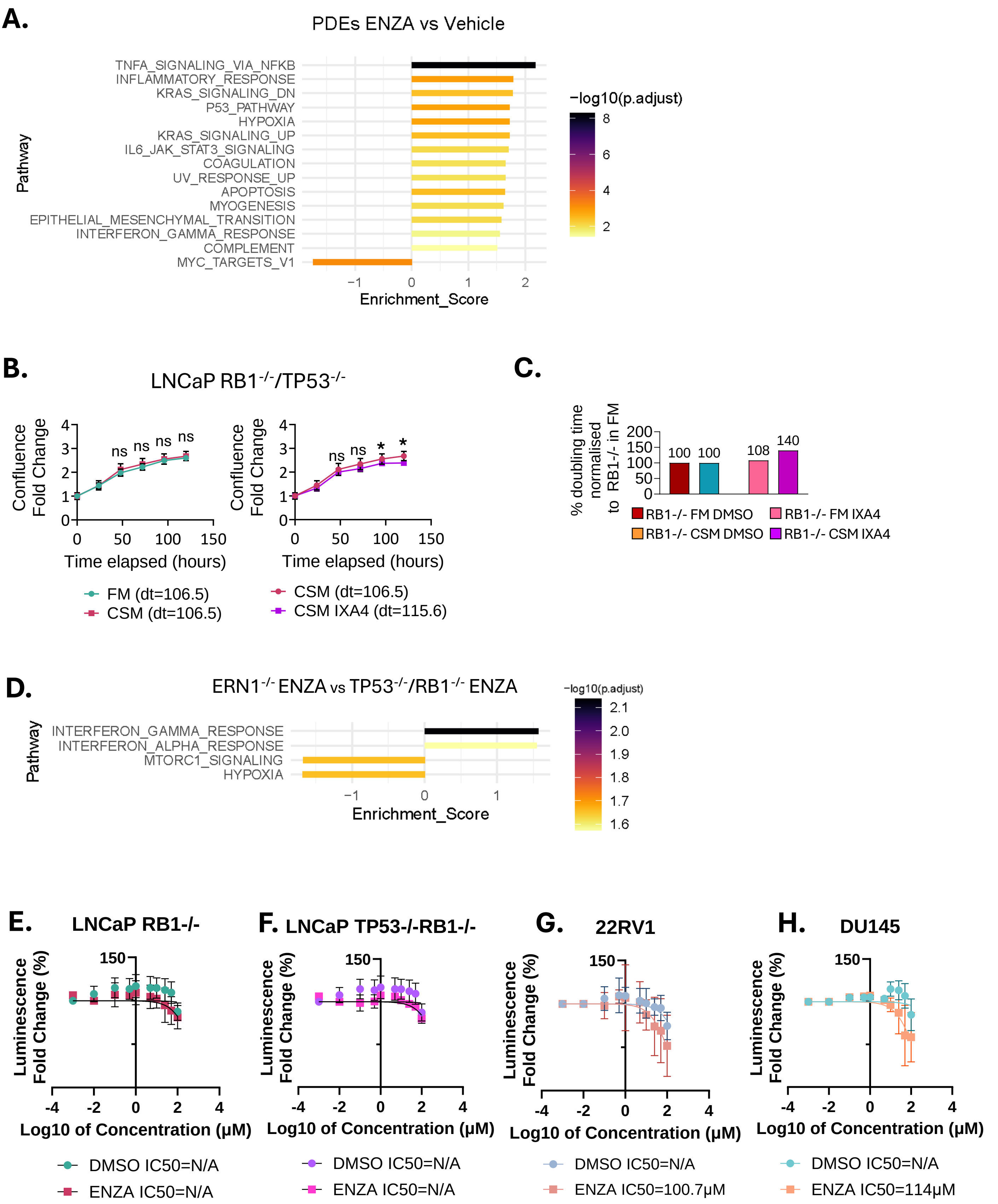

Appendix Figure S3. A) Significant Hallmarks upregulated in PDEs treated with ENZA compared to PDEs treated with vehicle control. Whole transcriptome RNAseq GSEA Hallmark analysis carried out with bar length signifying Hallmark enrichment score. Bar colour signifies log fold change of Hallmark enrichment compared to control. B) Growth curves of LNCaP RB1-/-TP53-/- cells (n=3) grown over 120 hours in FM (green), CSM (red) or in CSM in the presence of subtoxic (25µM) doses of IXA4 (purple). Confluence fold change was normalised to Incucyte readings taken at the 0-hour time point of each condition. T-tests were used for individual time point comparisons between FM and CSM; CSM plus DMSO and CSM plus IXA4. Exponential (Malthusian) growth curve equations were used to estimate the doubling time of each condition. C) Growth curves of LNCaP RB1-/- cells (n=3) grown over 120 hours in FM (green), CSM (red) or in CSM in the presence of subtoxic (25µM) doses of IXA4 (purple). Confluence fold change was normalised to Incucyte readings taken at the 0-hour time point of each condition. T-tests were used for individual time point comparisons between FM and CSM; CSM and CSM plus IXA4. Exponential (Malthusian) growth curve equations were used to estimate the doubling time of each condition. D) Significant Hallmarks upregulated in the RB1-/-TP53-/- cells treated with ENZA compared to ERN1-/- cells treated with ENZA. Whole transcriptome RNAseq GSEA Hallmark analysis carried out with bar length signifying Hallmark enrichment score. Bar colour signifies log fold change of Hallmark enrichment compared to control. E-H) ENZA concentration gradient measuring cytotoxicity through Cell Titer Glo assays in LNCaP RB1-/-, LNCAP LNCaP RB1-/-TP53-/-, 22RV1 and DU145 cells treated for 12 hours with increasing concentrations of ENZA (n=3). Logarithmic concentration vs normalised response curves were fit to calculate the IC50 of ENZA for each cell line. Data information: Data are presented as mean ± SD. ns – not significant, *P $\leq$ 0.05, **P $\leq$ 0.01, ***P $\leq$ 0.001, ****P $\leq$ 0.0001.

Appendix Figure S4

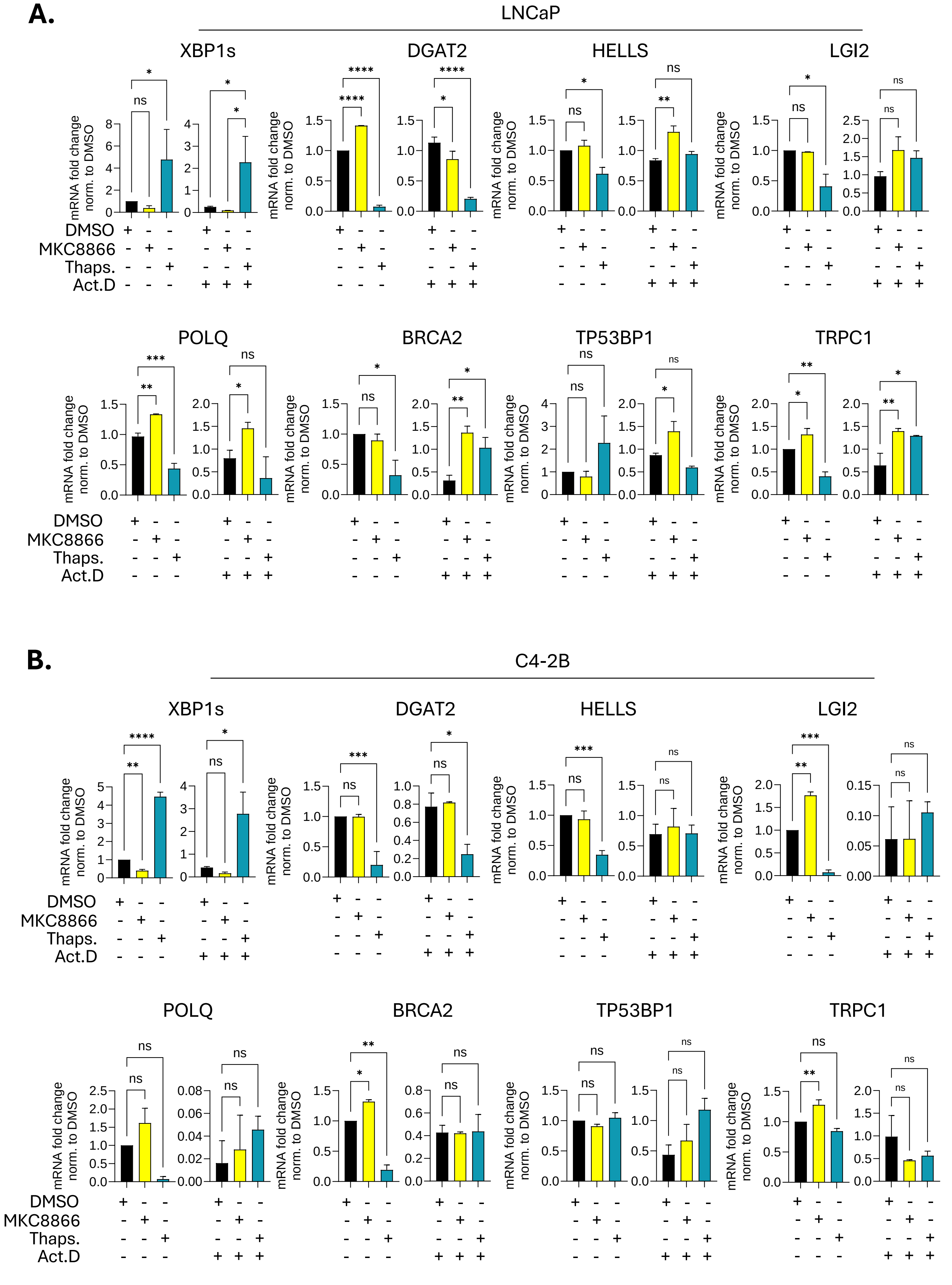

Appendix Figure S4. mRNA fold change of ERN1, XBP1s, DGAT2, HELLS, LGI2, POLQ, BRCA2, TP53BP1, TRPC1 in A) LNCaP parental cells (n=3) and B) C42B parental cells (n=3) pre-treated with 2µg/ml actinomycin D for two hours and then treated with 5µM MKC886 or 1nM thapsigargin for a further 5 hours. All values are normalised to parental DMSO only condition. Statistical analysis was performed using an ordinary two-way ANOVA and Sidak's multiple comparisons test with single pooled variance. Data information: Data are presented as mean ± SD. ns – not significant, *P ≤ 0.05, **P ≤ 0.01, ***P ≤ 0.001, ****P ≤ 0.0001.

Appendix Figure S5

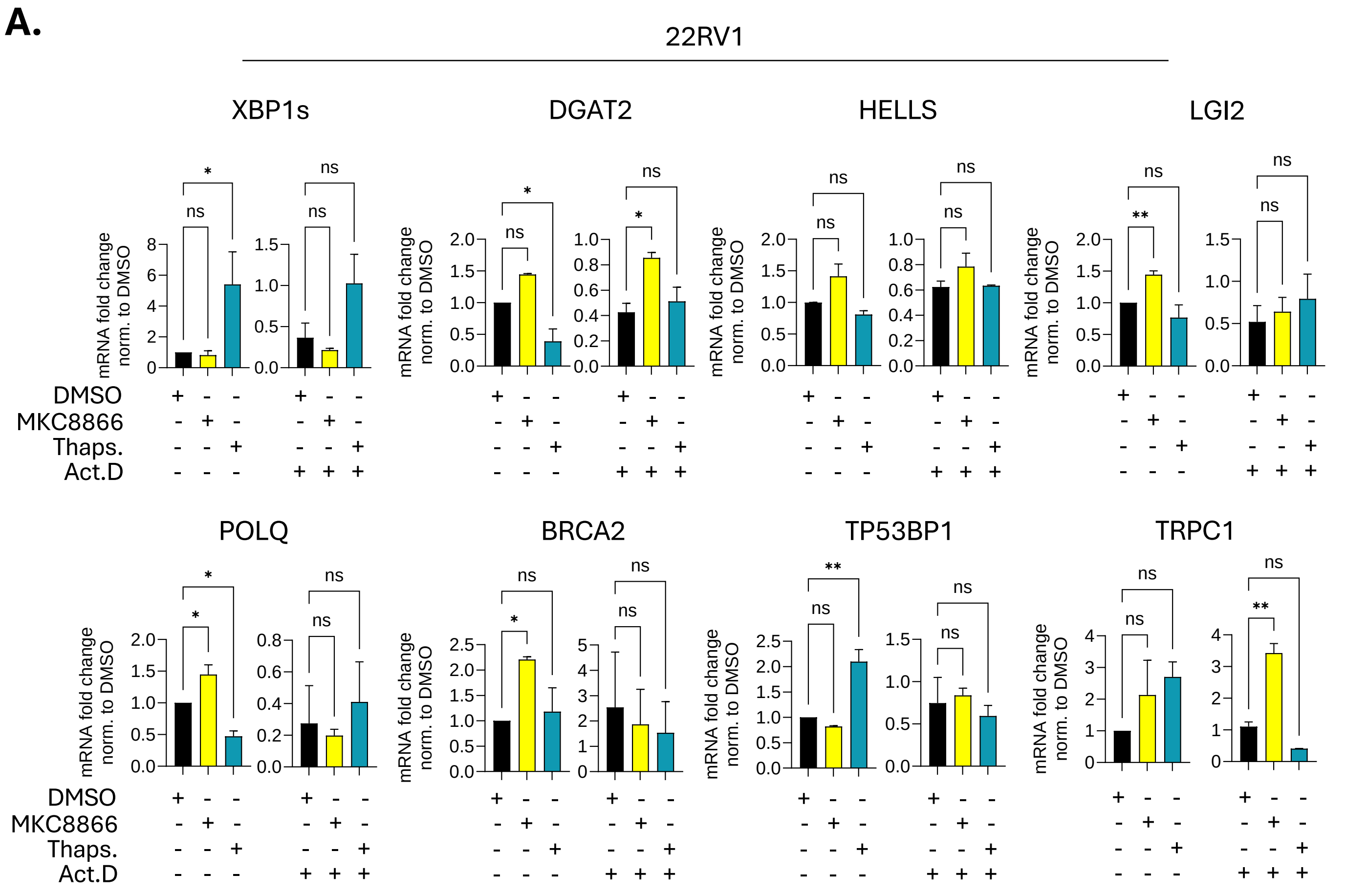

Appendix Figure S5. mRNA fold change of ERN1, XBP1s, DGAT2, HELLS, LGI2, POLQ, BRCA2, TP53BP1, TRPC1 in A) 22RV1 parental cells pre-treated with 2µg/ml actinomycin D for two hours and then treated with 5µM MKC886 or 1nM thapsigargin for a further 5 hours (n=3). All values are normalised to parental DMSO only condition. Statistical analysis was performed using an ordinary two-way ANOVA and Sidak's multiple comparisons test with single pooled variance. Data information: Data are presented as mean ± SD. ns – not significant, *P ≤ 0.05, **P ≤ 0.01, ***P ≤ 0.001, ****P ≤ 0.0001.

Appendix Figure S6

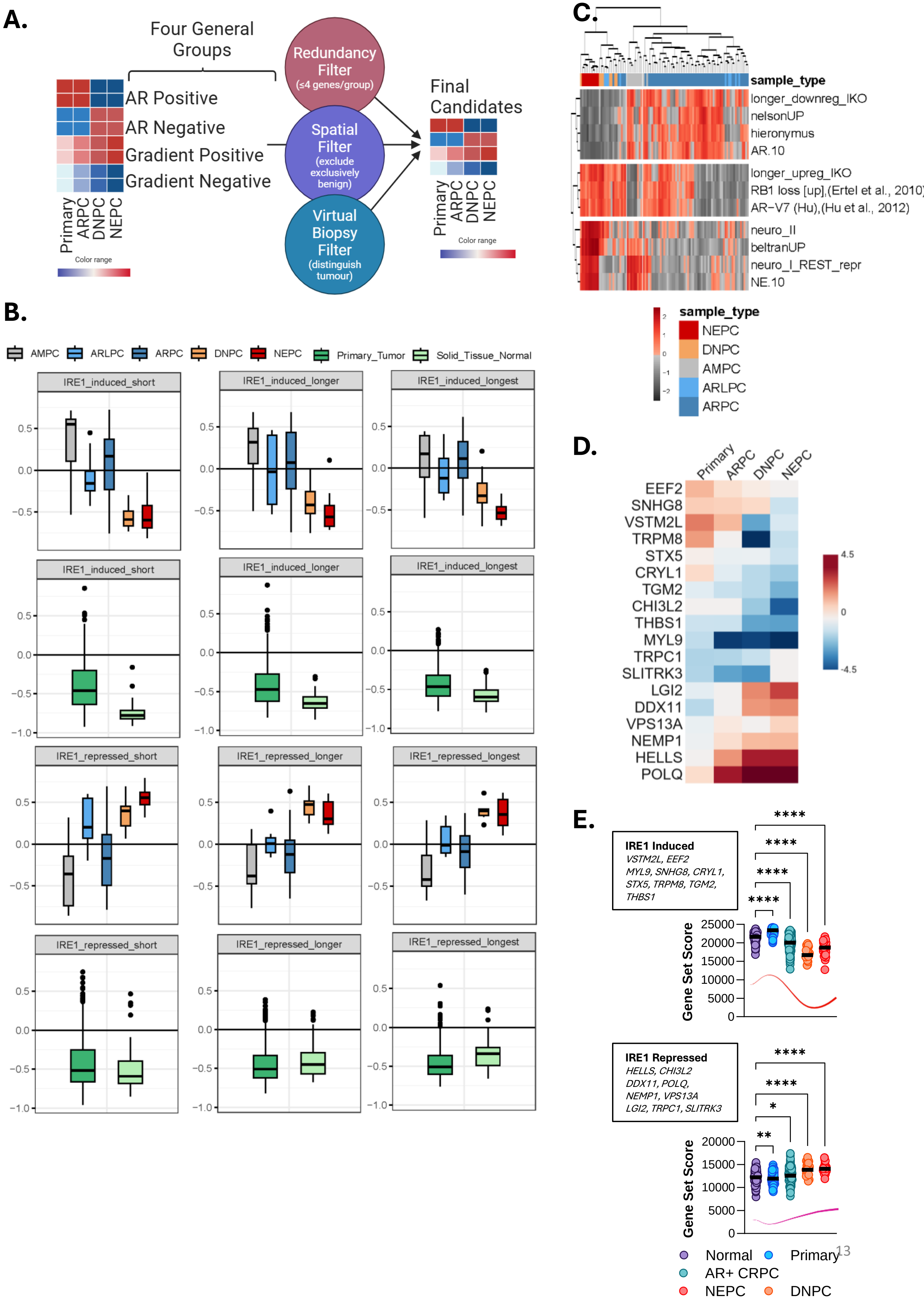

Appendix Figure S6. A) Schematic of filtering and reduction process to reduce IRE1 activity signature. Patterns identified in Figure 5F are filtered for redundancy, spatial distribution and bulk prognostication to produce a final list of candidates. B) Box plots of IRE1 activity induced and repressed gene sets, benchmarked in mCRPC (Labreque) and localised disease (TCGA) datasets. Patient populations represented include AR positive (dark blue), AR low (light blue), amphicrine (grey), NEPC (red), double negative (DNPC, orange), normal tissue (light green) and primary tumour (dark green). Using the filtering process described in figure S8A three different lengths of the IRE1 gene set are tested. C) Heatmap of GSVA (single-sample gene set) enrichment scores for selected gene signatures shown across samples from the Labrecque patient cohort (N=98) together with LNCaP parental and ERN1-/- cell line samples (N=5). Colour scale represents regulon enrichment GSVA scores (from lowest in greyscale to highest in red-scale). D) IRE1_18. 18 genes representing different biologies, cell types and clinical designations. E) Gene set enrichment scores of the IRE1 induced and IRE1 repressed parts of IRE1_18 in normal, primary, AR+ CRPC, DNPC and NEPC samples.

Appendix Figure S7

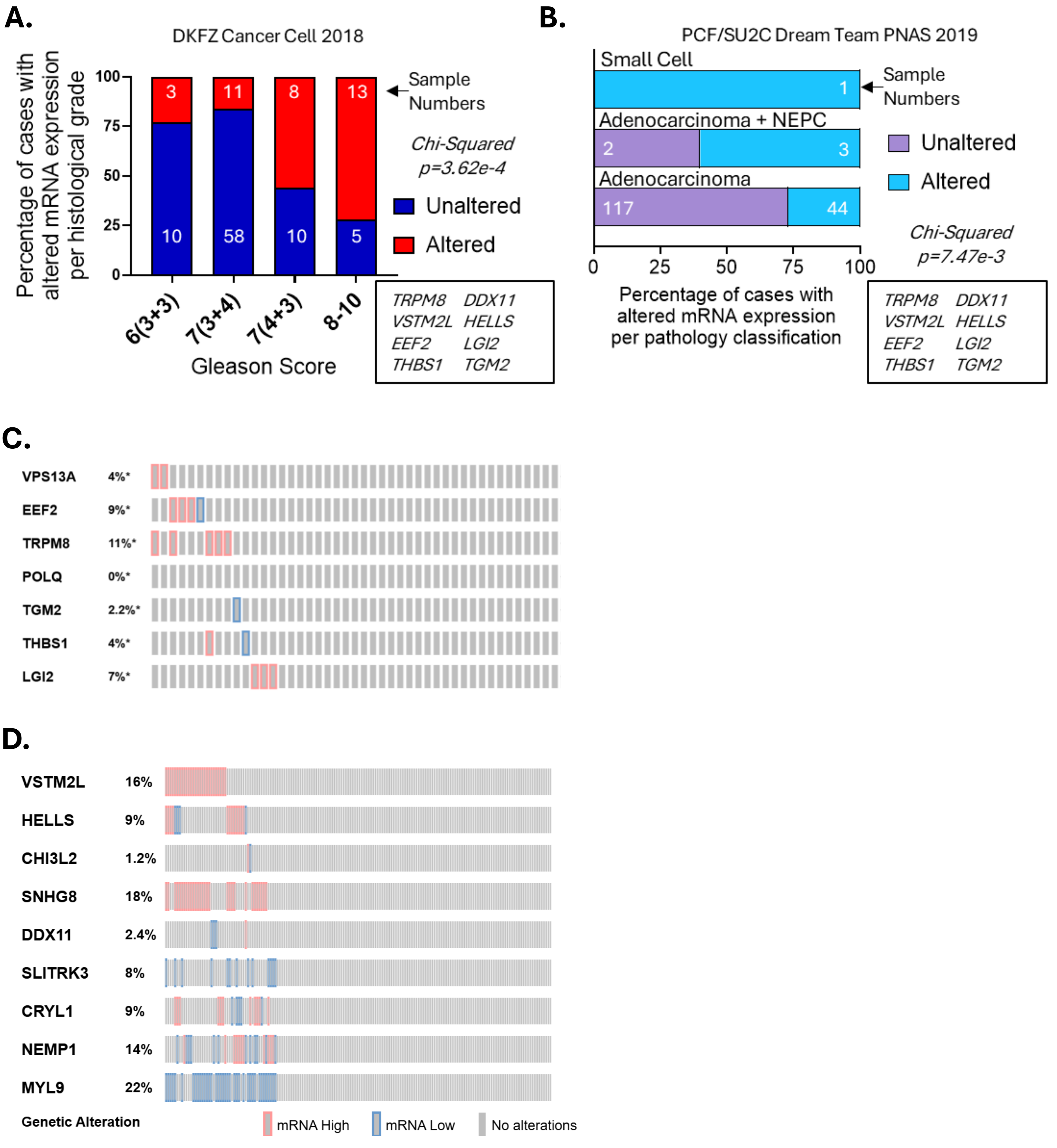

Appendix Figure S7. A) Correlation of expression pattern of the IRE1 activity gene set to radical prostatectomy Gleason score in the DKFZ Cancer Cell 2018 cohort. Statistical analysis was performed using Chi-squared test. Altered (red) status is defined as differentially significant z-score expression in tumour samples compared to the expression distribution of all log-transformed mRNA expression in adjacent normal samples in the cohort. B) Correlation of expression pattern of the IRE1 activity signature to pathology classification including adenocarcinoma (green), small cell (orange) and adenocarcinoma with NEPC features (purple). Statistical analysis was performed using Chi-squared test. Altered gene expression is defined as differentially significant log-transformed mRNA z-scores compared to mRNA signal from all samples in the cohort. Oncoprints of IRE1_18 activity genes mRNA in C) PCF/SU2C cohort compared to all samples and D) TCGA/PRAD cohort compared to normal samples.
