# Supplementary material for: IRE1 activity is prognostic in localised and metastatic prostate cancer and affects epithelial lineage states
Source: EMBO Mol Med. 2026 Aug 22;18(10):3898–938. doi: 10.1038/s44321-026-00490-w (PMC13658804; doi:10.1038/s44321-026-00490-w)
Supplement: Supplementary file 3 [file 44321_2026_490_MOESM2_ESM.pdf]

EMBO Molecular Medicine

IRE1 activity prognosticates prostate cancer and is a mediator of epithelial lineage control.

Dimitrios Doultsinos, Ingrid Tomljanovic, Eleftherios Pilalis, Sophia Abusamra, Mohammed Alshalalfa, Romuald Parmentier, Vidhula Ahire, Imogen Bridges, Sandy Figiel, Joanna Hester, Damien Leach, Ashwin Nandakumar, Rensheng Wan, Yanis Zekri, Jichang Zhang, Charlotte Bevan, Edward O'Neill, Alastair Lamb, Valentine Macauley, Jean-Philippe Theurillat, Alfonso Urbanucci, R Karnes, Daniel Spratt, Elai Davicioni, David Quigley, Wilbert Zwart, Clementine Le Magnen, Aristotelis Chatziioannou, and Ian Mills

Corresponding authors: Dimitrios Doultsinos (dimitrios.doultsinos@nds.ox.ac.uk) , Ian Mills (ian.mills@nds.ox.ac.uk)

Review Timeline:

Submission Date:	4th Jun 25
Editorial Decision:	1st Jul 25
Revision Received:	18th Dec 25
Editorial Decision:	22nd Jan 26
Revision Received:	17th May 26
Editorial Decision:	26th May 26
Revision Received:	17th Jun 26
Accepted:	6th Jul 26

Editor: Lise Roth

Transaction Report:

(Note: With the exception of the correction of typographical or spelling errors that could be a source of ambiguity, letters and reports are not edited. Depending on transfer agreements, referee reports obtained elsewhere may or may not be included in this compilation. Referee reports are anonymous unless the Referee chooses to sign their reports.)

1st Jul 2025

Dear Dr. Doultsinos,

Thank you for the submission of your manuscript to EMBO Molecular Medicine. We have now received feedback from the three reviewers who agreed to evaluate your manuscript.

As you will see from the enclosed reports, the reviewers agree that the findings are of potential interest, but also raise serious and partly overlapping concerns, related to the unclear choice and inconsistent cellular models, the lack of in vivo validation, the lack of demonstrated causality, and the insufficient validation of IRE1_18 as a prognostic biomarker.

Based on the nature of the concerns and the amount of time and work that would be needed to address them, considering also that at EMBO Press we encourage a single round of revisions in a limited time frame, we prefer to return the manuscript to you at this point with the decision that we cannot offer to publish it.

However, given the potential interest of the findings, we would welcome a new manuscript on the same topic if you obtained data that would strengthen the translational advance of your study.

To be completely clear, however, I would like to stress that if you were to send a new manuscript, this would be treated as a new submission rather than a revision and would be reviewed afresh, in particular with respect to the literature and the novelty of your findings at the time of resubmission. If you decide to follow this route, please make sure you nevertheless upload a letter of response to the referees' comments.

I am sorry that I could not bring better news this time and hope that the referee comments are helpful in your continued work in this area.

With kind regards,

Lise Roth

Lise Roth, Ph.D
Senior Editor
EMBO Molecular Medicine

***** Reviewer's comments *****

Referee #1 (Comments on Novelty/Model System for Author):

This is an interesting manuscript; however, a number of problems are apparent. ERN1 is most highly expressed in LNCaP cells, with much lower expression in AR negative and CRPC and NEPC cells. There is a huge discrepancy between what the cell line studies show and what the human tissue studies are showing. LNCaP cells are hormone sensitive whereas the CRPC and NEPC cells are not. If the authors are intending to show ERN1 effects on LNCaP cells, they should follow up with studies that include hormone sensitive tissues. If they are focused on CRPC and NEPC, they should use cell lines with those characteristics. If they are showing how ERN1 loss makes the cells less hormone sensitive, then they should use a model that follows that trend. Instead, they are using LNCaP cells as a cell line model and testing ideas in CRPC and NEPC tissues.

Referee #1 (Remarks for Author):

The overall goal of this manuscript is to characterize the impact of the UPR transducer IRE1 on AR-dependent biology and treatment resistance. Here, they develop a PCa-specific IRE1 activity gene set called IRE1_18 which includes both tumoral and micro-environmental niches. They use IRE1_18 to determine tumoral identity, androgen deprivation treatment suitability, prognosticate localized and metastatic disease independently from AR activity, and guide IRE1 modulation as a novel combination therapeutic.
This is an interesting manuscript; however, a number of problems are apparent. ERN1 is most highly expressed in LNCaP cells, with much lower expression in AR negative and CRPC and NEPC cells. There is a huge discrepancy between what the cell line studies show and what the human tissue studies are showing. LNCaP cells are hormone sensitive whereas the CRPC and

NEPC cells are not. If the authors are intending to show ERN1 effects on LNCaP cells, they should follow up with studies that include hormone sensitive tissues. If they are focused on CRPC and NEPC, they should use cell lines with those characteristics. If they are showing how ERN1 loss makes the cells less hormone sensitive, then they should use a model that follows that trend. Instead, they are using LNCaP cells as a cell line model and testing ideas in CRPC and NEPC tissues. Figure S7 comes close to this but the figures in the main text are lacking. Other figures that explore existing datasets do not test the expression of the IRE1_18 set in one single analysis.

There are other details that need to be amended -

1. Figure 1: All analyses are conducted only in LNCaP cells that are hormone sensitive PCa (HSPC). Should be repeated in at least one more hormone sensitive cell line.
2. Figure 1F,G - not clear why authors used CRPC/NEPC lines since initial studies were conducted in LNCaP cells. True comparison is between HSPC vs CRPC not CRPC vs NEPC.
3. The authors used LNCaP cells as an AR positive cell. There are many other AR positive cells that are castration resistant. Would IRE1 behave the same way in AR-positive CRPC cells?
4. Materials and Methods: Explain how tumoral, luminal, basal and club cells were distinguished from one another.
5. In the Introduction, please explain in detail the structure of IRE1 and how the Q780 mutation affects its structure and function.
6. Line 71: Please elaborate on the functions of club cells (Clara cells) in the prostate. These are commonly found in lung cells, but also seen in the prostate, especially in PCa but are mostly present in the prostatic urethra and collecting ducts, and are associated with inflammation (line 243). Authors have not explained their role in IRE1-related disease.
7. Figure 2a: include p-values.
8. Line 196-198: describe which ones are significantly upregulated vs those that are downregulated.
9. Explain in the Discussion how MKC8866 inhibits IRE1 and how IXA4 activates it.

Referee #2 (Remarks for Author):

In this manuscript, Doultsinos and colleagues are examining the role of the unfolded protein response (UPR) sensor IRE1 in prostate cancer progression, lineage plasticity, and therapeutic response by integrating bulk, single-cell, and spatial transcriptomic data from various preclinical models, including cell lines, patient-derived explants (PDEs), and clinical tumor samples. Using this integrative approach the authors develop a prostate cancer-specific IRE1 activity gene signature (dubbed IRE1_18), which stratifies patients by tumor subtype, AR dependency, histological grade, and therapy response. Moreover, the authors demonstrate that loss of IRE1 leads to transcriptomic changes associated with lineage reprogramming, including the emergence of club cell-like features and increased expression of stemness-associated factors (such as SOX2, EZH2, and POU3F2). This suggests that IRE1 may play a critical role in maintaining luminal epithelial identity and that loss of IRE1 may create a permissive state for treatment-resistant phenotypes to emerge.

Although this study could be of translational potential, the study has several limitations that should be addressed to improve the manuscript:

• While the authors describe correlations between loss of IRE1 and lineage plasticity, the mechanistic explanation of this relationship remains illusive, in particular whether the effects are mediated through XBP1s, RIDD, or both. Further functional experiments, such as rescue experiments using specific mutants, XBP1 ChIP-seq, or combinatorial knockdown of IRE1 with lineage reprogramming factors, would strengthen the causal interpretation of the findings.
• The study is primarily based on correlation-based analyses, such as transcription factor regulon enrichment and gene expression clustering. While these are informative, they do not establish causality. It would be interesting to include mechanistic dissection of selected genes of the IRE1_18 signature to assess whether they are directly regulated by IRE1, if so by which of IRE1s dual functions, XBP1s or RIDD, and to elucidate their functional role in tumor progression. Moreoever, all mechanistic data come from in vitro or ex vivo models. Consider complement the data with in vivo models using IRE1 knockout or pharmacologic modulation to validate findings in more complex systems.
• The authors claim that IRE1-responsive genes are prostate cancer-specific (line 359), but the basis for this specificity is unclear. Was this tested directly? How does the gene signature perform in other cancers compared to normal tissue? Moreover, for the downstream targets included in the IRE1 signature (Figure 5), it is important to distinguish between genes regulated via XBP1s and those dependent on IRE1, but not XBP1s (i.e., potential RIDD targets, see also comment #1).
• The IRE1_18 signature lacks external validation in independent patient cohorts with prospective clinical outcomes. To strengthen its clinical relevance, validation in additional datasets is recommended. Furthermore, assessing its applicability in liquid biopsies would be of interest.
• The authors propose IRE1_18 as a prognostic biomarker. However, key metrics such as effect sizes, AUCs, and hazard ratios are not clearly reported. To better support this claim, it would be valuable to include multivariate survival analyses comparing IRE1_18 with established clinical predictors (e.g., PSA) and other genomic signatures. Inclusion of ROC curves, such as in Figure 6D, would help quantify its prognostic performance. For the analyses using the DKFZ and PCF/SU2C cohorts, presenting odds ratios or hazard ratios in a summary table would more effectively illustrate whether increasing Gleason scores are associated with changes in gene expression. Additionally, it should be clearly stated whether the observed changes represent upregulation or downregulation to enhance biological interpretability.
• The data presentation and writing, though comprehensive, are at times unnecessarily redundant. A more focused narrative, along with the streamlining of figures by moving less critical data to the supplementary materials would improve clarity and overall readability. Key terms such as club cells, RIDD, and enzalutamide should be clearly and precisely defined early in the

manuscript.
• Statistical methods are often not clearly specified. For example, in Line 391: "Recapitulating multicellularity, IRE1 activity could distinguish between SC luminal, basal and club cells," the statistical test used to assess this segregation is not reported. Similarly, Figure 5I lacks both sample size information and details on the statistical test used to compare the two groups. Including effect size metrics, such as Cohen's d, would strengthen the analysis, assuming the data meet normality assumptions. Additionally, Figures 6A, 6B, and 6C require clearer explanation. Although the results section (lines 471-474) refers to correlations with IRE1_18 expression, the figures display correlations with individual genes rather than with the composite IRE1_18 signature, which should be addressed for consistency and clarity.

Referee #3 (Comments on Novelty/Model System for Author):

Most of the data were based on a single cell line (LNCaP), and no animal data support the main conclusion. The regulation of IRE1 activity/UPR by androgen signaling and the role of UPR in prostate cancer progression has been reported previously.

Referee #3 (Remarks for Author):

This manuscript uses LNCaP-based cell models to investigate how IRE1 signaling regulates gene expression and cellular responses to androgen deprivation. They further derived a IRE1 activity gene set and claimed this gene set could stratify prostate cancers at different stages. The authors performed excellent transcriptomic analysis and bioinformatical analysis, however, "wet" experimental evidence is often limited, lacks rigor, and not very convincing. The potential application of the described IRE1 gene signature in clinical diagnosis and treatment is unknown.

1. The role of IRE1/UPR signaling in prostate cancer development and the regulation between IRE1 and AR have been reported previously. The data in Fig 1 are highly predictable. Although the authors stated in Introduction that the IRE1-RIDD branch of the IRE1 activity is largely ignored in literature, this manuscript unfortunately ignored this as well. Only limited experiments used a mutant Q780 cell line.

2. Most of the data are based on a single cell line model. The data need to be validated in other cell line models. It would also be interesting to investigate IRE1 activity in AR-negative cells, or castration-resistant cell models.

3. The authors may need to talk more about "club" cells, as these cells are quite "new" to the field.

4. Only signal knockout clones were used to derived all the experimental data including the gene signature. The authors did not consider potential clonal variation. The data are generally not rigorous enough to support the main conclusion. Similar issues with siRNA experiments - at least two siRNAs need to be tested.

5. Fig 3I, the ERN1 level in the RB-/- cells was reduced to almost zero (Fig 3C). If so, how can IXA4 increase the IRE1 activity in this cell line?

6. Fig 4A, the 3 ENZA response curves look similar. How did the authors derived different IC50 values. This is particularly problematic for ERN1-/-Q780 cells, as the calculated IC50 was 93.18 uM, but the highest concentration tested is 100 uM.

7. Fig 4G, does IXA4 alone affect cell growth?

8. Many IRE1 signature genes were only marginally altered by siERN1 and siXBP1 (e.g., VSTM2L, HELLS, Fig 5B). How did the authors define a threshold value for these genes in clinical samples?

As a service to authors, EMBO provides authors with the possibility to transfer a manuscript that one journal cannot offer to publish to another EMBO publication. The full manuscript and if applicable, reviewers reports are automatically sent to the receiving journal to allow for fast handling and a prompt decision on your manuscript. For more details of this service, and to transfer your manuscript to another EMBO title please click on Link Not Available

Please do not share this URL as it will give anyone who clicks it access to your account.

Comments to the reviewers

Reviewer 1

Summary comments:

"The overall goal of this manuscript is to characterize the impact of the UPR transducer IRE1 on AR-dependent biology and treatment resistance. Here, they develop a PCa-specific IRE1 activity gene set called IRE1_18 which includes both tumoral and micro-environmental niches. They use IRE1_18 to determine tumoral identity, androgen deprivation treatment suitability, prognosticate localized and metastatic disease independently from AR activity, and guide IRE1 modulation as a novel combination therapeutic.

This is an interesting manuscript; however, a number of problems are apparent. ERN1 is most highly expressed in LNCaP cells, with much lower expression in AR negative and CRPC and NEPC cells. There is a huge discrepancy between what the cell line studies show and what the human tissue studies are showing. LNCaP cells are hormone sensitive whereas the CRPC and NEPC cells are not. If the authors are intending to show ERN1 effects on LNCaP cells, they should follow up with studies that include hormone sensitive tissues. If they are focused on CRPC and NEPC, they should use cell lines with those characteristics. If they are showing how ERN1 loss makes the cells less hormone sensitive, then they should use a model that follows that trend. Instead, they are using LNCaP cells as a cell line model and testing ideas in CRPC and NEPC tissues. Figure S7 comes close to this but the figures in the main text are lacking. Other figures that explore existing datasets do not test the expression of the IRE1_18 set in one single analysis."

We thank the reviewer for their comments, the time commitment to reviewing our manuscript and their interest in it. We agree that a single cell line does not recapitulate the full disease spectrum and as such have generated data in C4-2B, 22RV1 and DU145 cells to complement our previous experiments in LNCaP and their derivates.

Lines used in original manuscript:	Additional lines in new version:
LNCaP	C4-2B
LNCaP ERN1-/-	22RV1
LNCaP ERN1-/- Q780*	DU145
LNCaP RB1-/-	C4-2B Q780*
LNCaP TP53-/-RB1-/-	BPH1
C4-2B	

Regarding analysis in externally generated and published datasets we have advanced our analysis of the Prostate Cancer Atlas which includes harmonised normal, primary, AR positive and AR negative disease as well as externally validate our IRE1 activity signature in both the GRID (55000 patients, Veracyte), West Coast Dream Team (200 patients, UCSF), META855 (855 patients, Wisconsin) and others. We hope that these extra steps address the majority of concerns raised and strengthen our manuscript.

Specific comments

- LNCaP cells do not recapitulate the full disease spectrum.

We agree that LNCaP cells do not recapitulate the full disease spectrum and as such have introduced new data on cell lines representing HSPC (LNCaP), AR+ CRPC (C4-2B), ARV7 CRPC (22RV1), AR-CRPC (DU145) and benign prostatic hyperplasia (BPH1) for this purpose. We attempted to describe

IRE1 activity's involvement across dynamic disease progression states rather than focusing on HSPC or NEPC exclusively. As such we used the curated library of the Prostate Cancer Atlas comprising normal, primary (HSPC), AR+ CRPC and AR- CRPC (NEPC) samples as well. Consequently, we show the change of IRE1 activity (as defined by mRNA expression of consensus XBP1 and RIDD targets in both clinical datasets and cell lines as seen in new figures 1A and 1B:

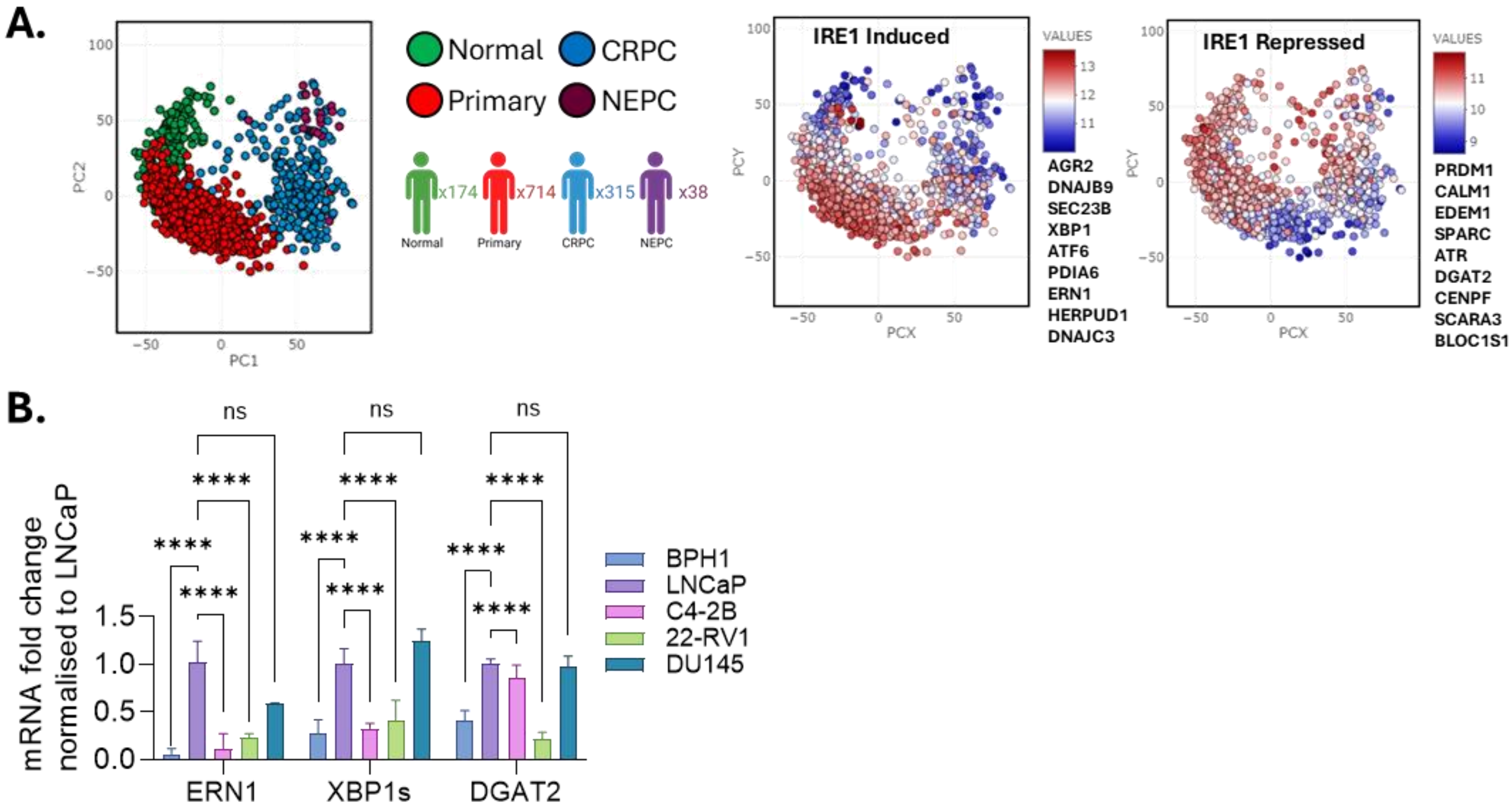

To further account for the phenotypic shift caused by IRE1 loss we introduced whole transcriptomes of LNCaP, LNCaP ERN1$^{-/-}$, 22RV1 and DU145 cells into the Prostate Cancer Atlas showcasing a shift on the AR dependency axis caused by IRE1 loss (Figure 1C):

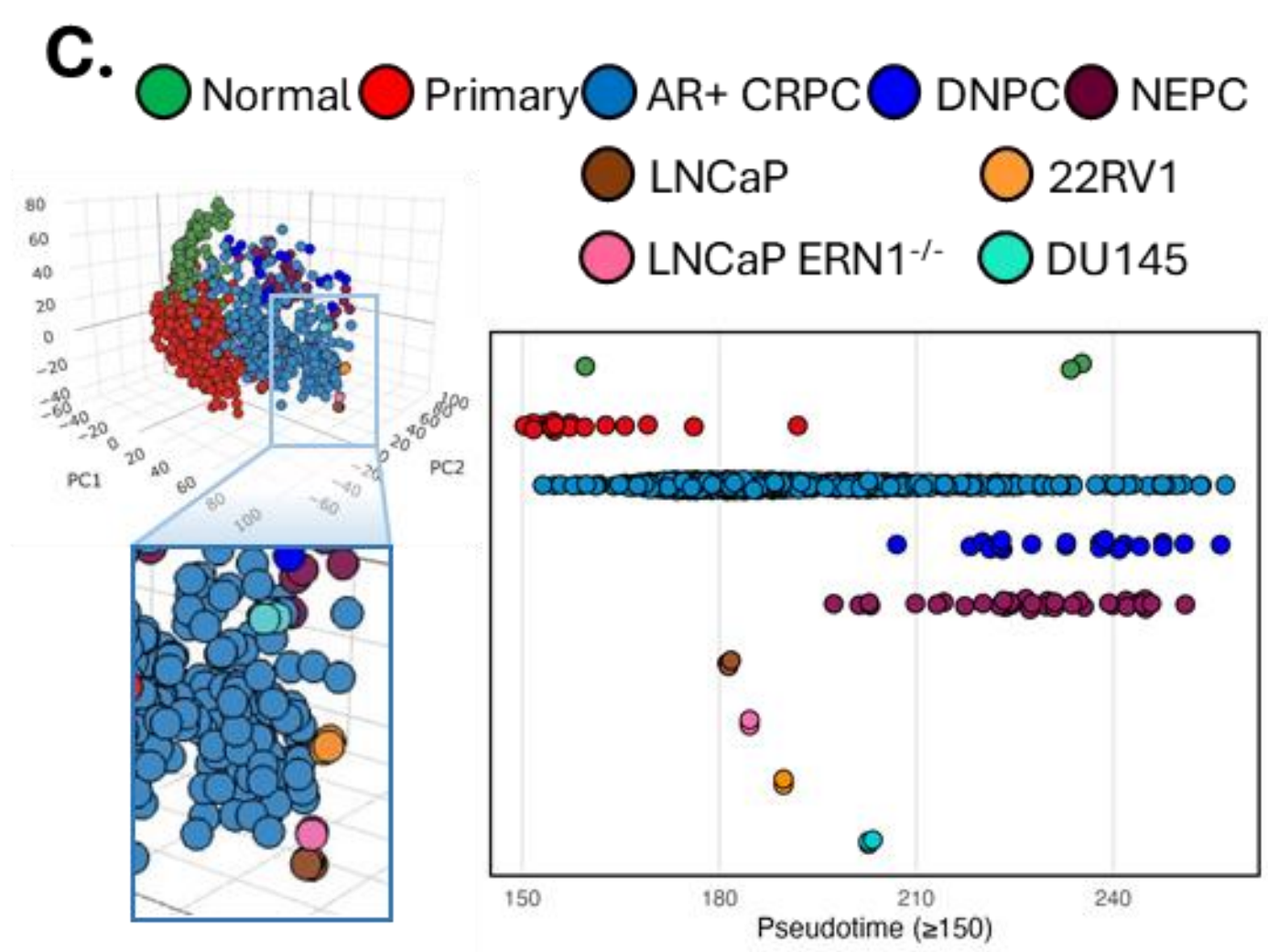

We then document AR activity as defined by AR and AR target mRNA levels in LNCaP cells deficient for IRE1 activity to showcase that neither in LNCaP ERN1$^{-/-}$ nor in LNCaP ERN1$^{-/-}$ Q780* cells do we see a parallel downregulation of AR activity in response to IRE1 loss (Figures 1D):

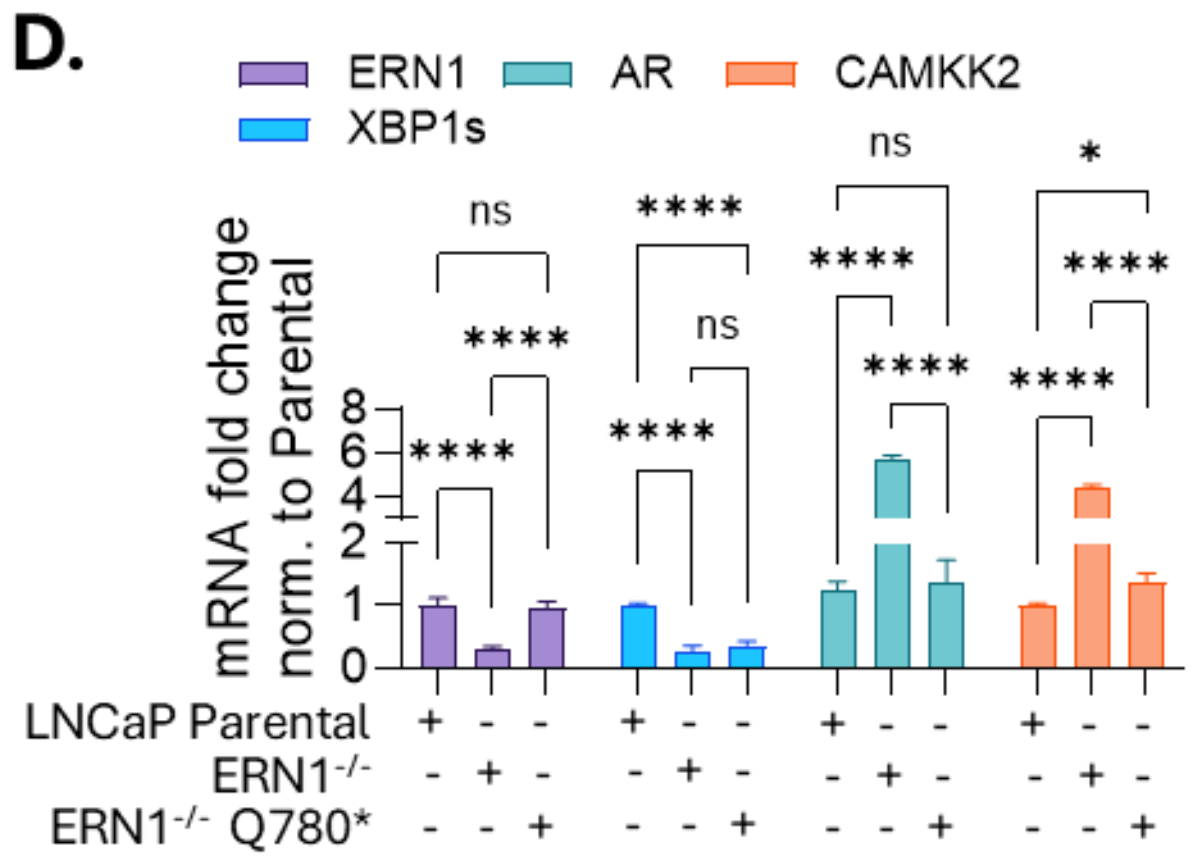

- Figure 1: All analyses are conducted only in LNCaP cells that are hormone sensitive PCa (HSPC). Should be repeated in at least one more hormone sensitive cell line.
  The focus of the manuscript is on how IRE1 activity tracks with disease progression and not on solely HSPC to CRPC transition nor HSPC or CRPC centric. Hence, we expanded our models to include CRPC lines to more closely resemble a broad range of clinical presentations in PCa as well as introduced further analyses in clinical datasets of varying disease progression.

Additional lines in new version:

AR+ CRPC: C4-2B
AR+ CRPC plus ARV7 variant: 22RV1
AR- CRPC: DU145
AR+ CRPC plus IRE1 modulation: C4-2B Q780*

- Figure 1F, G - not clear why authors used CRPC/NEPC lines lines? These are patients since initial studies were conducted in LNCaP cells. True comparison is between HSPC vs CRPC not CRPC vs NEPC.
  The data shown in original 1F, G are publicly available patient data. As we wanted to track IRE1 activity in these clinical designations we used these datasets and additional CRPC cell lines. The utility of this patient dataset is to signify the lineage shift from an AR positive adenocarcinoma to an AR negative neuroendocrine carcinoma. We have already seen in figure 1A (new) that IRE1 activity steadily decreases from HSPC to AR+ CRPC to AR- CRPC. We have also showcased both in the Prostate Cancer Atlas and in the TCGA that IRE1 activity increases in primary disease before decreasing in CRPC presentations (figure 1A, 5G, 5H).

Figure 1

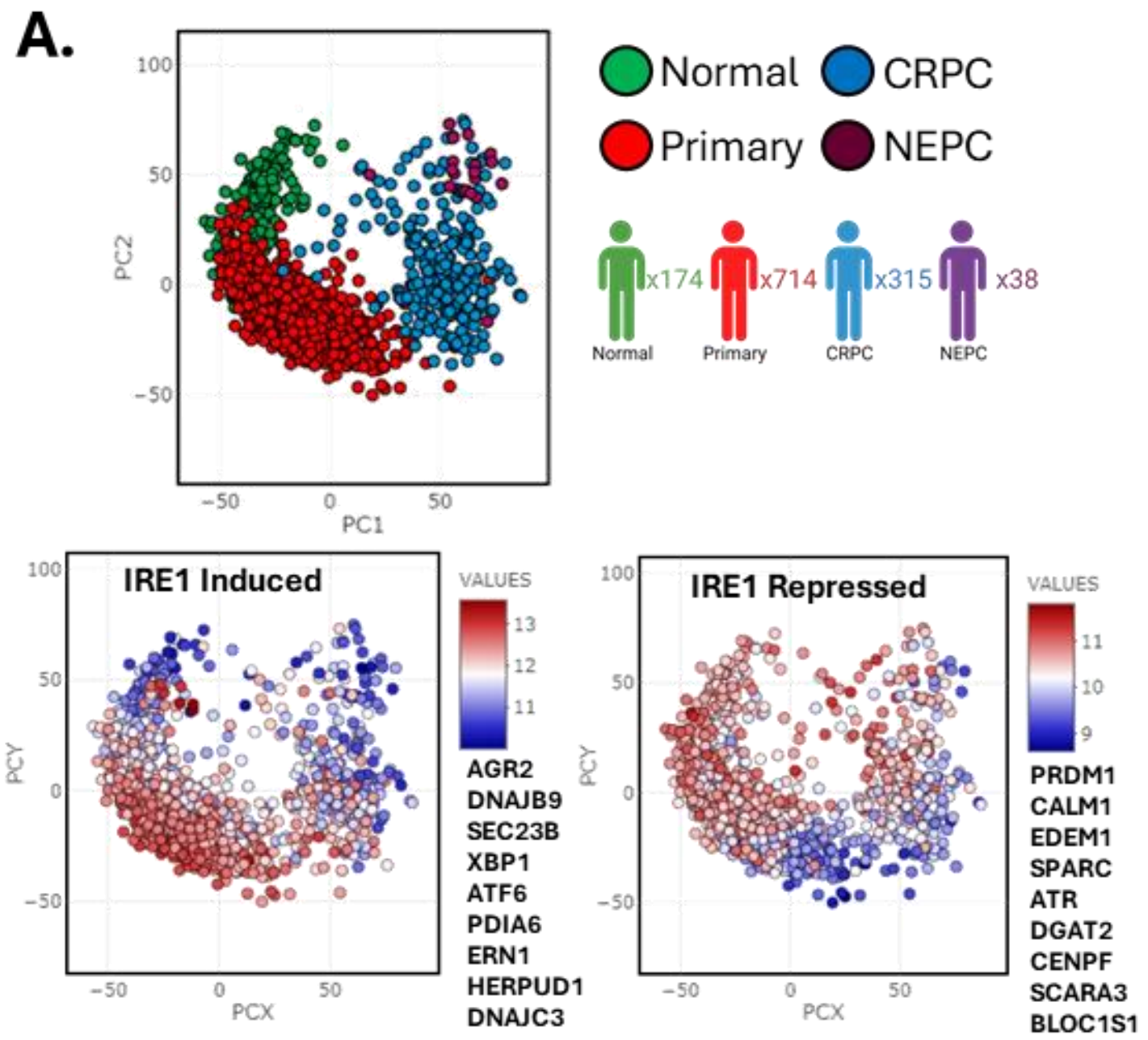

Figure 5

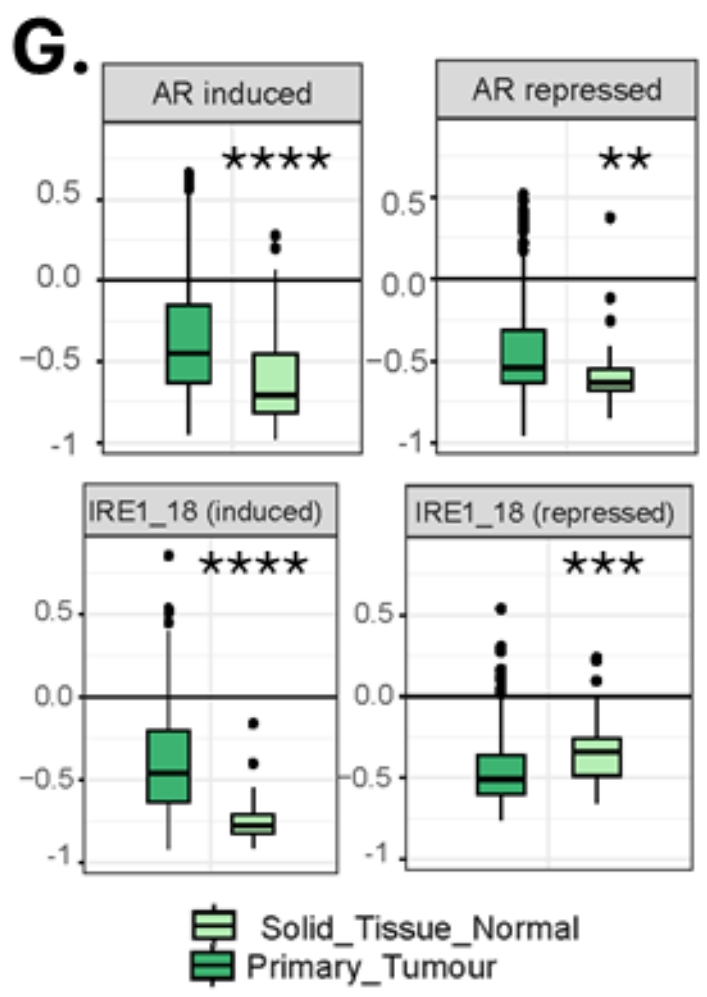

As such, this analysis serves to link the similarities seen in the regulons affected by IRE1 loss in our pre-clinical models. Having observed that these deregulated transcription factors also include lineage reprogramming markers (Figure 2A)

Figure 2

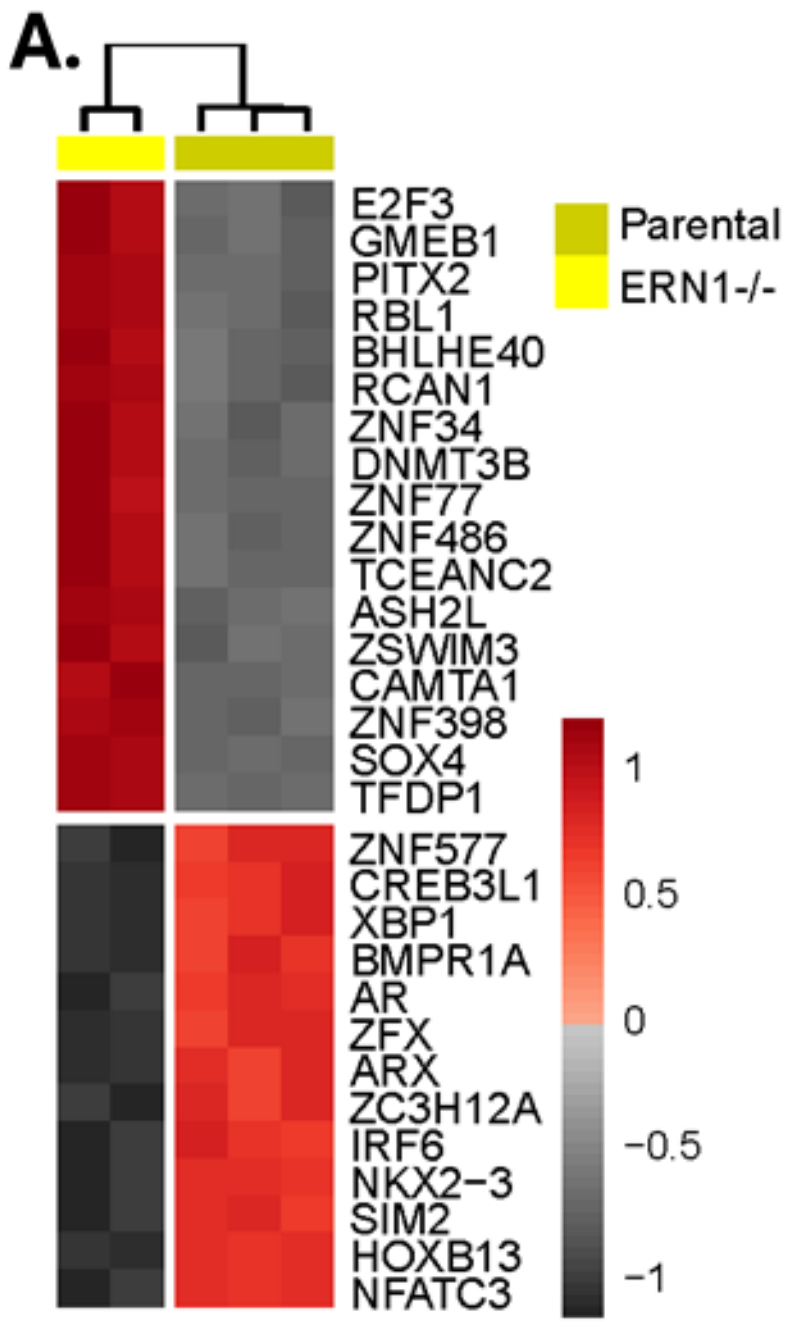

we wanted to make a comparison between a clinical dataset characterised by lineage plasticity (NEPC) and equivalent AR positive control (AR+ CRPC). This analysis serves the purpose to correlate downregulation of IRE1 activity (low XBP1) with upregulation of reprogramming markers such as SOX2 and POU3F2 (Figure 3B-C).

Figure 3

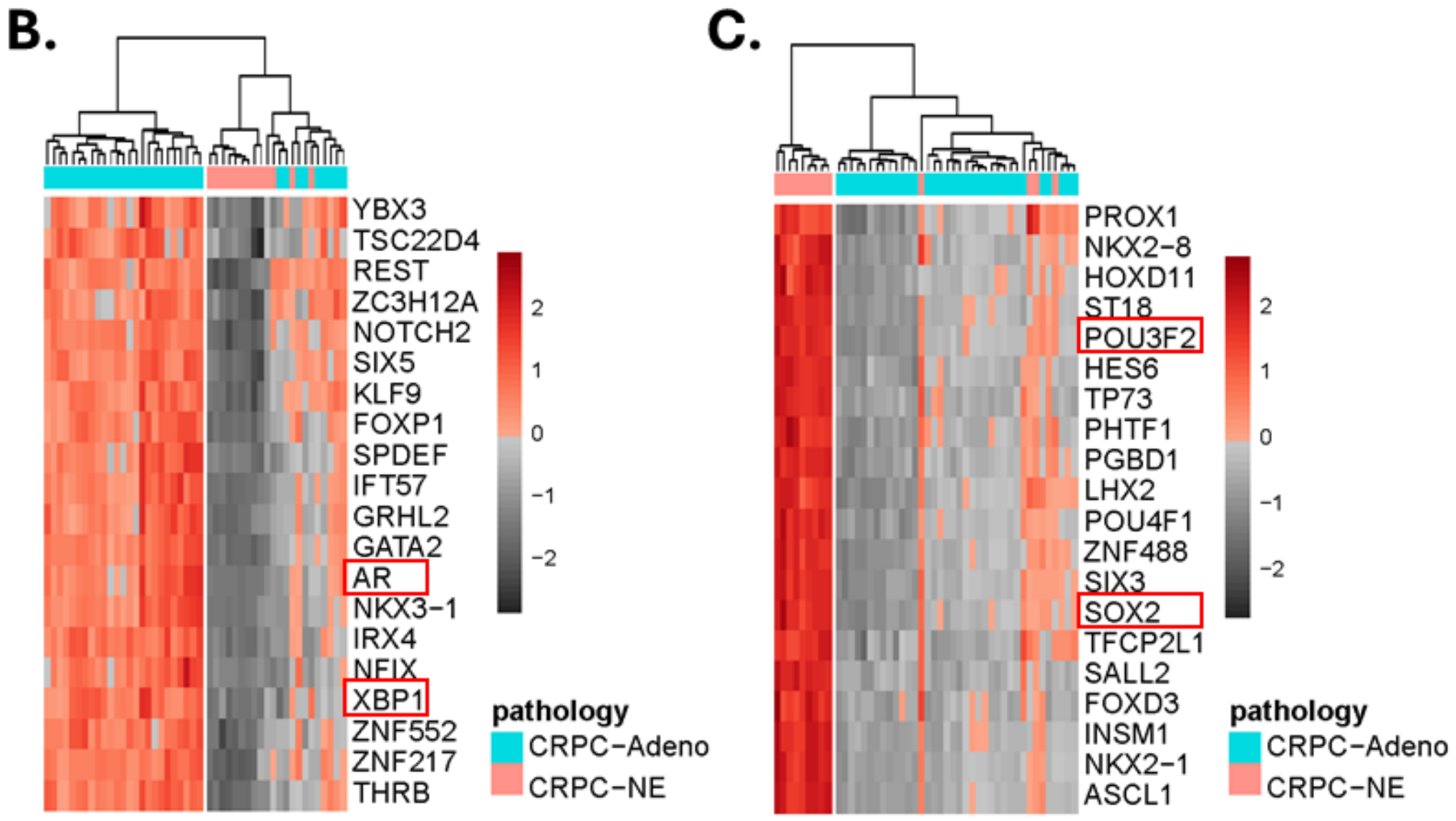

- The authors used LNCaP cells as an AR positive cell. There are many other AR positive cells that are castration resistant. Would IRE1 behave the same way in AR-positive CRPC cells? C42B?
  We have introduced AR+ CRPC cell lines C4-2B and 22RV1 to showcase that IRE1 perturbation induces similar changes to the mRNA of lineage and club markers in these lines (figures 3F-H).

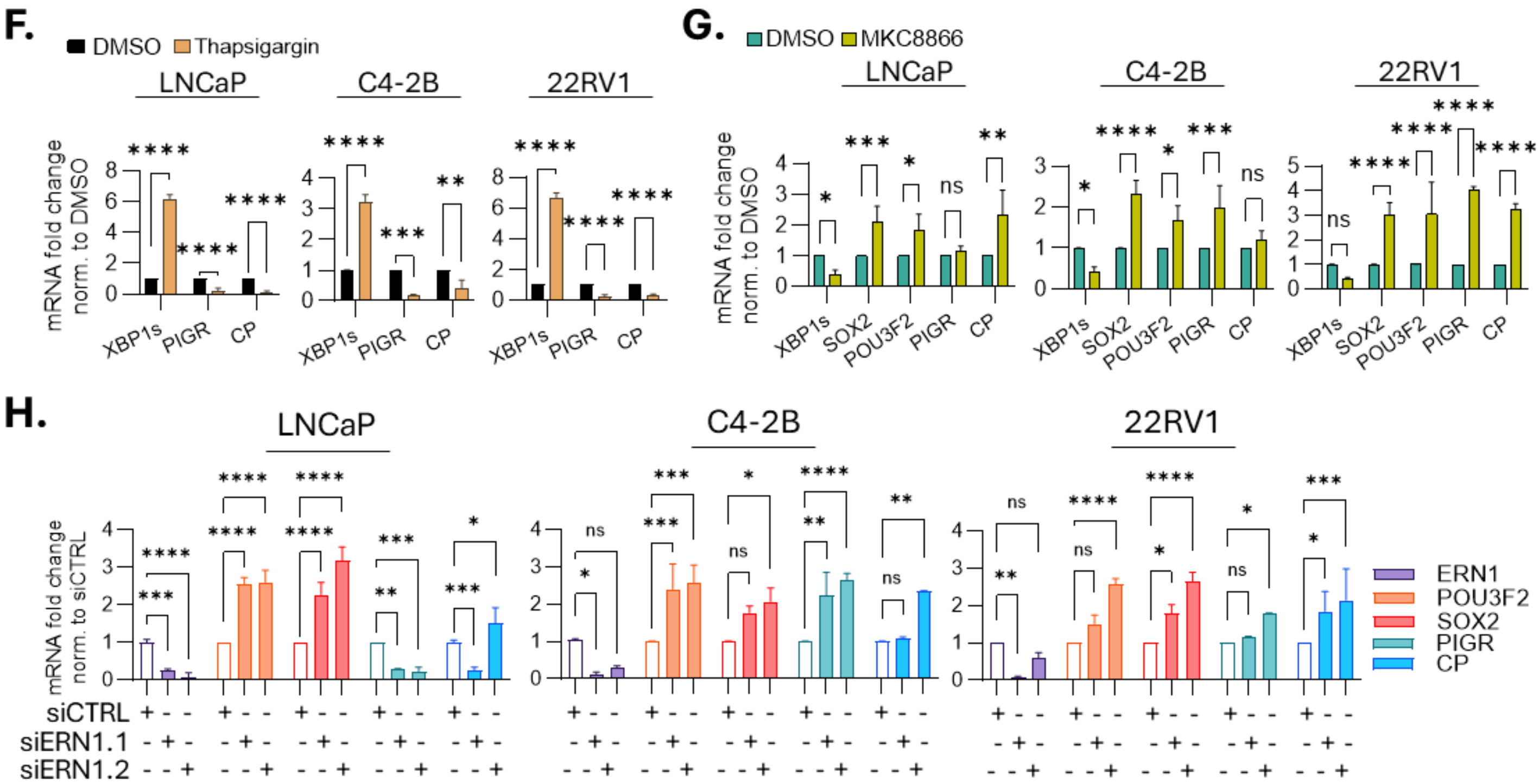

- Materials and Methods: Explain how tumoral, luminal, basal and club cells were distinguished from one another.
  Information on cell type definition can be found in the materials and methods section on lines 985-994.
- In the Introduction, please explain in detail the structure of IRE1 and how the Q780 mutation affects its structure and function.
  Information the IRE1 structure and Q780* mutant has been added in lines 81-85.
- Line 71: Please elaborate on the functions of club cells (Clara cells) in the prostate. These are commonly found in lung cells, but also seen in the prostate, especially in PCa but are mostly present in the prostatic urethra and collecting ducts, and are associated with inflammation (line 243). Authors have not explained their role in IRE1-related disease.
  We have introduced further commentary on club cells in the introduction in lines 98-100, 315-317. Until this manuscript we are unaware of any links between IRE1 activity and the club phenotype. As such, any role in IRE1 related disease would be at this stage purely correlative and hence we would refrain from making any such assertion without follow-up data. We have shown that IRE1 loss induces a club-like phenotype in cancer cells. We are going to embark on a full research programme that tries to determine whether IRE1 loss can actually induce a bona-fide club phenotype in non-tumoral cells.

- Figure 2a: include p-values.
  We have added results of 2-way ANOVA for all three graphs in figure 1E.

  Figure 1

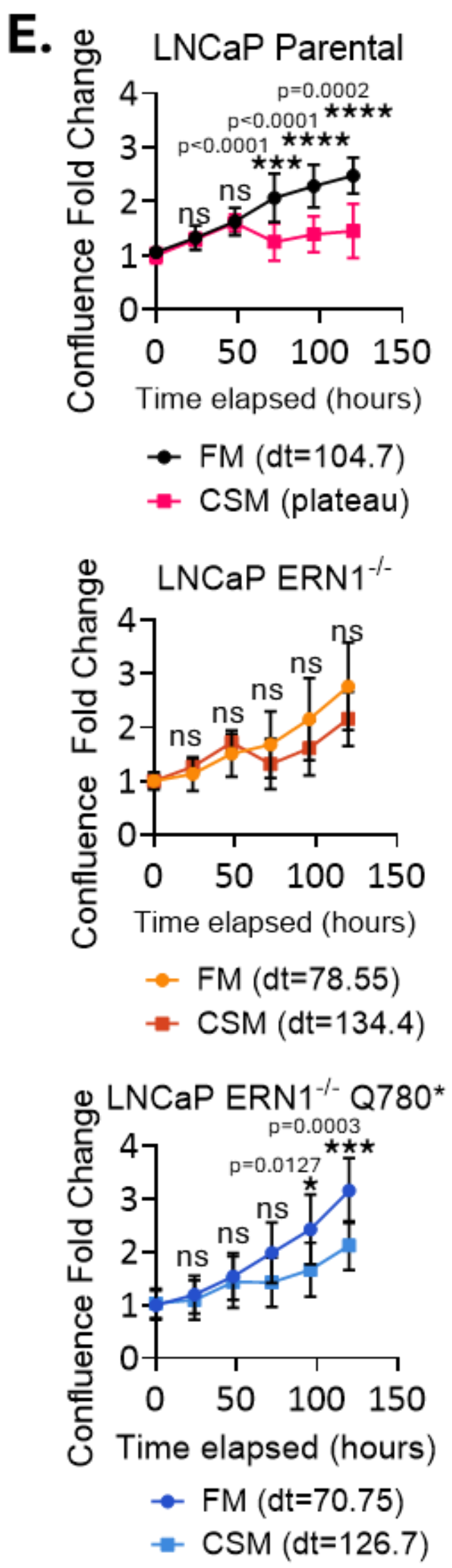

- Line 196-198: describe which ones are significantly upregulated vs those that are downregulated.
  Changes have been introduced in lines 212-219.

- Explain in the Discussion how MKC8866 inhibits IRE1 and how IXA4 activates it.

  Changes have been introduced in lines 232-234. We did not feel that the mechanisms of action of these compounds that have been extensively published merited any space in the discussion section.

Reviewer 2

Summary comments:

In this manuscript, Doultsinos and colleagues are examining the role of the unfolded protein response (UPR) sensor IRE1 in prostate cancer progression, lineage plasticity, and therapeutic response by integrating bulk, single-cell, and spatial transcriptomic data from various preclinical models, including cell lines, patient-derived explants (PDEs), and clinical tumor samples. Using this integrative approach the authors develop a prostate cancer-specific IRE1 activity gene signature (dubbed IRE1_18), which stratifies patients by tumor subtype, AR dependency, histological grade, and therapy response. Moreover, the authors demonstrate that loss of IRE1 leads to transcriptomic changes associated with lineage reprogramming, including the emergence of club cell-like features and increased expression of stemness-associated factors (such as SOX2, EZH2, and POU3F2). This suggests that IRE1 may play a critical role in maintaining luminal epithelial identity and that loss of IRE1 may create a permissive state for treatment-resistant phenotypes to emerge. Although this study could be of translational potential, the study has several limitations that should be addressed to improve the manuscript. While the authors describe correlations between loss of IRE1 and lineage plasticity, the mechanistic explanation of this relationship remains illusive, in particular whether the effects are mediated through XBP1s, RIDD, or both. Further functional experiments, such as rescue experiments using specific mutants, XBP1 ChIP-seq, or combinatorial knockdown of IRE1 with lineage reprogramming factors, would strengthen the causal interpretation of the findings.

We thank the reviewer for their comments, the time commitment to reviewing our manuscript and seeing the translational potential in this kind of work. We agree that using single cell line models and single RNA silencing reagents do not robustly demonstrate how changing IRE1 activity may alter these phenotypes. To this end we have used extra siRNA sequences and expanded our cell line repertoire to more comprehensively catalogue the responses to IRE1 loss of different models. We agree that we should determine in detail how IRE1 activity impacts each reprogramming or club-like gene. However, we feel that as evidenced in this pre-print: https://www.biorxiv.org/content/10.1101/594630v4 by Doultsinos et al, documenting the association of even a single gene like SOX2 with IRE1 takes the bulk of a manuscript. Having chosen to focus on the translational significance of the signature as the main output of this paper, a deep dive into the regulation of each target by IRE1 will form part of our studies in the immediate future. However, we do provide chip-seq and further actinomycin D experiments to determine whether the IRE1 induced or repressed genes are direct IRE1 targets. Furthermore, we have externally validate IRE1_18 in large clinical datasets to solidify its translational potential.

Specific comments

- The study is primarily based on correlation-based analyses, such as transcription factor regulon enrichment and gene expression clustering. While these are informative, they do not establish causality. It would be interesting to include mechanistic dissection of selected genes of the IRE1_18 signature to assess whether they are directly regulated by IRE1, if so by which of IRE1s dual functions, XBP1s or RIDD, and to elucidate their functional role in tumor progression. Moreoever, all mechanistic data come from in vitro or ex vivo models. Consider complement the data with in vivo models using IRE1 knockout or pharmacologic modulation to validate findings in more complex systems.

We have added extra siRNA sequences against IRE1 and XBP1 to our existing pharmacological and genetic modulation of LNCaP (figure 5B-E), C4-2B and 22RV1 (Supplemental figure S11) with siERN1, siXBP1, MKC8866, IXA4 and Thapsigargin to validate the up or downregulation of genes within the IRE1_18 signature:

Figure 5

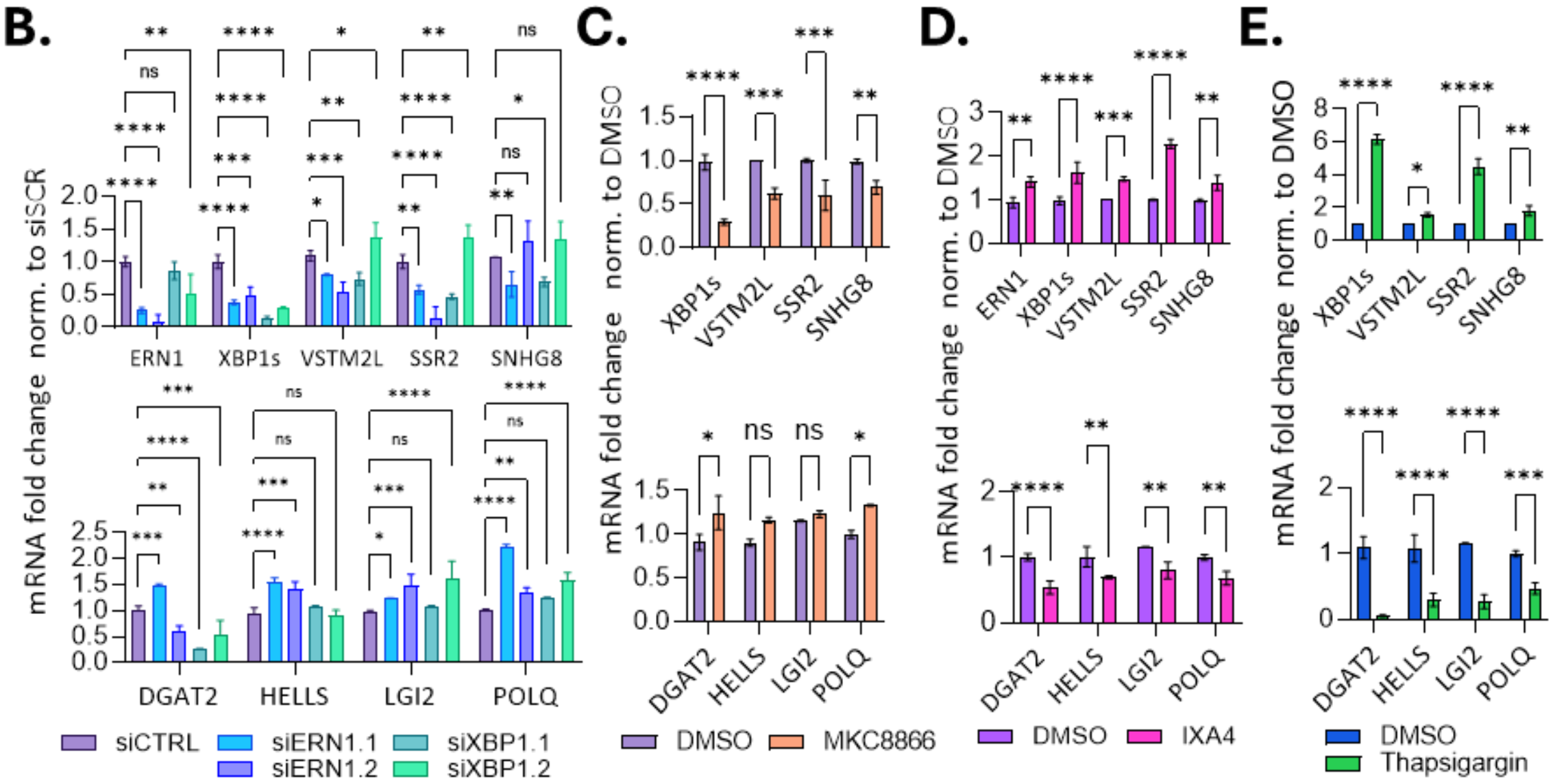

Supplementary Figure S11

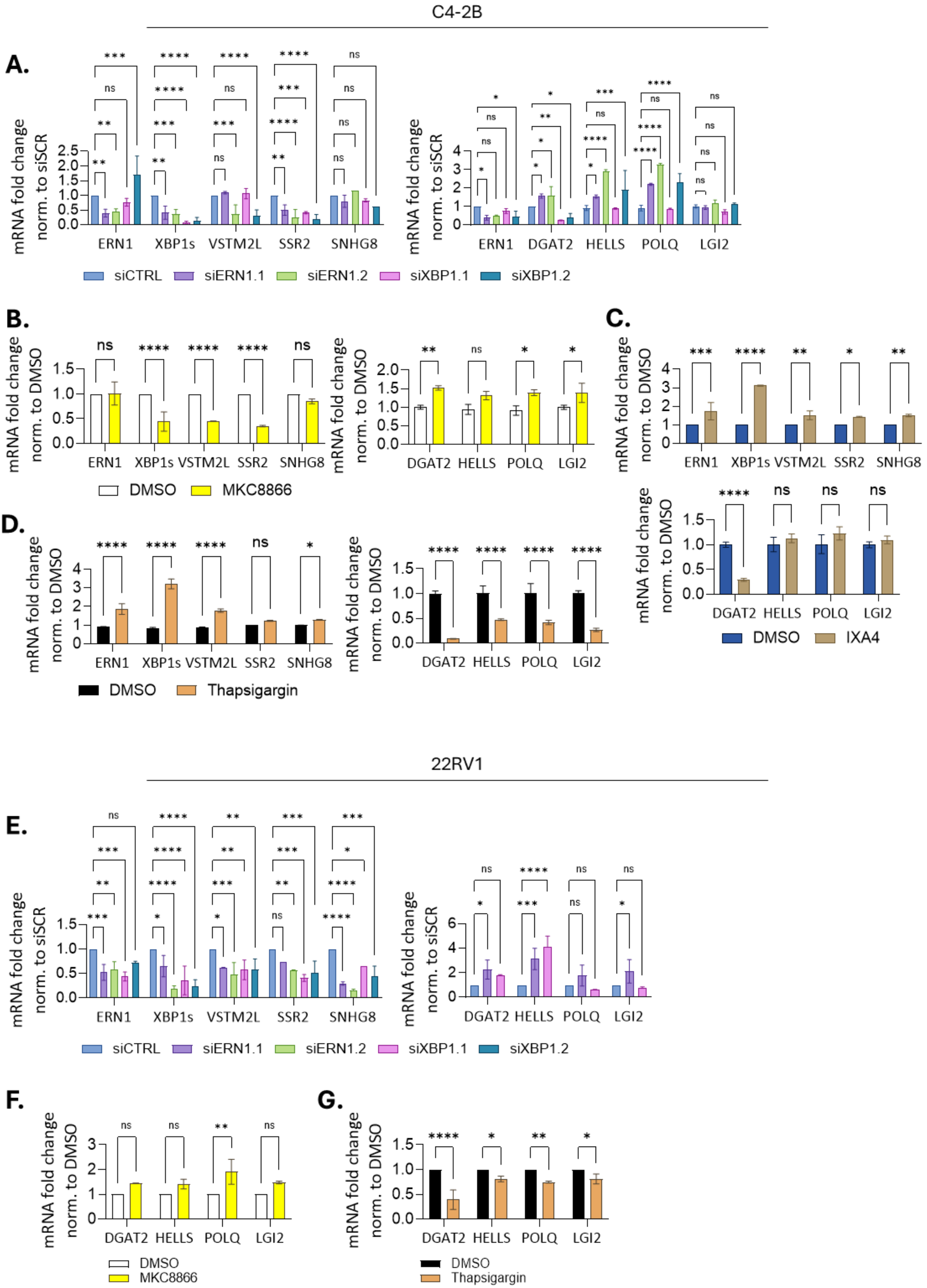

Furthermore, we carried out actinomycin D co-treatment experiments with MKC8866 and Thapsigargin to determine which "IRE1 repressed" genes were bona fide RIDD targets in LNCaP, C4-2B and 22RV1 cells (supplemental figures S12, S13):

Supplementary Figure S12

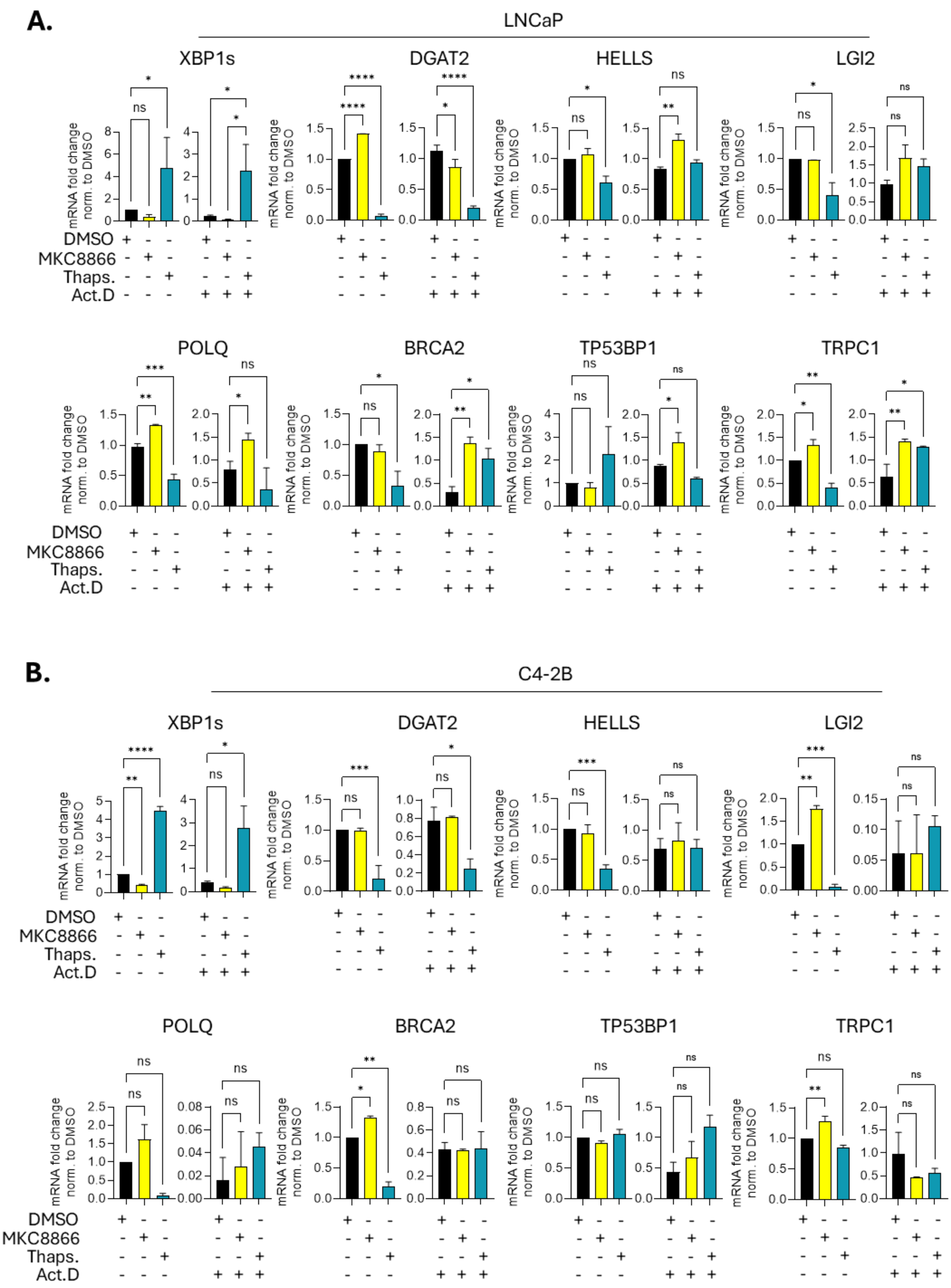

Supplementary Figure S13

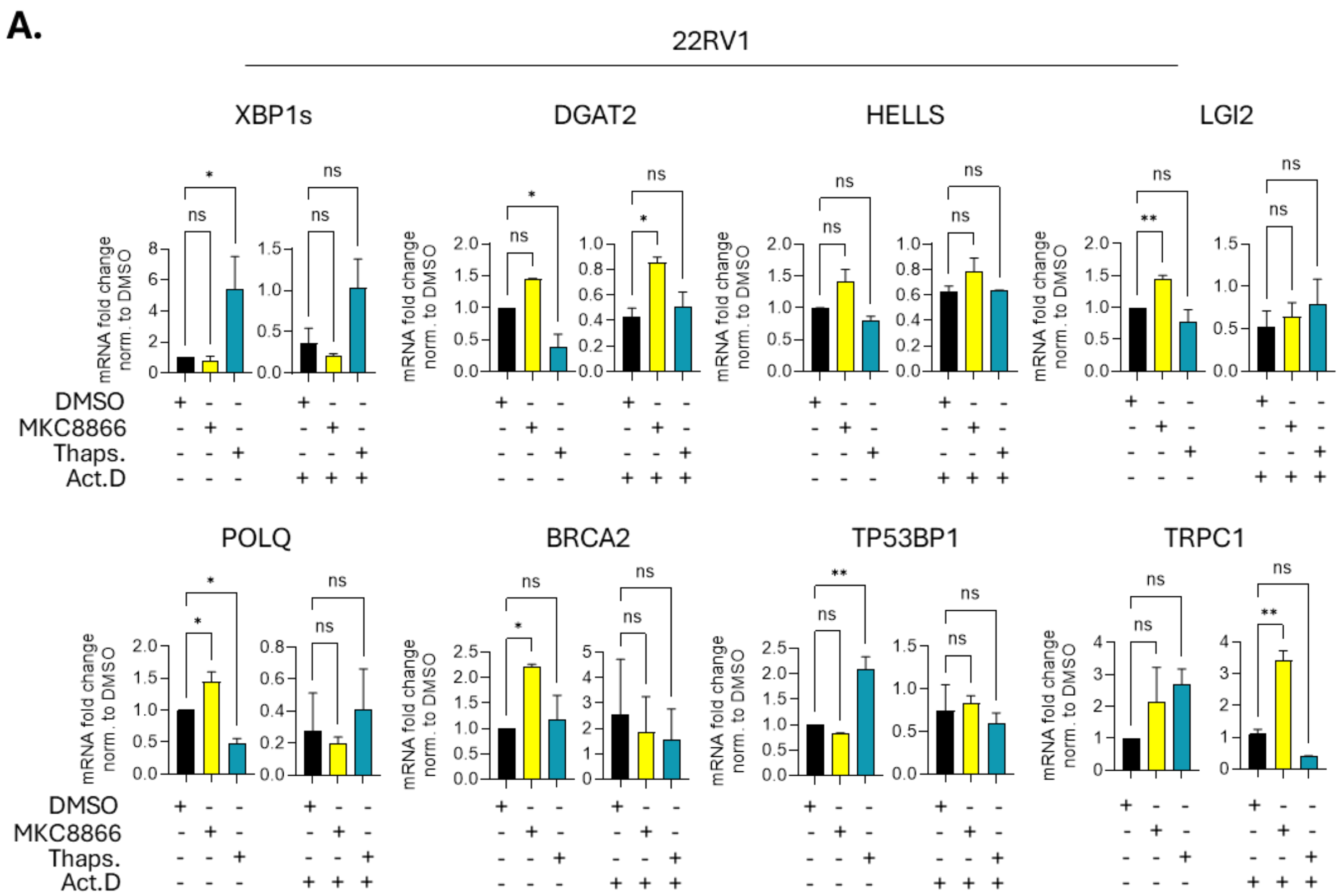

Finally, we determined in XBP1 CHIPseq datasets if IRE1 induced or IRE1 repressed genes had XBP1 peaks near their TSS and showed that XBP1 is present at several induced genes' TSS but is much more distant from the TSS of repressed genes (Supplementary Figure S10):

Supplementary Figure S10

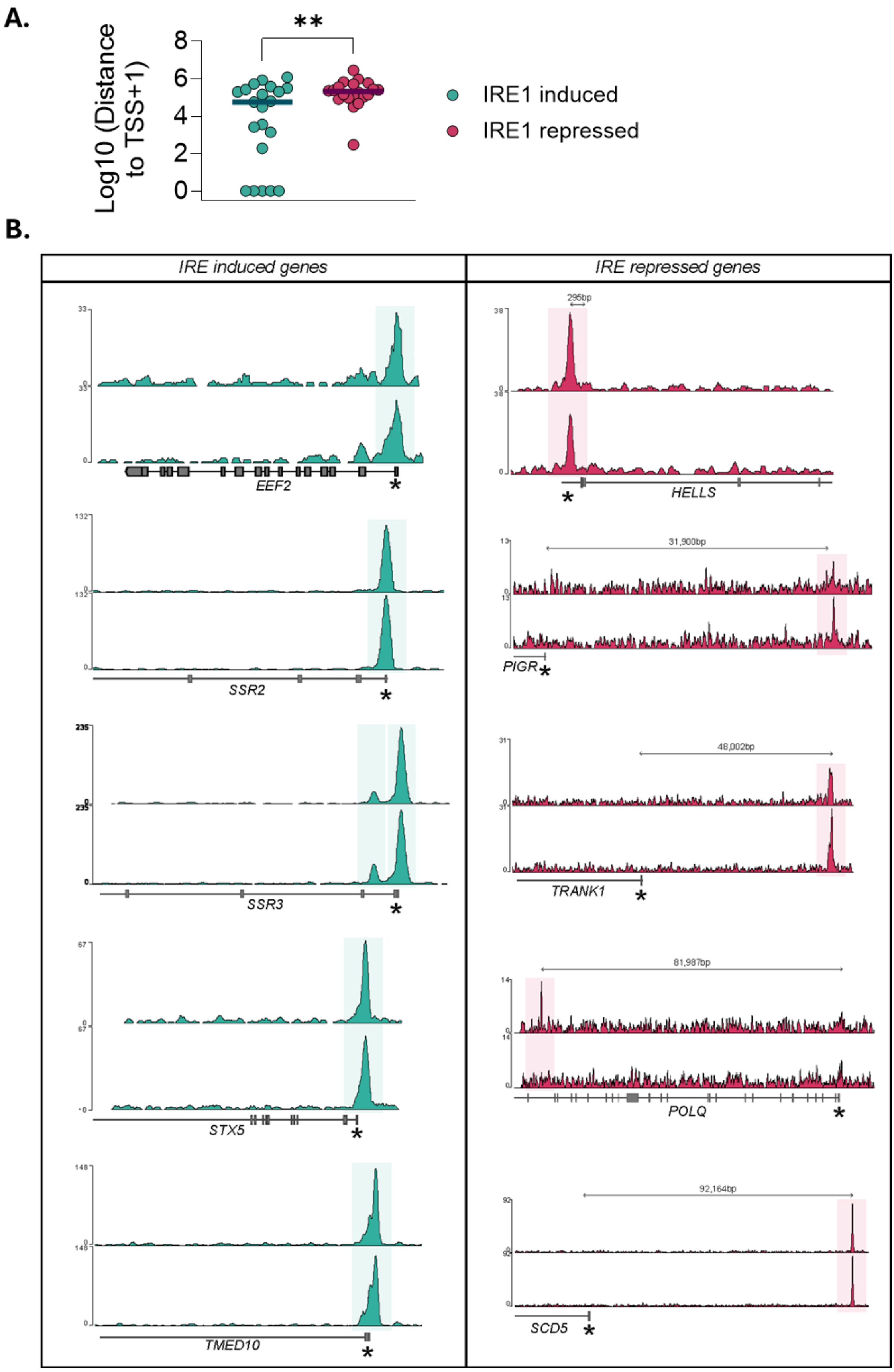

- The authors claim that IRE1-responsive genes are prostate cancer-specific (line 359), but the basis for this specificity is unclear. Was this tested directly? How does the gene signature perform in other cancers compared to normal tissue is this relevant?? Moreover, for the downstream targets included in the IRE1 signature (Figure 5), it is important to distinguish between genes regulated via XBP1s and those dependent on IRE1, but not XBP1s (i.e., potential RIDD targets, see also comment #1).

  We agree that genes in our signature may not be prostate specific and it is not within the scope of this study to investigate other cancers. What we know is that these genes performed better prognostically than consensus IRE1 activity genes in prostate samples. The wording in the original manuscript was not clear enough to reflect that prostate specificity only stemmed from deriving the genes from prostate models and tissues. The text has been amended to reflect this and any mention of prostate specificity has been removed.

- The IRE1_18 signature lacks external validation in independent patient cohorts with prospective clinical outcomes. To strengthen its clinical relevance, validation in additional datasets is recommended. Furthermore, assessing its applicability in liquid biopsies would be of interest. The authors propose IRE1_18 as a prognostic biomarker. However, key metrics such as effect sizes, AUCs, and hazard ratios are not clearly reported. To better support this claim, it would be valuable to include multivariate survival analyses comparing IRE1_18 with established clinical predictors (e.g., PSA) and other genomic signatures. Inclusion of ROC curves, such as in Figure 6D, would help quantify its prognostic performance. For the analyses using the DKFZ and PCF/SU2C cohorts, presenting odds ratios or hazard ratios in a summary table would more effectively illustrate whether increasing Gleason scores are associated with changes in gene expression. Additionally, it should be clearly stated whether the observed changes represent upregulation or downregulation to enhance biological interpretability.

  Liquid biopsy testing is indeed of interest but considering the volume of work within this study further testing in liquid biopsies will form future investigations as standalone studies and the discussion has been changed to reflect this.

  Furthermore, we have validated the IRE1_18 signature in the GRID (55000 patients, Veracyte) and META855 cohorts (855 patients) against multiple clinical correlates and outcomes such as metastasis free survival, prostate cancer specific mortality, Gleason Grade, NCCN risk scores and Decipher scores correlating it with AR signatures, univariate and multivariate analyses and different clinical designations and biologies (Figures 5G-M, 6C-G, Supplementary Figures S16, S19, Supplementary tables S26-S32):

Figure 5 *"clearly stated whether the observed changes represent upregulation or downregulation to enhance biological interpretability."* We have introduced data to show both induced and repressed components of the signature tracking with AR or other biologies such as DNA repair (5J, M, Supplementary figure S16) and correlation with response to ARSI in complex systems (PDEs) or patient data (5K,L). Moreover, we have correlated induced and repressed components with clinical designations in extra datasets (5I, West Coast Cohort, 200 samples) in addition to existing TCGA (5G, 493 samples), and Labrecque (5H, 98 samples).

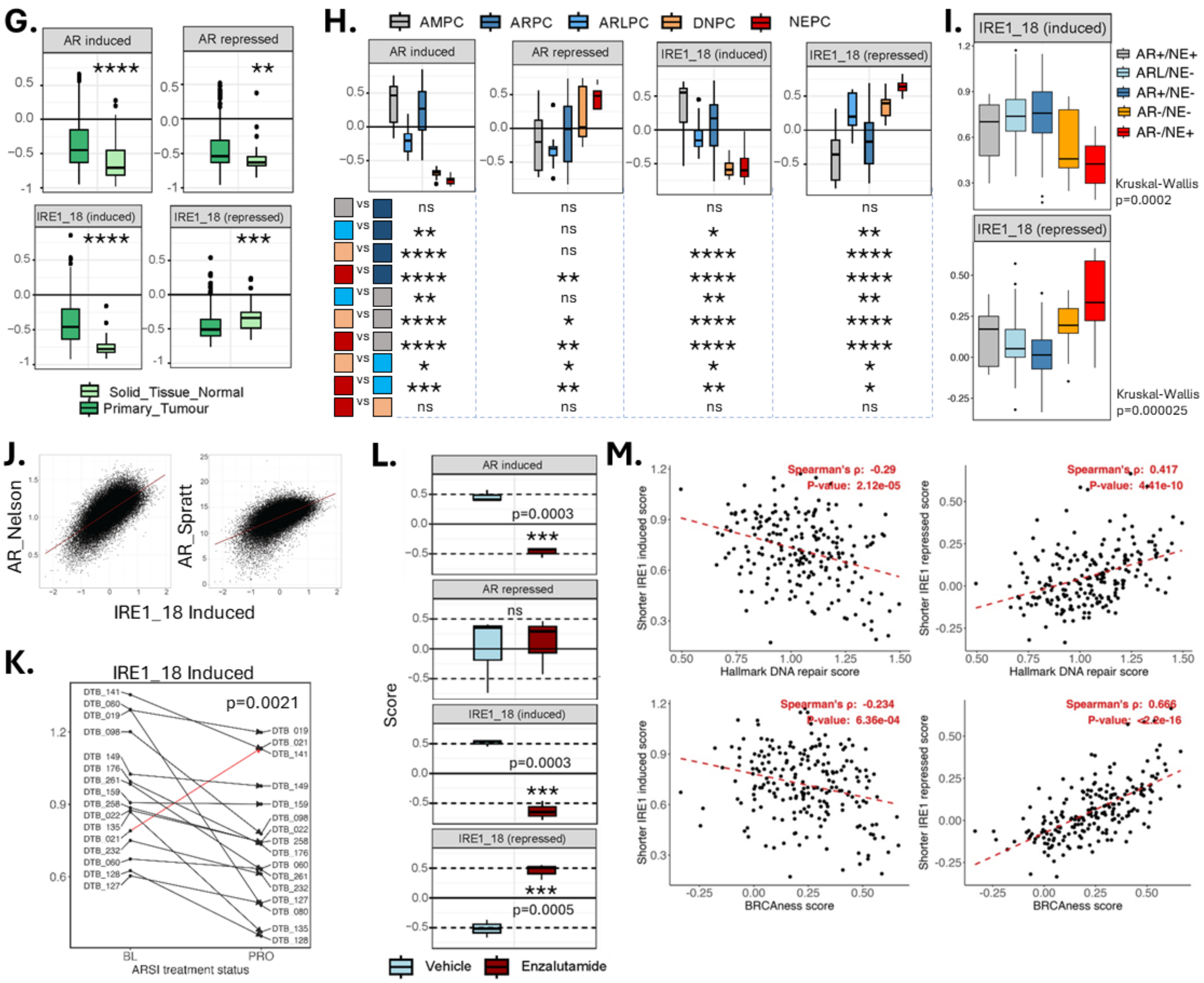

Figure 6. *"more effectively illustrate whether increasing Gleason scores are associated with changes in gene expression."* We have correlated both IRE1 induced and repressed parts of the signature with Gleason Grade, Decipher score and high NCCN risk scores (Figure 6C) as well as produce Kaplan Meier curves for IRE1_18 induced, repressed, mixed induced/repressed and unified score in multiple prospectively collected cohorts (PCF/SU2C, TCGA PRAD, META855, WCDT) (Figures 6D-G, Supplementary figure S19, Supplementary tables S26-32)

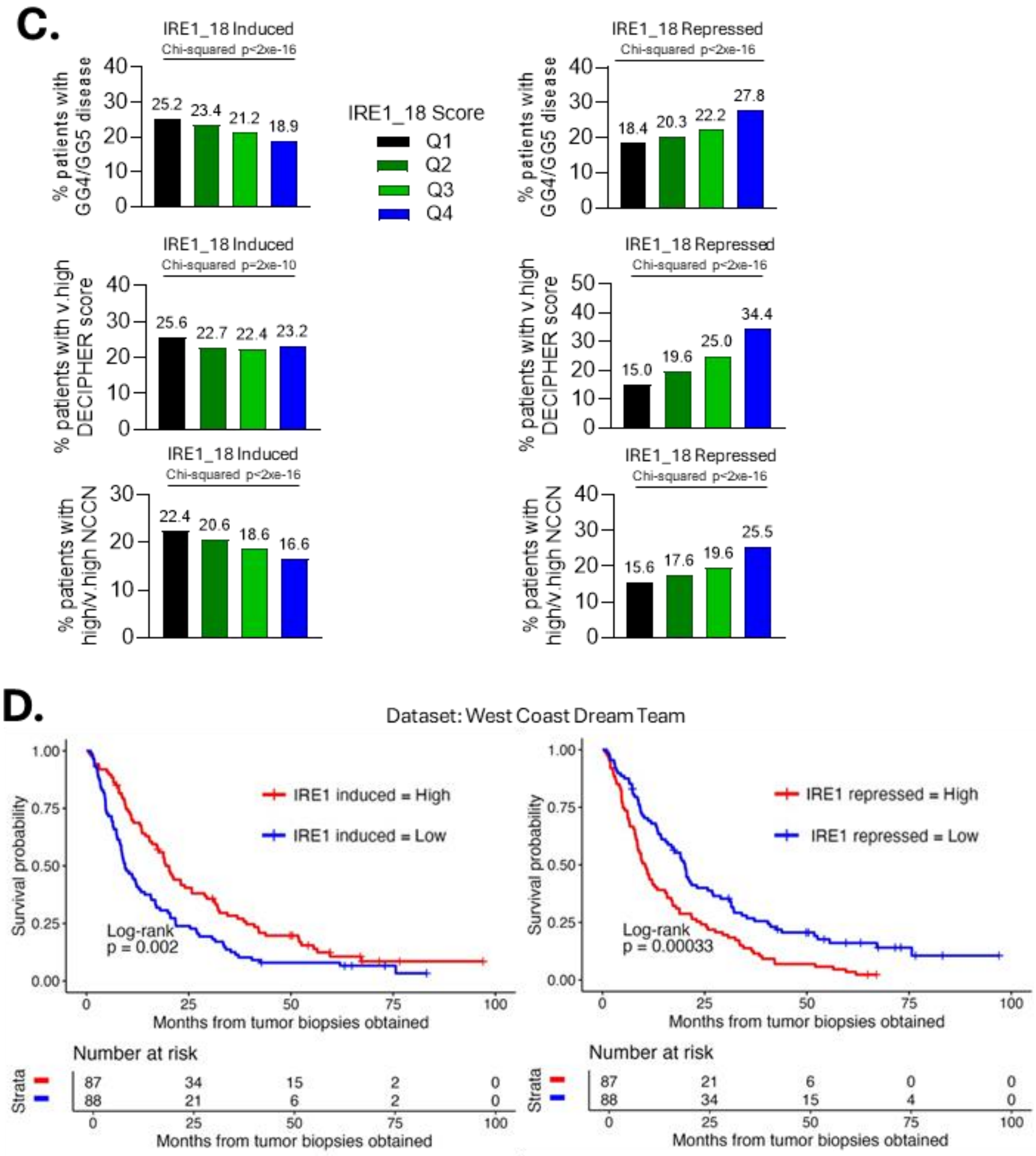

E.

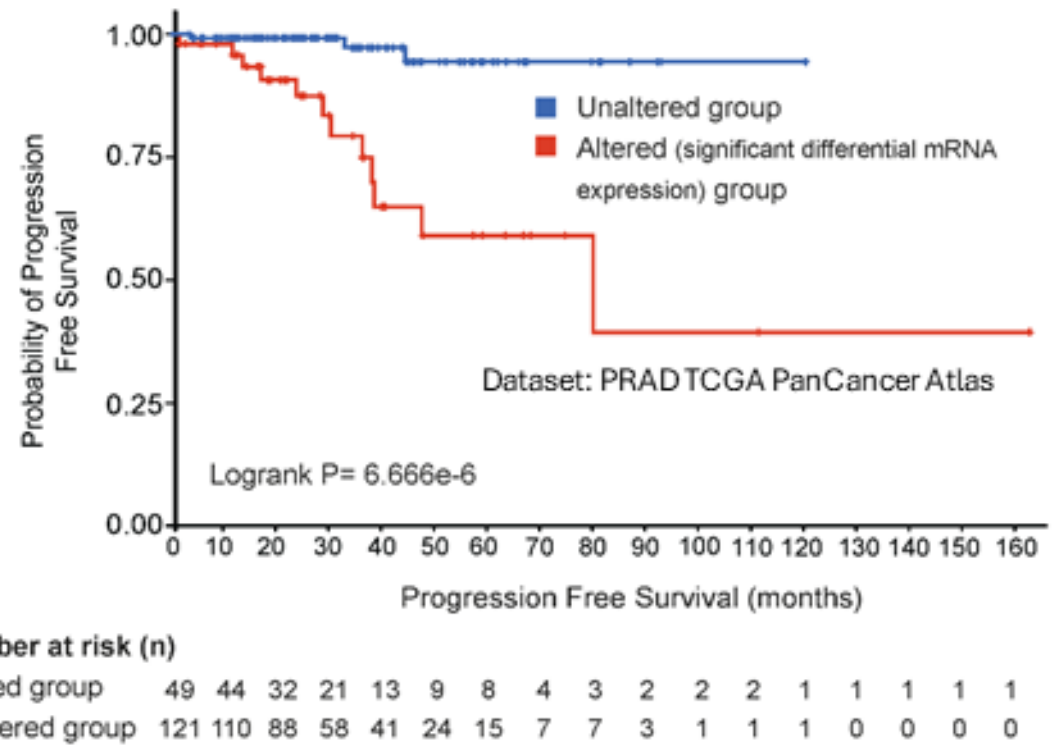

F.

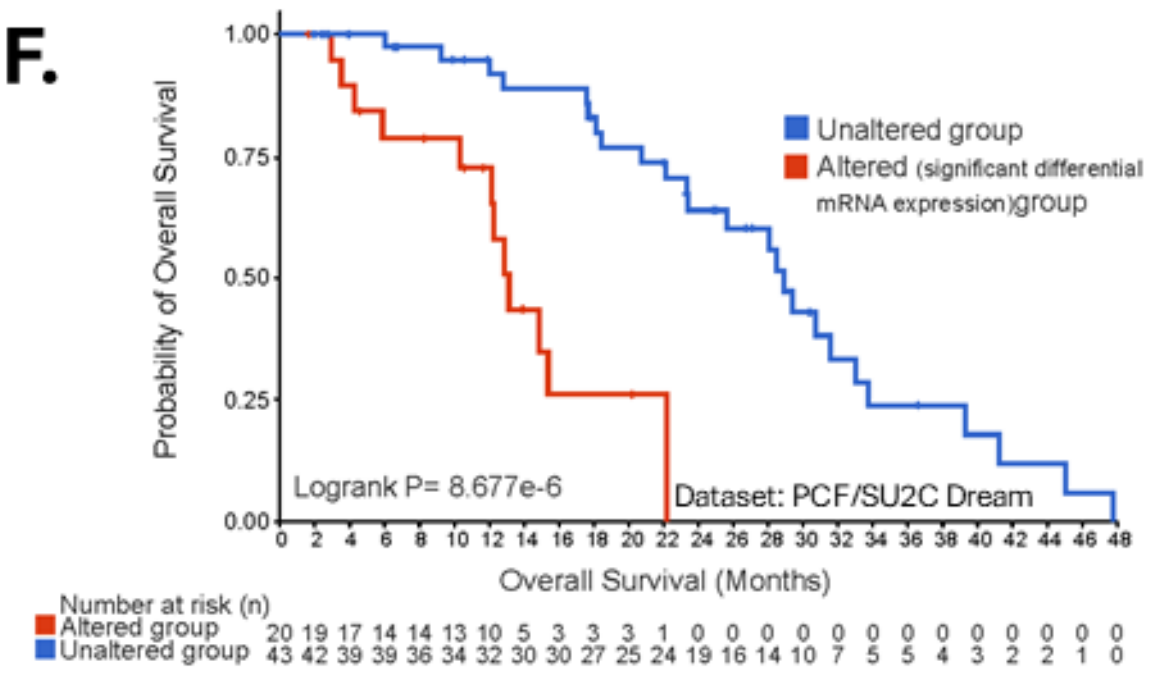

G.

Variable	Metastasis: Univariate HR [95% CI]	Univariate pvalue	Multivariate HR [95% CI]	Multivariate pvalue
IRE1_18 quartiles				
Q2-3	ref	1	ref	1
Q1	2.05 [1.29-3.2]	0.002	2.17 [1.31-3.57]	0.002
Q4	0.56 [0.28-1.09]	0.09	1.03 [0.5-2.11]	0.93
RP Gleason				
≤3+4	ref	1	ref	1
4+3	3.19 [1.7-6]	0.0003	2.64 [1.33-5.25]	0.005
≥8	6.16 [3.6-10.5]	2.00E-11	3.75 [2.04-6.91]	2.00E-05
log2PSA	1.31 [1.06-1.61]	0.01	1 [0.79-1.25]	0.98
Positive surgical margin (SM)	1.07 [0.69-1.67]	0.74	1.23 [0.78-1.97]	0.36
Extraprostatic extension (EPE)	3.4 [2.14-5.46]	2.00E-07	2.39 [1.36-4.19]	0.002
Seminal vesicle invasion (SVI)	2.79 [1.82-4.27]	2.00E-06	1.75 [1.09-2.81]	0.02
Lymph node invasion (LNI)	5.8 [3.46-9.7]	2.00E-11	3.34 [1.9-5.88]	2.00E-05

Dataset: META855

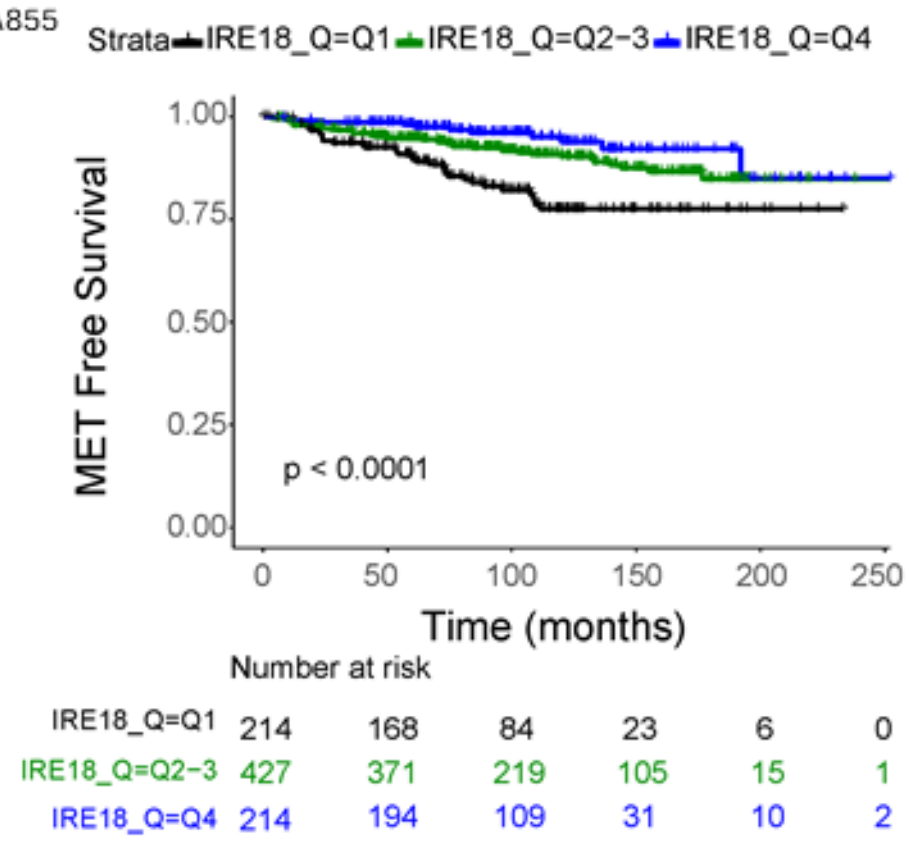

Supplementary Figure S16

Supplementary figure S16

A.

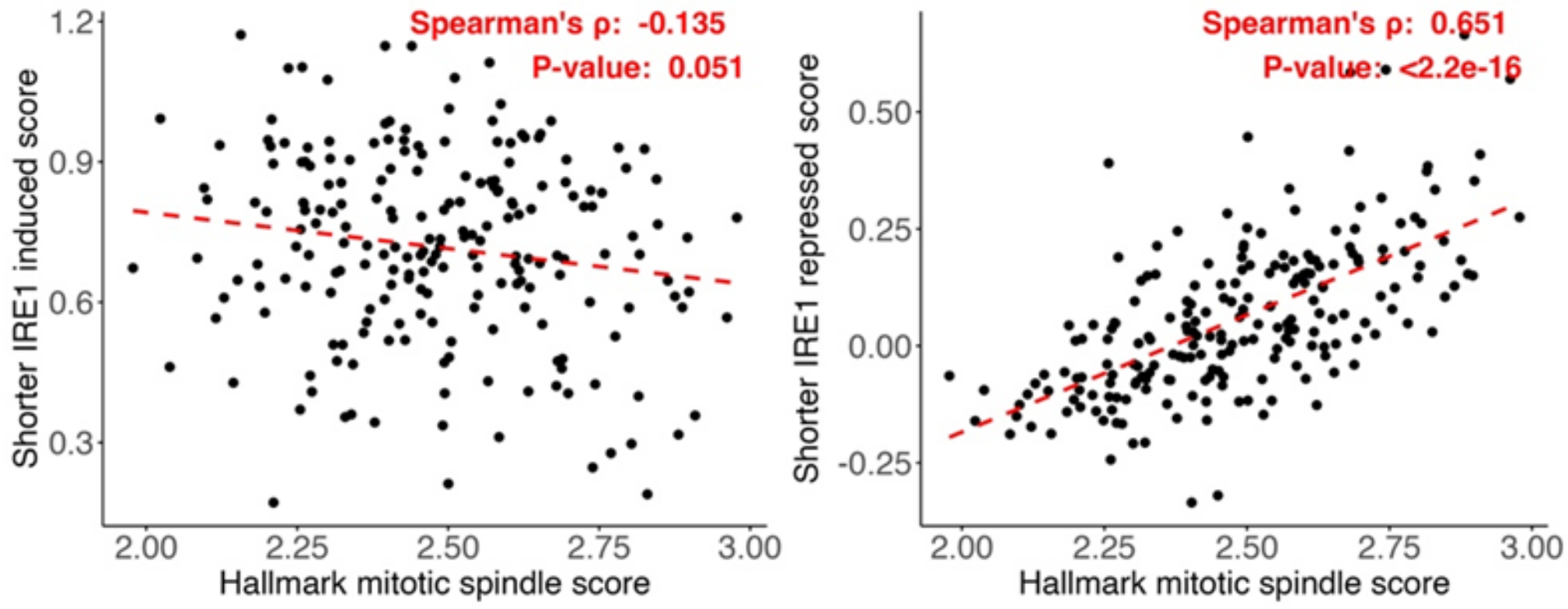

Supplementary Figure S19

Supplementary figure S19

A.

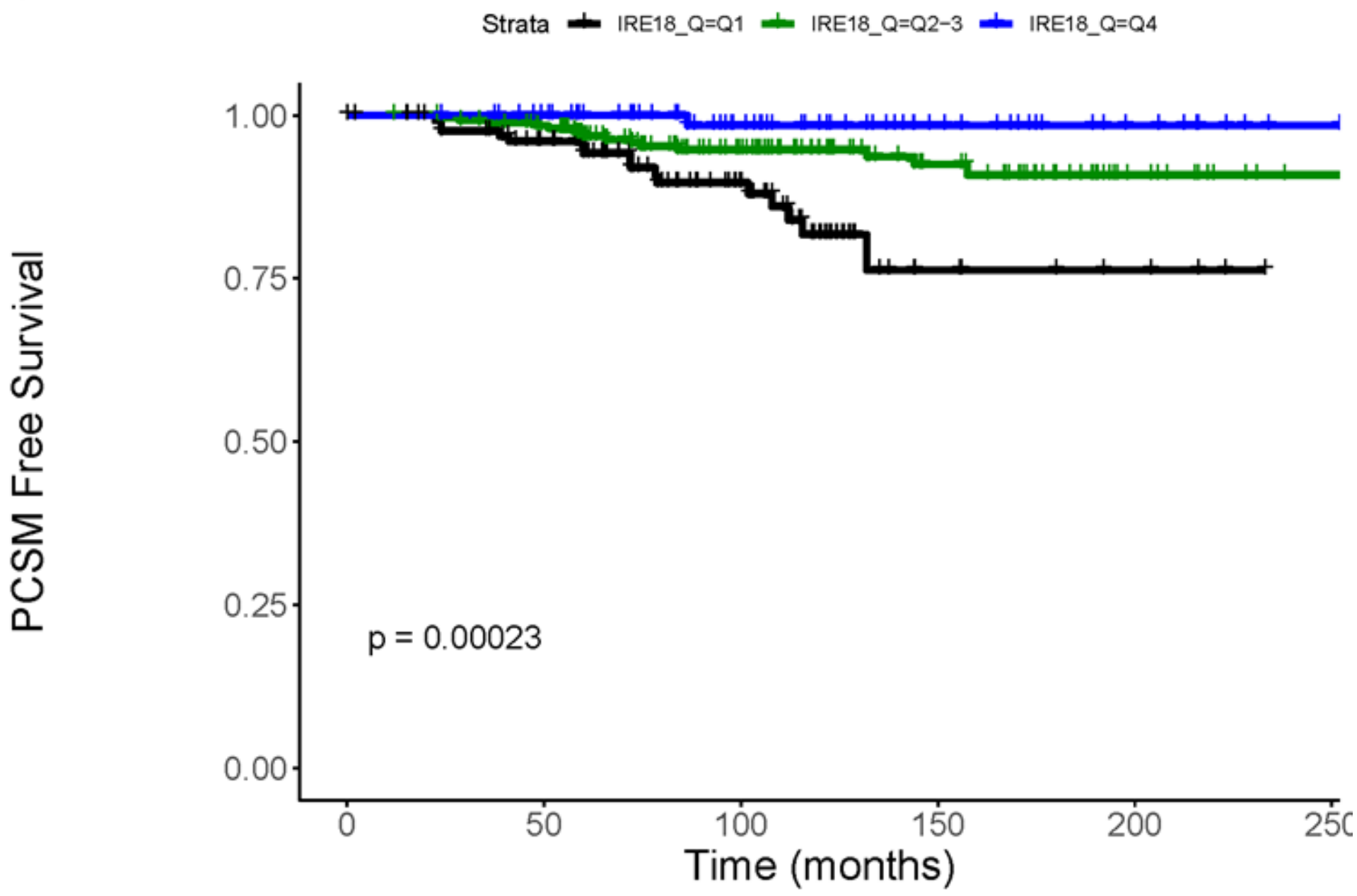

Number at risk

	0	50	100	150	200	250
IRE18_Q=Q1	133	112	55	10	5	0
IRE18_Q=Q2-3	258	244	142	66	15	1
IRE18_Q=Q4	101	95	54	22	9	2

Supplementary tables

IRE1_18 induced scores pairwise comparison between subtypes West Coast Cohort				
Benjamini–Hochberg corrected padj	AR+/NE+	ARL/NE-	AR+/NE-	AR-/NE-
ARL/NE-	0.3897	/	/	/
AR+/NE-	0.3666	0.925	/	/
AR-/NE-	0.3096	0.0087	0.008	/
AR-/NE+	0.0458	0.001	0.0027	0.3897

IRE1_18 repressed scores pairwise comparison between subtypes				
Benjamini–Hochberg corrected padj	AR+/NE+	ARL/NE-	AR+/NE-	AR-/NE-
ARL/NE-	0.2682	/	/	/
AR+/NE-	0.0867	0.0867	/	/
AR-/NE-	0.3237	0.0414	0.0039	/
AR-/NE+	0.0867	0.0068	0.0039	0.2682

IRE1 signature scores correaltion with DNA repair and proliferation signature scores West Coast cohort				
Spearman's ρ (p value)	Longer.IRE1.induced	Longer.IRE1.repressed	Shorter.IRE1.induced	Shorter.IRE1.repressed
Hallmark DNA repair	-0.278 (4.6e-5)	0.345 (3.38e-7)	-0.29 (2.12e-5)	0.417 (4.41e-10)
BRCAness	-0.261 (1.39e-4)	0.586 (<2.2e-16)	-0.234 (6.36e-4)	0.666 (<2.2e-16)
Hallmark mitotic spindle	-0.232 (7.33e-4)	0.544 (<2.2e-16)	-0.135 (0.051)	0.651 (<2.2e-16)

	GRID RP
Total	55,329
Age median [IQR]	65 [60-69]
Pathology Grade Group (GG)	
1	2,398 (4.3%)
2	23,488 (42.4%)
3	17,106 (30.9%)
4	4,279 (7.7%)
5	7,999 (14.5%)
Decipher risk groups	
Low/intermediate (<0.6)	26,719 (48.3%)
High (0.6-0.85)	15,600 (28.2%)
Very high (>0.85)	13,010 (23.5%)
Exraprostatic extension (EPE)	
Positive	30,747 (55.6%)
Negative	24,578 (44.4%)
Lymph node invasion (LNI)	
Positive	2,553 (4.6%)
Negative	52,774 (95.3%)
Seminal vesicle invasion (SVI)	
Positive	10,580 (19.1%)
Negative	44,745 (80.8%)
NCCN risk	
High/V.high	10,834 (19.6%)
unfavorable Intermediate	44,745 (80.9%)

IRE1-induced			
	GG45	Very high Decipher	Nigh/veryhigh NCCN
Q1	25.2	25.6	22.4
Q2	23.4	22.7	20.6
Q3	21.2	22.4	18.6
Q4	18.9	23.2	16.6
Chi-squared p	p<2xe-16	p=2e-10	p<2xe-16
IRE1-repressed			
	GG45	Very high Decipher	Nigh/veryhigh NCCN
Q1	18.4	15	15.6
Q2	20.3	19.6	17.6
Q3	22.2	25	19.6
Q4	27.8	34.4	25.5
Chi-squared p	p<2xe-16	p<2xe-16	p<2xe-16
IRE18 score			
	GG45	Very high Decipher	Nigh/veryhigh NCCN
Q1	29.3	32.5	26.7
Q2	22.8	24.2	20.3
Q3	18.9	20.3	16.2
Q4	17.8	17	15.1
Chi-squared p	p<2xe-16	p<2xe-16	p<2xe-16

"To better support this claim, it would be valuable to include multivariate survival analyses comparing IRE1_18 with established clinical predictors (e.g., PSA) and other genomic signatures." We have included multivariate and univariate analysis on metastasis and PCa specific mortality against PSA, RP Gleason and other metrics in 855 patients in the META855 cohort.

IRE1_18 META855

	Metastasis				
Variable	Univariate			Multivariate	
	HR (95% CI)	pvalue		HR (95% CI)	pvalue
IRE-quartiles					
Q2-3	ref	1		ref	1
Q1	2.05 [1.29-3.2]	0.002		2.17 [1.31-3.57]	0.002
Q4	0.56[0.28-1.09]	0.09		1.03 [0.5-2.11]	0.93
log2PSA	1.31 [1.06-1.61	0.01		1 [0.79-1.25]	0.98
RP Gleason					
<=3+4	ref	1		ref	1
4+3	3.19 [1.7-6]	0.0003		2.64 [1.33-5.25]	0.005
>=8	6.16 [3.6-10.5]	2.00E-11		3.75 [2.04-6.91]	2.00E-05
Positive surgical margin (SM)	1.07 [0.69-1.67]	0.74		1.23 [0.78-1.97]	0.36
Extraprostatic extension (EPE)	3.4 [2.14-5.46]	2.00E-07		2.39 [1.36-4.19]	0.002
Seminal vesicle invasion (SVI)	2.79 [1.82-4.27]	2.00E-06		1.75 [1.09-2.81]	0.02
Lymph node invasion (LNI)	5.8 [3.46-9.7]	2.00E-11		3.34 [1.9-5.88]	2.00E-05

	PCSM				
Variable	Univariate			Multivariate	
	HR (95% CI)	pvalue		HR (95% CI)	pvalue
IRE-quartiles					
Q2-3	ref	1		ref	1
Q1	2.75 [1.34-5.63]	0.005		3.07 [1.41-6.68]	0.004
Q4	0.17 [0.02-1.3]	0.09			
log2PSA	1.31 [0.92-1.87]	0.13		1.05 [0.74-1.47]	0.78
RP Gleason					
<=3+4	ref	1		ref	1
4+3	2.03 [0.66-6.23]	0.21		1.49 [0.42-5.29]	0.53
>=8	5.16 [2.25-11.85]	1.00E-04		2.86 [1.12-7.3]	0.027
Positive surgical margin (SM)	1.09 [0.54-2.2]	0.8		1.23 [0.57-2.6]	0.59
Extraprostatic extension (EPE)	2.44 [1.15-5.15]	0.02		1.18 [0.46-3]	0.72
Seminal vesicle invasion (SVI)	4.83 [2.39-9.72]	1.00E-05		2.55 [1.13-5.7]	0.022
Lymph node invasion (LNI)	4.72 [2.2-10.1]	6.00E-05		2.85 [1.23-6.62]	0.014

"it should be clearly stated whether the observed changes represent upregulation or downregulation to enhance biological interpretability." Oncoprints are added to showcase mRNA up or down regulation per gene in supplemental in the PCF/SU2C and TCGA PRAD cohorts (Supplementary Figures S18C-D).

Supplementary Figure S18

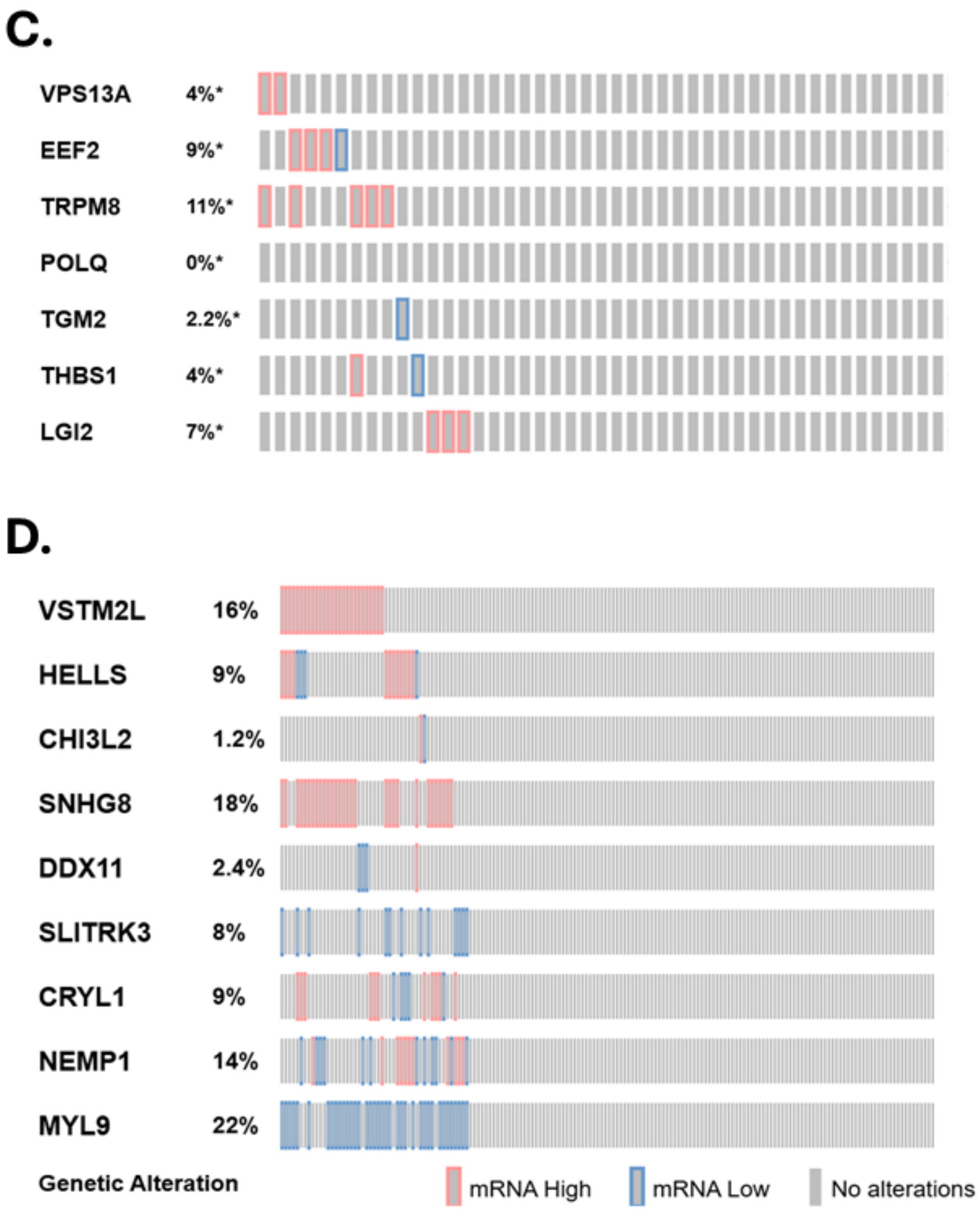

- The data presentation and writing, though comprehensive, are at times unnecessarily redundant. A more focused narrative, along with the streamlining of figures by moving less critical data to the supplementary materials would improve clarity and overall readability. Key terms such as club cells, RIDD, and enzalutamide should be clearly and precisely defined early in the manuscript.

  We have moved a significant number of figures (almost entirety of original figures 1 and 2) to the supplementary and there are changes across the manuscript trying to minimise redundancy. All terms are defined at first time of mention. RIDD is defined in line 78 of the introduction, club cells are defined in lines 98-100 and enzalutamide is defined in line 167 of the results section.

- Statistical methods are often not clearly specified. For example, in Line 391: "Recapitulating multicellularity, IRE1 activity could distinguish between SC luminal, basal and club cells," the statistical test used to assess this segregation is not reported. Similarly, Figure 5I lacks both

sample size information and details on the statistical test used to compare the two groups. Including effect size metrics, such as Cohen's d, would strengthen the analysis, assuming the data meet normality assumptions. Additionally, Figures 6A, 6B, and 6C require clearer explanation. Although the results section (lines 471-474) refers to correlations with IRE1_18 expression, the figures display correlations with individual genes rather than with the composite IRE1_18 signature, which should be addressed for consistency and clarity.

The methods used to separate different cell clusters and measure gene abundance in each cluster are described in the materials and methods “Single Cell” section. In addition, for clarification purposes we have defined IRE1 activity at this stage as IRE1 activity signature in lines 445-446. We have corrected the text to reflect that (now) figure 6B shows correlations with individual genes in the spatial transcriptomic data from a single patient. In figure 6C, we then present correlations of the IRE1_18 induced and repressed components as composites in the much larger GRID dataset.

Reviewer 3

Summary comments:

"Most of the data were based on a single cell line (LNCaP), and no animal data support the main conclusion. The regulation of IRE1 activity/UPR by androgen signaling and the role of UPR in prostate cancer progression has been reported previously. This manuscript uses LNCaP-based cell models to investigate how IRE1 signaling regulates gene expression and cellular responses to androgen deprivation. They further derived a IRE1 activity gene set and claimed this gene set could stratify prostate cancers at different stages. The authors performed excellent transcriptomic analysis and bioinformatical analysis, however, "wet" experimental evidence is often limited, lacks rigor, and not very convincing. The potential application of the described IRE1 gene signature in clinical diagnosis and treatment is unknown."

We thank the reviewer for their feedback and the time dedicated to review our manuscript. We agree that although our original figure 1 was necessary to determine the robustness of our models and was key in setting the scene for the rest of the paper, it largely describes known associations between AR signalling and the UPR with some novel observations on RIDD targets and the utility of multicellular explant models of prostate cancer. As such, we have moved original figure 1 into supplemental with the addition of Prostate cancer atlas data to showcase this parallel relationship in multiple clinical designations (Supplementary figure S1, S2). Common to comments addressed for reviewer 1 we have added data on a further three cell lines to more accurately represent prostate cancer heterogeneity.

Specific comments

1. The role of IRE1/UPR signaling in prostate cancer development and the regulation between IRE1 and AR have been reported previously. The data in Fig 1 are highly predictable. Although the authors stated in Introduction that the IRE1-RIDD branch of the IRE1 activity is largely ignored in literature, this manuscript unfortunately ignored this as well. Only limited experiments used a mutant Q780 cell line.

We absolutely agree that the data in original figure 1 have been previously reported and as such have moved it to supplemental. We agree that a role of IRE1 in PCa pathophysiology has been described and the relationship between IRE1 and AR has been the subject of studies we have cited in our manuscript. Literature has focused on the archetypal role of IRE1 as a survival adaptation mechanism in early-stage disease as seen in other malignancies such as glioblastoma. However, a more detailed map of IRE1 activity across the prostate cancer disease spectrum and evolution is what our manuscript is aiming to lay the foundations for; not just for cancer cells but also the microenvironment.

In addition to our comments above and the data presented in response to reviewers 1 and 2 we have utilised the Q780* mutant line further demonstrating the impact of IRE1 lacking an RNAse domain but present at the protein level has on mRNA of reprogramming, AR, club and cell cycling genes. We also stably overexpressed the Q780* mutant in the C4-2B line creating a dominant negative line as previously described (Figure 1D-F, 2D, 3D, Supplementary figure S7).

Figure 1

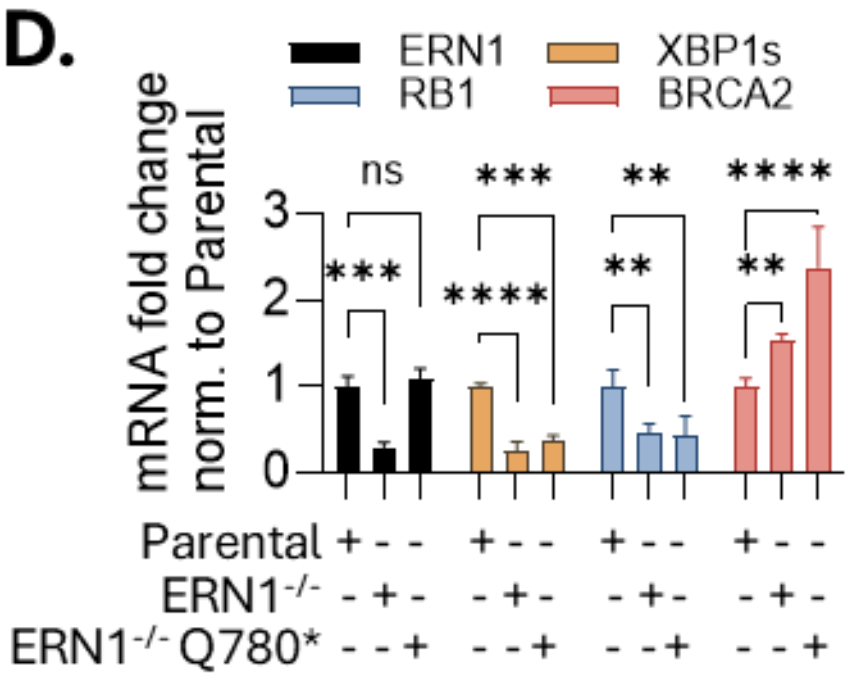

Figure 2

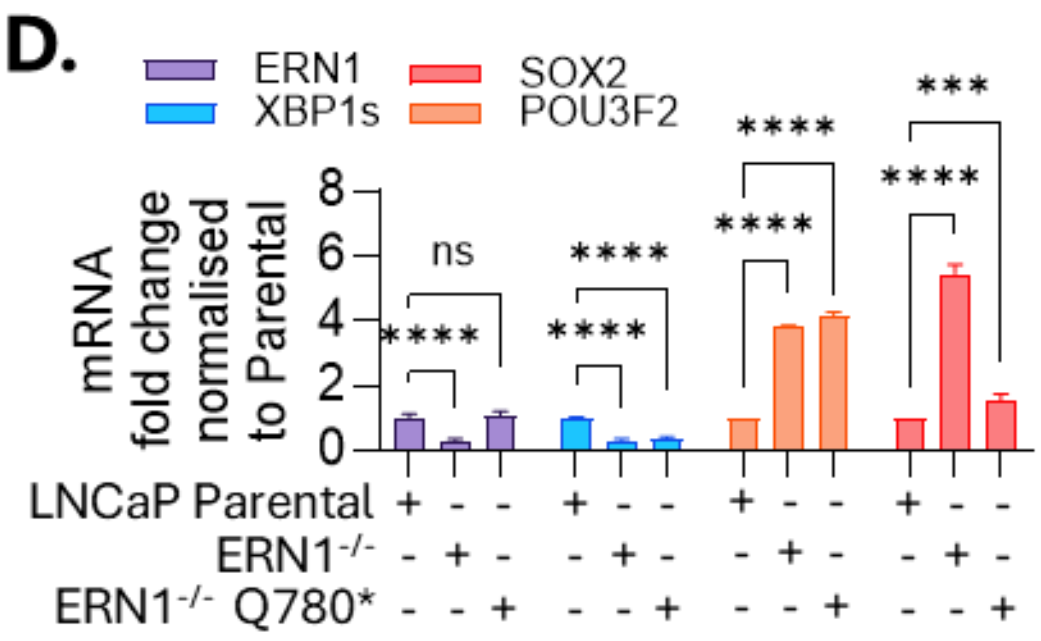

Figure 3

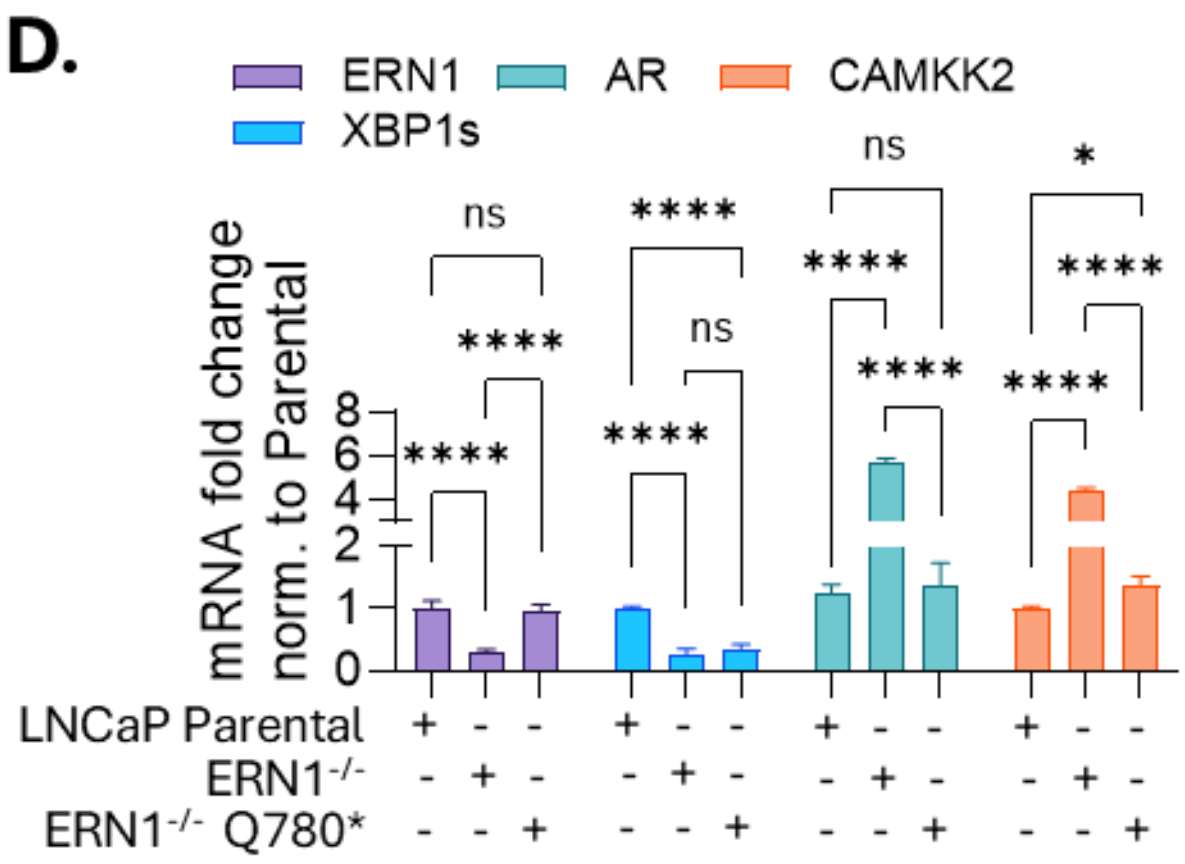

Supplementary figure S7

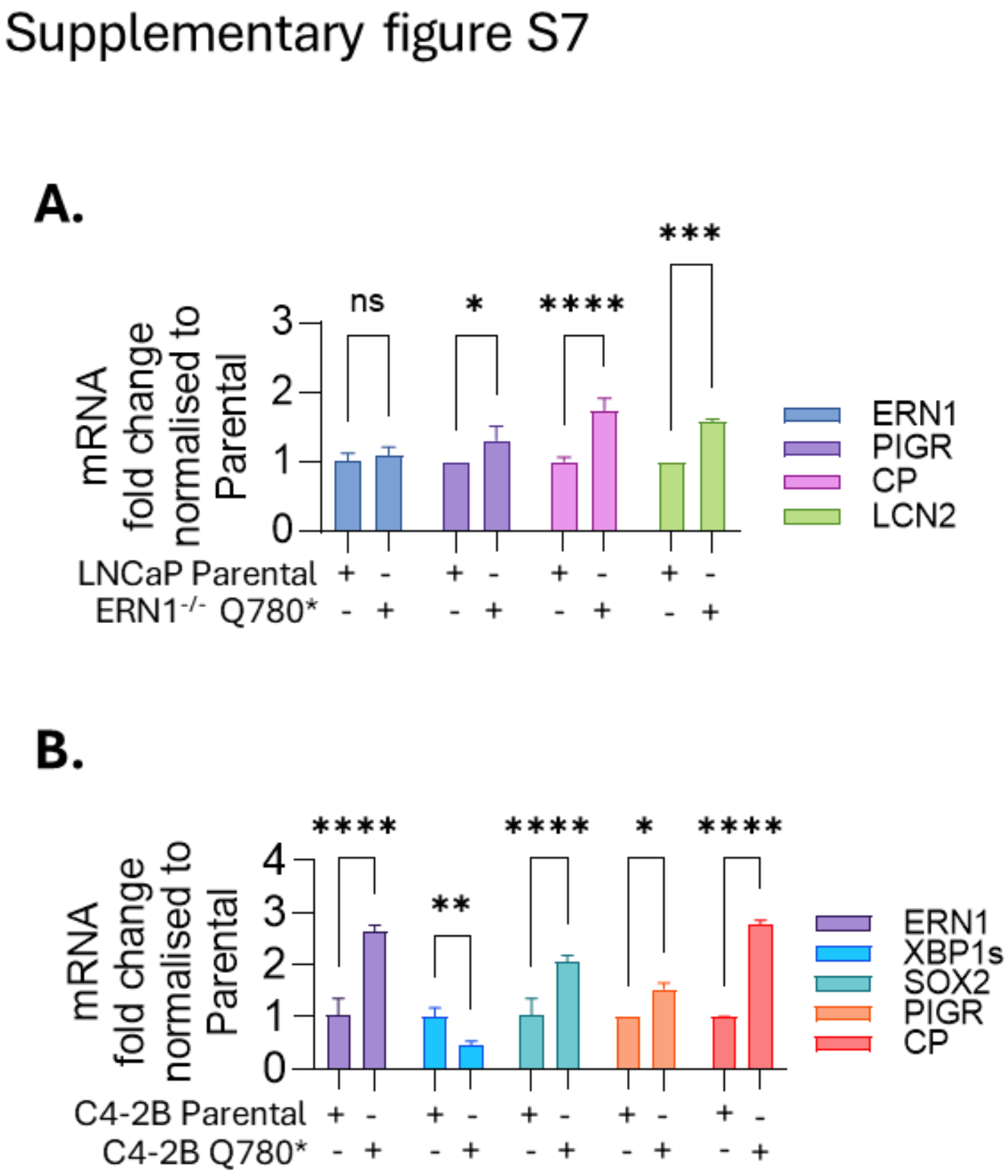

2. Most of the data are based on a single cell line model. The data need to be validated in other cell line models. It would also be interesting to investigate IRE1 activity in AR-negative cells, or castration-resistant cell models.

We agree that more cell lines should be used to recapitulate the full disease spectrum. As such we have generated data in C4-2B, 22RV1 and DU145 cells as seen in in main figures 1, 3, 4 and supplementary figures S5, S7, S8, S11, S12, S13.

We have validated expression changes of RB1, BRCA2 using two separate siRNAs against ERN1 (supplementary figure S5A,B), expression changes of SOX2, POU3F2, PIGR and CP in the presence of MKC8866 (figure 3G), expression changes of PIGR and CP in the presence of thapsigargin and expression changes of SOX2, POU3F2 and CP in the presence of two separate siRNAs against ERN1 in LNCaP, C4-2B and 22RV1.

3. The authors may need to talk more about "club" cells, as these cells are quite "new" to the field.

We have made sure to include further references in the main text in all three of introduction (in lines 98-100, 315-317.), results and discussion sections with appropriate references to make sure that club cell concepts have adequate exposure with a few recent publications such as the Kiviaho et al, Pitzen et al and Karthaus et al cited.

4. Only signal knockout clones were used to derived all the experimental data including the gene signature. The authors did not consider potential clonal variation. The data are generally not rigorous enough to support the main conclusion. Similar issues with siRNA experiments - at least two siRNAs need to be tested.

Guide RNA targeting human ERN1 (TCGGGTTTT-GGTGTCGTACA) was cloned into the PX330 CRISPR Cas9 plasmid (Harmsen et al. 2018). LNCaP cells were transiently transfected using Lipofectamine 2000 transfection reagent (Invitrogen) according to the manufacturer's instructions. After transfection, cells were selected using puromycin (0.5 µg/mL), and single clones were picked and analyzed by TIDE (Tracking of Indels by Decomposition) (Brinkman et al. 2014) and Western blot. Finally, the clone with the best editing efficiency was selected as reported in Stelloo et al, 2020. We agree that a single siRNA was not sufficient so we added a second siRNA for siERN1 and siXBP1 as demonstrated earlier in figures 2E, 3H, 5B, and Supplementary figures S5, S11.

5. Fig 3I, the ERN1 level in the RB-/- cells was reduced to almost zero (Fig 3C). If so, how can IXA4 increase the IRE1 activity in this cell line?

ERN1 mRNA is not zero, just significantly diminished as a relative steady state fold change to levels in LNCaP parental cells. We'd also like to point out that although ERN1 mRNA is very low, XBP1s mRNA in RB1 knockout cells is no different to parental LNCaP cells. As such, IRE1 activity is present and consequently can logically be amplified. IRE1 levels do not necessarily correlate with IRE1 activity and hence we have centred the entire manuscript on IRE1 activity and not IRE1 levels.

6. Fig 4A, the 3 ENZA response curves look similar. How did the authors derived different IC50 values. This is particularly problematic for ERN1-/-Q780 cells, as the calculated IC50 was 93.18 uM, but the highest concentration tested is 100 uM.

IC50s are extrapolated using inhibition regression line best fit models using Prism as defined in the materials and methods. As the measurable outcome is an ATP based luciferase assay over 12 hours and the 100µM concentration does not give a value of 0 then a sigmoidal curve cannot be generated to classically determine an IC50.

7. Fig 4G, does IXA4 alone affect cell growth?

We include data on IXA4 treatment alone in figures 4D-G to show that IXA4 treatment alone does not affect cell growth:

Figure 4:

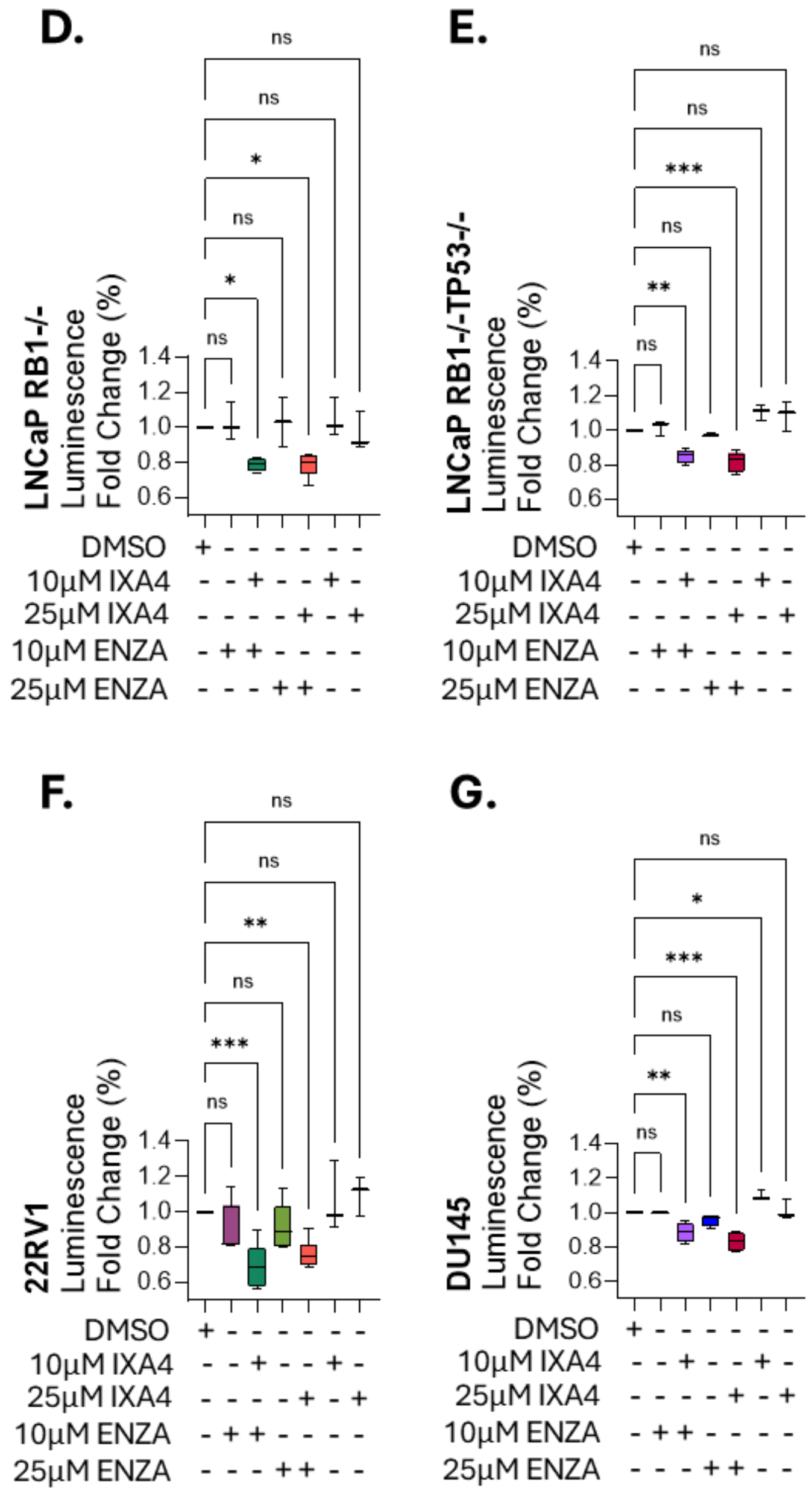

8. Many IRE1 signature genes were only marginally altered by siERN1 and siXBP1 (e.g., VSTM2L, HELLS, Fig 5B). How did the authors define a threshold value for these genes in clinical samples?

Thresholds were not chosen for individual genes. Clinical datasets were compared as totals against their controls (e.g. TCGA normal vs tumour) and accepted thresholds chosen for log2FC (>2) and adjusted p value (0.05).

22nd Jan 2026

Dear Dr. Doultsinos, Dear Dimitrios,

Thank you for submitting your revised manuscript to EMBO Molecular Medicine, and please accept my apologies for the delay in responding, which is due to the holiday season affecting both the editorial team and the referees.

The manuscript has been reviewed by the three initial referees. As you will see below, while referees #1 and #2 are satisfied with the revisions, referee #3 has remaining concerns. After discussing the manuscript and the reports further within the team and with the referees, we agreed to invite a minor round of revisions, in which we would like you to adequately discuss and, where needed, tone down the conclusions, and conduct experiments for points 5 (WB), 6 (E2F1 genes) and 7 (ERN1 protein level).

Please note that the revised manuscript will be reviewed again, and we cannot guarantee a positive outcome at this stage.

Should you find that the requested revisions are not feasible within the outlined constraints and therefore prefer to submit your paper elsewhere, we would welcome a message to this effect.

I look forward to seeing a revised form of your manuscript as soon as possible.

With kind regards,

Lise Roth

Lise Roth, Ph.D
Senior Editor
EMBO Molecular Medicine

When preparing your revised manuscript, please refer to our guidelines: https://link.springer.com/journal/44321/submission-guidelines#cms-Revised-submissions. We perform an initial quality control of all revised manuscripts before re-review; failure to include requested items will delay the evaluation of your revision.

We require:

1) A .docx formatted version of the manuscript text (including legends for main figures, EV figures and tables). Please make sure that the changes are highlighted to be clearly visible.

2) Individual production quality figure files as .eps, .tif, .jpg (one file per figure). For guidance, download the 'Figure Guide PDF': https://media.springernature.com/original/springer-cms/rest/v1/content/27825798/data/v1.

3) A .docx formatted letter INCLUDING the reviewers' reports and your detailed point-by-point responses to their comments. As part of the EMBO Press transparent editorial process, the point-by-point response is part of the Review Process File (RPF), which will be published alongside your paper.

4) A complete author checklist, which you can download from our author guidelines. Please insert information in the checklist that is also reflected in the manuscript. The completed author checklist will also be part of the RPF.

5) Please note that all corresponding authors are required to supply an ORCID ID for their name upon submission of a revised manuscript.

6) It is mandatory to include a 'Data Availability' section after the Materials and Methods. Before submitting your revision, primary datasets produced in this study need to be deposited in an appropriate public database, and the accession numbers and database listed under 'Data Availability'. Please remember to provide a reviewer password if the datasets are not yet public.

In case you have no data that requires deposition in a public database, please state so in this section. Note that the Data

Availability Section is restricted to new primary data that are part of this study.

7) For data quantification: please specify the name of the statistical test used to generate error bars and P values, the number (n) of independent experiments (specify technical or biological replicates) underlying each data point and the test used to calculate p-values in each figure legend. The figure legends should contain a basic description of n, P and the test applied. Graphs must include a description of the bars and the error bars (s.d., s.e.m.).

8) At EMBO Press we ask authors to provide source data for the main manuscript figures. You will receive a separate email with instructions for providing source data with your revised manuscript, including how to upload and organize the files.

9) Our journal encourages inclusion of *data citations in the reference list* to directly cite datasets that were re-used and obtained from public databases. Data citations in the article text are distinct from normal bibliographical citations and should directly link to the database records from which the data can be accessed. In the main text, data citations are formatted as follows: "Data ref: Smith et al, 2001" or "Data ref: NCBI Sequence Read Archive PRJNA342805, 2017". In the Reference list, data citations must be labeled with "[DATASET]". A data reference must provide the database name, accession number/identifiers and a resolvable link to the landing page from which the data can be accessed at the end of the reference.

10) We replaced Supplementary Information with Expanded View (EV) Figures and Tables that are collapsible/expandable online. A maximum of 5 EV Figures can be typeset. EV Figures should be cited as 'Figure EV1, Figure EV2" etc... in the text and their respective legends should be included in the main text after the legends of regular figures.

- For the figures that you do NOT wish to display as Expanded View figures, they should be bundled together with their legends in a single PDF file called *Appendix*, which should start with a short Table of Content. Appendix figures should be referred to in the main text as: "Appendix Figure S1, Appendix Figure S2" etc.

- Additional Tables/Datasets should be labeled and referred to as Table EV1, Dataset EV1, etc. Legends have to be provided in a separate tab in case of .xls files. Alternatively, the legend can be supplied as a separate text file (README) and zipped together with the Table/Dataset file.

11) The paper explained: EMBO Molecular Medicine articles are accompanied by a summary of the articles to emphasize the major findings in the paper and their medical implications for the non-specialist reader. Please provide a draft summary of your article highlighting
- the medical issue you are addressing,
- the results obtained and
- their clinical impact.

This may be edited to ensure that readers understand the significance and context of the research. Please refer to any of our published articles for an example.

12) Author contributions: You will be asked to provide CRediT (Contributor Role Taxonomy) terms in the submission system. These replace a narrative author contribution section in the manuscript.

13) A Conflict of Interest statement should be provided in the main text.

14) Every published paper includes a 'Synopsis' to further enhance discoverability. Synopses are displayed on the journal webpage and are freely accessible to all readers. They include a short stand first (maximum of 300 characters, including space) as well as 2-5 one-sentences bullet points that summarizes the paper. Please write the bullet points to summarize the key NEW findings. They should be designed to be complementary to the abstract - i.e. not repeat the same text. We encourage inclusion of key acronyms and quantitative information (maximum of 30 words / bullet point). Please use the passive voice. Please attach these in a separate file or send them by email, we will incorporate them accordingly.

Please also suggest a striking image or visual abstract to illustrate your article as a PNG file 550 px wide x 300-600 px high.

15) Include a Reagents and Tools Table as part of the Methods section, which can be downloaded from our author guidelines.

EMBO Molecular Medicine has a "scooping protection" policy, whereby similar findings that are published by others during review or revision are not a criterion for rejection. Should you decide to submit a revised version, I do ask that you get in touch after three months if you have not completed it, to update us on the status.

***** Reviewer's comments *****

Referee #1 (Comments on Novelty/Model System for Author):

Model used is adequate for the problem proposed.

Referee #1 (Remarks for Author):

The authors have satisfactorily responded to all prior criticism.

Referee #2 (Comments on Novelty/Model System for Author):

The authors have adequately discussed the paper's limitations and addressed all issues raised. The topic itself is significant and has the potential to advance the field and inform future research.

Referee #2 (Remarks for Author):

Although the manuscript was initially rejected, the authors carried out a thorough and detailed revision and provided adequate point-by-point responses to all issues raised.

Referee #3 (Remarks for Author):

This revised manuscript has partly addressed the previous comments, particularly adding new data validating some findings in more cell lines and analyzing additional public datasets. However, instead of providing numerous "transcriptomics" data without careful validation, the manuscript remains difficult to be understood. Most concerns from the previous review are not adequately addressed.

1. A major issue is the lack of mechanistic insights. How does ERN1-/- increase AR activity (Fig 1D)? and how does RB1 knockout almost completely diminish ERN1 expression but do not affect XBP1 expression (Fig 2J)? The authors failed to address why and how ERN1 loss confers castration/enzalutamide resistance and drives neuroendocrine reprogramming as well. These questions need to be experimentally addressed. In vivo validation of the role of IRE1 in castration resistance/progression with some animal experiments is also needed.

2. Conclusions are often over-stated and not supported by the presented data. For example, the title "IRE activity regulates tumour and microenvironment cell lineage states..." is over-stated. No data supports that the IRE activity regulates "microenvironment" cell lineage. Changes in IRE1 expression in stroma do not support "regulation of microenvironment cell lineage. On the other hand, the changes in cell proliferation/survival in Fig 1E, 1F, S4B,and 4D are marginal. Although they appear to be "significantly" different, it is not clear if these changes are sufficient to support castration/enzalutamide resistance. The scales of the Y-axis in Fig 4D/E use a range of 0.6 to 1.4, which is misleading. Line 160-161, "ERN-/- cells showed significant increase in AR mRNA levels and activity". No AR activity is measured for this experiment.

3. Fig 1A, the authors did not explain why the IRE1 activity is reduced in primary tumors as compared to normal tissues. The data indicate that at least a half of CRPC samples have a IRE1 level as high as the primary tumors, which do not support the statement that "IRE1 activity increased in primary disease while steadily decreasing in ...CRPC and NEPC" (line 120-121). It is not clear what Fig 1C really means.

4. To confirm the effects of ERN1-/-, it would be better to re-express a wildtype ERN1 in the cells to rule out off-target effects, but rather expressing the Q780 "dominant-negative" mutant. This is particularly important given that clonal variation in effects on cell proliferation and therapy responses is commonly acknowledged.

5. Most of the data are transcriptomics/qRT-PCR. The authors must do some Western blotting to demonstrate the changes in mRNA level, particularly the decrease in ERN1, RB1, and p53 protein levels in the used knockout cell lines and siRNA treatments. The ERN-/- cells appear to be obtained from another lab. Were the RB1-/- and p53-/- cells from the same lab? If not, how can the authors ensure they are isogenic?

6. Fig 2D-G, why investigated BRCA2? How the changes in this gene contribute to the effects of IRE1 loss? RB1 activity can be measured with expression of E2F1 genes. Several well-established E2F1 genes need to be investigated instead.

7. The authors often disregard inconsistent data when drawing their conclusions. For example, Fig 3D, Q780 did not increase SOX2 level as the same as ERN1-/-. Two ERN1 siRNA often showed difference in regulating gene expression. Fig 3H, siERN1.1 decreased CP expression but siERN1.2 increased CP expression in LNCaP cells; siERN1.1 did not alter CP level in C4-2B and PIGR in 22Rv1 cels, but siERN1.2 increased the expression of these genes in these cells. The authors need to show ERN1 protein level in these cells to ensure the siRNA work as expected.

8. Fig 4B, RB1 knockout almost completely depletes ERN1 expression (Fig 2J). If ERN1-/- cells were sensitize to androgen

depletion (Fig 1E), shouldn't RB1-/- cells respond to androgen depletion as well?

9. Fig S10A/B, the previous reviews asked to investigate if R1881 alters XBP1 binding to target genes. The authors did not address this question but rather looked at XBP1 chromatin binding in general. Fig S10A indicates that the average distance of the peaks to TSS is 10(5) (i.e., log10 = 5), which is more than 100kb. Can one define a region 100 kb away from transcription start sites as promoters?

10. Fig 5B, the two siRNAs often have opposite effects on gene expression. For example, DGAT2, VSTM2L. The off-target effects of siRNA cannot be ruled out.

Dear Dr Roth, Dear Lise,

Thank you very much once again for your continued support with this manuscript submission and the latest written communication which read:

"The manuscript has been reviewed by the three initial referees. As you will see below, while referees #1 and #2 are satisfied with the revisions, referee #3 has remaining concerns. After discussing the manuscript and the reports further within the team and with the referees, we agreed to invite a minor round of revisions, in which we would like you to adequately discuss and, where needed, tone down the conclusions, and conduct experiments for points 5 (WB), 6 (E2F1 genes) and 7 (ERN1 protein level)."

In the following letter I am addressing the points that we were asked to focus on which included:

1. Adequately discuss and tone down conclusions.
2. Carry out Western Blots to demonstrate changes in RB1, TP53, IRE1 in the cell lines deficient for IRE1, RB1 and TP53 and in parental lines treated with siRNA against ERN1 (points 5 and 7 of reviewer 3).
3. Measure the effect of IRE1 loss on cell cycling through markers other than BRCA2 (point 6 of reviewer 3).

Point 1: Adequately discuss and tone down conclusions.

The Discussion section has been rewritten (lines 561-638) to more accurately reflect both the limitations of this study as well as the scope for follow up investigations. For example, we mention the likely co-regulation of club markers by other UPR transducers (lines 602-604), the non-equivalence of IRE1 and RB1 (lines 610-611), the mRNA to protein level discordance (line 612), the modest albeit significant changes in ADT response through short treatments (line 609) and the histologically benign nature of club phenotypes (line 618). We then follow up with commentary on future work refining IRE1sig1.0 to reflect microenvironmental changes (line 586, 629), temporally mapping all UPR transducers on ADT responses (line 617), characterising the IRE1-loss lineage as a bona fide tumour cell type (line 622) and validating IRE1 modulation as differentiation therapy candidates *in vivo* (line 631).

In addition, we have included commentary in the Results section to reflect that different siRNAs do not equivalently downregulate IRE1 that may have an impact on the variability we see in some panels in the main figures (lines 291-293) and that sensitisation changes are modest (lines 349-352). We also altered the axis of figure 4D to start at 0.0 rather than 0.4 to fully illustrate the range of responses:

D.

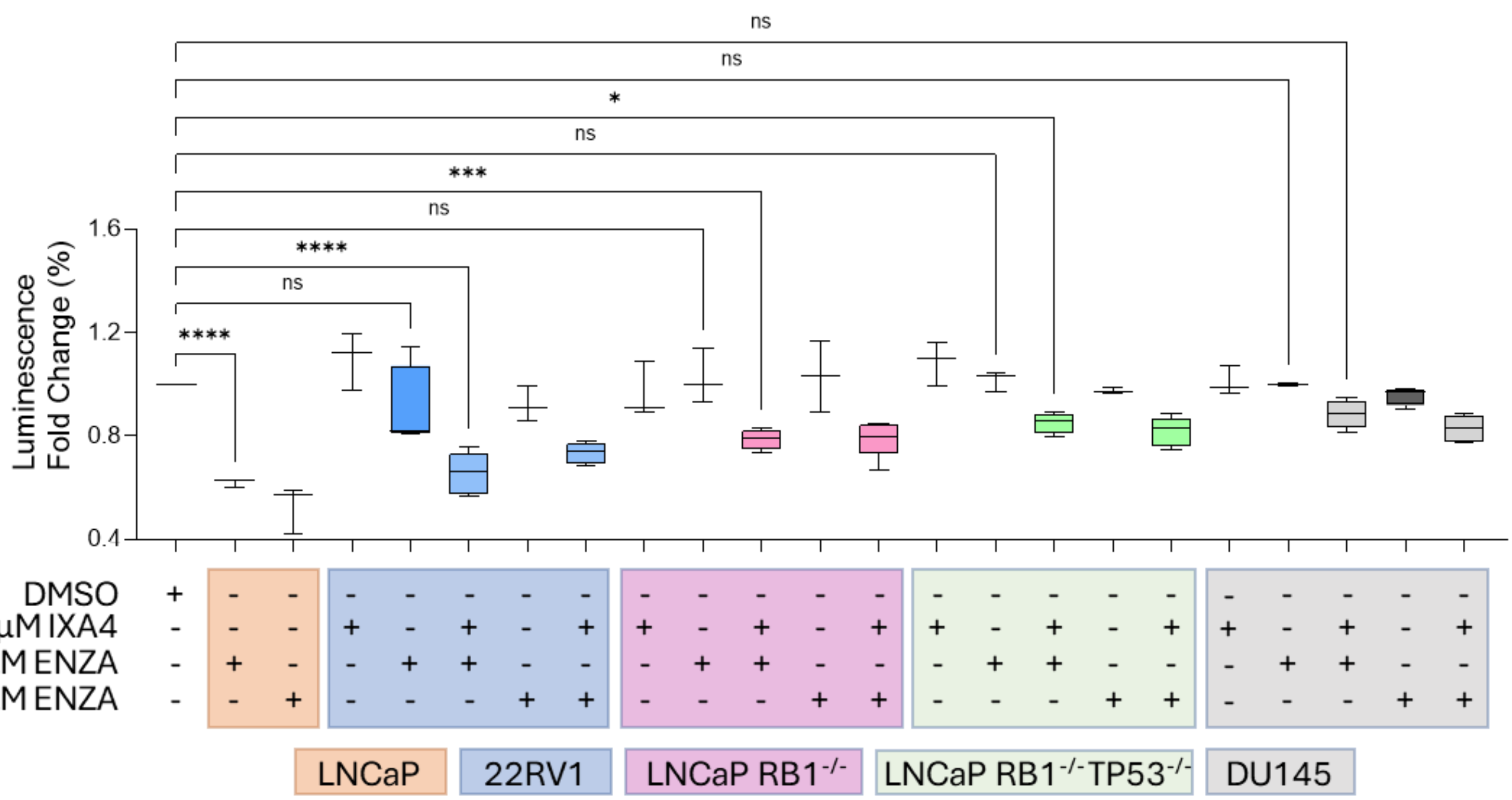

Point 2 and 3: Carry out Western Blots to demonstrate changes in RB1, TP53, IRE1 in the cell lines deficient for IRE1, RB1 and TP53 and in parental lines treated with siRNA against ERN1 (points 5 and 7 of reviewer 3). Measure the effect of IRE1 loss on cell cycling through markers other than BRCA2 (point 6 of reviewer 3).

We have carried out western blots to include E2F targets as well as TP53 and RB1 validation of the $RB1^{-/-}$ and $RB1^{-/-}/TP53^{-/-}$ cell lines and the IRE1 deficient lines. We blotted for IRE1, RB1, TP53, RBL1, E2F1, CCNE1, CCNA2, CDK4 and Actin and these results are presented in figure 2G:

G.

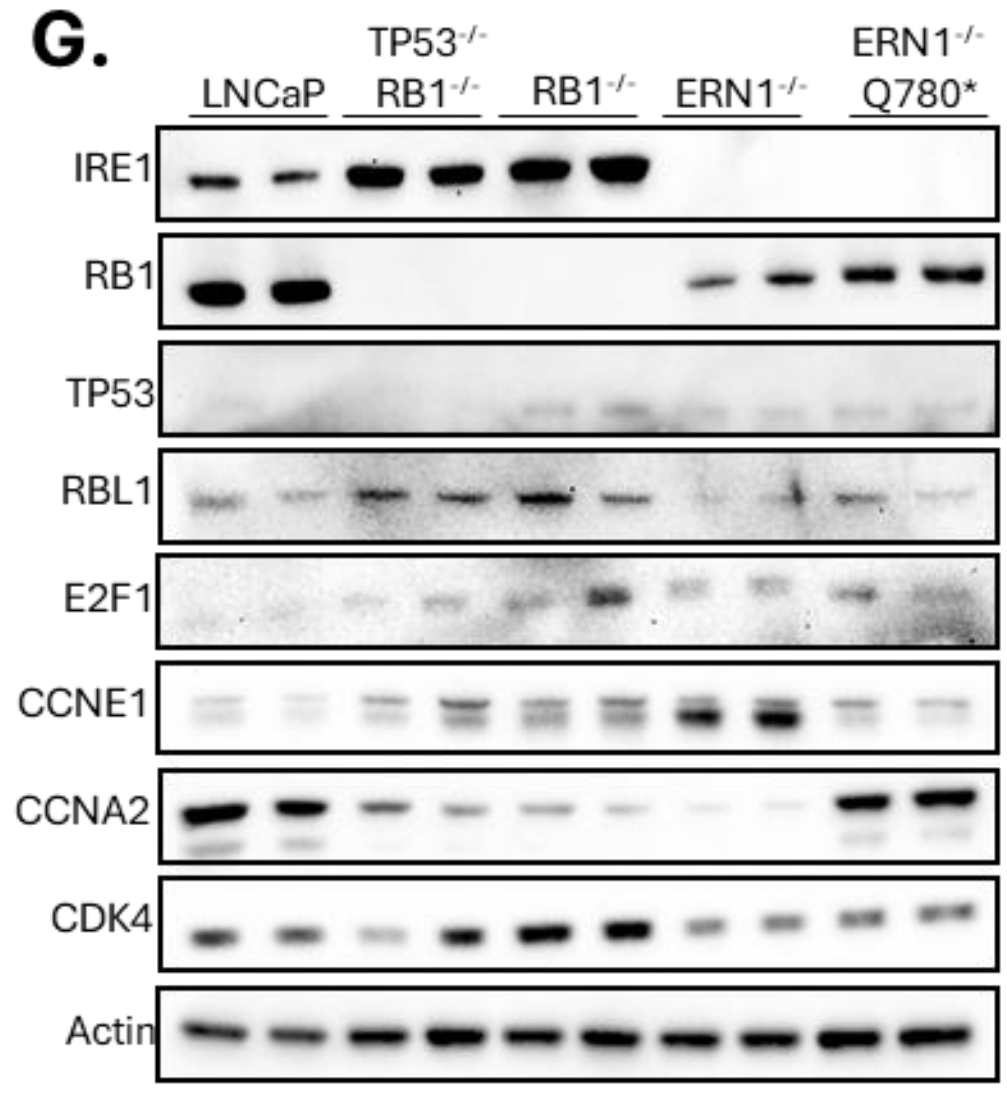

Densitometry of these results can be found in supplementary figure S5E:

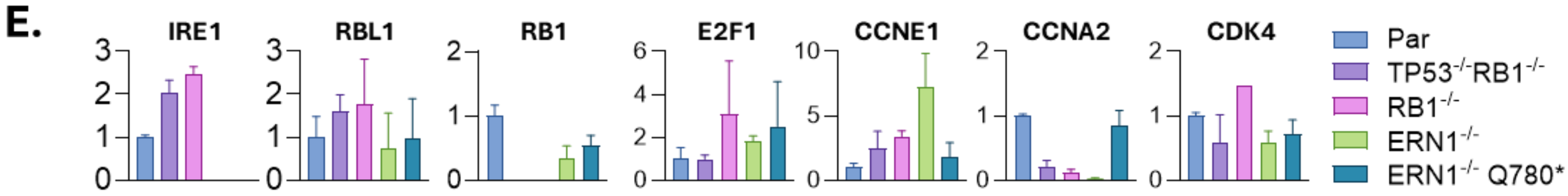

In addition, we included a heatmap of some of these markers in the RNAseq data of ERN1$^{-/-}$ cells compared to parental seen in figure 2H:

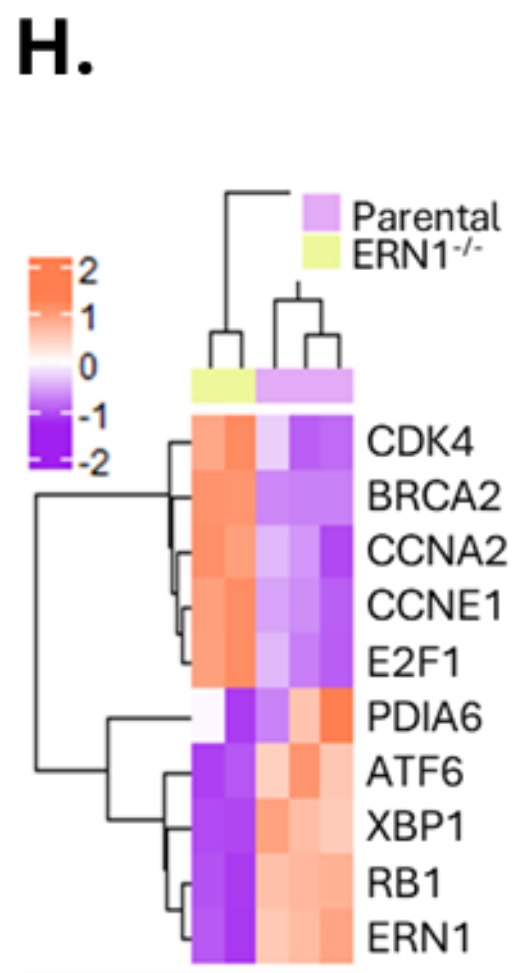

Finally, we blotted for IRE1 in all three parental cell lines (LNCaP, C42B, 22RV1) treated with siCTRL or the two separate siRNAs against ERN1. This can be found in supplementary figure S5A which does show that both siRNAs decrease IRE1 levels but not to the same degree.

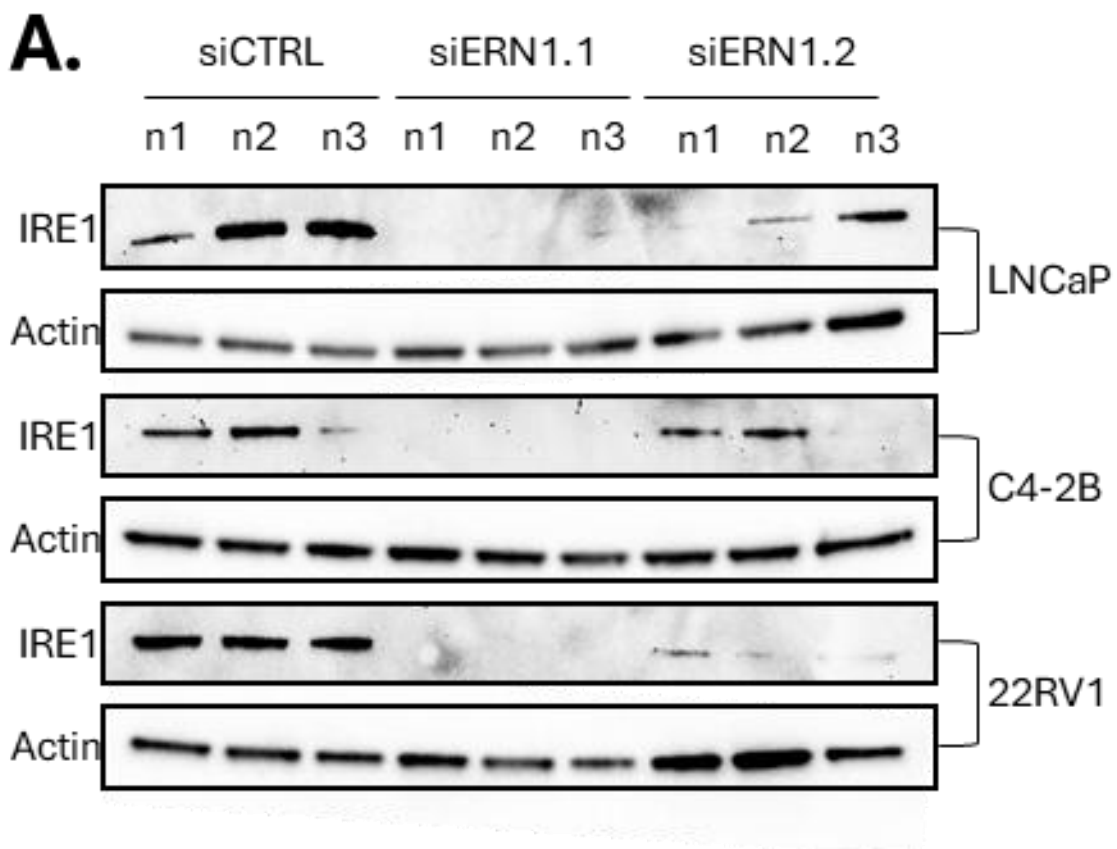

Additional changes:

1. To further document the reprogramming capacity of IRE1 deficient cells we carried out clonogenic experiments the results of which can be seen in figure 2L and supplementary figure S5F:

L.

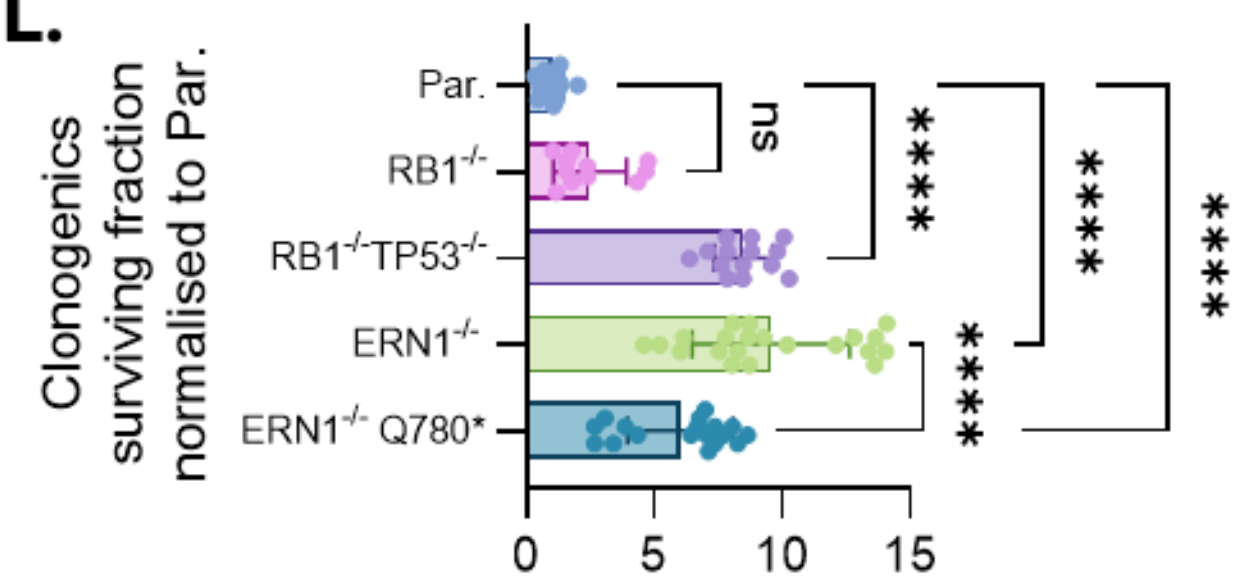

F.

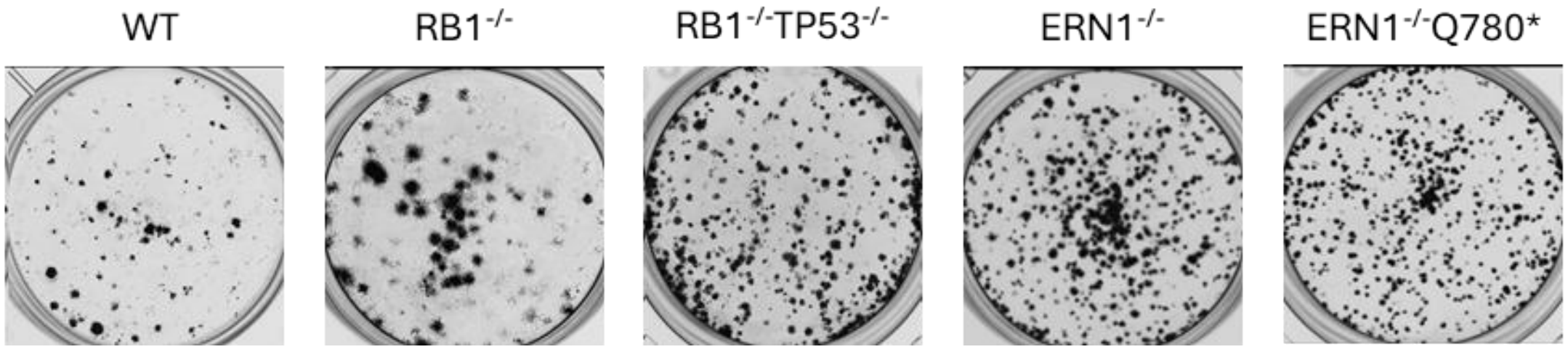

As a result, two co-authors have been added who contributed to this experiment and this is reflected in the author list (lines 3-6) and acknowledgments (lines 783-792).

2. Two further supplementary tables S33 and S34 have been included to document all exact statistical tests and p values represented by stars throughout the manuscript.
3. Further text changes that address comments and concerns include:
    a. Changing the title of the manuscript to better reflect the content removing the microenvironmental angle to “IRE1 activity prognosticates localised and metastatic prostate cancer and is a mediator of tumoral epithelial lineage control.”
    b. Changing AR levels and activity to AR and CAMKK2/KLK3 expression (lines 135,152).
    c. Fully explain the point of figure 1C (line 143-147) explaining the shift toward but not fully to AR independence.

We hope that these adequately address the reviewer comments. We remain at your disposal and are grateful for your assistance with this manuscript submission.

Sincerely,

Prof. Ian G. Mills.

Dr Dimitrios Doultsinos

26th May 2026

Dear Dr. Doultsinos, Dear Dimitrios,

Thank you for submitting your revised manuscript. Referee #2 has evaluated the revised version of your study. As you will see below, they are satisfied with the revisions, and I will therefore be able to accept your manuscript once the following editorial points have been addressed:

1/ Manuscript text:
- Please remove accept the previous changes, and only keep in track changes mode any new modification.
- Authors: Please add a note that author V. Macauley is deceased in the author list on the title page. Emails bounced for Cherlotte Bevan (Charlotte charlotte.bevan@imperial.ac.uk), Edward O'Neill (edward.oneil@nds.ox.ac.uk) and Imogen Bridges (imogen.bridges@ndm.ox.ac.uk), please check. Please add the authors' full names to the manuscript title page, they should be listed as they should be published. Please also correct the order of the author names in our system so that it matches how they are listed in the manuscript text.
- The manuscript sections should be in the following order: Title page - Abstract & Keywords - Introduction - Results - Discussion - Methods - Data Availability - Acknowledgments - Disclosure Statement & Competing Interests - References - Figure Legends - (Main Tables with legends if applicable) - Expanded View Figure Legends.
- Methods:
o Please upload a Reagents and Tools Table as an individual text file, which can be downloaded from our author guidelines.
o Cells: please indicate whether the cells were authenticated and tested for mycoplasma contamination and update the checklist accordingly.
o Patient samples: Include a statement confirming that informed consent was obtained from all subjects and that the experiments conformed to the principles set out in the WMA Declaration of Helsinki and the Department of Health and Human Services Belmont Report. If collected and within the bounds of privacy constraints report on age, sex and gender or ethnicity for all study participants.
o Remove the information regarding the use of BioRender from the figure legends and add a dedicated section to the Methods section, following this example:
Graphics:
(some of the... OR Figure #... OR synopsis) Graphics were created with BioRender.com.
- Please add a separate Data Availability section with URLs, and note that data must be deposited and publicly accessible before acceptance of the manuscript
- A "Disclosure and Competing Interests Statement" should be provided in the main text. E. Pilalis and A. Chatziioannou work for e-NIOS PC; M. Alshalalfa and E. Davicioni work for Veracyte; this should be mentioned in the Disclsoure Statement.
- Funding information should be merged with Acknowledgements, project numbers added if available (they should also be added to the list of funders in our system, which will be linked in PubMed after publication).
- Please remove authors contributions from the manuscript file and provide CRediT (Contributor Role Taxonomy) terms in the submission system instead. These replace a narrative author contribution section in the manuscript.
- References: In the text of the manuscript, a reference should be cited by author and year of publication; no more than two authors may be cited per reference; 'et al' should be used if there are more than two authors (i.e. Smith & Jones, 2003; Smith et al, 2000). In the reference list, citations should be listed in alphabetical order and then chronologically, with the authors' surnames and initials inverted; where there are more than 10 authors on a paper, 10 will be listed, followed by 'et al.'.
- Our journal encourages inclusion of *data citations in the reference list* to directly cite datasets that were re-used and obtained from public databases. Data citations in the article text are distinct from normal bibliographical citations and should directly link to the database records from which the data can be accessed. In the main text, data citations are formatted as follows: "Data ref: Smith et al, 2001" or "Data ref: NCBI Sequence Read Archive PRJNA342805, 2017". In the Reference list, data citations must be labeled with "[DATASET]". A data reference must provide the database name, accession number/identifiers and a resolvable link to the landing page from which the data can be accessed at the end of the reference.

2/ Figures:
- Please provide individual data points where possible.
- Add a numbers and short descriptions to each table in the manuscript text and move them after the figure legends. Also add a callout in the manuscript text so the typesetters will know where to place them.
- We replaced Supplementary Information with Expanded View (EV) Figures and Tables that are collapsible/expandable online. EV Figures should be cited as 'Figure EV1, Figure EV2" etc... in the text and their respective legends should be included in the main text after the legends of regular figures. For the figures that you do NOT wish to display as Expanded View figures, they should be bundled together with their legends in a single PDF file called *Appendix*, which should start with a short Table of Content. Appendix figures should be referred to in the main text as: "Appendix Figure S1, Appendix Figure S2" etc.
- Supplementary tables: they can remain in one file, but they should be renamed Dataset EV1- Dataset EV32.
- Please make sure that all figures and figure panels are referenced in the manuscript text. Currently, there is a callout for a Table S33 with p values, but this has not been uploaded. We suggest renaming it and adding it to the appendix with the

supplementary figures and renaming it Appendix Table S1. A callout is missing for Fig 3H.
- Figure reuse was found between Figure 4i and Figure 6B, Figure 3i and Appendix Figure S14B, Figure 7B and Appendix Figure S14B. Please clarify. Note that figure reuse is allowed, but should be mentioned in the figure legends.
- Please address the queries from our data editors in the figure legends:
o Please define the annotated p values ****/***/**/* as well as provide the exact p-values for the same in the legend of figure 1B, D; 2D, E, F, K, L; 3D, F, G, H; 4A, B, D; 5B-E, G, H as appropriate.
o Please note that the exact p values are not provided in the legend of figure 1E
o Please indicate the statistical test used for data analysis in the legends of figures 1B, D, G, H; 2B, D, E, F, K, L; 3A, D, F, G, H; 4A, B, D; 5B-E, G, H, L; 6G
o Please note that the box plots need to be defined in terms of minima, maxima, centre, bounds of box and whiskers, and percentile in the legends of figures 2B, 3A, 4A, D; 5G, H, L
o Please note that information related to n is missing in the legends of figures 1B, D, E, F; 2B, D, E, F, K, L; 3A, D, F, G, H; 4A, B, D; 5B-EG, H, L
o Please note that the error bars are not defined in the legends of figures 1B, D, E, F; 2D, E, F, K, L; 3D, F, G, H; 4B; 5B-E

3/ Checklist:
- Add the manuscript ID number in the top left corner
- Check and fill in if needed the following sections:
o Experimental study design and statistics
o Studies involving human participants.
o Human clinical and genomic datasets deposition in a public access-controlled repository in accordance to ethical obligations to the patients and to the applicable consent agreement?

4/ Source Data: Please provide the numerical raw data underlying your graphs. For the blots, please add annotations.

5/ The paper explained: EMBO Molecular Medicine articles are accompanied by a summary of the articles to emphasize the major findings in the paper and their medical implications for the non-specialist reader. Please provide a draft summary of your article highlighting
- the medical issue you are addressing,
- the results obtained and
- their clinical impact.

This may be edited to ensure that readers understand the significance and context of the research. Please refer to any of our published articles for an example.

6/ Please provide a synopsis text: Synopses are displayed on the journal webpage and are freely accessible to all readers. They include a short stand first (maximum of 300 characters, including space) as well as 2-5 one-sentences bullet points that summarizes the paper (maximum of 30 words / bullet point). They should be designed to be complementary to the abstract - i.e. not repeat the same text.

The visual abstract should be uploaded separately, as a jpeg/tiff or png file 550 px wide x 300 px high.

7/ As part of the EMBO Publications transparent editorial process initiative (see our Editorial at http://embomolmed.embopress.org/content/2/9/329), EMBO Molecular Medicine will publish online a Review Process File (RPF) to accompany accepted manuscripts.
This file will be published in conjunction with your paper and will include the anonymous referee reports, your point-by-point response and all pertinent correspondence relating to the manuscript. Let us know whether you agree with the publication of the RPF and as here, if you want to remove or not any figures from it prior to publication.
Please note that the Authors checklist will be published at the end of the RPF.

I look forward to receiving your revised manuscript.

With kind regards,

Lise

Lise Roth, PhD
Senior Editor
EMBO Molecular Medicine

***** Reviewer's comments *****

Referee #2 (Remarks for Author):

Is suitable for publication

The authors addressed the remaining editorial issues.

6th Jul 2026

Dear Dr. Doultsinos,

Thank you for submitting your revised files. I am pleased to inform you that your manuscript is now accepted for publication in EMBO Molecular Medicine!

Please note that I still found issues in your references. Kindly refer to our guidelines and correct as appropriate (https://link.springer.com/journal/44321/submission-guidelines#cms-Reference-guidelines).
In particular:

This study has been published and the reference should be updated:
Almanza A, Carlesso A, Chintha C, Creedican S, Doultsinos D, Leuzzi B, Luís A, McCarthy N, Montibeller L, More S, et al (2019) Endoplasmic reticulum stress signalling - from basic mechanisms to clinical applications. FEBS Journal 286: 241-278 doi:10.1111/febs.14608 [PREPRINT]

These studies are preprints and should be labeled as such:
Clinical testing of transcriptome-wide expression profiles in high-risk localized and metastatic prostate cancer starting androgen deprivation therapy: an ancillary study of the STAMPEDE abiraterone Phase 3 trial.

Doultsinos D, McMahon M, Voutetakis K, Obacz J, Pineau R, Le Reste P-J, Obiedat A, Samal J, Patterson JB, Zheng Q, et al (2019) Constitutive UPRER activation sustains tumor cell differentiation. bioRxiv

Song H, Xu J, Velazquez-Arcelay K, Demirci A, Raizenne BL, Hsu SC, Choi J, Pham JH, Chen Y-A, Weinstein HNW, et al (2026) A single-cell and spatial atlas of prostate cancer reveals the combinatorial nature of gene modules underlying lineage plasticity and metastasis. bioRxiv: 2026.03.25.711335

Once this is done, please send me your manuscript file via email and we'll transfer the files to our publisher.

Your manuscript will be processed for publication by EMBO Press. It will be copy edited and you will receive page proofs prior to publication. Please note that you will be contacted by Springer Nature Author Services to complete licensing and payment information.

You may qualify for financial assistance for your publication charges - either via a Springer Nature fully open access agreement or an EMBO initiative. Check your eligibility: https://link.springer.com/journal/44321/how-to-publish-with-us

Should you be planning a Press Release on your article, please notify the production team via this form in order to coordinate publication and release dates: https://forms.cloud.microsoft/e/eH4A8JVP6g

If you have any questions, please do not hesitate to contact the Editorial Office. Thank you for your contribution to EMBO Molecular Medicine.

With kind regards,

Lise

Lise Roth, Ph.D
Senior Editor
EMBO Molecular Medicine

>>> Please note that it is EMBO Molecular Medicine policy for the transcript of the editorial process (containing referee reports and your response letter) to be published as an online supplement to each paper. If you do NOT want this, you will need to inform the Editorial Office via email immediately. More information is available here: https://link.springer.com/partners/embo-press/editorial-policies#Peer%20review

EMBO Press Author Checklist

Corresponding Author Name: Dr Dimitrios Doultsinos
Journal Submitted to: EMBO Molecular Medicine
Manuscript Number: EMM-2025-22052

USEFUL LINKS FOR COMPLETING THIS FORM
The EMBO Journal - Author Guidelines
EMBO Reports - Author Guidelines
Molecular Systems Biology - Author Guidelines
EMBO Molecular Medicine - Author Guidelines

Reporting Checklist for Life Science Articles (updated January
This checklist is adapted from Materials Design Analysis Reporting (MDAR) Checklist for Authors. MDAR establishes a minimum set of requirements in transparent reporting in the life sciences (see Statement of Task: 10.31222/osf.io/9sm4x). Please follow the journal's guidelines in preparing your
Please note that a copy of this checklist will be published alongside your article.

Abridged guidelines for figures
1. Data
The data shown in figures should satisfy the following conditions:
- the data were obtained and processed according to the field's best practice and are presented to reflect the results of the experiments in an accurate and unbiased manner.
- ideally, figure panels should include only measurements that are directly comparable to each other and obtained with the same assay.
- plots include clearly labeled error bars for independent experiments and sample sizes. Unless justified, error bars should not be shown for technical
- if n<5, the individual data points from each experiment should be plotted. Any statistical test employed should be justified.
- Source Data should be included to report the data underlying figures according to the guidelines set out in the authorship guidelines on Data

2. Captions
Each figure caption should contain the following information, for each panel where they are relevant:
- a specification of the experimental system investigated (eg cell line, species name).
- the assay(s) and method(s) used to carry out the reported observations and measurements.
- an explicit mention of the biological and chemical entity(ies) that are being measured.
- an explicit mention of the biological and chemical entity(ies) that are altered/varied/perturbed in a controlled manner.
- the exact sample size (n) for each experimental group/condition, given as a number, not a range;
- a description of the sample collection allowing the reader to understand whether the samples represent technical or biological replicates (including how many animals, litters, cultures, etc.).
- a statement of how many times the experiment shown was independently replicated in the laboratory.
- definitions of statistical methods and measures:
  - common tests, such as t-test (please specify whether paired vs. unpaired), simple χ^2 tests, Wilcoxon and Mann-Whitney tests, can be unambiguously identified by name only, but more complex techniques should be described in the methods section;
  - are tests one-sided or two-sided?
  - are there adjustments for multiple comparisons?
  - exact statistical test results, e.g., P values = x but not P values < x;
  - definition of 'center values' as median or average;
  - definition of error bars as s.d. or s.e.m.

Please complete ALL of the questions below.
Select "Not Applicable" only when the requested information is not relevant for your study.

Materials

Newly Created Materials	Information included in the manuscript?	In which section is the information available? (Reagents and Tools Table, Materials and Methods, Figures, Data Availability Section)
New materials and reagents need to be available; do any restrictions apply?	Yes	Cell lines and datasets in materials and methods section

Antibodies	Information included in the manuscript?	In which section is the information available? (Reagents and Tools Table, Materials and Methods, Figures, Data Availability Section)
For **antibodies** provide the following information:<br>- Commercial antibodies: RRID (if possible) or supplier name, catalogue number and or/clone number<br>- Non-commercial: RRID or citation	Yes	Materials and Methods

DNA and RNA sequences	Information included in the manuscript?	In which section is the information available? (Reagents and Tools Table, Materials and Methods, Figures, Data Availability Section)
**Short novel DNA or RNA including primers, probes**: provide the sequences.	Yes	Materials and Methods

Cell materials	Information included in the manuscript?	In which section is the information available? (Reagents and Tools Table, Materials and Methods, Figures, Data Availability Section)
**Cell lines:** Provide species information, strain. Provide accession number in repository **OR** supplier name, catalog number, clone number, and/**OR** RRID.	Yes	Commercial lines acquired from ATCC. Donated lines in the acknowledgment section and materials and methods
**Primary cultures:** Provide species, strain, sex of origin, genetic modification status.	Not Applicable	
Report if the cell lines were recently **authenticated** (e.g., by STR profiling) and tested for mycoplasma contamination.	Yes	Routinely mycoplasma tested. Mycoplasma negative

Experimental animals	Information included in the manuscript?	In which section is the information available? (Reagents and Tools Table, Materials and Methods, Figures, Data Availability Section)
**Laboratory animals or Model organisms:** Provide species, strain, sex, age, genetic modification status. Provide accession number in repository **OR** supplier name, catalog number, clone number, **OR** RRID.	Not Applicable	
**Animal observed in or captured from the field:** Provide species, sex, and age where possible.	Not Applicable	
Please detail **housing and husbandry conditions**.	Not Applicable	

Plants and microbes	Information included in the manuscript?	In which section is the information available? (Reagents and Tools Table, Materials and Methods, Figures, Data Availability Section)
**Plants:** provide species and strain, ecotype and cultivar where relevant, unique accession number if available, and source (including location for collected wild specimens).	Not Applicable	
**Microbes:** provide species and strain, unique accession number if available, and source.	Not Applicable	

Human research participants	Information included in the manuscript?	In which section is the information available? (Reagents and Tools Table, Materials and Methods, Figures, Data Availability Section)
If collected and within the bounds of privacy constraints report on age, sex and gender or ethnicity for all study participants.	Not Applicable	

Core facilities	Information included in the manuscript?	In which section is the information available? (Reagents and Tools Table, Materials and Methods, Figures, Data Availability Section)
If your work benefited from core facilities, was their service mentioned in the acknowledgments section?	Not Applicable	

Design

Study protocol	Information included in the manuscript?	In which section is the information available? (Reagents and Tools Table, Materials and Methods, Figures, Data Availability Section)
If study protocol has been **pre-registered, provide DOI in the manuscript**. For clinical trials, provide the trial registration number **OR** cite DOI.	Not Applicable	
Report the **clinical trial registration number** (at ClinicalTrials.gov or equivalent), where applicable.	Not Applicable	

Laboratory protocol	Information included in the manuscript?	In which section is the information available? (Reagents and Tools Table, Materials and Methods, Figures, Data Availability Section)
Provide DOI OR other citation details if **external detailed step-by-step protocols** are available.	Not Applicable	

Experimental study design and statistics	Information included in the manuscript?	In which section is the information available? (Reagents and Tools Table, Materials and Methods, Figures, Data Availability Section)
Include a statement about **sample size** estimate even if no statistical methods were used.	Yes	Throughout figure legends, supplementary tables 33 and 34, and materials and methods
Were any steps taken to minimize the effects of subjective bias when allocating animals/samples to treatment (e.g. **randomization procedure**)? If yes, have they been described?	Not Applicable	
Include a statement about **blinding** even if no blinding was done.	Not Applicable	
Describe **inclusion/exclusion criteria** if samples or animals were excluded from the analysis. Were the criteria pre-established?<br><br>If sample or data points were omitted from analysis, report if this was due to attrition or intentional exclusion and provide justification.	Not Applicable	
For every figure, are **statistical tests** justified as appropriate? Do the data meet the assumptions of the tests (e.g., normal distribution)? Describe any methods used to assess it. Is there an estimate of variation within each group of data? Is the variance similar between the groups that are being statistically compared?	Yes	Supplementrary tables 33 and 34 and materials and methods

Sample definition and in-laboratory replication	Information included in the manuscript?	In which section is the information available? (Reagents and Tools Table, Materials and Methods, Figures, Data Availability Section)
In the figure legends: state number of times the experiment was **replicated** in laboratory.	Yes	Figure legends throughout
In the figure legends: define whether data describe **technical or biological replicates**.	Yes	Figure legends throughout

Ethics

Ethics	Information included in the manuscript?	In which section is the information available? (Reagents and Tools Table, Materials and Methods, Figures, Data Availability Section)
Studies involving **human participants**: State details of **authority granting ethics approval** (IRB or equivalent committee(s), provide reference number for approval.	Yes	Materials and methods. Imperial College Healthcare Tissue Bank (REC 22/WA/0214)
Studies involving **human participants**: Include a statement confirming that **informed consent** was obtained from all subjects and that the experiments conformed to the principles set out in the WMA Declaration of Helsinki and the Department of Health and Human Services Belmont Report.	Yes	Materials and methods -> Patient Samples
Studies involving **human participants: For publication of patient photos**, include a statement confirming that consent to publish was obtained.	Not Applicable	
Studies involving experimental **animals**: State details of **authority granting ethics approval** (IRB or equivalent committee(s), provide reference number for approval. Include a statement of compliance with ethical regulations.	Not Applicable	
Studies involving **specimen and field samples:** State if relevant **permits** obtained, provide details of authority approving study; if none were required, explain why.	Not Applicable	

Dual Use Research of Concern (DURC)	Information included in the manuscript?	In which section is the information available? (Reagents and Tools Table, Materials and Methods, Figures, Data Availability Section)
Could your study fall under dual use research restrictions? Please check biosecurity documents and list of **select agents and toxins** (CDC): https://www.selectagents.gov/sat/list.htm	Not Applicable	
If you used a select agent, is the security level of the lab appropriate and reported in the manuscript?	Not Applicable	
If a study is subject to dual use research of concern regulations, is the name of the **authority granting approval and reference number** for the regulatory approval provided in the manuscript?	Not Applicable	

Reporting

The MDAR framework recommends adoption of discipline-specific guidelines, established and endorsed through community initiatives. Journals have their own policy about requiring specific guidelines and recommendations to complement MDAR.

Adherence to community standards	Information included in the manuscript?	In which section is the information available? (Reagents and Tools Table, Materials and Methods, Figures, Data Availability Section)
State if relevant guidelines or checklists (e.g., **ICMJE, MIBBI, ARRIVE, PRISMA**) have been followed or provided.	Not Applicable	
For **tumor marker prognostic studies,** we recommend that you follow the **REMARK** reporting guidelines (see link list at top right). See author guidelines, under 'Reporting Guidelines'. Please confirm you have followed these guidelines.	Not Applicable	
For **phase II and III randomized controlled trials**, please refer to the **CONSORT** flow diagram (see link list at top right) and submit the CONSORT checklist (see link list at top right) with your submission. See author guidelines, under 'Reporting Guidelines'. Please confirm you have submitted this list.	Not Applicable	

Data Availability

Data availability	Information included in the manuscript?	In which section is the information available? (Reagents and Tools Table, Materials and Methods, Figures, Data Availability Section)
Have **primary datasets** been deposited according to the journal's guidelines (see 'Data Deposition' section) and the respective accession numbers provided in the Data Availability Section?	Yes	Biostudies and GEO. Materials and Methods
Were **human clinical and genomic datasets** deposited in a public access-controlled repository in accordance to ethical obligations to the patients and to the applicable consent agreement?	Not Applicable	
Are **computational models** that are central and integral to a study available without restrictions in a machine-readable form? Were the relevant accession numbers or links provided?	Not Applicable	
If publicly available data were reused, provide the respective **data citations in the reference list**.	Yes	Materials and Methods and References section
